# Comparison of dorsal root ganglion gene expression in rat models of traumatic and HIV-associated neuropathic pain

**DOI:** 10.1016/j.ejpain.2008.05.011

**Published:** 2009-04

**Authors:** Klio Maratou, Victoria C.J. Wallace, Fauzia S. Hasnie, Kenji Okuse, Ramine Hosseini, Nipurna Jina, Julie Blackbeard, Timothy Pheby, Christine Orengo, Anthony H. Dickenson, Stephen B. McMahon, Andrew S.C. Rice

**Affiliations:** a Pain Research Group, Department of Anaesthetics, Pain Medicine and Intensive Care, Faculty of Medicine, Imperial College London, Chelsea and Westminster Hospital Campus, London SW10 9NH, UK; b Physiological Genomics and Medicine Group, MRC Clinical Sciences Centre, Du Cane Road, W12 0NN, UK; c Division of Cell and Molecular Biology, Faculty of Natural Sciences, Imperial College London, London SW7 2AZ, UK; d Department of Anatomy and Developmental Biology, University College London, London WC1E 6BT, UK; e ICH Gene Microarray Centre, Institute of Child Health, University College London, London WC1N 1EH, UK; f Biomolecular Structure and Modelling Unit, Department of Biochemistry and Molecular Biology, University College London, London WC1E 6BT, UK; g Department of Neuropharmacology, University College London, London WC1E 6BT, UK; h Department of Anatomy and Human Sciences, Kings College London, Guy’s Hospital Campus, London SE1 1UL, UK

**Keywords:** Neuropathic pain, HIV, Mechanical hypersensitivity, Microarray

## Abstract

To elucidate the mechanisms underlying peripheral neuropathic pain in the context of HIV infection and antiretroviral therapy, we measured gene expression in dorsal root ganglia (DRG) of rats subjected to systemic treatment with the anti-retroviral agent, ddC (Zalcitabine) and concomitant delivery of HIV-gp120 to the rat sciatic nerve. L4 and L5 DRGs were collected at day 14 (time of peak behavioural change) and changes in gene expression were measured using Affymetrix whole genome rat arrays. Conventional analysis of this data set and Gene Set Enrichment Analysis (GSEA) was performed to discover biological processes altered in this model. Transcripts associated with G protein coupled receptor signalling and cell adhesion were enriched in the treated animals, while ribosomal proteins and proteasome pathways were associated with gene down-regulation. To identify genes that are directly relevant to neuropathic mechanical hypersensitivity, as opposed to epiphenomena associated with other aspects of the response to a sciatic nerve lesion, we compared the gp120 + ddC-evoked gene expression with that observed in a model of traumatic neuropathic pain (L5 spinal nerve transection), where hypersensitivity to a static mechanical stimulus is also observed. We identified 39 genes/expressed sequence tags that are differentially expressed in the same direction in both models. Most of these have not previously been implicated in mechanical hypersensitivity and may represent novel targets for therapeutic intervention. As an external control, the RNA expression of three genes was examined by RT-PCR, while the protein levels of two were studied using western blot analysis.

## 1 Introduction

Peripheral nerve disorders are frequent complications of HIV disease. Distal symmetrical polyneuropathy (DSP) is the most common peripheral nerve disorder associated with HIV (Verma et al., 2005). About 40% of ambulatory HIV patients in the developed world population have peripheral neuropathy, a prevalence which has not altered with the advent of antiretroviral therapy (Smyth et al., 2007). Of these 74–93% have pain, which is severe in 37–43% of patients. In addition, 52% of people living with advanced HIV have peripheral neuropathy associated with symptoms such as pain (Simpson et al., 2006). The most common clinical features of DSP are measurable sensory abnormalities, most noticeably loss of sensitivity to thermal stimuli (Martin et al., 2003) and on-going, paroxysmal or stimulus evoked pain (Dalakas and Pezeshkpour, 1988, Martin et al., 1999). There are two predominant (and clinically similar) settings in which painful DSP may occur in HIV disease. First, a disease-related DSP associated with HIV disease, likely involving virally mediated mechanisms such as the interaction of the coat protein, gp120, with sensory neurones precipitating chemokine mediated neuronal damage (Oh et al., 2001, Keswani et al., 2003b, Melli et al., 2006, Wallace et al., 2007a) and; second a drug-induced DSP, particularly associated with the use of nucleoside reverse transcriptase inhibitors as part of an anti-retroviral therapy (Cherry et al., 2003, Cherry et al., 2006, Wallace et al., 2007b).

We have characterized a model of HIV-related neuropathy, termed gp120 + ddC (Wallace et al., 2007b), which combines perineural administration of the HIV-1 coat protein, glycoprotein 120 (gp120), with systemic treatment with the nucleoside reverse transcriptase inhibitor, 2′, 3′-dideoxycytidine (ddC/Zalcitabine). Gp120 + ddC treatment is associated with development of hypersensitivity to static punctate mechanical stimuli, without hypersensitivity to heat or cold, and thigmotaxis (anxiety-like behaviour) (Wallace et al., 2007b). This model represents one clinical scenario of DSP in the HIV patient treated with nucleoside reverse transcriptase inhibitors, whereby the sensory neurones are damaged by a combination of gp120 and ddC effects (Cherry et al., 2003). In this study, the gp120 + ddC model is used to elucidate the pathophysiology of DSP using gene microarrays of dorsal root ganglions.

To distinguish between genes that are directly related to the sensory dysfunction observed in the gp120 + ddC model and those that are related to other aspects of the pathophysiological response to a nerve lesion, we compared the gp120 + ddC-evoked gene expression with that measured in a model of traumatic neuropathic pain, the L5 spinal nerve transection model (L5 SNT), where hypersensitivity to a static mechanical stimulus is also observed. This is a well established and commonly employed model of peripheral nerve lesion in rats and results in hypersensitivity to mechanical, cold and heat stimuli. (Kim and Chung, 1992, Choi et al., 1994, Ringkamp et al., 1999, Bridges et al., 2001). Variants on this model have been utilized in previous microarray investigations (Wang et al., 2002, Valder et al., 2003, Lacroix-Fralish et al., 2006, Levin et al., 2007) making this an appropriate comparator model for this study. By comparing gene expression profiles in these two distinct models, we aim to highlight genes associated with HIV-related neuropathy as well as mechanical hypersensitivity in neuropathic pain conditions in general.

## 2 Methods

### 2.1 Animals

All experiments conformed to the British Home Office Regulations and IASP guidelines (Zimmermann, 1983). Male Wistar rats weighing 200-250 g at baseline were used for all experiments (B&K, Hull, UK) and were housed in a temperature-controlled environment, maintained on a 14:10 h light–dark cycle (experiments were performed during the light phase) and provided with feed and water *ad libitum*.

### 2.2 Experimental animals

#### 2.2.1 Spinal nerve transection model (SNT)

Surgery was performed under general anaesthesia (1–2% isoflurane in 1% O_2_ and 1% N_2_0), using standard aseptic techniques. SNT (*n* = 12) surgery was performed as previously described (Bridges et al., 2001). Using the transverse processes of L6 as a guide, the left paraspinal muscles were exposed and separated from the spinous processes. The L6 transverse process was then removed by hemi-laminectomy and the L5 spinal nerve exposed, ligated tightly with a 3–0 silk suture, and cut 1 ± 2 mm distal to the suture. Sham surgery (*n* = 12) was performed by exposing the L5 spinal nerve as described above, but not damaging it.

#### 2.2.2 Gp120 + ddC model

Rats in this model were treated as described by Wallace and colleagues (2007b). In eight anaesthetized (1–2% isoflurane in 1% O_2_ and 1% N_2_0) rats the left sciatic nerve was exposed to gp120 by placing a 5 mm × 2 mm piece of gel foam soaked in saline containing 200 ng HIV-1 gp120-MN (>95% pure; Immunodiagnostics, Bedford, MA, USA) in direct contact with the nerve, to form a pool of protein solution around the nerve that is left in place for 30 min. Following this, oxidized regenerated cellulose (Surgicell, Ethicon); previously soaked until saturation in the same saline-gp120 solution, was wrapped loosely around the sciatic nerve 2-3 mm proximal to the trifurcation so as not to cause any nerve constriction and left *in situ*. The nerve was gently manipulated back into place and the muscle and skin incisions closed with 4/0 silk sutures. Animals were injected on the day of surgery with ddC (Roche, Basel, Switzerland; 50 mg/kg in saline, i.p.) and injected thereafter three times per week. Fourteen days after the day of surgery animals were sacrificed and DRG tissue was collected. Sham surgery (*n* = 8) was performed by exposing the left sciatic nerve as described above, but exposing it to 0.1% rat serum albumin. The sham group also received saline as a control for ddC.

#### 2.2.3 Varicella Zoster Virus (VZV) infection model

We used an additional model of neuropathic pain, the VZV infection model, for validation of the differential expression of three of our candidate genes by RT-PCR and immunoblot analysis. Rats in this model were treated as previously described by Hasnie and colleagues (2007). In brief, VZV (strain Dumas) was propagated on fibroblast (primary human embryonic lung) cells and harvested when cells exhibited 80% cytopathic effect (cpe) (equivalent to 10^4^–10^5^ plaque forming units). Animals (*n* = 16) were subcutaneously injected with 50 μl viral inoculum into the mid-plantar glabrous footpad of the left hind limb. Control animals (*n* = 16) received similar injection of uninfected fibroblast cells. Fourteen days after the injection, animals were sacrificed and L4 and L5 DRG tissue was harvested.

### 2.3 Behavioural reflex testing

Punctate mechanical hypersensitivity was assessed using graded “von Frey” nylon filaments (Alan Ainsworth, UCL, London), which were used to deliver a calibrated indentation pressure against the hairless skin of the hind paws. The withdrawal threshold was determined by sequentially increasing and decreasing the stimulus strength and threshold response was defined by the nominal bending force of the filament that evoked foot withdrawal at least three times in every five applications when delivered at a rate of 1 Hz (Chaplan et al., 1994). Baseline measurements were obtained for all animals on two separate occasions within the week prior to surgery. Following surgery, behavioural reflex tests were carried out in a blinded manner. Animals were re-tested on day 14 post-surgery/infection and only animals demonstrating a significant (paired *t*-test, *p* < 0.05) reduction in ipsilateral PWT compared to baseline values were retained for microarray analysis.

### 2.4 Tissue collection and microarray

Animals were sacrificed by administration of an overdose of pentobarbitone (100 mg/kg Animalcare Ltd., York, UK). Ipsilateral DRG from lumbar segments L4 and L5 were dissected on ice, and snap frozen in liquid nitrogen. Transection of the L5 spinal nerve in the SNT model was confirmed on post-mortem and only these animals were included in the microarray analysis. Prior to RNA preparation, for the gp120 + ddC model, ipsilateral L4 and L5 DRGs from two animals were pooled, to provide enough tissue for RNA preparation. For the SNT model, only ipsilateral L5 DRGs from three animals were collected and pooled. Four replicates per condition were prepared. Total RNA was extracted using TRIzol reagent (Invitrogen, Carlsband, CA, USA), and further purified using an RNeasy Mini kit (Qiagen, Crawley, UK), according to the manufacturer protocols. Affymetrix GeneChip® Rat Genome 230 2.0 arrays (Santa Clara, CA, USA) that study over 30,000 transcripts and variants from over 28,000 well-substantiated rat genes, were used for these experiments. Biotin labeled cRNA was generated using the Affymetrix Small Sample Labeling Protocol vII (http://www.affymetrix.com). Approximately 200 ng total starting RNA was used for each sample. Labeled cRNA fragmentation, as well as array hybridization, washing, and staining were performed as described in the Affymetrix GeneChip® Expression Analysis Technical Manual (http://www.affymetrix.com).

### 2.5 Data analyses

CEL files were obtained with Affymetrix Microarray Suite software. Data was analysed using R v2.3.1 and Bioconductor v1.8 packages (Gentleman et al., 2004) as follows: Quality control tests and RMA data normalization were performed using Simpleaffy (Wilson and Miller, 2005) and Affy (Gautier et al., 2004). The normalized data was filtered, using Genefilter (http://www.bioconductor.org/packages/1.8/bioc/html/genefilter.html), to remove probe sets with minimal expression levels (i.e., probe sets failing to have a signal higher than log_2_(100) in three or more arrays). Statistical analysis was performed using linear models and empirical Bayes methods implemented in the Limma package (Smyth, 2004, Smyth, 2005). Benjamini and Hochberg’s step-up method (Reiner et al., 2003) with false discovery rate (FDR) less than 0.1 was used to control for multiple testing. The threshold *p* value consistent with an FDR near 10% was identified as 0.03 for the SNT model (10.4% FDR) and 0.004 for the gp120 + ddC model (9.6% FDR). The lists of statistically significant genes were loaded into GeneSpring GX (v7.3.1) software (Agilent Technologies, Cheshire, UK), where a second filter (fold difference less than 1.2-fold) was applied to further reduce false positive results (Bakay et al., 2002). We chose 1.2-fold change, which is a moderate cut-off, to signify differential expression, because the two cycle amplification protocol used in this study is thought to suppress fold differences (see discussion). Finally, Venn diagrams were used to cross-compare data between models. The microarray data is available in MIAME-compliant (minimum information about a microarray experiment) format at the ArrayExpress database (http://www.ebi.ac.uk/arrayexpress) (Parkinson et al., 2007) under accession codes E-MEXP-974, E-MEXP-976.

#### 2.5.1 Functional association analysis

Associations with the annotations of the Gene Ontology (GO) Consortium (Ashburner et al., 2000) were obtained, for the lists of significant probe sets (10% FDR and over 1.2-fold difference) that correspond to each model, using MAPPFinder 2.0, a part of the GenMAPP 2.1 application package (Dahlquist et al., 2002, Doniger et al., 2003). To ease the interpretation of results, output data were manually filtered, using criteria used by Doniger and colleagues (2003), to remove terms that represented the same genes (typically parent–child processes). For a process to be included in the results, it was required that the *z* score from the MAPPFinder statistics was higher than 2, with a permute *p* value less than 0.01, and that at least one gene changed significantly for this node (local results). Also, terms that (a) comprised of 5 or less genes; or (b) had more than 200 genes changed (nested results) were removed, because they were either too specific or too general for the data interpretation.

Pathway analysis was also performed using Gene Set Enrichment Analysis (GSEA) version 2.0 (Subramanian et al., 2005, Subramanian et al., 2007). A total of 253 gene sets were applied. These were obtained from the C2/Canonical Pathways collection of MSigDB version 2.1 (Subramanian et al., 2005), which contains gene sets collected from various sources such as online pathway databases, publications in PubMed, and knowledge of domain experts. Fourteen additional gene sets were generated by querying the Affymetrix NetAffx tool (https://www.affymetrix.com/analysis/netaffx/index.affx) with pain related key words. GSEA was run with default settings by using the gene_set permutation option and performing 1000 gene permutations for the determination of statistical significance. Significant FDR and *p* values were less than 25% and 0.01, respectively, in accordance with GSEA recommendations.

### 2.6 RT PCR

RT-PCR was performed as previously described (Boucher et al., 2000). The sequence of primers used is listed in Table 1. New pools of DRG RNA from SNT-, gp120 + ddC- and VZV-treated animals were used for these experiments. DRG RNA was extracted by using guanidine isothiocyanate. Total RNA (2 μg) from L4 and/or L5 DRGs of sham or treated animals (*n* = 3 per group) was treated with DNase I, and cDNA was synthesized with Superscript reverse transcriptase (Invitrogen, Paisley, UK) using random hexamers in a total volume of 20 μl. In order to identify the optimal conditions for a linear range of amplification in the quantitative RT-PCR analysis, the amount of each amplified product was first checked at 24, 27, 30, 33, 36, and 39 cycles. Portions of the reverse-transcribed solution (0.5 μl) of total RNA from treated or sham ganglia were used for PCR. Each PCR (94 °C, 30 s; 58 °C, 30 s; 72 °C, 1 min; 33 cycles, 30 μl) was started with the primer pair specific for genes of interest (see Table 1). The primer pairs for cyclophilin A (PpiA) were added to the PCR reaction 5 cycles after the start. PCR products (10 μl) were visualized on 1.5% agarose gels, using ethidium bromide. The intensity of each band was analysed using Image J software (http://rsb.info.nih.gov/ij/) and normalized to cyclophilin A. To test for differential expression, a Student’s *t* test with a significance level of *p* < 0.05 was used.

**Table 1:** Primer sequences used for RT-PCR

Gene name	5′ primer	3′ primer	Size (bp)
TrkB_FL	TCTATGAAGACTGGACCACGC	TTCTCCAAGCTCCCTCTTCAG	449
TrkB_T1	TCTATGAAGACTGGACCACGC	CCAGTCACAGCTCACAACAAG	422
TrkB_T2	TCTATGAAGACTGGACCACGC	AGTGGGCAAGGCTGAGTAATC	399
Npy	ACTGACCCTCGCTCTATCCC	AACGACAACAAGGGAAATGG	400
Pap/Reg2	TGGCCTTCCCAGTCATGTC	AGATCTTGACAAGCTGCCACAG	445
Vgf	TACCCAGAACGAGGATTGCG	CAACAGTACCGCGGCCAG	381
PpiA	ACCCCACCGTGTTCTTCGAC	CATTTGCCATGGACAAGATG	300

### 2.7 Western Blots

Protein extraction, SDS–PAGE, and Western blotting were performed as previously described (Okuse et al., 1997). DRG were isolated and snap frozen, and protein was extracted by homogenization in RIPA buffer (25 mM Tris-HCl, pH 7.6, 150 mM NaCl, 1% NP-40, 1% sodium deoxycholate, 0.1% SDS, 1 mM PMSF, 1 μg/ml each aprotinin, leupeptin, pepstatin) followed by centrifugation at 10,000 g for 10 min. The protein concentration of the supernatant was determined by Bradford assay, and 50 μg protein was applied to each lane of 12% SDS–PAGE. Wet transfer was performed using Hybond ECL (Amersham), and the membrane was blocked for 60 min in 5% non-fat dried milk in TBS-T (50 mM Tris-HCl, pH 7.6, 140 mM NaCl, 0.1% Tween 20). The blot was probed with goat anti-Reg2/Pap (1:1000 dilution, R&D systems), rabbit anti-Atf3 (1:500 dilution, Santa Cruz Biotechnology) and anti-Actin (1:5000, Sigma) antibodies. Results were visualized with horseradish peroxidase-coupled anti-goat or anti-rabbit immunoglobulins (Dako) using ECL Western blotting detection system (Amersham).

## 3 Results

### 3.1 Mechanical hypersensitivity data

**Fig. 1:**
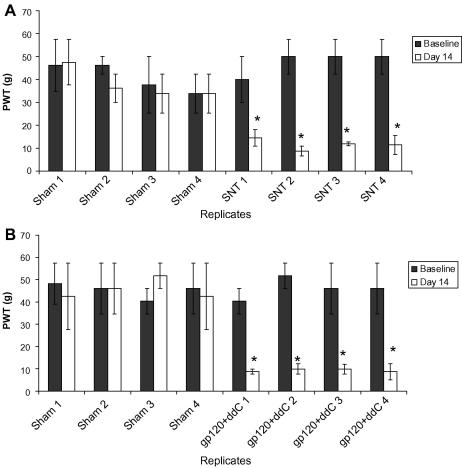
Paw withdrawal thresholds (PWT) to graded von Frey filaments at day 14 vs. baseline for (A) SNT and (B) gp120 + ddC. Sample size is three rats per replicate for A and two rats per replicate for B. ∗, Significant paired *t* test with *p* < 0.05 for comparing day 14 with the baseline value. Data are means ± SEM.

At day 14 post-intervention, all animals displayed hypersensitivity to a punctate mechanical stimulus, in the limb ipsilateral to treatment (Fig. 1). This has been previously established to be the time of maximal mechanical hypersensitivity in both models (Dowdall et al., 2005, Wallace et al., 2007b). On average there was an 80% reduction from baseline in gp120 + ddC-treated animals and a 75% reduction in SNT-treatment. Mechanical hypersensitivity was not observed in the contralateral paws of the treated animals (data not shown), nor in the ipsilateral paws of sham animals.

### 3.2 Model-specific differential expression of genes

The microarray experiment was conducted at one time point post-injury (day 14) and consisted of two conditions per model (treated animal versus sham) with four replicates per condition. To test for outlying chips, a number of quality control tests were carried out on the raw and RMA normalized data files (data not shown), which resulted in the exclusion of one of the replicates for the treated SNT animals.

We defined differentially expressed genes using criteria of 10% FDR and overall fold difference from sham >1.2 (see Section 2). Of the approximately, 30,000 transcripts queried on the Affymetrix GeneChip® Rat Genome 230 2.0 array, 656 probe sets were significantly disregulated in the gp120 + ddC DRGs. Of those, 243 were upregulated versus sham, whereas 413 were downregulated (supplementary tables 1 and 2). In SNT DRG, 2696 probe sets were upregulated versus sham and 2444 downregulated (supplementary tables 3 and 4). Moreover, considering fold changes alone, while only 40 probe sets showed over 2-fold expression differences between sham and treated animals in the gp120 + ddC model, 1593 probe sets showed over 2-fold difference in the SNT model. We interpret this as a reflection of the moderate amount of nerve damage induced in the sciatic nerve in the gp120 + ddC models, which is in contrast to the extensive trauma inflicted in the SNT model.

### 3.3 Comparison of SNT data with the literature

To verify the accuracy of the microarray results, we compared our SNT model results with two published microarray datasets describing differential gene expression (13 or 14 days after injury) in the DRG of rats subjected to spinal nerve ligation (SNL) (Wang et al., 2002, Valder et al., 2003). Our SNT model is a variation of the SNL model and thus our data are comparable to the SNL datasets. Both previous studies used rat RG-U34A arrays that contain 8799 probe sets and published lists of differentially expressed genes that show greater than 2-fold difference with *p* < 0.05. They compared ipsilateral to contralateral pools of L4, L5, and L6 DRG. Despite these analytical and methodological differences to our study, the data are expected to show strong similarities. Wang and colleagues (2002) identified 102 upregulated and 46 downregulated genes, while Valder and colleagues (2003) identified 98 upregulated and 113 downregulated genes. We merged these lists and identified probe sets for a total of 195 genes in the Rat Genome 230 2.0 array that we employed in this study. Hundred and fifty-nine of these probe sets (81.5%) are significant in our SNT dataset, with *p* < 0.04 (FDR < 0.1) and over 1.2-fold difference in the same direction as the previously reported studies. For a more detailed breakdown of these results, see supplementary Table 5. The correlation between the SNT results and published observations substantiates the accuracy of our data.

### 3.4 Gene Ontology results

We used MAPPFinder/GenMAPP and GSEA to perform a general search for pathways associated with gp120 + ddC treatment. With MAPPFinder/GenMAPP we found that 50% of the 243 significantly upregulated and 48% of the 413 downregulated genes in this model are linked to a GO term. Eleven GO functional annotation terms are over-represented in the upregulated gene list, neuropeptide signalling being the most significant (Table 2). Several other terms are related to organ development (e.g., tube morphogenesis, axonogenesis). Three GO terms are over-represented in the downregulated gene list (Table 2). They are all related to chloride channel activity (GABA-A receptor activity, chloride ion binding, chloride channel activity).

**Table 2:** Functional categories of genes highlighted by MAPPFinder for the gp120 + ddC model

GO name	GO type	No. of genes changing/no. of genes measured in the array	*Z* score	Permute *p*
*Upregulated*				
Neuropeptide signalling	P	4/29	4.852	0
Hyaluronic acid binding	F	2/8	4.924	0.003
Homophilic cell adhesion	P	4/35	4.281	0.003
Organ morphogenesis	P	9/154	3.801	0.004
Sex differentiation	P	3/25	3.83	0.005
Blood vessel morphogenesis	P	6/83	3.727	0.005
Regulation of epithelial cell proliferation	P	2/7	5.315	0.006
Electron carrier activity	F	5/71	3.326	0.006
Tube morphogenesis	P	4/36	4.198	0.008
Branching morphogenesis of a tube	P	3/22	4.169	0.009
Axonogenesis	P	5/68	3.447	0.009

*Downregulated*				
GABA-A receptor activity	F	3/8	5.767	0
Chloride ion binding	F	3/17	3.577	0.005
Chloride channel activity	F	3/18	3.435	0.006

**Fig. 2:**
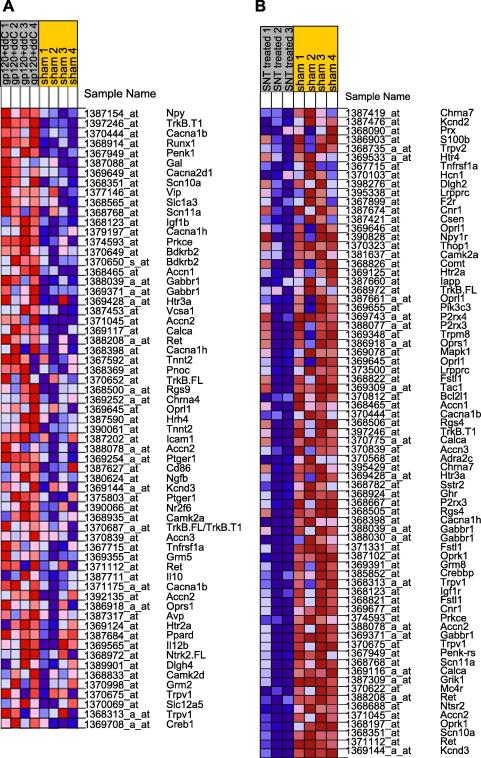
Heat map of the genes contributing most to the high enrichment score of the pain gene set for (A) gp120 + ddC and (B) SNT. Genes are ranked according to their expression under each experimental condition. For gp120 + ddC there is an association with upregulation, and thus the most differentially expressed genes are shown at the top of the heat map. For SNT there is an association with downregulation and thus the most differentially expressed genes are shown at the bottom of the heat map. Expression values are shown for each replicate within a condition and are represented as colours, where the range of colours (red, pink, light blue, dark blue) shows the range of expression values (high, moderate, low, and lowest).

The GSEA software was used to obtain a more detailed classification of the data into various affected pathways. GSEA considers all of the genes in an experiment, not only those above an arbitrary cut-off in terms of fold-change or significance. When GSEA was applied to the gp120 + ddC dataset, 20 gene sets were found to be up-regulated (FDR < 2.5 and *p* < 0.01) (Table 3). These include five gene sets that involve calcium dependent signalling (G alpha q pathway, Wnt/Ca2+/cyclic GMP pathway, calcium regulation in cardiac cells pathway, Pyk2 pathway and calcium homeostasis) and three cell adhesion gene sets (cell adhesion molecule activity, Brentani cell adhesion and GO cell adhesion). This is in agreement with the MAPPFinder results. The upregulation enriched gene sets also include a list of pain related genes. The genes that contribute to the high enrichment score for this gene set are illustrated in Fig. 2. Twelve gene sets are downregulated in the gp120 + ddC model (Table 4). They include a gene set of ribosomal proteins, three gene sets related to proteasomes and two for RNA/DNA transcriptome reactome genes.

**Table 3:** Gene sets associated with gene upregulation in gp120 + ddC using gene set enrichment analysis

Name	Top 5 genes	Source	Size	NOM *p*-val	FDR *q*-val
CELL_ADHESION_MOLECULE_ACTIVITY	Ncam1, Mpz, Gp9, L1cam, Cdh1	G0	103	0	0.064
PELP1PATHWAY	Mapk3, Pelp1, Crebbp, Ep300, Src	BioCarta	15	0	0.066
BRENTANI_CELL_ADHESION	Itga7, Ncam1, Ank1, Jup, Timp2	Brentani et al., Proc Natl Acad Sci USA, 2003, 100:13418–23	123	0	0.068
ST_GAQ_PATHWAY	Itpr3, Dag1, Gnaq, Itpr2, Nfkb2	STKE	43	0	0.079
ST_WNT_CA2_CYCLIC_GMP_PATHWAY	Itpr3, Dag1, Itpr2, Slc6A13	STKE	31	0	0.094
CELL_ADHESION	Ptprf, Ncam1, Gpr56, Atp2A2, Jup	G0	178	0	0.148
PENG_RAPAMYCIN_UP	Kcnh2, Tp53, Aes, Ucp2, Cd37	Peng et al., Mol. Cell Biol, 2002, 22:5575–84	153	0	0.157
PAIN_RELATED	NpY, TrkB.T1, Cacna1b, Runx1, Penk-rs	NetAffx	257	0	0.159
CALCIUM_REGULATION_IN_CARDIAC_ CELLS	Itpr3, Cacna1b, Cacba1c, Atp2a2, Atp1b2	GenMAPP	243	0	0.172
BRENTANI_SIGNALING	Stat3, Itga7, Wnt4, Arhgef12, Il6st	Brentani et al., Proc Natl Acad Sci USA, 2003, 100:13418–23	214	0	0.217
AMINOSUGARS_METABOLISM	Hk1, Gck, Syb5r3, Hexa, Hk2	KEGG	15	0.002	0.158
INFLAMMATORY_RESPONSE_PATHWAY	Lamc1, Lama5, Cd40lg, Cd86, Lamc2	Broad Institute	29	0.004	0.068
ST_T_CELL_SIGNAL_TRANSDUCTION	Dag1, Plcg1, Grb2, Cd3d, Nfkb2	STKE	44	0.005	0.172
PYK2PATHWAY	Mapk3, Plcg1, Gnaq, Jun, Grb2	BioCarta	63	0.005	0.206
INSULIN_SIGNALING	Npy, Ptprf, Srebf1, Jun, Slc27a4	Broad Institute	138	0.006	0.214
HIFPATHWAY	Jun, Ep300, Edn1, Hif1a, Creb1	BioCarta	19	0.006	0.074
STARCH_AND_SUCROSE_METABOLISM	Hk1, Gusb, Gaa, Gck, Gp1	KEGG	30	0.007	0.164
G2PATHWAY	Tp53, Gadd45a, Cdkn2d, Ep300, Cdc25b	BioCarta	28	0.007	0.164
CALCIUM_HOMEOSTASIS	Stat3, Casr, Cacna1c, Dnm1, Atp2a2	NetAffx	247	0.007	0.127
ARAPPATHWAY	Cltb, Arfgef2, Arfgap1, Pscd1, Pscd2	BioCarta	22	0.009	0.119

Top 5 genes – top 5 ranking genes belonging to a gene set; source – source for the gene set; size – number of genes in a gene set; NOM *p*-val – nominal *p* value; FDR *q*-val – false discovery rate.

**Table 4:** Gene sets associated with gene downregulation in gp120 + ddC using gene set enrichment analysis

Gene set name	Top 5 genes	Source	Size	NOM *p*-val	FDR *q*-val
PROTEASOME_DEGRADATION	Psmc6, Rps27a, Psma4, Psma1, H2afz	Broad Institute	39	0	2.40e^−04^
PROTEASOME	Psma2, Psma4, Psma1, Psma5, Psmb1	GenMAPP	18	0	3.60e^−04^
RIBOSOMAL_PROTEINS	Rps24, Rps27a, Rps25, Rpl21, Rpl7	GenMAPP	86	0	7.21e^−04^
STRIATED_MUSCLE_CONTRACTION	Tpm1, Ttn, Tnnt1, Tnni1, Myl2	GenMAPP	39	0	0.003
PROTEASOMEPATHWAY	Psma2, Psma4, Psma1, Psma5, Psmb1	BioCarta	22	0	0.02
ELECTRON_TRANSPORT_CHAIN	Cox6a2, Cox17, Nduf5, Cox7b, Ndyfs4	Broad Institute	80	0	0.021
ROME_INSULIN_2F_UP	Dpysl3, Ppp1r12a, Sh3glb1, Canx, Tpm1	Rome et al., J. Biol. Chem., 2003	204	0	0.161
DNA_REPLICATION_REACTOME	Orc3l, Prim2a, Rfc1, Rps27a, Orc4l	GenMAPP	34	0.002	0.075
OXIDATIVE_PHOSPHORYLATION	Cox6a2, Ndufa5, Cox7b, Atp6v1d, Atp5o	KEGG	48	0.003	0.066
RNA_TRANSCRIPTION_REACTOME	Ccnh, Gtf2f2, Cdk7, Mnat1, Polr2g	GenMAPP	28	0.009	0.115
PORPHYRIN_AND_CHLOROPHYLL_ METABOLISM	Eprs, Blvra, Cp, Urod, Hmox1	GenMAPP	18	0.020	0.153
UBIQUITIN_MEDIATED_PROTEOLYSIS	Ube2b, Ube2n, Ube2g1, Ube2d3	GenMAPP	20	0.022	0.1813

Top 5 genes – top 5 ranking genes belonging to a gene set; source – source for the gene set; size – number of genes in a gene set; NOM *p*-val – nominal *p* value; FDR *q*-val – false discovery rate.

In the SNT model data, 44% of the significantly upregulated and 43% of the downregulated genes are linked to a GO term. As shown in supplementary Table 6, the majority of biological process GO terms identified as significant in the list of upregulated genes are linked to neuroinflammation and immune system activation, highlighting the presence of dramatic neuroinflammation in SNT DRG. In contrast, multiple GO terms linked with ion transport (especially potassium ion transport), cell–cell signalling and cell communication are identified as significant in the list of downregulated genes. The obtained results agree with Wang et al., 2002, Valder et al., 2003 and provide another level of validation of our experimental results. The same pathways were also identified using GSEA analysis (supplementary tables 7 and 8). Interestingly, the gene set containing pain related genes is amongst those linked with down regulation in the SNT model. A detailed breakdown of the genes that contribute to the high enrichment score for this gene set are illustrated in Fig. 2.

### 3.5 Model cross comparisons

**Fig. 3:**
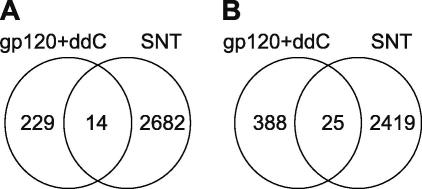
Venn diagrams to cross-compare the lists of significant probe sets (>1.2-fold difference and 10% FDR) between the SNT and gp120 + ddC models. (A) comparison of upregulated gene lists; (B) comparison of downregulated gene lists.

To identify genes that are relevant to mechanical hypersensitivity, we compared the lists of differentially expressed genes in both models, created using criteria of 10% FDR and overall >1.2-fold difference from sham, with Venn diagrams (see Fig. 3). We found 14 probe sets that are commonly upregulated in the DRGs of SNT and gp120 + ddC treated animals (Table 5). Only one of these is an EST of unknown function. The remaining probe sets correspond to annotated genes which can be subdivided into four groups on the basis of their function: signal peptides (Pap/Reg2, NpY, Scube1, Vgf), ion/protein transporters (Atp2B4, Dlg5, Slc4a8), transcription factors (Arnt2, Bptf, Irf7, Midn, Per2) and a cell differentiation molecule (Lama5) (Table 5).

**Table 5:** Probe sets upregulated for the SNT and gp120 + ddC models

Probe set ID	Gene title	Gene symbol	Fold increase SNT	Fold increase gp120 + ddc
1373315_at	Aryl hydrocarbon receptor nuclear translocator 2	Arnt2	1.4	1.3
1376911_at	ATPase, Ca^2+^ transporting, plasma membrane 4 (homo sapiens) (predicted)	Atp2B4_predicted	2.9	1.3
1376182_at	Bromodomain PHD finger transcription factor (Mus) (predicted)	Bptf_predicted	1.8	1.2
1377121_at	Discs, large homolog 5 (Drosophila) (predicted)	Dlg5_ predicted	1.5	1.2
1383564_at	Interferon regulatory factor 7	Irf7	3.4	1.2
1388932_at	Laminin, alpha 5	Lama5	1.8	1.3
1377103_at	Midnolin (predicted)	Midn_ predicted	1.6	1.3
1387154_at	Neuropeptide Y	Npy	8.6	1.9
1368238_at	Pancreatitis-associated protein	Pap/Reg2	22.3	1.2
1368303_at	Period homolog 2 (Drosophila)	Per2	2.6	1.3
1371910_at	Signal peptide, CUB domain, EGF-like 1 (Mus) (predicted)	Scube1_ predicted	1.6	1.4
1392238_at	Solute carrier family 4 (anion exchanger), member 8 (Mus) (predicted)	Slc4a8_predicted	2.2	1.3
1368359_a_at	VGF nerve growth factor inducible	Vgf	3.5	1.3
1393477_at	–	–	5.0	1.5

–, Unknown.

Twenty-five probe sets corresponding to 24 genes or ESTs were downregulated in the DRGs of SNT and gp120 + ddC treated animals (Table 6). Thirteen of these have unknown function while the remaining eleven are implicated in a variety of processes varying from metabolism (Gla, Fpgt, St8sia3), to ion/protein transport (Efcbp1, Kif5a, Rab27b, Sv2b), transcriptional activation (Esrrg), mitochondrial function (Senp5) axon guidance (Robo2) and signal transduction (Nkiras1).

**Table 6:** Probe sets downregulated for the SNT and gp120 + ddC models

Probe set ID	Gene title	Gene symbol	Fold decrease SNT	Fold decrease gp120 + ddc
1390892_at	DEP domain containing 1B (predicted)	Depdc1b_predicted	2.0	1.3
1386120_at	Mus musculus EF hand calcium binding protein 1 (Mus musculus) (predicted)	Efcbp1_predicted	1.7	1.4
1381445_at	Estrogen-related receptor gamma	Esrrg	2.5	1.4
1376753_at	Fucose-1-phosphate guanylyltransferase	Fpgt	1.7	1.3
1382063_at	Galactosidase, alpha	Gla	1.7	1.3
1382787_at	Kinesin family member 5A	Kif5a	2.0	1.7
1396215_at	Similar to RIKEN cDNA 2610022G08	LOC502782	1.7	1.3
1396117_at	Leucine zipper protein 2 (predicted)	Luzp2_predicted	2.0	1.7
1397555_at	Leucine zipper protein 2 (predicted)	Luzp2_ predicted	1.4	2.0
1393847_at	NFKB inhibitor interacting Ras-like protein 1 (predicted)	Nkiras1_predicted	1.7	1.3
1370122_at	RAB27B, member RAS oncogene family	Rab27b	2.0	1.7
1393909_at	Similar to KIAA1841 protein (predicted)	RGD1305110_predicted	1.4	1.7
1382812_at	Similar to Protein Njmu-R1 (predicted)	RGD1310429_predicted	2.0	1.4
1378245_at	Similar to 6430514L14Rik protein (predicted)	RGD1311958_predicted	1.7	1.3
1382632_at	Roundabout homolog 2 (Drosophila)	Robo2	2.5	1.7
1382477_at	SUMO/sentrin specific protease 5 (predicted)	Senp5_predicted	1.4	1.4
1387435_at	ST8 alpha-*N*-acetyl-neuraminide alpha-2,8-sialyltransferase 3	St8sia3	1.4	1.3
1369627_at	Synaptic vesicle glycoprotein 2b	Sv2b	1.7	2.5
1394412_at	Transmembrane protein 16C (predicted)	Tmem16c_ predicted	3.3	1.4
1392045_at	Transmembrane protein 22	Tmem22	2.0	1.3
1377917_at	Transcribed locus	–	1.7	1.4
1383162_at	–	–	1.3	1.4
1385972_at	–	–	1.4	1.4
1392663_at	Transcribed locus	–	1.7	1.3
1396676_at	Transcribed locus	–	2.7	1.4

–, Unknown.

### 3.6 RT-PCR and immunoblotting results

We sought to confirm the gene expression changes of a selected subset of genes using semi-quantitative RT-PCR analysis. Cyclophilin A was used as internal control.

**Fig. 4:**
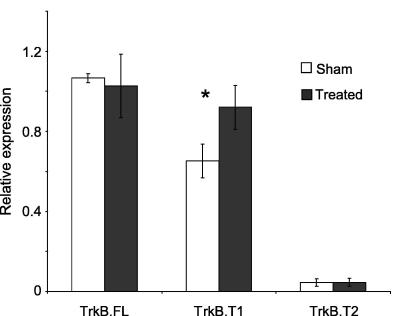
RT-PCR results for TrkB isoforms in gp120 + ddC treated animals. Normalized expression levels for each isoform are expressed as ratios to cyclophilin. Data are expressed as means ± SD. ∗, (*p* < 0.05) indicates statistical significance between treated and sham groups of animals (Student’s *t* test).

First, we measured the truncated isoform of TrkB (TrkB.T1), a receptor for BDNF (Huang and Reichardt, 2003) as this was the most upregulated gene in the gp120 + ddC DRG (supplementary information table 3). The Affymetrix GeneChip® Rat Genome 230 2.0 array also contains a probe set (1368972_at) that corresponds to the full length isoform of this gene (TrkB.FL). It showed no significant change in expression between treated and sham animals. We designed primers specific for TrkB.FL, for TrkB.T1 as well as for a second truncated isoform of TrkB (TrkB.T2) which is absent from the microarray. Only TrkB.T1 was found to be up-regulated in gp120 + ddC treated animals supporting the microarray findings (Fig. 4).

**Fig. 5:**
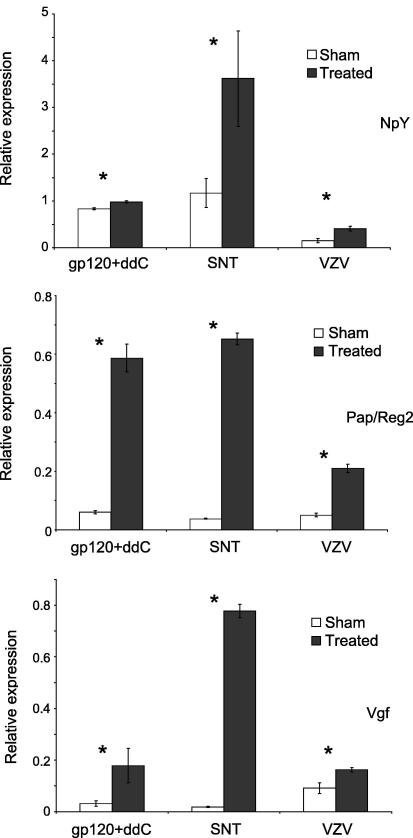
RT-PCR results for Npy, Pap/Reg2 and Vgf in SNT, gp120 + ddC and VZV infected animals. Normalized expression levels for each gene are expressed as ratios to cyclophilin. Data are expressed as means ± SD. ∗, (*p* < 0.05) indicates statistical significance between treated and sham groups of animals (Student’s *t* test).

We also measured levels of NpY, Pap/Reg2 and Vgf, the three neuropeptides that are commonly upregulated in SNT and gp120 + ddC models, by RT-PCR. All three genes were found to be markedly upregulated in agreement with the microarray results (Fig. 5). As a further test for the link between these genes and mechanical hypersensitivity, we also measured the differential expression of these three genes in L4 and L5 DRG of rats infected with Varicella Zoster virus. This is a disparate animal model of neuropathic pain, used to model the mechanisms underlying post-herpetic neuralgia (Sadzot-Delvaux et al., 1990, Fleetwood-Walker et al., 1999, Dalziel et al., 2004, Garry et al., 2005), which is also associated with similar mechanical hypersensitivity and thigmotaxis behaviour as is observed in SNT and GP120 + ddC (Hasnie et al., 2007, Wallace et al., 2007b). All three genes were also upregulated in the VZV treated animals (Fig. 5).

**Fig. 6:**
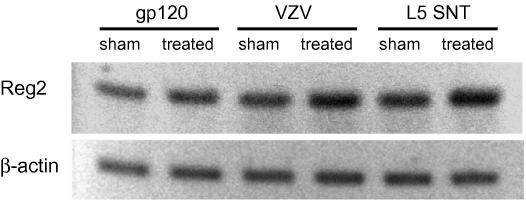
Immunoblots showing the induction of REG2/PAP protein expression in DRG from sham and SNT-, gp120 + ddC- and VZV-treated animals. The same membrane was re-probed with an anti β-ACTIN antibody.

We further confirmed the upregulation of PAP/Reg2 using immunoblot analysis to test if the observed mRNA level changes are maintained at the protein level. Pap/Reg2 was found to be upregulated by 1.4-fold for gp120 + ddC, 1.4-fold for SNT and by 1.1-fold for VZV (Fig. 6) further validating our microarray results.

## 4 Discussion

To elucidate the mechanisms underlying HIV DSP pain, we performed gene expression profiling in the L4 and L5 DRG of a recently developed rat model (gp120 + ddC) (Wallace et al., 2007b). We then focused our analysis towards the identification of genes that are directly relevant to presentation of mechanical hypersensitivity. To do so, we compared the gene profile of the gp120 + ddC model, with that of a model of direct sciatic nerve trauma (SNT) that although based on a distinct aetiology, shares the common outcome of mechanical hypersensitivity. As predicted, the models are associated with distinct sets of gene expression changes. However, we identified 39 genes/ESTs that are differentially regulated in the same direction in both models which may therefore underlie the common link of mechanical hypersensitivity.

Gp120 and nucleoside analogues are thought to affect cells by a variety of mechanisms including via cytokine and chemokine signalling pathways (Oh et al., 2001, Bhangoo et al., 2007). Accordingly, many biological process pathways overrepresented in the gp120 + ddC upregulated gene list involve signal transduction, such as neuropeptide signalling and the G alpha q pathway. Upregulated genes associated with these terms include TrkB.T1, Penk1, Celsr3, Nfkb2 and Itpr3. Interestingly, of these genes, the truncated isoform of TrkB (TrkB.T1), a receptor for BDNF (Huang and Reichardt, 2003), is the most upregulated gene in the gp120 + ddC model, which was further validated with RT-PCR. TrkB full length isoform (TrkB.FL)-BDNF signalling has been shown to protect neurons from gp120 induced cell death (Mocchetti and Bachis, 2004), but also thought to contribute to pain facilitation (Guo et al., 2006). TrkB.T1 is thought to modify the action of full length TrkB (TrkB.FL) signalling (Biffo et al., 1995, Eide et al., 1996, Haapasalo et al., 2001) and thus it is an interesting candidate for the development of hypersensitivity in the gp120 + ddC model.

DdC is associated with mitochondrial toxicity (Dalakas et al., 2001, Keswani et al., 2003a), while gp120 may cause axonal degeneration directly through activation of a mitochondrial caspase pathway and indirectly through neuronal apoptosis mediated by the activation of Schwann cells (Melli et al., 2006). GSEA analysis identified a gene set of ribosomal proteins as significantly associated with downregulation in the gp120 + ddC model. This set contains genes like Rps24, Rps27a, Rps25, and Rpl7.

The mitochondrial dysfunction induced by ddC alters calcium homeostasis in cultured DRG neurons (Werth et al., 1994) and in a model of ddC-associated painful peripheral neuropathy (Joseph et al., 2004). Gp120 is also associated with an increase in [Ca^2+^]_*i*_ in subsets of cultured DRG cells through the activation of chemokines (Oh et al., 2001). Therefore, alterations in calcium activity could underlie a synergistic effect of a combination of gp120 and ddC exposure. Such synergism of the treatments is suggested by the fact that behavioural indices of pain are enhanced in the gp120 + ddC model as compared to either treatment alone (Wallace et al., 2007b). We find that several genes involved in calcium ion transport (Casr, Plcg1 and Atp2a2) and calcium binding (e.g., Bcan, Egfl4, Dag1, Stat3, Capn1) are upregulated in the gp120 + ddC model suggesting that changes in calcium regulation may contribute to the development of neuropathy and merit further investigation.

Comparison of the gp120 + ddC and SNT model DRG gene expression profiles indicates that the pathological processes inherent to each model influence multiple gene expression pathways. For example, pathways involved in signalling or cell–cell adhesion are mainly downregulated in the SNT model and upregulated in the gp120 + ddC model. Interestingly, certain genes implicated in neuropathy formation and/or maintenance showed opposing patterns of differential expression between models. For example; protein kinase C, epsilon (PKCε) and its signalling pathways have been implicated in primary afferent nociceptor sensitization (Khasar et al., 1999) and it is considered an important contributor to the development of painful diabetic neuropathy (Ahlgren and Levine, 1994), taxol-induced painful peripheral neuropathy (Dina et al., 2001b), painful alcoholic neuropathy (Dina et al., 2000, Dina et al., 2006), and inflammatory hyperalgesia (Khasar et al., 1999, Aley et al., 2000, Dina et al., 2001a, Parada et al., 2005). PKCε was down regulated in SNT DRG and up-regulated in gp120 + ddC DRG. The calcitonin-related polypeptide alpha (Calca) gene, that codes for CGRP, which has been implicated in the development of thermal hypersensitivity in mice (Mogil et al., 2005) and mechanical hypersensitivity in a traumatic model of rat (Jang et al., 2004). Calca was found to be downregulated in the SNT model, but showed no significant changes in expression for the gp120 + ddC model, which is in line with previous investigations (Wallace et al., 2007b). This suggests that although these genes have been deemed important in traumatic neuropathy, they may not be associated with neuropathic pain of multiple pathophysiological origins or the manifestation of mechanical hypersensitivity.

There are some limitations of our experimental approach which require discussion. First, this study focuses on a single time point, when mechanical hypersensitivity is well established. Thus the genes identified are potentially important for this phase of mechanical hypersensitivity. To fully identify candidate genes for hypersensitivity generation and maintenance, this study would have to be repeated at a number of time points, which is prohibitively expensive given the current cost of this technology. Secondly, these microarray data reflects changes in gene expression in the entire DRG, which is a heterogenous tissue, composed of many cell types. Thus, this study cannot link changes to specific cell types in the DRG. *In situ* hybridization and/or immunohistochemical experiments are required to further clarify this point. Thirdly, in an effort to minimize animal usage and reduce biological variability, we employed a 2 cycle amplification protocol to generate the microarray targets. The 2 cycle amplification protocol produces reproducible results with high correlation between amplified and non-amplified RNAs (Saghizadeh et al., 2003, Klur et al., 2004, Li et al., 2005). However, RNA amplification may cause slight distortion (mainly decrease) of the expression ratios (Diboun et al., 2006) and failure to detect transcripts from the low intensity range (van Haaften et al., 2006). Therefore, our data might suffer from a slight increase in false negative results. This offers an explanation as to why differential expression for ATF3, Galanin, CCL2 and GFAP were detected by immunohistochemistry (Wallace et al., 2007b) but not by microarrays for the gp120 + ddC model (which exhibits subtle gene changes). Finally, we used a model of HIV-related neuropathy that includes two insults to the nervous system, gp120 and ddC, instead of the insults been applied individually. This model is clinically relevant since the decision to commence antiretroviral therapy is taken at a time when there is a high viral load and therefore circulating gp120 levels are high, with patients being simultaneously exposed to the double neurotoxic insults of gp120 and antiretroviral drugs (Gazzard et al., 2006, Wallace et al., 2007b). However, whereas our model reflects the clinical situation, we cannot be certain whether it is gp120, ddC or the combination which drives the gene changes seen.

Despite the potential limitations of this microarray study, we identified several potential targets for mechanical hypersensitivity maintenance. Thirty-nine genes/ESTs are differentially regulated in the same direction in both of our models. Of these genes, NPY has previously been suggested to contribute to nerve injury-induced mechanical hyperalgesia (White, 1997, Ossipov et al., 2002, Intondi et al., 2007). However, the majority of genes highlighted by our experiment represent potential novel targets in pain processing, two of which are discussed in more detail below.

Vgf is upregulated in the DRG of both gp120 + ddC and SNT models. VGF expression is induced by neurotrophins such as nerve growth factor (NGF) and brain-derived neurotrophic factor (BDNF) which are themselves important in the development, maintenance and normal functioning of neurons in the nervous system (Levi et al., 1985, Cho et al., 1989, Salton et al., 1991, Alder et al., 2003). The majority of experimental work on VGF peptide has focused upon its role as an energy homeostasis regulator (Hahm et al., 1999, Hahm et al., 2002, Watson et al., 2005, Bartolomucci et al., 2006) whilst the precise mechanism of VGF action and its role in pain requires further study. However, it is known that in the PNS, VGF is expressed mainly in small to medium diameter sensory neurons that project to the superficial laminae of the spinal cord (Ferri et al., 1992). VGF is a secreted polypeptide (Possenti et al., 1989), and thus may potentially mediate neuronal communication. Furthermore, another function of VGF is synaptic strengthening associated with learning ,memory and anti-depressant like behavioural effects (Alder et al., 2003, Thakker-Varia et al., 2007, Hunsberger et al., 2007). VGF has also been shown to be up-regulated in rat DRG at 2–28 days following axotomy (Xiao et al., 2002, Costigan et al., 2002). Although, axotomized rats do not develop mechanical hypersensitivity of the hind-paw due to deafferentation, VGF is also implicated as an important target in this model and clearly merits further investigation with regard to its role in pain.

Pap/Reg2 was also significantly up-regulated in both models. Reg-2 is a macrophage chemoattractant that is dynamically expressed in rat sensory neurons after peripheral nerve injury (Averill et al., 2002, Namikawa et al., 2005, Namikawa et al., 2006) and has been implicated in regeneration of motor and sensory neurons (Livesey et al., 1997, Namikawa et al., 2006). This regenerative role may account for the increase in SNT DRG as compared to the gp120 + ddC model. However, although largely expressed by injured neurons, Reg-2 has also been shown to be upregulated in uninjured axons also (Averill et al., 2002), which may implicate a non-regeneration associated role that may relate to mechanisms of hypersensitivity. The role of Reg-2 in pain is yet to be determined and our results suggest that this may be a good candidate to investigate for such properties.

This study has identified 39 potential mechanical hypersensitivity related genes/ESTs associated with the development of mechanical hypersensitivity and thigmotaxis following damage to the peripheral nervous system. Functional studies are now required to verify that the products of these genes are indeed involved in the generation and/or maintenance of neuropathic pain. Some of these genes may become targets for developing novel therapeutics to treat neuropathic pain at the level of the primary sensory neurone; where there is probably the most favourable prospect of developing analgesic drugs with a favourable therapeutic index in respect of CNS mediated adverse effects.

## References

[bib1] Ahlgren S.C., Levine J.D. (1994). Protein kinase C inhibitors decrease hyperalgesia and C-fiber hyperexcitability in the streptozotocin-diabetic rat. J Neurophysiol.

[bib2] Alder J., Thakker-Varia S., Bangasser D.A., Kuroiwa M., Plummer M.R., Shors T.J. (2003). Brain-derived neurotrophic factor-induced gene expression reveals novel actions of VGF in hippocampal synaptic plasticity. J Neurosci.

[bib3] Aley K.O., Messing R.O., Mochly-Rosen D., Levine J.D. (2000). Chronic hypersensitivity for inflammatory nociceptor sensitization mediated by the epsilon isozyme of protein kinase C. J Neurosci.

[bib4] Ashburner M., Ball C.A., Blake J.A., Botstein D., Butler H., Cherry J.M. (2000). Gene ontology: tool for the unification of biology. The Gene Ontology Consortium. Nat Genet.

[bib5] Averill S., Davis D.R., Shortland P.J., Priestley J.V., Hunt S.P. (2002). Dynamic pattern of reg-2 expression in rat sensory neurons after peripheral nerve injury. J Neurosci.

[bib6] Bakay M., Chen Y.W., Borup R., Zhao P., Nagaraju K., Hoffman E.P. (2002). Sources of variability and effect of experimental approach on expression profiling data interpretation. BMC Bioinformatics.

[bib7] Bartolomucci A., La C.G., Possenti R., Locatelli V., Rigamonti A.E., Torsello A. (2006). TLQP-21, a VGF-derived peptide, increases energy expenditure and prevents the early phase of diet-induced obesity. Proc Natl Acad Sci USA.

[bib8] Bhangoo S.K., Ren D., Miller R.J., Chan D.M., Ripsch M.S., Weiss C. (2007). CXCR4 chemokine receptor signaling mediates pain hypersensitivity in association with antiretroviral toxic neuropathy. Brain Behav Immun.

[bib9] Biffo S., Offenhauser N., Carter B.D., Barde Y.A. (1995). Selective binding and internalisation by truncated receptors restrict the availability of BDNF during development. Development.

[bib10] Boucher T.J., Okuse K., Bennett D.L., Munson J.B., Wood J.N., McMahon S.B. (2000). Potent analgesic effects of GDNF in neuropathic pain states. Science.

[bib11] Bridges D., Ahmad K., Rice A.S. (2001). The synthetic cannabinoid WIN55,212-2 attenuates hyperalgesia and allodynia in a rat model of neuropathic pain. Br J Pharmacol.

[bib12] Chaplan S.R., Bach F.W., Pogrel J.W., Chung J.M., Yaksh T.L. (1994). Quantitative assessment of tactile allodynia in the rat paw. J Neurosci Methods.

[bib13] Cherry C.L., McArthur J.C., Hoy J.F., Wesselingh S.L. (2003). Nucleoside analogues and neuropathy in the era of HAART. J Clin Virol.

[bib14] Cherry C.L., Skolasky R.L., Lal L., Creighton J., Hauer P., Raman S.P. (2006). Antiretroviral use and other risks for HIV-associated neuropathies in an international cohort. Neurology.

[bib15] Cho K.O., Skarnes W.C., Minsk B., Palmieri S., Jackson-Grusby L., Wagner J.A. (1989). Nerve growth factor regulates gene expression by several distinct mechanisms. Mol Cell Biol.

[bib16] Choi Y., Yoon Y.W., Na H.S., Kim S.H., Chung J.M. (1994). Behavioral signs of ongoing pain and cold allodynia in a rat model of neuropathic pain. Pain.

[bib17] Costigan M., Befort K., Karchewski L., Griffin R.S., D’Urso D., Allchorne A. (2002). Replicate high-density rat genome oligonucleotide microarrays reveal hundreds of regulated genes in the dorsal root ganglion after peripheral nerve injury. BMC Neurosci.

[bib18] Dahlquist K.D., Salomonis N., Vranizan K., Lawlor S.C., Conklin B.R. (2002). GenMAPP, a new tool for viewing and analyzing microarray data on biological pathways. Nat Genet.

[bib19] Dalakas M.C., Pezeshkpour G.H. (1988). Neuromuscular diseases associated with human immunodeficiency virus infection. Ann Neurol.

[bib20] Dalakas M.C., Semino-Mora C., Leon-Monzon M. (2001). Mitochondrial alterations with mitochondrial DNA depletion in the nerves of AIDS patients with peripheral neuropathy induced by 2′, 3′-dideoxycytidine (ddC). Lab Invest.

[bib21] Dalziel R.G., Bingham S., Sutton D., Grant D., Champion J.M., Dennis S.A. (2004). Allodynia in rats infected with varicella zoster virus – a small animal model for post-herpetic neuralgia. Brain Res Brain Res Rev.

[bib22] Diboun I., Wernisch L., Orengo C.A., Koltzenburg M. (2006). Microarray analysis after RNA amplification can detect pronounced differences in gene expression using limma. BMC Genomics.

[bib23] Dina O.A., Aley K.O., Isenberg W., Messing R.O., Levine J.D. (2001). Sex hormones regulate the contribution of PKCepsilon and PKA signalling in inflammatory pain in the rat. Eur J Neurosci.

[bib24] Dina O.A., Chen X., Reichling D., Levine J.D. (2001). Role of protein kinase Cepsilon and protein kinase A in a model of paclitaxel-induced painful peripheral neuropathy in the rat. Neuroscience.

[bib25] Dina O.A., Barletta J., Chen X., Mutero A., Martin A., Messing R.O. (2000). Key role for the epsilon isoform of protein kinase C in painful alcoholic neuropathy in the rat. J Neurosci.

[bib26] Dina O.A., Messing R.O., Levine J.D. (2006). Ethanol withdrawal induces hyperalgesia mediated by PKCepsilon. Eur J Neurosci.

[bib27] Doniger S.W., Salomonis N., Dahlquist K.D., Vranizan K., Lawlor S.C., Conklin B.R. (2003). MAPPFinder: using Gene Ontology and GenMAPP to create a global gene-expression profile from microarray data. Genome Biol.

[bib28] Dowdall T., Robinson I., Meert T.F. (2005). Comparison of five different rat models of peripheral nerve injury. Pharmacol Biochem Behav.

[bib29] Eide F.F., Vining E.R., Eide B.L., Zang K., Wang X.Y., Reichardt L.F. (1996). Naturally occurring truncated trkB receptors have dominant inhibitory effects on brain-derived neurotrophic factor signaling. J Neurosci.

[bib30] Ferri G.L., Levi A., Possenti R. (1992). A novel neuroendocrine gene product: selective VGF8a gene expression and immuno-localisation of the VGF protein in endocrine and neuronal populations. Brain Res Mol Brain Res.

[bib31] Fleetwood-Walker S.M., Quinn J.P., Wallace C., Blackburn-Munro G., Kelly B.G., Fiskerstrand C.E. (1999). Behavioural changes in the rat following infection with varicella-zoster virus. J Gen Virol.

[bib32] Garry E.M., Delaney A., Anderson H.A., Sirinathsinghji E.C., Clapp R.H., Martin W.J. (2005). Varicella zoster virus induces neuropathic changes in rat dorsal root ganglia and behavioral reflex sensitisation that is attenuated by gabapentin or sodium channel blocking drugs. Pain.

[bib33] Gautier L., Cope L., Bolstad B.M., Irizarry R.A. (2004). affy – analysis of Affymetrix GeneChip data at the probe level. Bioinformatics.

[bib34] Gazzard B., Bernard A.J., Boffito M., Churchill D., Edwards S., Fisher N. (2006). British HIV Association (BHIVA) guidelines for the treatment of HIV-infected adults with antiretroviral therapy (2006). HIV Med.

[bib35] Gentleman R.C., Carey V.J., Bates D.M., Bolstad B., Dettling M., Dudoit S. (2004). Bioconductor: open software development for computational biology and bioinformatics. Genome Biol.

[bib36] Guo W., Robbins M.T., Wei F., Zou S., Dubner R., Ren K. (2006). Supraspinal brain-derived neurotrophic factor signaling: a novel mechanism for descending pain facilitation. J Neurosci.

[bib37] Haapasalo A., Koponen E., Hoppe E., Wong G., Castren E. (2001). Truncated trkB. T1 is dominant negative inhibitor of trkB.TK+-mediated cell survival. Biochem Biophys Res Commun.

[bib38] Hahm S., Fekete C., Mizuno T.M., Windsor J., Yan H., Boozer C.N. (2002). VGF is required for obesity induced by diet, gold thioglucose treatment, and agouti and is differentially regulated in pro-opiomelanocortin- and neuropeptide Y-containing arcuate neurons in response to fasting. J Neurosci.

[bib39] Hahm S., Mizuno T.M., Wu T.J., Wisor J.P., Priest C.A., Kozak C.A. (1999). Targeted deletion of the Vgf gene indicates that the encoded secretory peptide precursor plays a novel role in the regulation of energy balance. Neuron.

[bib40] Hasnie F.S., Breuer J., Parker S., Wallace V., Blackbeard J., Lever I. (2007). Further characterization of a rat model of varicella zoster virus-associated pain: Relationship between mechanical hypersensitivity and anxiety-related behavior, and the influence of analgesic drugs. Neuroscience.

[bib41] Huang E.J., Reichardt L.F. (2003). Trk receptors: roles in neuronal signal transduction. Annu Rev Biochem.

[bib42] Hunsberger J.G., Newton S.S., Bennett A.H., Duman C.H., Russell D.S., Salton S.R. (2007). Antidepressant actions of the exercise-regulated gene VGF. Nat Med.

[bib43] Intondi A.B., Dahlgren M.N., Eilers M.A., Taylor B.K. (2007). Intrathecal neuropeptide Y reduces behavioral and molecular markers of inflammatory or neuropathic pain. Pain.

[bib44] Jang J.H., Nam T.S., Paik K.S., Leem J.W. (2004). Involvement of peripherally released substance P and calcitonin gene-related peptide in mediating mechanical hyperalgesia in a traumatic neuropathy model of the rat. Neurosci Lett.

[bib45] Joseph E.K., Chen X., Khasar S.G., Levine J.D. (2004). Novel mechanism of enhanced nociception in a model of AIDS therapy-induced painful peripheral neuropathy in the rat. Pain.

[bib46] Keswani S.C., Chander B., Hasan C., Griffin J.W., McArthur J.C., Hoke A. (2003). FK506 is neuroprotective in a model of antiretroviral toxic neuropathy. Ann Neurol.

[bib47] Keswani S.C., Polley M., Pardo C.A., Griffin J.W., McArthur J.C., Hoke A. (2003). Schwann cell chemokine receptors mediate HIV-1 gp120 toxicity to sensory neurons. Ann Neurol.

[bib48] Khasar S.G., Lin Y.H., Martin A., Dadgar J., McMahon T., Wang D. (1999). A novel nociceptor signaling pathway revealed in protein kinase C epsilon mutant mice. Neuron.

[bib49] Kim S.H., Chung J.M. (1992). An experimental model for peripheral neuropathy produced by segmental spinal nerve ligation in the rat. Pain.

[bib50] Klur S., Toy K., Williams M.P., Certa U. (2004). Evaluation of procedures for amplification of small-size samples for hybridization on microarrays. Genomics.

[bib51] Lacroix-Fralish M.L., Tawfik V.L., Tanga F.Y., Spratt K.F., Deleo J.A. (2006). Differential spinal cord gene expression in rodent models of radicular and neuropathic pain. Anesthesiology.

[bib52] Levi A., Eldridge J.D., Paterson B.M. (1985). Molecular cloning of a gene sequence regulated by nerve growth factor. Science.

[bib53] Levin M.E., Jin J.G., Ji R.R., Tong J., Pomonis J.D., Lavery D.J. (2007). Complement activation in the peripheral nervous system following the spinal nerve ligation model of neuropathic pain. Pain.

[bib54] Li L., Roden J., Shapiro B.E., Wold B.J., Bhatia S., Forman S.J. (2005). Reproducibility, fidelity, and discriminant validity of mRNA amplification for microarray analysis from primary hematopoietic cells. J Mol Diagn.

[bib55] Livesey F.J., O’Brien J.A., Li M., Smith A.G., Murphy L.J., Hunt S.P. (1997). A Schwann cell mitogen accompanying regeneration of motor neurons. Nature.

[bib56] Martin C., Pehrsson P., Osterberg A., Sonnerborg A., Hansson P. (1999). Pain in ambulatory HIV-infected patients with and without intravenous drug use. Eur J Pain.

[bib57] Martin C., Solders G., Sonnerborg A., Hansson P. (2003). Painful and non-painful neuropathy in HIV-infected patients: an analysis of somatosensory nerve function. Eur J Pain.

[bib58] Melli G., Keswani S.C., Fischer A., Chen W., Hoke A. (2006). Spatially distinct and functionally independent mechanisms of axonal degeneration in a model of HIV-associated sensory neuropathy. Brain.

[bib59] Mocchetti I., Bachis A. (2004). Brain-derived neurotrophic factor activation of TrkB protects neurons from HIV-1/gp120-induced cell death. Crit Rev Neurobiol.

[bib60] Mogil J.S., Miermeister F., Seifert F., Strasburg K., Zimmermann K., Reinold H. (2005). Variable sensitivity to noxious heat is mediated by differential expression of the CGRP gene. Proc Natl Acad Sci USA.

[bib61] Namikawa K., Fukushima M., Murakami K., Suzuki A., Takasawa S., Okamoto H. (2005). Expression of Reg/PAP family members during motor nerve regeneration in rat. Biochem Biophys Res Commun.

[bib62] Namikawa K., Okamoto T., Suzuki A., Konishi H., Kiyama H. (2006). Pancreatitis-associated protein-III is a novel macrophage chemoattractant implicated in nerve regeneration. J Neurosci.

[bib63] Oh S.B., Tran P.B., Gillard S.E., Hurley R.W., Hammond D.L., Miller R.J. (2001). Chemokines and glycoprotein120 produce pain hypersensitivity by directly exciting primary nociceptive neurons. J Neurosci.

[bib64] Okuse K., Chaplan S.R., McMahon S.B., Luo Z.D., Calcutt N.A., Scott B.P. (1997). Regulation of expression of the sensory neuron-specific sodium channel SNS in inflammatory and neuropathic pain. Mol Cell Neurosci.

[bib65] Ossipov M.H., Zhang E.T., Carvajal C., Gardell L., Quirion R., Dumont Y. (2002). Selective mediation of nerve injury-induced tactile hypersensitivity by neuropeptide Y. J Neurosci.

[bib66] Parada C.A., Reichling D.B., Levine J.D. (2005). Chronic hyperalgesic priming in the rat involves a novel interaction between cAMP and PKCepsilon second messenger pathways. Pain.

[bib67] Parkinson H., Kapushesky M., Shojatalab M., Abeygunawardena N., Coulson R., Farne A. (2007). ArrayExpress – a public database of microarray experiments and gene expression profiles. Nucleic Acids Res.

[bib68] Possenti R., Eldridge J.D., Paterson B.M., Grasso A., Levi A. (1989). A protein induced by NGF in PC12 cells is stored in secretory vesicles and released through the regulated pathway. EMBO J.

[bib69] Reiner A., Yekutieli D., Benjamini Y. (2003). Identifying differentially expressed genes using false discovery rate controlling procedures. Bioinformatics.

[bib70] Ringkamp M., Eschenfelder S., Grethel E.J., Habler H.J., Meyer R.A., Janig W. (1999). Lumbar sympathectomy failed to reverse mechanical allodynia- and hyperalgesia-like behavior in rats with L5 spinal nerve injury. Pain.

[bib71] Sadzot-Delvaux C., Merville-Louis M.P., Delree P., Marc P., Piette J., Moonen G. (1990). An in vivo model of varicella-zoster virus latent infection of dorsal root ganglia. J Neurosci Res.

[bib72] Saghizadeh M., Brown D.J., Tajbakhsh J., Chen Z., Kenney M.C., Farber D.B. (2003). Evaluation of techniques using amplified nucleic acid probes for gene expression profiling. Biomol Eng.

[bib73] Salton S.R., Fischberg D.J., Dong K.W. (1991). Structure of the gene encoding VGF, a nervous system-specific mRNA that is rapidly and selectively induced by nerve growth factor in PC12 cells. Mol Cell Biol.

[bib74] Simpson D.M., Kitch D., Evans S.R., McArthur J.C., Asmuth D.M., Cohen B. (2006). HIV neuropathy natural history cohort study: assessment measures and risk factors. Neurology.

[bib75] Smyth G.K. (2004). Linear models and empirical bayes methods for assessing differential expression in microarray experiments. Stat Appl Genet Mol Biol.

[bib76] Smyth G.K., Gentleman Vcsdriwh R. (2005). Bioinformatics and computational biology solutions using R and bioconductor.

[bib77] Smyth K., Affandi J.S., McArthur J.C., Bowtell-Harris C., Mijch A.M., Watson K. (2007). Prevalence of and risk factors for HIV-associated neuropathy in Melbourne, Australia 1993–2006. HIV Med.

[bib78] Subramanian A., Kuehn H., Gould J., Tamayo P., Mesirov J.P. (2007). GSEA-P: a desktop application for Gene Set Enrichment Analysis. Bioinformatics.

[bib79] Subramanian A., Tamayo P., Mootha V.K., Mukherjee S., Ebert B.L., Gillette M.A. (2005). Gene set enrichment analysis: a knowledge-based approach for interpreting genome-wide expression profiles. Proc Natl Acad Sci USA.

[bib80] Thakker-Varia S., Krol J.J., Nettleton J., Bilimoria P.M., Bangasser D.A., Shors T.J. (2007). The neuropeptide VGF produces antidepressant-like behavioral effects and enhances proliferation in the hippocampus. J Neurosci.

[bib81] Valder C.R., Liu J.J., Song Y.H., Luo Z.D. (2003). Coupling gene chip analyses and rat genetic variances in identifying potential target genes that may contribute to neuropathic allodynia development. J Neurochem.

[bib82] van Haaften R.I., Schroen B., Janssen B.J., van E.A., Debets J.J., Smeets H.J. (2006). Biologically relevant effects of mRNA amplification on gene expression profiles. BMC Bioinformatics.

[bib83] Verma S., Estanislao L., Simpson D. (2005). HIV-associated neuropathic pain: epidemiology, pathophysiology and management. CNS Drugs.

[bib84] Wallace V.C., Blackbeard J., Pheby T., Segerdahl A.R., Davies M., Hasnie F. (2007). Pharmacological, behavioural and mechanistic analysis of HIV-1 gp120 induced painful neuropathy. Pain.

[bib85] Wallace V.C., Blackbeard J., Segerdahl A.R., Hasnie F., Pheby T., McMahon S.B. (2007). Characterization of rodent models of HIV-gp120 and anti-retroviral-associated neuropathic pain. Brain.

[bib86] Wang H., Sun H., Della P.K., Benz R.J., Xu J., Gerhold D.L. (2002). Chronic neuropathic pain is accompanied by global changes in gene expression and shares pathobiology with neurodegenerative diseases. Neuroscience.

[bib87] Watson E., Hahm S., Mizuno T.M., Windsor J., Montgomery C., Scherer P.E. (2005). VGF ablation blocks the development of hyperinsulinemia and hyperglycemia in several mouse models of obesity. Endocrinology.

[bib88] Werth J.L., Zhou B., Nutter L.M., Thayer S.A. (1994). 2′,3′-Dideoxycytidine alters calcium buffering in cultured dorsal root ganglion neurons. Mol Pharmacol.

[bib89] White D.M. (1997). Intrathecal neuropeptide Y exacerbates nerve injury-induced mechanical hyperalgesia. Brain Res.

[bib90] Wilson C.L., Miller C.J. (2005). Simpleaffy: a BioConductor package for Affymetrix Quality Control and data analysis. Bioinformatics.

[bib91] Xiao H.S., Huang Q.H., Zhang F.X., Bao L., Lu Y.J., Guo C. (2002). Identification of gene expression profile of dorsal root ganglion in the rat peripheral axotomy model of neuropathic pain. Proc Natl Acad Sci USA.

[bib92] Zimmermann M. (1983). Ethical guidelines for investigations of experimental pain in conscious animals. Pain.

