# Supplementary material for: Comparison of dorsal root ganglion gene expression in rat models of traumatic and HIV-associated neuropathic pain
Source: Eur J Pain. 2009 Apr;13(4):387–98. doi: 10.1016/j.ejpain.2008.05.011 (PMC2706986; doi:10.1016/j.ejpain.2008.05.011)
Supplement: Supplementary data [file mmc1.pdf]

supplementary table 1

Supplementary table 1: Probe sets identified as up-regulated 10% FDR (p<0.004) and >1.2 fold in the L4+L5 DRGs of gp120+ddC animals

Probe set ID	Gene Title	Gene Symbol	Fold increase	p value	FDR value
1397246_at	Neurotrophic tyrosine kinase, receptor, type 2	TrkB.T1	1.6	1.03E-05	0.035179964
1385556_at	similar to KIAA0467 protein (predicted)	RGD1308616_predicted	1.4	2.59E-05	0.051799469
1371491_at	Notch gene homolog 1 (Drosophila)	Notch1	1.4	3.59E-05	0.051799469
1378672_at	Transcribed locus	---	1.4	4.80E-05	0.051799469
1373189_at	megakaryoblastic leukemia (translocation) 1 (predicted)	Mkl1_predicted	1.4	6.65E-05	0.051799469
1378876_at	Transcribed locus	---	1.7	6.67E-05	0.051799469
1372515_at	Transcribed locus	---	1.4	7.99E-05	0.054591631
1393477_at	---	---	1.5	9.34E-05	0.056951495
1377458_at	sortilin-related receptor, LDLR class A repeats-containing	Sorl1	1.3	0.000109667	0.060429896
1387934_at	brevican	Bcan	1.5	0.000138977	0.060484027
1379918_at	---	---	1.4	0.000145916	0.060484027
1375946_at	Transcribed locus	---	1.3	0.000151334	0.060484027
1399063_at	ZUBR1	Rbaf600	1.4	0.000165181	0.060484027
1386941_at	plectin 1	Plec1	1.3	0.000172174	0.060484027
1367707_at	fatty acid synthase	Fasn	1.4	0.000184671	0.060484027
1398791_at	thioredoxin reductase 1	Txnrd1	1.4	0.000189395	0.060484027
1371023_at	EGF-like-domain, multiple 4	Egfl4	1.4	0.000200363	0.060484027
1388808_at	polymerase (RNA) II (DNA directed) polypeptide A	Polr2a	1.4	0.000206835	0.060484027
1377061_at	RhoGAP involved in beta-catenin-N-cadherin and NMDA receptor signaling (predicted)	RICS_predicted	1.4	0.000221395	0.060484027
1387154_at	neuropeptide Y	Npy	1.9	0.000240474	0.060484027
1368036_at	protein tyrosine phosphatase, receptor type, F	Ptprf	1.3	0.000244316	0.060484027
1381063_at	adenylate cyclase 9 (predicted)	Adcy9_predicted	1.3	0.000271716	0.061369734
1387367_at	golgi apparatus protein 1	Glg1	1.3	0.000273333	0.061369734
1374910_at	cadherin EGF LAG seven-pass G-type receptor 2	Celsr2	1.5	0.000278571	0.061369734
1387924_at	neuronal guanine nucleotide exchange factor	Ngef	1.4	0.000287989	0.061369734
1374191_at	rhomboid family 1 (Drosophila)	Rhbdf1	1.4	0.000306245	0.061438374
1368005_at	inositol 1,4,5-triphosphate receptor 3	Itpr3	1.4	0.000316108	0.061438374
1387109_at	P450 (cytochrome) oxidoreductase	Por	1.3	0.00033466	0.062137848
1368347_at	procollagen, type V, alpha 3	Col5a3	1.4	0.000351122	0.062681954
1385525_at	similar to C-type lectin-like receptor 2 (predicted)	RGD1563517_predicted	1.8	0.000363911	0.062681954
1379324_at	Transcribed locus	---	1.4	0.000372034	0.062681954
1386929_at	hexokinase 1	Hk1	1.5	0.000372364	0.062681954
1390710_x_at	sortilin-related receptor, LDLR class A repeats-containing	Sorl1	1.3	0.00039997	0.063382433
1383620_at	---	---	1.3	0.000409741	0.063629048
1373751_at	Transcribed locus	---	1.3	0.000414477	0.063711755
1398445_at	Transcribed locus	---	1.4	0.0004445	0.064896995
1391032_at	seizure related 6 homolog (mouse)	Sez6	1.3	0.000455109	0.064970017
1376768_at	Transcribed locus	---	1.3	0.000470121	0.065894944
1388464_at	similar to cullin 7	LOC680835 /// LOC682940	1.4	0.000499194	0.067677153
1367963_at	G protein beta subunit-like	Gbl	1.3	0.000507123	0.067677153
1371429_at	dystroglycan 1	Dag1	1.4	0.000529007	0.068488178
1392534_at	transmembrane, prostate androgen induced RNA (predicted)	Tmepai_predicted	1.4	0.000529237	0.068488178
1390815_at	cyclin M1 (predicted)	Cnnm1_predicted	1.3	0.000529238	0.068488178
1382203_at	longevity assurance homolog 1 (S. cerevisiae) (predicted) /// growth differentiation factor 1 (predicted)	Gdf1_predicted /// Lass1_predicted	1.3	0.00055587	0.069012738
1371910_at	Transcribed locus	---	1.4	0.000558215	0.069012738
1386957_at	nuclear pore membrane glycoprotein 121	Pom121	1.3	0.000565297	0.069012738
1391856_at	sema domain, immunoglobulin domain (Ig), short basic domain, secreted, (semaphorin) 3G	Sema3g	1.5	0.000573304	0.069012738
1389809_at	Transcribed locus	---	1.3	0.000620774	0.069012738
1392593_a_at	similar to KIAA1205 protein (predicted)	RGD1309896_predicted	1.3	0.000658534	0.069012738

1372441_at	chromodomain helicase DNA binding protein 4	Chd4	1.3	0.00068437	0.069198114
1372196_at	nuclear receptor co-repressor 2 (predicted)	Ncor2_predicted	1.3	0.000684608	0.069198114
1392786_at	dysferlin (predicted)	Dysf_predicted	1.3	0.000692004	0.069376603
1389522_at	pleckstrin homology domain containing, family A member 6 (predicted)	Plekha6_predicted	1.4	0.000701967	0.069439606
1382123_at	---	---	1.4	0.000729405	0.069459846
1367801_at	endothelin converting enzyme 1	Ece1	1.3	0.000740059	0.069459846
1394982_at	Similar to 1200003M09Rik protein (predicted)	RGD1305553_predicted	1.3	0.000745101	0.069550925
1375793_at	myeloid/lymphoid or mixed-lineage leukemia	Mll	1.3	0.00076797	0.069652105
1378148_at	Transcribed locus	---	1.4	0.000774929	0.069652105
1374030_at	similar to KIAA0999 protein	LOC684112	1.3	0.000847756	0.0739693
1392187_at	Transcribed locus	---	1.3	0.000854135	0.0739693
1397685_at	Transcribed locus	---	1.3	0.000855003	0.0739693
1368914_at	runt related transcription factor 1	Runx1	1.3	0.000868673	0.074076268
1373551_at	similar to KIAA0614 protein (predicted)	RGD1309762_predicted	1.3	0.000878007	0.074076268
1383215_at	similar to TPR repeat-containing protein KIAA1043	LOC304558	1.3	0.000880686	0.074076268
1368710_at	MAP/microtubule affinity-regulating kinase 2	Mark2	1.3	0.000881363	0.074076268
1370199_at	nucleobindin 1	Nucb1	1.4	0.000901324	0.074182573
1367882_at	microtubule-associated protein 1 A	Mtap1a	1.3	0.000921305	0.075075244
1379389_at	Neural cell adhesion molecule 1	Ncam1	1.3	0.000924187	0.075075244
1377926_at	Centaurin, gamma 2 (predicted)	Centg2_predicted	1.3	0.000951153	0.075265191
1389787_at	PTK7 protein tyrosine kinase 7 (predicted)	Ptk7_predicted	1.4	0.000968033	0.075265191
1368137_at	microtubule-associated protein tau	Mapt	1.4	0.000969344	0.075265191
1398299_at	Rho guanine nucleotide exchange factor (GEF) 11	Arhgef11	1.3	0.000986027	0.075530525
1379847_at	dysferlin (predicted)	Dysf_predicted	1.4	0.001015693	0.076331462
1372055_at	similar to P-Rex1 (predicted)	RGD1306534_predicted	1.3	0.001027832	0.076489501
1372728_at	Sortilin 1	Sort1	1.2	0.001031771	0.076489501
1367636_at	insulin-like growth factor 2 receptor	Igf2r	1.2	0.001052247	0.076489501
1376303_a_at	membralin	RGD1311136	1.3	0.001069337	0.076489501
1390214_a_at	CDKN1A interacting zinc finger protein 1 (predicted)	Ciz1_predicted	1.3	0.001078116	0.076489501
1373541_at	Rho guanine nucleotide exchange factor (GEF) 17 (predicted)	Arhgef17_predicted	1.3	0.001095607	0.076489501
1383092_a_at	similar to mKIAA0023 protein (predicted)	RGD1304977_predicted	1.3	0.001097057	0.076489501
1383418_at	a disintegrin and metallopeptidase domain 11 (predicted)	Adam11_predicted	1.3	0.001128081	0.076887011
1392746_x_at	La ribonucleoprotein domain family, member 1 (predicted)	Larp1_predicted	1.4	0.001147467	0.076887011
1385967_at	forkhead box K2 (predicted) /// similar to forkhead box K2 isoform 1	Foxk2_predicted /// LOC688330	1.3	0.001155932	0.076887011
1377103_at	midnolin (predicted)	Midn_predicted	1.3	0.001165415	0.076887011
1387175_a_at	HLA-B-associated transcript 3	Bat3	1.3	0.001180668	0.076887011
1370648_a_at	SH3 domain binding protein CR16	Cr16	1.3	0.001183777	0.076887011
1369562_at	hippocalcin-like 1	Hpcal1	1.8	0.001202064	0.077473761
1387913_at	cytochrome P450, family 2, subfamily d, polypeptide 22	Cyp2d22	1.3	0.001206417	0.077473761
1368438_at	phosphodiesterase 10A	Pde10a	1.5	0.001235867	0.077760255
1369777_a_at	SH3/ankyrin domain gene 2	Shank2	1.2	0.001255962	0.077760255
1369158_at	calcium-sensing receptor	Casr	1.4	0.001271148	0.077760255
1390179_at	ankyrin repeat domain 52 (predicted)	Ankrd52_predicted	1.3	0.001275086	0.077760255
1378610_at	polyhomeotic like 3 (Drosophila) (predicted)	Phc3_predicted	1.3	0.001305742	0.077760255
1389400_at	SPEN homolog, transcriptional regulator (Drosophila) (predicted) /// similar to Msx2-interacting protein (SPEN homolog) (SMART/HDAC1-associated repressor protein)	LOC690911 /// Spen_predicted	1.3	0.001309416	0.077760255
1368267_at	protein-O-mannosyltransferase 1	Pomt1	1.3	0.001317724	0.077760255
1390563_at	ATP-binding cassette, sub-family A (ABC1), member 3	Abca3	1.3	0.001336944	0.077760255
1388240_a_at	integrin alpha 7	Itga7	1.4	0.001364084	0.077760255
1384959_at	Transcribed locus	---	1.2	0.001364612	0.077760255
1382995_at	Neuropilin 2	Nrp2	1.2	0.001372309	0.077760255

1390083_at	Retinitis pigmentosa 9 homolog (human) (predicted)	Rp9h_predicted	1.3	0.001377725	0.077760255
1368472_at	cadherin EGF LAG seven-pass G-type receptor 3	Celsr3	1.3	0.001380476	0.077760255
1375451_at	pecanex-like 3 (Drosophila)	Pcnxl3	1.3	0.001394837	0.077760255
1368303_at	period homolog 2 (Drosophila)	Per2	1.3	0.001397518	0.077760255
1386956_at	scavenger receptor class B, member 1	Scarb1	1.3	0.001413359	0.078056888
1394605_at	similar to CDNA sequence BC017647 (predicted)	RGD1566149_predicted	1.2	0.001426363	0.078093392
1376928_at	Similar to A disintegrin-like and metalloprotease (reprolysin type) with thrombospondin type 1 motif, 2 (predicted)	RGD1565950_predicted	1.3	0.001431577	0.078128436
1375983_at	MAM domain containing 2	Mamdc2	1.3	0.001469184	0.079661954
1388452_at	Transcribed locus, strongly similar to XP_579967.1 hypothetical protein XP_579967 [Rattus norvegicus]	---	1.3	0.001473667	0.079661954
1388193_at	huntingtin interacting protein 1	Hip1	1.3	0.001490663	0.079890524
1374246_at	Transcribed locus	---	1.2	0.001497287	0.079890524
1372943_at	similar to Ubiquitin-associated protein 2-like	LOC497952	1.3	0.001497991	0.079890524
1371018_at	cadherin EGF LAG seven-pass G-type receptor 2	Celsr2	1.3	0.00150128	0.079890524
1375212_at	ankyrin repeat domain 52 (predicted)	Ankrd52_predicted	1.3	0.001523059	0.080458803
1381685_a_at	similar to CG12753-PA (predicted)	RGD1305773_predicted	1.2	0.001544419	0.080458803
1376911_at	Transcribed locus	---	1.3	0.001550022	0.080458803
1368292_at	dynamin 1	Dnm1	1.3	0.001557029	0.080458803
1387729_at	gamma-glutamyltransferase-like activity 1	Ggtla1	1.3	0.001576014	0.080458803
1389376_at	Transcribed locus	---	1.3	0.001576906	0.080458803
1368352_at	syntaxin 1B2	Stx1b2	1.3	0.001586271	0.080458803
1370355_at	stearoyl-Coenzyme A desaturase 1	Scd1	1.4	0.001588115	0.080458803
1370231_at	general transcription factor III C 1	Gtf3c1	1.3	0.00160368	0.080458803
1389844_at	FK506 binding protein 4	Fkbp4	1.4	0.001644734	0.081884988
1379591_at	---	---	1.2	0.001678694	0.082142371
1369249_at	progressive ankylosis homolog (mouse)	Ank	1.3	0.001687857	0.082142371
1390113_a_at	La ribonucleoprotein domain family, member 1 (predicted)	Larp1_predicted	1.3	0.001737155	0.08335993
1368359_a_at	VGF nerve growth factor inducible	Vgf	1.3	0.001737275	0.08335993
1383318_at	Transcribed locus	---	1.3	0.001758944	0.083561954
1383331_at	Transcribed locus	---	1.9	0.001765747	0.083561954
1368047_at	solute carrier family 13 (sodium-dependent dicarboxylate transporter), member 3	Slc13a3	1.3	0.001765945	0.083561954
1374089_at	Transcribed locus	---	1.4	0.001787442	0.083605912
1388426_at	sterol regulatory element binding factor 1	Srebf1	1.2	0.001804221	0.083605912
1368183_at	phospholipase C, gamma 1	Plcg1	1.2	0.001820189	0.083605912
1379536_at	pleckstrin homology domain containing, family A member 6 (predicted)	Plekha6_predicted	1.2	0.001853098	0.083605912
1373900_at	keratin complex 2, basic, gene 7	Krt2-7	1.4	0.001853478	0.083605912
1370264_at	CPG2 protein	CPG2	1.3	0.001860414	0.083605912
1370362_at	protein tyrosine phosphatase, receptor type, N	Ptprn	1.3	0.001872453	0.083605912
1398458_at	similar to protein kinase, lysine deficient 1; kinase deficient protein (predicted)	RGD1307284_predicted	1.3	0.001874067	0.083605912
1371781_at	signal transducer and activator of transcription 3	Stat3	1.5	0.001879329	0.083605912
1367708_a_at	fatty acid synthase	Fasn	1.3	0.001883092	0.083605912
1385716_at	Hypothetical protein LOC679624	LOC679624	1.3	0.001891543	0.083605912
1389769_at	---	---	1.2	0.001902118	0.08362479
1388478_at	transducin (beta)-like 1 X-linked (predicted)	Tbl1x_predicted	1.3	0.001907869	0.08362479
1372048_at	podocalyxin-like 2 (predicted)	Podxl2_predicted	1.3	0.001945617	0.084352863
1384264_at	myosin, heavy polypeptide 14	Myh14	1.3	0.001958792	0.084516929
1398862_at	ATPase, Ca++ transporting, cardiac muscle, slow twitch 2	Atp2a2	1.3	0.001979164	0.084533769
1371569_at	Transcribed locus	---	1.3	0.001980877	0.084533769
1376205_at	Transcribed locus	---	1.2	0.001996086	0.0846852
1391863_at	Transcribed locus	---	1.5	0.002056214	0.085882478
1392238_at	Transcribed locus	---	1.3	0.002073471	0.085968527
1372063_at	Similar to novel protein of unknown function (DUF423) family member (predicted)	RGD1563438_predicted	1.3	0.00213855	0.087394051
1367949_at	proenkephalin 1	Penk1	1.3	0.002156044	0.087898676

1395642_at	nucleolar protein 9	Nol9	1.2	0.002166618	0.08796992
1378628_at	Transcribed locus	---	1.4	0.002168091	0.08796992
1373855_at	Transcribed locus	---	1.3	0.002173514	0.087980946
1375049_at	---	---	1.3	0.002196743	0.088340206
1381346_at	Transcribed locus	---	1.3	0.002216856	0.088892787
1373245_at	procollagen, type IV, alpha 1	Col4a1	1.4	0.002236789	0.089149533
1375433_at	Dispatched homolog 2 (Drosophila) (predicted)	Disp2_predicted	1.3	0.00227302	0.089464804
1386958_at	thioredoxin reductase 1	Txnrd1	1.2	0.002307385	0.089988014
1376584_at	Transcribed locus	---	1.3	0.002355904	0.090683187
1387874_at	D site albumin promoter binding protein	Dbp	1.4	0.002395687	0.091144684
1379743_at	Nucleotide binding protein 2	Nubp2	1.8	0.002398294	0.091144684
1392215_at	Eph receptor B1	Ephb1	1.2	0.002427504	0.091740327
1388932_at	laminin, alpha 5	Lama5	1.3	0.002442582	0.091899558
1387009_at	calpain 1	Capn1	1.3	0.002488514	0.09261177
1368641_at	wingless-related MMTV integration site 4	Wnt4	1.4	0.002498766	0.092791124
1374655_at	Transcribed locus	---	1.3	0.002513866	0.092947745
1368483_a_at	slit homolog 1 (Drosophila)	Slit1	1.3	0.002519918	0.092970268
1381460_at	---	---	1.3	0.002555734	0.093602564
1383337_at	Similar to genetic suppressor element 1 (predicted)	RGD1562686_predicted	1.3	0.002558974	0.093602564
1377618_at	Kruppel-like factor 7 (ubiquitous) (predicted)	Klf7_predicted	1.4	0.002606679	0.094904798
1390409_at	LOC361774 (predicted)	RGD1306116_predicted	1.2	0.002611243	0.094904798
1371558_at	nischarin	Nisch	1.3	0.002645093	0.095391055
1376095_at	Transcribed locus	---	1.2	0.002667436	0.095499738
1371874_at	ubiquitin specific protease 19	Usp19	1.3	0.002672338	0.095499738
1396920_at	G protein-coupled receptor 176	Gpr176	1.3	0.002695947	0.095518141
1372222_at	Similar to hypothetical protein F730001J03 (predicted)	RGD1311757_predicted	1.2	0.002706203	0.095518141
1375417_at	protein kinase C binding protein 1	Prkcbp1	1.2	0.00270633	0.095518141
1373190_at	CCR4-NOT transcription complex, subunit 4	Cnot4	1.2	0.002734781	0.095833195
1394179_s_at	Transcribed locus	---	1.3	0.002737724	0.095833195
1389637_at	Similar to WW domain binding protein 2	LOC688642	1.4	0.002740052	0.095833195
1383963_at	Transcribed locus	---	1.2	0.002751518	0.095833195
1384316_at	Transcribed locus	---	1.2	0.002760211	0.095833195
1371923_at	acyltransferase like 2 (predicted)	Aytl2_predicted	1.2	0.002775193	0.096048203
1373422_at	La ribonucleoprotein domain family, member 1 (predicted)	Larp1_predicted	1.2	0.002810554	0.096058869
1370599_a_at	protein tyrosine phosphatase, receptor type, D	Ptprd	1.3	0.002816419	0.096058869
1372966_at	hypothetical LOC298504 (predicted)	RGD1310174_predicted	1.3	0.002820812	0.096058869
1377693_at	FERM and PDZ domain containing 1 (predicted)	Frmpd1_predicted	1.2	0.002825639	0.096058869
1371797_at	carbamyl phosphatate synthetase 2	Cad	1.2	0.002832024	0.096058869
1374262_at	Transcribed locus	---	1.2	0.002842527	0.096058869
1373565_at	SWI/SNF related, matrix associated, actin dependent regulator of chromatin, subfamily a, member 4	Smarca4	1.2	0.002867983	0.096058869
1374641_at	Transcribed locus	---	1.2	0.002871559	0.096058869
1389679_at	similar to testhymin (predicted)	RGD1563612_predicted	1.4	0.002879179	0.096058869
1384302_at	solute carrier family 6 (neurotransmitter transporter), member 17	Slc6a17	1.7	0.002896553	0.09627783
1394705_at	Transcribed locus	---	1.3	0.002900753	0.09627783
1372937_at	Transcribed locus	---	1.2	0.002906757	0.09627783
1373315_at	Aryl hydrocarbon receptor nuclear translocator 2	Arnt2	1.3	0.002938292	0.09627783
1374496_at	similar to RIKEN cDNA 1200009O22; EST AI316813	RGD1310827	1.3	0.002945702	0.09627783
1394078_at	---	---	1.3	0.002962938	0.09627783
1388951_at	similar to Trithorax homolog 2 (Mixed lineage leukemia gene homolog 2 protein) (predicted)	RGD1308331_predicted	1.3	0.002978719	0.09627783
1383135_at	Neurotrophic tyrosine kinase, receptor, type 2	Ntrk2	1.3	0.002991103	0.09627783
1384771_at	---	---	1.2	0.002995933	0.09627783
1369722_a_at	xylosyltransferase II	Xylt2	1.2	0.003001512	0.09627783
1376233_at	Discs, large (Drosophila) homolog-associated protein 3	Dlgap3	1.2	0.003009739	0.09627783

1385442_at	Transcribed locus	---	1.3	0.003039757	0.096962183
1373793_at	immunoglobulin superfamily, member 8	Igsf8	1.2	0.003102831	0.097459196
1367511_at	nicastrin	Ncstn	1.3	0.003134056	0.097459196
1374419_at	adenylate cyclase 9 (predicted)	Adcy9_predicted	1.3	0.003152172	0.097459196
1372095_at	similar to KIAA1161 protein (predicted)	RGD1309821_predicted	1.3	0.003198632	0.097873471
1377121_at	discs, large homolog 5 (Drosophila) (predicted)	Dlg5_predicted	1.2	0.003216822	0.098124556
1389341_at	plexin D1 (predicted)	Plxnd1_predicted	1.2	0.003282282	0.098971977
1368238_at	pancreatitis-associated protein	Pap	1.2	0.003409729	0.10043969
1377821_at	Transcribed locus	---	1.3	0.003410873	0.10043969
1375994_at	Neurofibromatosis 1	Nf1	1.2	0.003464795	0.10043969
1375096_at	stratifin (predicted)	Sfn_predicted	1.2	0.003469567	0.10043969
1388609_at	Similar to RIKEN cDNA 1700027M01	RGD1311815	1.2	0.00347154	0.10043969
1394957_at	nischarin	Nisch	1.4	0.003520337	0.101066225
1391672_at	Transcribed locus	---	1.2	0.003534263	0.101277209
1372875_at	Transcribed locus	---	1.3	0.003565843	0.101391987
1376733_at	immunoglobulin superfamily, member 11	Igsf11	1.3	0.003573432	0.101391987
1379906_at	ATPase, Na+/K+ transporting, beta 2 polypeptide	Atp1b2	1.3	0.003617825	0.101907022
1378414_at	Transcribed locus, strongly similar to XP_230530.3 similar to hypothetical protein [Rattus norvegicus]	---	1.2	0.003619822	0.101907022
1374135_at	importin 4 (predicted)	Ipo4_predicted	1.3	0.003624553	0.101907022
1387061_at	junction plakoglobin	Jup	1.3	0.003633145	0.101907022
1398415_at	Transcribed locus	---	1.2	0.003677214	0.102267664
1369678_a_at	nuclear factor I/A	Nfia	1.3	0.003685127	0.102267664
1376182_at	Transcribed locus, weakly similar to NP_066564.1 general control of amino-acid synthesis 5-like 2 [Homo sapiens]	---	1.2	0.003692444	0.102267664
1371383_at	Dr1 associated protein 1 (negative cofactor 2 alpha) (predicted)	Drap1_predicted	1.4	0.003709557	0.102267664
1371250_at	chemokine (C-X-C motif) ligand 4	Cxcl4	1.8	0.003734276	0.102371868
1384138_at	Transcribed locus	---	1.3	0.00374589	0.102371868
1373187_at	Transcribed locus	---	1.3	0.003747407	0.102371868
1390026_at	Bcl2-associated athanogene 3	Bag3	1.2	0.003767569	0.102371868
1383564_at	interferon regulatory factor 7	Irf7	1.2	0.003773645	0.102371868
1387813_at	v-erb-b2 erythroblastic leukemia viral oncogene homolog 2, neuro/glioblastoma derived oncogene homolog (avian)	Erbb2	1.3	0.003794216	0.102371868
1389002_at	talin 1	Tln1	1.3	0.003812505	0.102371868
1371473_at	cleft lip and palate associated transmembrane protein 1 (predicted)	Clptm1_predicted	1.3	0.003831387	0.102371868
1374593_at	protein kinase C, epsilon	Prkce	1.2	0.003835499	0.102371868
1383229_at	ATP-binding cassette, sub-family A (ABC1), member 7	Abca7	1.2	0.003847424	0.10243669
1376025_at	HMT1 hnRNP methyltransferase-like 1 (S. cerevisiae)	Hrmt1l1	1.3	0.003873734	0.102461219
1391688_at	Transcribed locus	---	1.2	0.003876702	0.102461219
1375699_at	Transcribed locus	---	1.2	0.003880339	0.102461219
1367852_s_at	glycoprotein Ib, beta polypeptide /// septin 5	Gp1bb /// Sept5	1.3	0.003914541	0.102525381
1372351_at	bromodomain containing 4	Brd4	1.2	0.003993916	0.103684001

supplementary table 2

Supplementary table 2: Probe sets identified as down-regulated 10% FDR (p<0.004) and >1.2 fold in the L4+L5 DRGs of gp120+ddC animals

Probe set ID	Gene Title	Gene Symbol	Fold increase	p value	FDR value
1390706_at	spectrin beta 2	Spnb2	0.4	2.05E-06	0.021095388
1380447_a_at	mitochondrial ribosomal protein S18C (predicted)	Mrps18c_predicted	0.6	2.47E-06	0.021095388
1374974_at	Transcribed locus	---	0.5	5.50E-06	0.031340167
1373347_at	acyl-Coenzyme A binding domain containing 3	Acbd3	0.6	9.72E-06	0.035179964
1394600_at	Transcribed locus	---	0.5	2.01E-05	0.051799469
1379715_at	similar to CG9346-PA (predicted)	RGD1307882_predicted	0.5	2.45E-05	0.051799469
1377694_at	Similar to cisplatin resistance-associated overexpressed protein (predicted)	RGD1307981_predicted	0.6	2.85E-05	0.051799469
1382466_at	similar to RIKEN cDNA 6530403A03	RGD1309020	0.6	3.87E-05	0.051799469
1391170_at	similar to mKIAA1757 protein (predicted)	RGD1310433_predicted	0.4	4.91E-05	0.051799469
1377728_at	RGD1565641 (predicted)	RGD1565641_predicted	0.7	4.99E-05	0.051799469
1375442_at	M-phase phosphoprotein 10 (U3 small nucleolar ribonucleoprotein) (predicted)	Mphosph10_predicted	0.5	5.03E-05	0.051799469
1372543_at	similar to RIKEN cDNA 2610029G23 (predicted)	RGD1562502_predicted	0.7	5.72E-05	0.051799469
1389444_at	Similar to RIKEN cDNA 5033406L14	RGD1305755	0.6	5.94E-05	0.051799469
1382632_at	roundabout homolog 2 (Drosophila)	Robo2	0.6	6.36E-05	0.051799469
1383343_at	similar to reduced expression 2	LOC690559	0.7	6.37E-05	0.051799469
1376096_a_at	Transcribed locus	---	0.7	6.41E-05	0.051799469
1385250_at	chromodomain helicase DNA binding protein 6 (predicted)	Chd6_predicted	0.7	7.20E-05	0.053477666
1377262_at	similar to KIAA2010 protein (predicted)	RGD1309450_predicted	0.5	7.72E-05	0.054591631
1384339_s_at	casein kinase II, alpha 1 polypeptide	Csnk2a1	0.5	8.75E-05	0.056951495
1375894_at	leucine zipper transcription factor-like 1	Lztfl1	0.7	9.26E-05	0.056951495
1382362_at	Transcribed locus	---	0.7	0.000105921	0.06040267
1379469_at	transducin (beta)-like 1 X-linked (predicted)	Tbl1x_predicted	0.6	0.000106081	0.06040267
1395595_at	Mki67 (FHA domain) interacting nucleolar phosphoprotein	Mki67ip	0.6	0.000116185	0.060484027
1377501_at	similar to zinc finger protein 75 (predicted)	RGD1565419_predicted	0.6	0.000121873	0.060484027
1392207_at	LUC7-like (S. cerevisiae)	Luc7l	0.6	0.000125541	0.060484027
1369627_at	synaptic vesicle glycoprotein 2b	Sv2b	0.4	0.000130204	0.060484027
1384792_at	pre-mRNA processing factor 40 homolog A (yeast) (predicted)	Prpf40a_predicted	0.5	0.000135794	0.060484027
1393764_at	Opioid growth factor receptor-like 1	Ogfrl1	0.7	0.000150369	0.060484027
1392838_at	similar to CG13957-PA (predicted)	RGD1309995_predicted	0.5	0.00015941	0.060484027
1394837_at	---	---	0.4	0.000161803	0.060484027
1391075_at	regulator of G-protein signaling 17 (predicted)	Rgs17_predicted	0.7	0.000165817	0.060484027
1375644_at	Transthyretin	Ttr	0.5	0.000173943	0.060484027
1394243_at	spermine synthase /// similar to spermine synthase (predicted) /// similar to spermine synthase	LOC683008 /// LOC690027 /// RGD1559938_predicted /// Sms	0.6	0.000177339	0.060484027
1394891_at	Transcribed locus	---	0.7	0.000188321	0.060484027
1393909_at	similar to KIAA1841 protein (predicted)	RGD1305110_predicted	0.6	0.000190074	0.060484027
1376641_at	THO complex 1	Thoc1	0.7	0.000195323	0.060484027
1372793_at	single-stranded DNA binding protein 1	Ssbp1	0.7	0.000201979	0.060484027
1390893_at	transmembrane protein 69	Tmem69	0.7	0.000204216	0.060484027
1373538_at	ubiquitin specific peptdiase 1	Usp1	0.5	0.000208087	0.060484027
1389989_at	alpha thalassemia/mental retardation syndrome X-linked homolog (human)	Atrx	0.5	0.000211189	0.060484027
1368083_at	cyclin H	Ccnh	0.7	0.000213804	0.060484027
1384181_at	Transcribed locus	---	0.6	0.000215546	0.060484027
1383549_at	similar to RIKEN cDNA C030048B08	RGD1310794	0.7	0.000220745	0.060484027
1374232_at	phosphatidylinositol 3-kinase, catalytic, alpha polypeptide	Pik3ca	0.6	0.000228094	0.060484027
1398617_at	Transcribed locus, strongly similar to XP_001060577.1 similar to ribosomal protein S27a [Rattus norvegicus]	---	0.7	0.000231796	0.060484027

1385349_at	similar to centrin 4	LOC684095	0.7	0.00024025	0.060484027
1384154_at	WW domain binding protein 4	Wbp4	0.6	0.000241051	0.060484027
1399164_a_at	similar to RIKEN cDNA 1810030N24 (predicted)	RGD1305158_predicted	0.7	0.00024276	0.060484027
1376175_at	similar to NipSnap2 protein (Glioblastoma amplified sequence)	LOC498174	0.6	0.000251365	0.061340155
1390168_a_at	zinc finger, CSL-type containing 3 (predicted)	Zcsl3_predicted	0.7	0.00026214	0.061369734
1372928_at	Transcribed locus, strongly similar to XP_580054.1 hypothetical protein XP_580054 [Rattus norvegicus]	---	0.7	0.000262363	0.061369734
1398716_at	Non-coding RNA expressed in the brain, repeat sequence, clone 3 /// Non-coding RNA expressed in the brain, repeat sequence, clone 2 /// Non-coding RNA expressed in the brain, repeat sequence, clone 12 /// Non-coding RNA expressed in the brain, repeat sequence, clone 7	---	0.5	0.000276284	0.061369734
1380523_at	F-box protein 15 (predicted)	Fbxo15_predicted	0.6	0.000291515	0.061369734
1395199_at	eukaryotic translation initiation factor 3, subunit 1 alpha (predicted) /// similar to Eukaryotic translation initiation factor 3 subunit 1 (eIF-3 alpha)	Eif3s1_predicted /// LOC691947	0.7	0.000291872	0.061369734
1370122_at	RAB27B, member RAS oncogene family	Rab27b	0.6	0.000294263	0.061369734
1392166_at	Non-coding RNA expressed in the brain, repeat sequence, clone 12	---	0.5	0.000297011	0.061369734
1390549_at	adiponectin receptor 2	Adipor2	0.6	0.000297692	0.061369734
1391466_at	---	---	0.7	0.00029819	0.061369734
1381542_at	UBX domain containing 2	Ubxd2	0.5	0.000314647	0.061438374
1390454_at	4-nitrophenylphosphatase domain and non-neuronal SNAP25-like protein homolog 1 (C. elegans)	Nipsnap1	0.7	0.000316522	0.061438374
1385871_at	DEAH (Asp-Glu-Ala-His) box polypeptide 36 (predicted)	Dhx36_predicted	0.7	0.000318832	0.061438374
1381445_at	Estrogen-related receptor gamma	Esrrg	0.7	0.000320104	0.061438374
1378741_at	similar to putative phosphatase subunit	RGD1309207	0.8	0.000328852	0.062137848
1384519_at	SEC63-like (S. cerevisiae) (predicted)	Sec63_predicted	0.7	0.000337994	0.062137848
1394566_at	similar to hypothetical protein FLJ13188 (predicted)	RGD1305500_predicted	0.6	0.000338299	0.062137848
1391212_at	transcription elongation factor A (SII)-like 1	Tceal1	0.7	0.000345436	0.062681954
1375676_at	Transcribed locus	---	0.6	0.000359406	0.062681954
1389202_at	similar to Ribulose-5-phosphate-3-epimerase	MGC124653	0.7	0.000365922	0.062681954
1377105_at	zinc finger protein 91	Zfp91	0.6	0.00037363	0.062681954
1382419_at	SoxLZ/Sox6 leucine zipper binding protein in testis (predicted)	Solt_predicted	0.8	0.000376444	0.062681954
1374959_at	NAD(P)H dehydrogenase, quinone 2	Nqo2	0.7	0.000377956	0.062681954
1397469_at	dystonin (predicted)	Dst_predicted	0.7	0.000381963	0.062737351
1392449_at	RAD18 homolog (S. cerevisiae) (predicted)	Rad18_predicted	0.7	0.000399022	0.063382433
1376523_at	AT rich interactive domain 4A (Rbp1 like) (predicted)	Arid4a_predicted	0.6	0.000400405	0.063382433
1368158_at	sec1 family domain containing 1	Scfd1	0.7	0.000400732	0.063382433
1390435_at	LOC361111 (predicted)	RGD1307583_predicted	0.8	0.000408792	0.063629048
1373177_x_at	---	---	0.7	0.000417733	0.063711755
1393248_at	NMD3 homolog (S. cerevisiae) (predicted)	Nmd3_predicted	0.7	0.000425164	0.064271321
1381847_at	Similar to BC021442 protein (predicted)	RGD1561413_predicted	0.7	0.000433101	0.064896738
1384795_at	zinc finger, matrin-like (predicted)	Zfml_predicted	0.5	0.000438435	0.064896995
1396403_at	similar to KIAA2026 protein	RGD1311595	0.7	0.000442699	0.064896995
1376208_at	---	---	0.6	0.000449658	0.064970017
1370442_at	thymosin beta-like protein 1	Tmsbl1	0.7	0.00045641	0.064970017
1390655_at	---	---	0.5	0.000470623	0.065894944
1391968_at	Transcribed locus	---	0.6	0.000477815	0.066357972
1376853_at	similar to RIKEN cDNA 2310042P20	MGC94954	0.6	0.000495496	0.067677153
1381826_at	---	---	0.7	0.000504681	0.067677153
1393166_at	---	---	0.7	0.000506946	0.067677153
1387706_at	gamma-aminobutyric acid A receptor, gamma 1	Gabrg1	0.7	0.00052721	0.068488178
1392818_at	growth arrest specific 5	Gas5	0.7	0.000539693	0.069012738
1394059_s_at	YME1-like 1 (S. cerevisiae)	Yme1l1	0.7	0.000546984	0.069012738

1397468_at	similar to RIKEN cDNA 2610207I16	RGD1305387	0.6	0.000549781	0.069012738
1383007_at	Bardet-Biedl syndrome 4 homolog (human) (predicted)	Bbs4_predicted	0.6	0.000560415	0.069012738
1377182_at	integrin beta 3 binding protein (beta3-endonexin)	Itgb3bp	0.8	0.000573546	0.069012738
1396480_at	Hyaluronoglucosaminidase 3	Hyal3	0.7	0.00057678	0.069012738
1373387_at	similar to CG33714-PB, isoform B	LOC681987 /// LOC688717	0.8	0.000590882	0.069012738
1381098_at	similar to RIKEN cDNA 1810048J11 (predicted) /// similar to B0511.12	LOC690217 /// RGD1305147_predicted	0.5	0.00059305	0.069012738
1388101_at	dihydropyrimidinase-like 3	Dpysl3	0.4	0.000596405	0.069012738
1382477_at	SUMO/sentrin specific protease 5 (predicted) /// similar to SUMO/sentrin specific protease 5 (predicted) /// similar to SUMO/sentrin specific protease 5	LOC686286 /// RGD1564247_predicted /// Senp5_predicted	0.7	0.00059664	0.069012738
1380503_at	hypothetical LOC305452 (predicted)	RGD1309634_predicted	0.7	0.000603396	0.069012738
1398308_at	replication protein A3 (predicted)	Rpa3_predicted	0.7	0.000603978	0.069012738
1388911_at	DNA primase, p58 subunit	Prim2	0.7	0.000619172	0.069012738
1398983_at	mitochondrial ribosomal protein L30 (predicted)	Mrpl30_predicted	0.8	0.000622792	0.069012738
1373982_at	similar to hypothetical protein	RGD1306595	0.8	0.000629178	0.069012738
1398384_at	exosome component 9	Exosc9	0.7	0.000630345	0.069012738
1371623_at	similar to CG7224-PA, isoform A /// hypothetical protein LOC685888	LOC684207 /// LOC685888	0.8	0.000632198	0.069012738
1385972_at	kelch repeat and BTB (POZ) domain containing 3 (predicted)	Kbtbd3_predicted	0.7	0.00063455	0.069012738
1384759_at	PHD finger protein 17 (predicted)	Phf17_predicted	0.8	0.000641591	0.069012738
1391280_at	similar to malignant T cell amplified sequence 1	LOC682340 /// LOC689500	0.8	0.000649588	0.069012738
1397700_x_at	Non-coding RNA expressed in the brain, repeat sequence, clone 3 /// Non-coding RNA expressed in the brain, repeat sequence, clone 2 /// Non-coding RNA expressed in the brain, repeat sequence, clone 12 /// Non-coding RNA expressed in the brain, repeat sequence, clone 7	---	0.6	0.000652561	0.069012738
1373499_at	growth arrest specific 5	Gas5	0.7	0.000652598	0.069012738
1393088_at	RNA (guanine-9-) methyltransferase domain containing 1	Rg9mtd1	0.7	0.00065277	0.069012738
1381850_at	protein phosphatase 1, regulatory (inhibitor) subunit 12A	Ppp1r12a	0.6	0.000656651	0.069012738
1372626_at	similar to Tumor protein D53 (mD53) (Tumor protein D52-like 1)	LOC689256	0.8	0.000657804	0.069012738
1392902_at	similar to chromosome 20 open reading frame 6	RGD1306067	0.8	0.000666301	0.069198114
1390808_at	similar to microfibrillar-associated protein 1 (predicted)	RGD1562232_predicted /// RGD1564148_predicted	0.8	0.000672749	0.069198114
1392514_at	brix domain containing 1 (predicted)	Bxdc1_predicted	0.8	0.000676997	0.069198114
1381193_at	similar to lysophosphatidylglycerol acyltransferase 1	LOC679692 /// LOC683760	0.7	0.000680469	0.069198114
1385999_at	YME1-like 1 (S. cerevisiae)	Yme1l1	0.7	0.000694497	0.069376603
1392995_at	similar to zinc finger protein 322a	LOC680201 /// LOC684943	0.7	0.000703258	0.069439606
1374061_at	CD302 antigen	Cd302	0.7	0.000718095	0.069459846
1374953_at	similar to CG12279-PA	LOC500420	0.7	0.000722467	0.069459846
1382957_at	similar to cisplatin resistance-associated overexpressed protein (predicted)	RGD1307981_predicted	0.6	0.000722671	0.069459846
1376917_at	zinc finger protein 292	Znf292	0.6	0.000725877	0.069459846
1391566_at	Transcribed locus	---	0.8	0.00072711	0.069459846
1390806_at	similar to RIKEN cDNA B230118H07 (predicted)	RGD1309730_predicted	0.8	0.000735115	0.069459846
1383827_at	tousled-like kinase 1 (predicted)	Tlk1_predicted	0.4	0.000738443	0.069459846
1379101_at	DEAH (Asp-Glu-Ala-His) box polypeptide 36 (predicted)	Dhx36_predicted	0.7	0.000752265	0.069652105
1389912_at	endosulfine alpha	Ensa	0.7	0.000759333	0.069652105

1380121_at	NIMA (never in mitosis gene a)-related expressed kinase 7 (predicted)	Nek7_predicted	0.7	0.0007622	0.069652105
1393842_at	similar to RIKEN cDNA 2700091N06 (predicted)	RGD1310710_predicted	0.7	0.000767618	0.069652105
1396676_at	Transcribed locus	---	0.7	0.000778707	0.069652105
1394706_at	Transcribed locus	---	0.5	0.000778805	0.069652105
1382478_at	BTB (POZ) domain containing 3 (predicted)	Btbd3_predicted	0.7	0.000845762	0.0739693
1392936_at	RNA binding motif protein 25 (predicted) /// similar to RNA binding motif protein 25 (predicted)	Rbm25_predicted /// RGD1565486_predicted	0.7	0.000847793	0.0739693
1373868_at	---	---	0.7	0.00085236	0.0739693
1393142_at	---	---	0.8	0.000857389	0.0739693
1393813_at	tetratricopeptide repeat domain 3 (predicted)	Ttc3_predicted	0.5	0.000881143	0.074076268
1379810_at	small inducible cytokine subfamily E, member 1	Scye1	0.8	0.000885297	0.074076268
1392738_at	similar to KIAA1096 protein (predicted) /// similar to HBxAg transactivated protein 2	LOC367520 /// LOC684194 /// RGD1566064_predicted	0.6	0.000888985	0.074076268
1393158_at	similar to 2610528M18Rik protein	LOC363198	0.8	0.000902566	0.074182573
1376690_at	SRB7 (suppressor of RNA polymerase B) homolog (S. cerevisiae) (predicted)	Surb7_predicted	0.8	0.000903289	0.074182573
1385101_a_at	similar to RIKEN cDNA 0610011N22 gene	RGD735106	0.5	0.000927343	0.075075244
1372847_at	ACN9 homolog (S. cerevisiae)	Acn9	0.8	0.0009318	0.075080197
1384314_at	mitochondrial translational initiation factor 2	Mtif2	0.8	0.000939492	0.075265191
1382206_a_at	A kinase (PRKA) anchor protein 2	Akap2	0.6	0.000944191	0.075265191
1382040_at	glutamyl-prolyl-tRNA synthetase	Eprs	0.6	0.000955423	0.075265191
1382787_at	kinesin family member 5A	Kif5a	0.6	0.000958591	0.075265191
1394095_at	similar to RIKEN cDNA 4930429M06Rik	RGD1307449	0.7	0.000967437	0.075265191
1375542_at	Radixin	Rdx	0.8	0.000973829	0.075271253
1392460_at	---	---	0.8	0.000979516	0.075369813
1381677_at	ELAV (embryonic lethal, abnormal vision, Drosophila)-like 2 (Hu antigen B)	Elavl2	0.6	0.00099089	0.07556419
1376554_at	---	---	0.7	0.00100354	0.076188778
1369981_at	immunoglobulin (CD79A) binding protein 1	Igbp1	0.8	0.001013569	0.076331462
1378391_at	Transcribed locus	---	0.7	0.001018825	0.076331462
1370169_at	upstream of NRAS	Unr	0.6	0.001053317	0.076489501
1389640_at	Transcribed locus	---	0.7	0.001056181	0.076489501
1367541_at	similar to 2810410A08Rik protein (predicted)	RGD1566062_predicted	0.7	0.001062292	0.076489501
1391549_at	DCN1, defective in cullin neddylation 1, domain containing 4 (S. cerevisiae) (predicted)	Dcun1d4_predicted	0.7	0.001064636	0.076489501
1386120_at	hypothetical protein LOC679389 /// hypothetical protein LOC689147	LOC679389 /// LOC689147	0.7	0.001072913	0.076489501
1393276_at	mediator of RNA polymerase II transcription, subunit 31 homolog (yeast) (predicted)	Med31_predicted	0.8	0.001074955	0.076489501
1379555_at	Transcribed locus	---	0.6	0.001077762	0.076489501
1373355_at	---	---	0.8	0.001086496	0.076489501
1385496_at	similar to RIKEN cDNA 0610011N22 gene	RGD735106	0.6	0.001086878	0.076489501
1385168_at	similar to receptor-interacting factor 1	RGD1306520	0.7	0.001095474	0.076489501
1392702_at	Transcribed locus, weakly similar to NP_055333.2 finger protein 229 [Homo sapiens]	---	0.8	0.001102516	0.076557639
1393922_at	similar to CDNA sequence BC020077 (predicted)	RGD1561825_predicted	0.6	0.00111739	0.076887011
1398756_at	nucleophosmin 1 /// similar to Nucleophosmin (NPM) (Nucleolar phosphoprotein B23) (Numatrin) (Nucleolar protein NO38)	LOC300303 /// Npm1	0.8	0.001125617	0.076887011
1392897_at	---	---	0.8	0.001127885	0.076887011
1381476_at	Transcribed locus	---	0.8	0.001139377	0.076887011
1397508_at	DEAD (Asp-Glu-Ala-Asp) box polypeptide 18	Ddx18	0.7	0.001144103	0.076887011
1388060_at	synaptotagmin XII	Syt12	0.7	0.001154932	0.076887011
1373885_at	chromobox homolog 5 (Drosophila HP1a) (predicted)	Cbx5_predicted	0.7	0.001169019	0.076887011
1392170_at	RWD domain containing 3	Rwdd3	0.7	0.001170984	0.076887011
1386266_at	Similar to male sterility domain containing 1 (predicted)	RGD1565966_predicted	0.8	0.001177272	0.076887011

1392541_at	similar to RIKEN cDNA A030007L17; EST AA673177 (predicted) /// similar to C44B7.7	LOC685702 /// RGD1304876_predicted	0.8	0.001177877	0.076887011
1392045_at	transmembrane protein 22	Tmem22	0.8	0.001179803	0.076887011
1392399_at	WD repeats and SOF domain containing 1 (predicted)	Wdsof1_predicted	0.8	0.00119782	0.077473761
1376681_at	similar to RIKEN cDNA 1500003O22	RGD1308302	0.8	0.001226655	0.077760255
1368775_at	gonadotropin inducible ovarian transcription factor 1	Giot1	0.8	0.001230689	0.077760255
1379689_at	Transcribed locus	---	0.6	0.001252908	0.077760255
1384352_at	PAP associated domain containing 4	Papd4	0.7	0.001254999	0.077760255
1373472_at	ARP6 actin-related protein 6 homolog (yeast) (predicted)	Actr6_predicted	0.8	0.001271022	0.077760255
1380489_at	Similar to RIKEN cDNA G430041M01 (predicted)	RGD1562563_predicted	0.7	0.001276711	0.077760255
1393847_at	NFKB inhibitor interacting Ras-like protein 1 (predicted)	Nkiras1_predicted	0.8	0.001285295	0.077760255
1393683_at	similar to KIAA0368 (predicted)	RGD1306148_predicted	0.5	0.001286894	0.077760255
1380546_at	similar to hypothetical protein FLJ10986	LOC298250	0.8	0.001287622	0.077760255
1388850_at	heat shock protein 1, alpha	Hspca	0.7	0.001304855	0.077760255
1382161_at	M-phase phosphoprotein 10 (U3 small nucleolar ribonucleoprotein) (predicted)	Mphosph10_predicted	0.7	0.001307206	0.077760255
1368588_at	DEAD (Asp-Glu-Ala-Asp) box polypeptide 52	Ddx52	0.7	0.001315151	0.077760255
1367500_at	similar to 26 proteasome complex subunit DSS1 (Deleted in split hand/split foot protein 1) (Split hand/foot deleted protein 1 homolog)	LOC679014 /// LOC680532	0.8	0.001317269	0.077760255
1384029_at	xeroderma pigmentosum, complementation group A (predicted)	Xpa_predicted	0.8	0.001332683	0.077760255
1391830_at	copine VIII (predicted)	Cpne8_predicted	0.7	0.001342538	0.077760255
1395587_at	similar to Alkaline phytoceramidase (aPHC) (Alkaline ceramidase) (predicted)	RGD1561254_predicted	0.8	0.001349247	0.077760255
1382948_at	similar to hypothetical protein E130310N06 (predicted)	RGD1559882_predicted	0.8	0.001349659	0.077760255
1398588_at	similar to step II splicing factor SLU7; DNA segment, Chr 11, ERATO Doi 730, expressed; DNA segment, Chr 3, Brigham & Womens Genetics 0878 expressed	LOC303057	0.7	0.00135763	0.077760255
1397335_at	sema domain, immunoglobulin domain (Ig), short basic domain, secreted, (semaphorin) 3D	Sema3d	0.6	0.001360788	0.077760255
1393615_at	similar to DEP domain containing 6 (predicted)	RGD1561030_predicted	0.5	0.001362291	0.077760255
1391649_a_at	Transcribed locus	---	0.8	0.001365823	0.077760255
1387077_at	cAMP-regulated phosphoprotein 19	Arpp19	0.8	0.001368654	0.077760255
1391078_at	replication factor C 1	Recc1	0.8	0.001376547	0.077760255
1389980_at	similar to Protein HSPC163 (predicted)	RGD1559740_predicted	0.7	0.001388147	0.077760255
1383643_at	similar to UPF0197 protein C11orf10 homolog (predicted)	RGD1560328_predicted	0.8	0.001388784	0.077760255
1375634_at	coiled-coil domain containing 53 (predicted)	Ccdc53_predicted	0.8	0.001394114	0.077760255
1385050_at	zinc finger protein 292	Znf292	0.7	0.001403051	0.07781467
1373273_at	PRP38 pre-mRNA processing factor 38 (yeast) domain containing A (predicted)	Prpf38a_predicted	0.7	0.001416558	0.078056888
1376753_at	Fucose-1-phosphate guanylyltransferase	Fpgt	0.8	0.001421402	0.078072015
1379737_a_at	RNA-binding region (RNP1, RRM) containing 2	Rnpc2	0.6	0.001442707	0.078485104
1377684_at	arginyl-tRNA synthetase-like (predicted)	Rarsl_predicted	0.8	0.001498879	0.079890524
1372697_at	mitochondrial ribosomal protein S15	Mrps15	0.7	0.001523039	0.080458803
1392154_at	zinc finger, MYND domain containing 11	Zmynd11	0.7	0.001535013	0.080458803
1383579_at	Transcribed locus, moderately similar to XP_577744.1 similar to IKEN cDNA 6720480D16 [Rattus norvegicus]	---	0.7	0.001539542	0.080458803
1389384_at	hormone-regulated proliferation associated protein 20	Hrpap20	0.8	0.001545004	0.080458803
1380695_at	Transcribed locus	---	0.5	0.001550012	0.080458803
1383161_a_at	---	---	0.8	0.001575556	0.080458803
1382415_at	LOC360807	LOC360807	0.7	0.001597517	0.080458803
1388353_at	proliferation-associated 2G4	Pa2g4	0.6	0.001601052	0.080458803
1398905_at	ATPase, H transporting, lysosomal V1 subunit G1 (predicted)	Atp6v1g1_predicted	0.7	0.001602231	0.080458803

1372927_at	---	---	0.8	0.00160248	0.080458803
1385006_at	alpha thalassemia/mental retardation syndrome X-linked homolog (human)	Atrx	0.6	0.001606162	0.080458803
1391050_at	metal response element binding transcription factor 2	Mtf2	0.8	0.001634078	0.081617875
1378656_at	Bardet-Biedl syndrome 5 homolog (human) (predicted)	Bbs5_predicted	0.8	0.001650242	0.081884988
1368712_at	zinc finger protein 386 (Kruppel-like)	Znf386	0.8	0.001661803	0.081884988
1377720_x_at	---	---	0.8	0.001662292	0.081884988
1397200_at	chromodomain helicase DNA binding protein 4	Chd4	0.7	0.001663394	0.081884988
1373875_at	similar to RIKEN cDNA 1190005P17 (predicted) /// hypothetical protein LOC690089	LOC690089 /// RGD1308261_predicted	0.8	0.001670353	0.081991316
1383162_at	---	---	0.7	0.001684867	0.082142371
1395318_at	similar to chromodomain helicase DNA binding protein 9	LOC680231 /// LOC682690	0.8	0.001697083	0.082356726
1384887_at	---	---	0.8	0.001717013	0.082920185
1375335_at	heat shock 90kDa protein 1, beta	Hspcb	0.7	0.001718402	0.082920185
1378474_at	Transcribed locus	---	0.8	0.001742668	0.083384476
1394814_at	translocated promoter region	Tpr	0.4	0.001765124	0.083561954
1388809_at	sphingomyelin phosphodiesterase, acid-like 3A	Smpdl3a	0.7	0.00177273	0.083605912
1390604_s_at	integrin beta 3 binding protein (beta3-endonexin)	Itgb3bp	0.8	0.00178076	0.083605912
1397555_at	---	---	0.5	0.001788648	0.083605912
1389874_at	similar to U2 small nuclear ribonucleoprotein auxiliary factor 35 kDa subunit related-protein 1 (U2(RNU2) small nuclear RNA auxillary factor 1-like 1) (SP2)	LOC498425	0.5	0.001805584	0.083605912
1383730_at	Tetratricopeptide repeat domain 9C	Ttc9c	0.8	0.001809057	0.083605912
1378404_at	Transcribed locus	---	0.8	0.001811176	0.083605912
1372815_at	mago-nashi homolog, proliferation-associated (Drosophila) (predicted)	Magoh_predicted	0.8	0.001811801	0.083605912
1382705_at	Transcribed locus	---	0.8	0.001839853	0.083605912
1390237_at	translocase of inner mitochondrial membrane 8 homolog a (yeast)	Timm8a	0.8	0.001839978	0.083605912
1384791_at	UDP-GlcNAc:betaGal beta-1,3-N-acetylglucosaminyltransferase 1 (predicted)	B3gnt1_predicted	0.6	0.001847123	0.083605912
1378245_at	similar to 6430514L14Rik protein (predicted)	RGD1311958_predicted	0.8	0.001875265	0.083605912
1373433_at	nucleosome binding protein 1 (predicted) /// similar to Nucleosome binding protein 1 (Nucleosome binding protein 45) (NBP-45) (GARP45 protein)	LOC680182 /// LOC681284 /// LOC682212 /// Nsbp1_predicted	0.7	0.001875798	0.083605912
1384956_at	Transcribed locus	---	0.7	0.001893078	0.083605912
1385594_at	Transcribed locus	---	0.8	0.00189309	0.083605912
1385020_at	similar to DNA segment, Chr 19, Brigham & Womens Genetics 1357 expressed (predicted) /// hypothetical protein LOC499339 /// similar to D19Bwg1357e protein	LOC499339 /// LOC683670 /// RGD1308750_predicted	0.8	0.001894128	0.083605912
1379307_at	sodium channel associated protein 1	Sap1	0.7	0.001909242	0.08362479
1391689_at	similar to Retinoblastoma-binding protein 2 (RBBP 2)	LOC312678	0.8	0.001921269	0.083936369
1394985_at	early endosome antigen 1 (predicted)	Eea1_predicted	0.5	0.001939906	0.084352863
1396612_at	ankyrin 2, neuronal	Ank2	0.6	0.001941622	0.084352863
1383628_at	Transcribed locus	---	0.8	0.001960287	0.084516929
1396392_at	Dynactin 6 (predicted)	Dctn6_predicted	0.7	0.001964244	0.084516929
1379862_at	similar to CG33331-PA	LOC362419	0.8	0.001978552	0.084533769
1393161_at	small nuclear RNA activating complex, polypeptide 3	Snapc3	0.7	0.00198443	0.084533769
1377917_at	Transcribed locus, weakly similar to XP_001074405.1 similar to zinc finger protein 709 [Rattus norvegicus]	---	0.7	0.0019979	0.0846852
1385347_at	Transcribed locus	---	0.6	0.002020348	0.085265264
1383256_at	deoxynucleotidyltransferase, terminal, interacting protein 2 (predicted)	Dnttip2_predicted	0.8	0.002021568	0.085265264
1391070_at	similar to hypothetical MGC48595 (predicted)	RGD1566220_predicted	0.8	0.002028738	0.085356891
1368843_at	YME1-like 1 (S. cerevisiae)	Yme1l1	0.7	0.002037595	0.085518905

1374323_at	BRCA2 and CDKN1A interacting protein (predicted)	Bccip_predicted	0.8	0.002056313	0.085882478
1383326_a_at	---	---	0.8	0.002063275	0.085963073
1376483_at	LSM5 homolog, U6 small nuclear RNA associated (S. cerevisiae) (predicted)	Lsm5_predicted	0.7	0.002069618	0.085968527
1381205_at	similar to small nuclear RNA activating complex, polypeptide 5	LOC686858 /// LOC691501	0.8	0.002090885	0.086480636
1383876_at	intraflagellar transport 74 homolog (Chlamydomonas)	Ift74	0.8	0.0021055	0.08683526
1371523_at	---	---	0.8	0.002109626	0.08683526
1391618_at	kinesin-associated protein 3 (predicted)	Kifap3_predicted	0.7	0.00213284	0.087394051
1386566_at	Transcribed locus, strongly similar to XP_509904.2 chromosome 14 open reading frame 10 isoform 6 [Pan troglodytes]	---	0.7	0.002134676	0.087394051
1370807_at	transmembrane protein 49	Tmem49	0.8	0.00219644	0.088340206
1373403_at	similar to Protein C8orf4 (Thyroid cancer protein 1) (TC-1)	LOC684871	0.7	0.002197903	0.088340206
1378743_at	similar to Peptidyl-prolyl cis-trans isomerase NIMA-interacting 4 (Rotamase Pin4) (PPIase Pin4)	LOC684441	0.8	0.00223674	0.089149533
1385273_at	Nuclear pore associated protein	Npap60	0.8	0.002240542	0.089149533
1384147_at	eukaryotic translation initiation factor 1A	Eif1a	0.7	0.002244134	0.089149533
1385491_at	similar to KIAA1183 protein (predicted)	RGD1560435_predicted	0.4	0.002253894	0.08928109
1385859_at	Glutaredoxin 2 (thioltransferase)	Glrx2	0.4	0.002257899	0.08928109
1372642_at	Transcribed locus	---	0.8	0.002268513	0.089464804
1382290_at	similar to CG9643-PA (predicted)	RGD1306300_predicted	0.7	0.002280935	0.089569968
1374780_at	Transcribed locus	---	0.8	0.002298971	0.089988014
1399130_at	similar to RIKEN cDNA 2610022G08	LOC502782	0.7	0.002305854	0.089988014
1386016_at	similar to Placental protein 25 homolog (PP25)	LOC683481 /// LOC685284	0.8	0.002324245	0.090439072
1377719_a_at	---	---	0.8	0.002345144	0.090683187
1375622_at	zinc finger, FYVE domain containing 20 (predicted)	Zfyve20_predicted	0.5	0.00234648	0.090683187
1390312_at	similar to mKIAA2005 protein (predicted) /// similar to sterile alpha motif domain containing 9-like /// similar to mKIAA2005 protein	LOC500013 /// LOC500015 /// RGD1561472_predicted	0.8	0.00235784	0.090683187
1382882_x_at	Non-coding RNA expressed in the brain, repeat sequence, clone 3 /// Non-coding RNA expressed in the brain, repeat sequence, clone 2 /// Non-coding RNA expressed in the brain, repeat sequence, clone 12 /// Non-coding RNA expressed in the brain, repeat sequence, clone 7	---	0.7	0.002364059	0.090683187
1384761_at	Transcribed locus, strongly similar to XP_001063039.1 similar to nuclear NF-kappaB activating protein [Rattus norvegicus]	---	0.7	0.002365716	0.090683187
1394884_s_at	single-stranded DNA binding protein 1	Ssbp1	0.8	0.00236768	0.090683187
1380696_at	Transcribed locus	---	0.7	0.002394183	0.091144684
1368060_at	heat-responsive protein 12	Hrsp12	0.8	0.002401748	0.091144684
1372143_at	ubiquitin-conjugating enzyme E2 variant 2	Ube2v2	0.8	0.002406407	0.091144684
1383819_at	Transcribed locus	---	0.7	0.002439207	0.091899558
1397917_at	---	---	0.7	0.002447857	0.091899558
1399018_at	similar to cisplatin resistance-associated overexpressed protein (predicted)	RGD1307981_predicted	0.8	0.002475468	0.09261177
1379200_at	similar to Ubiquitin ligase protein DZIP3 (DAZ-interacting protein 3 homolog) (predicted)	RGD1563278_predicted	0.5	0.002477687	0.09261177
1391193_at	similar to AD24 (predicted)	RGD1560656_predicted	0.8	0.00248762	0.09261177
1393369_at	Similar to RIKEN cDNA 4921511I16	RGD1305302	0.7	0.002507777	0.092923756
1381905_at	---	---	0.8	0.002540266	0.093518999
1383341_at	calcium binding protein 39-like	Cab39l	0.8	0.002547884	0.093597748
1390892_at	DEP domain containing 1B (predicted)	Depdc1b_predicted	0.8	0.002597337	0.094802787
1377503_at	RIO kinase 2 (yeast)	Riok2	0.8	0.002618574	0.09496919
1397708_at	hypothetical LOC288978	LOC288978	0.8	0.002626512	0.095055243

1382067_at	similar to UBX domain containing 4	LOC363500 /// LOC679377 /// LOC683882 /// LOC685859	0.7	0.002646959	0.095391055
1385077_at	similar to golgi-specific brefeldin A-resistance guanine nucleotide exchange factor 1 (predicted)	RGD1307160_predicted	0.7	0.002659073	0.095499738
1373866_at	similar to hypothetical protein FLJ13448	RGD1359509	0.8	0.00266347	0.095499738
1379378_at	mitochondrial ribosomal protein S18C (predicted)	Mrps18c_predicted	0.8	0.002678365	0.095515288
1396215_at	Similar to RIKEN cDNA 2610022G08	LOC502782	0.8	0.002702675	0.095518141
1392856_at	similar to small EDRK-rich factor 1 (predicted)	RGD1565105_predicted	0.8	0.002706403	0.095518141
1399062_at	CDNA clone IMAGE:7317308	---	0.8	0.00274678	0.095833195
1396063_at	DEK oncogene (DNA binding)	Dek	0.6	0.002756314	0.095833195
1394362_at	similar to intracellular protein transport like (XM453) (predicted)	RGD1307525_predicted	0.4	0.002757876	0.095833195
1385595_at	similar to antigenic determinant of rec-A protein	LOC683353 /// LOC689197	0.7	0.00277765	0.096048203
1393008_at	CAMP-regulated phosphoprotein 19	Arpp19	0.8	0.002784538	0.096058869
1382385_at	proteasome (prosome, macropain) 26S subunit, ATPase, 6	Psmc6	0.8	0.002820313	0.096058869
1384153_at	similar to Hypothetical protein KIAA0555 (predicted)	RGD1559742_predicted	0.7	0.00282541	0.096058869
1370165_at	small muscle protein, X-linked	Smpx	0.8	0.002826487	0.096058869
1389907_at	zinc finger and BTB domain containing 8 opposite strand (predicted)	Zbtb8os_predicted	0.8	0.002847667	0.096058869
1393242_at	similar to chromosome 16 open reading frame 33; minus -99 protein (predicted)	RGD1310922_predicted	0.8	0.002863195	0.096058869
1382014_at	---	---	0.8	0.002864585	0.096058869
1385163_at	ring finger protein 20 (predicted)	Rnf20_predicted	0.7	0.002868392	0.096058869
1377685_at	similar to establishment of cohesion 1 homolog 1 (predicted) /// similar to N-acetyltransferase ESCO1 (Establishment of cohesion 1 homolog 1) (ECO1 homolog 1)	LOC680014 /// LOC682499 /// LOC683403 /// RGD1562794_predicted	0.8	0.002878249	0.096058869
1393030_at	---	---	0.8	0.002935912	0.09627783
1391025_at	Transcribed locus	---	0.7	0.002944049	0.09627783
1397614_at	similar to Gamma-aminobutyric-acid receptor alpha-2 subunit precursor (GABA(A) receptor)	LOC289606	0.6	0.002959614	0.09627783
1382904_at	similar to hypothetical protein DKFZp434K1421	RGD1309863	0.7	0.002967716	0.09627783
1382413_at	Nucleoporin 155	Nup155	0.8	0.002972548	0.09627783
1382812_at	similar to Protein Njmu-R1 (predicted)	RGD1310429_predicted	0.7	0.002974116	0.09627783
1390836_at	---	---	0.8	0.002982836	0.09627783
1395399_at	similar to PS1D protein (predicted)	RGD1565267_predicted	0.8	0.002989622	0.09627783
1378530_at	Transcribed locus, strongly similar to XP_580104.1 hypothetical protein XP_580104 [Rattus norvegicus]	---	0.8	0.002995969	0.09627783
1387435_at	ST8 alpha-N-acetyl-neuraminide alpha-2,8-sialyltransferase 3	St8sia3	0.8	0.003002487	0.09627783
1371634_at	similar to RIKEN cDNA 1810020E01	RGD1305677	0.8	0.003006062	0.09627783
1383236_at	fragile X mental retardation gene 1, autosomal homolog	Fxr1h	0.8	0.003042504	0.096962183
1389126_at	coiled-coil-helix-coiled-coil-helix domain containing 1 (predicted)	Chchd1_predicted	0.8	0.003048161	0.096962183
1379829_at	---	---	0.8	0.003057755	0.097086559
1394626_at	similar to hypothetical protein FLJ21616 (predicted)	RGD1306787_predicted	0.5	0.003085464	0.097459196
1385487_at	LSM1 homolog, U6 small nuclear RNA associated (S. cerevisiae) (predicted)	Lsm1_predicted	0.8	0.003093189	0.097459196
1388689_at	similar to Acylphosphatase, muscle type isozyme (Acylphosphate phosphohydrolase)	LOC682245	0.7	0.003094799	0.097459196
1393144_at	N-myc (and STAT) interactor	Nmi	0.8	0.003099506	0.097459196
1394412_at	transmembrane protein 16C (predicted)	Tmem16c_predicted	0.7	0.003111997	0.097459196
1394710_at	5-azacytidine induced gene 2	Azi2	0.8	0.003112099	0.097459196
1369984_at	cytochrome c oxidase, subunit XVII assembly protein homolog (yeast)	Cox17	0.8	0.003140361	0.097459196

1372338_at	similar to CG9240-PA	LOC687395	0.7	0.003140884	0.097459196
1388977_at	choline/ethanolamine phosphotransferase 1	Cept1	0.8	0.003147141	0.097459196
1395232_at	similar to IQ motif and WD repeats 1 (predicted)	RGD1561961_predicted	0.7	0.00314911	0.097459196
1370024_at	Fatty acid binding protein 7, brain	Fabp7	0.7	0.003154951	0.097459196
1382735_at	autophagy-related 12 (yeast)	Atg12	0.7	0.003155072	0.097459196
1371089_at	glutathione S-transferase Yc2 subunit	Yc2	0.8	0.003181452	0.097873471
1368877_at	zinc finger protein 354A	Zfp354a	0.8	0.003196199	0.097873471
1393647_at	histone aminotransferase 1	Hat1	0.8	0.003200141	0.097873471
1392506_at	crystallin, zeta (quinone reductase)-like 1	Cryzl1	0.8	0.003201373	0.097873471
1391264_at	Transcribed locus	---	0.8	0.003202861	0.097873471
1384118_at	zinc finger protein 329 (predicted)	Zfp329_predicted	0.7	0.003223849	0.098163604
1391985_at	Nuclear receptor interacting protein 3 (predicted)	Nrip3_predicted	0.6	0.003231903	0.098233738
1368405_at	v-ral simian leukemia viral oncogene homolog A (ras related)	Rala	0.7	0.003252302	0.098509925
1380310_at	similar to hypothetical protein FLJ14007 (predicted)	RGD1309519_predicted	0.8	0.003252523	0.098509925
1379826_at	similar to hypothetical protein MGC31967 (predicted)	RGD1560252_predicted	0.8	0.003267751	0.098795964
1390881_at	actin-binding Rho activating protein	Abra	0.6	0.00328545	0.098971977
1392599_at	synapse associated protein 1	Syap1	0.8	0.003290954	0.098971977
1376606_a_at	similar to e(y)2 protein	LOC682575 /// LOC685258	0.8	0.003308756	0.099332467
1383577_at	Transcribed locus	---	0.8	0.003323164	0.099589989
1368164_at	biliverdin reductase A	Blvra	0.8	0.00333888	0.099885731
1382551_at	similar to Intersectin-2 (SH3 domain-containing protein 1B) (SH3P18) (SH3P18-like WASP-associated protein)	LOC313934	0.6	0.003351334	0.10000794
1388171_at	cyclin-dependent kinase 7 (homolog of Xenopus MO15 cdk-activating kinase)	Cdk7	0.8	0.003354675	0.10000794
1382063_at	galactosidase, alpha	Gla	0.8	0.003364004	0.100111344
1391695_at	---	---	0.8	0.003370494	0.100130035
1385282_at	zinc finger protein 628 (predicted)	Zfp628_predicted	0.7	0.003382101	0.100300427
1385787_at	ring finger protein 141	rnf141	0.8	0.00340284	0.10043969
1396117_at	similar to leucine zipper protein 2 (predicted)	RGD1563838_predicted	0.6	0.003405577	0.10043969
1383912_at	similar to Protein C6orf115	LOC684848 /// LOC685045	0.7	0.003426626	0.10043969
1389011_at	similar to RIKEN cDNA 1110014D18	RGD1305156	0.8	0.003435723	0.10043969
1379275_at	sorting nexin 10	Snx10	0.7	0.003460795	0.10043969
1381958_at	similar to mKIAA0259 protein (predicted)	RGD1562949_predicted	0.5	0.003463891	0.10043969
1398884_at	prefoldin 5 (predicted)	Pfdn5_predicted	0.8	0.003463912	0.10043969
1376704_a_at	necdin-like 2	Ndnl2	0.8	0.003470057	0.10043969
1376324_at	Transcribed locus, weakly similar to NP_055333.2 finger protein 229 [Homo sapiens]	---	0.7	0.003474703	0.10043969
1368584_a_at	complexin 2	Cplx2	0.7	0.003474995	0.10043969
1373913_at	polyribonucleotide nucleotidyltransferase 1	Pnpt1	0.8	0.003488752	0.100666988
1374515_at	similar to RIKEN cDNA 6330409N04	RGD1306437	0.8	0.003511477	0.101066225
1375612_at	Transcribed locus	---	0.7	0.003517909	0.101066225
1393118_at	armadillo repeat containing 1 (predicted)	Armc1_predicted	0.8	0.003539544	0.101277209
1377624_at	claudin 12 (predicted)	Cldn12_predicted	0.8	0.003549703	0.101391987
1372461_at	SET translocation (predicted)	Set_predicted	0.8	0.003569589	0.101391987
1373899_at	similar to dJ842G6.1.1 (novel protein) (predicted)	RGD1309829_predicted	0.8	0.003569726	0.101391987
1372827_at	peptidylprolyl isomerase D (cyclophilin D)	Ppid	0.8	0.003579169	0.101391987
1381878_at	ubinuclein 1 (predicted)	Ubn1_predicted	0.7	0.003619423	0.101907022
1382193_at	similar to RIKEN cDNA 1500009M05 (predicted)	RGD1566242_predicted	0.6	0.003630279	0.101907022
1392663_at	Transcribed locus, strongly similar to XP_001065986.1 hypothetical protein [Rattus norvegicus]	---	0.8	0.00364784	0.102151478
1386078_at	solute carrier family 35 (CMP-sialic acid transporter), member 1 (predicted)	Slc35a1_predicted	0.8	0.003677168	0.102267664
1395053_at	androgen-induced proliferation inhibitor (predicted)	Aprin_predicted	0.7	0.003687743	0.102267664
1395998_at	nucleolar protein 5A	Nol5a	0.7	0.003690958	0.102267664

1384323_at	proteasome (prosome, macropain) 26S subunit, ATPase, 6	Psmc6	0.7	0.003700573	0.102267664
1392551_at	zinc finger protein ZFP	LOC503192	0.8	0.003702528	0.102267664
1370088_at	sperm autoantigenic protein 17	Spa17	0.8	0.003711858	0.102267664
1393550_at	CAMP responsive element modulator	Crem	0.8	0.003758338	0.102371868
1390851_at	Lactamase, beta 2	Lactb2	0.8	0.003773214	0.102371868
1382452_at	serum deprivation response protein	Sdpr	0.6	0.003785381	0.102371868
1394318_at	---	---	0.8	0.003794051	0.102371868
1384166_a_at	---	---	0.8	0.003802495	0.102371868
1379850_at	proteasome (prosome, macropain) 26S subunit, ATPase, 6	Psmc6	0.7	0.003807203	0.102371868
1367818_at	coenzyme Q3 homolog, methyltransferase (yeast)	Coq3	0.8	0.003810446	0.102371868
1377111_at	Similar to TAK1-binding protein 3 isoform 1	LOC317546	0.6	0.003816892	0.102371868
1395058_at	WD repeat domain 75	Wdr75	0.6	0.003820882	0.102371868
1390933_a_at	RNA (guanine-9-) methyltransferase domain containing 3	Rg9mtd3	0.8	0.003821123	0.102371868
1382371_at	transmembrane protein 77	Tmem77	0.7	0.003835012	0.102371868
1376659_at	nucleotide binding protein-like (predicted)	Nubpl_predicted	0.8	0.003853362	0.10243669
1391342_at	Transcribed locus	---	0.8	0.003881117	0.102461219
1391578_at	similar to modulator of estrogen induced transcription	RGD1307526	0.7	0.003886832	0.102461219
1385334_at	Transcribed locus	---	0.8	0.003893305	0.102473707
1373074_at	similar to RIKEN cDNA 2700002I20	RGD1307279	0.8	0.003915895	0.102525381
1375215_x_at	pyroglutamyl-peptidase I	Pgpep1	0.8	0.003919721	0.102525381
1373592_at	similar to SPI6	MGC94010	0.8	0.003920853	0.102525381
1393167_at	---	---	0.5	0.003925278	0.102525381
1375425_at	hypothetical LOC287541 (predicted)	RGD1309400_predicted	0.8	0.003954967	0.103143121
1388313_at	ribosomal protein s25	Rps25	0.8	0.003979065	0.103613408

Supplementary table 3: Probe sets identified as up-regulated 10% FDR (p<0.03) and >1.2 fold in the L5 DRGs of SNT animals

Probe set ID	Gene Title	Gene Symbol	Fold increase	p value	FDR value
1368266_at	arginase 1	Arg1	18.0	2.08E-09	1.68E-05
1368892_at	Adenylate cyclase activating polypeptide 1 (Adcyap1) mRNA, 3' UTR	---	3.9	3.42E-09	1.68E-05
1384803_at	late cornified envelope 1F (predicted) /// RGD1561089 (predicted) /// late cornified envelope 1S (predicted) /// late cornified envelope 1L (predicted)	Lce1f_predicted /// Lce1l_predicted /// Lce1s_predicted /// RGD1561089_predicted	22.6	3.88E-09	1.68E-05
1394908_at	Adenylate cyclase activating polypeptide 1 (Adcyap1) mRNA, 3' UTR	---	5.8	4.31E-09	1.68E-05
1376390_at	membrane-spanning 4-domains, subfamily A, member 11 (predicted)	Ms4a11_predicted	11.2	8.14E-09	2.23E-05
1396451_at	Transcribed locus	---	6.8	1.06E-08	2.29E-05
1368238_at	pancreatitis-associated protein	Pap	22.3	1.11E-08	2.29E-05
1379573_at	---	---	7.2	1.54E-08	2.62E-05
1370249_at	benzodiazepine receptor, peripheral	Bzrp	5.3	1.59E-08	2.62E-05
1378925_at	---	---	2.9	2.21E-08	3.31E-05
1373032_at	musculoskeletal, embryonic nuclear protein 1	Mustn1	4.7	2.76E-08	3.79E-05
1393573_at	phosphodiesterase 6B, cGMP, rod receptor, beta polypeptide (predicted)	Pde6b_predicted	8.7	4.22E-08	5.24E-05
1397823_at	Calcium channel, voltage-dependent, alpha2/delta subunit 1	Cacna2d1	5.6	4.71E-08	5.24E-05
1380100_at	similar to Traf2 and NCK interacting kinase, splice variant 4 (predicted)	RGD1561817_predicted	3.4	4.78E-08	5.24E-05
1388385_at	crystallin, beta A2	Cryba2	5.1	5.87E-08	5.44E-05
1370628_at	granzyme B	Gzmb	15.5	6.12E-08	5.44E-05
1378057_at	fibronectin leucine rich transmembrane protein 3 (predicted)	Flrt3_predicted	6.0	6.44E-08	5.44E-05
1368000_at	complement component 3	C3	6.4	6.89E-08	5.44E-05
1371248_at	similar to Cornifin A (Small proline-rich protein 1A) (SPR1A) (SPRR1)	LOC499660	19.5	7.06E-08	5.44E-05
1373911_at	periostin, osteoblast specific factor (predicted)	Postn_predicted	12.6	7.59E-08	5.44E-05
1387908_at	RAS, dexamethasone-induced 1	Rasd1	8.5	7.76E-08	5.44E-05
1382017_at	---	---	7.4	8.15E-08	5.44E-05
1369224_at	cadherin 17	Cdh17	3.2	8.39E-08	5.44E-05
1392722_at	Transcribed locus	---	2.6	8.60E-08	5.44E-05
1387088_at	galanin	Gal	6.8	8.96E-08	5.46E-05
1383805_at	similar to Traf2 and NCK interacting kinase, splice variant 4 (predicted)	RGD1561817_predicted	3.7	9.43E-08	5.54E-05
1386967_at	ras homolog gene family, member Q	Rhoq	3.0	9.93E-08	5.63E-05
1383536_at	Dihydrofolate reductase	Dhfr	3.9	1.20E-07	5.82E-05
1392794_at	Transcribed locus	---	3.2	1.21E-07	5.82E-05
1379766_at	Src-like adaptor	Sla	7.1	1.28E-07	5.82E-05
1376601_at	sema domain, transmembrane domain (TM), and cytoplasmic domain, (semaphorin) 6A (predicted)	Sema6a_predicted	7.2	1.32E-07	5.82E-05
1384035_at	similar to liver-specific bHLH-Zip transcription factor	LOC685277 /// LOC686794	12.9	1.33E-07	5.82E-05
1392863_at	fibronectin leucine rich transmembrane protein 3 (predicted)	Flrt3_predicted	11.8	1.35E-07	5.82E-05
1387444_at	protein tyrosine phosphatase, receptor type, H	Ptprh	3.6	1.36E-07	5.82E-05
1367974_at	annexin A3	Anxa3	4.7	1.40E-07	5.82E-05
1370391_at	cellular retinoic acid binding protein 2	Crabp2	4.4	1.41E-07	5.82E-05
1383391_a_at	complement component 2	C2	6.5	1.42E-07	5.82E-05
1379794_at	granzyme B	Gzmb	18.8	1.49E-07	5.99E-05
1390713_at	Transcribed locus	---	4.5	1.75E-07	6.70E-05
1373781_a_at	similar to suprabasal-specific protein suprabasin (predicted)	RGD1562305_predicted	5.1	2.13E-07	7.95E-05
1368010_at	protein tyrosine phosphatase, non-receptor type 6	Ptpn6	2.6	2.29E-07	8.25E-05
1386552_at	Transcribed locus	---	4.2	2.31E-07	8.25E-05
1369904_at	gamma-aminobutyric acid (GABA-A) receptor, subunit beta 1	Gabrb1	2.6	2.52E-07	8.81E-05

1388337_at	nucleoside phosphorylase	Np	2.2	2.69E-07	9.22E-05
1389696_at	Transcribed locus	---	6.3	2.80E-07	9.39E-05
1368558_s_at	allograft inflammatory factor 1	Aif1	8.9	2.94E-07	9.66E-05
1368657_at	matrix metallopeptidase 3	Mmp3	7.9	3.60E-07	0.000115986
1370154_at	lysozyme	Lyz	5.5	4.13E-07	0.000130696
1389123_at	chemokine (C-C motif) ligand 6	Ccl6	5.2	4.35E-07	0.000133136
1370315_a_at	stathmin-like 4	Stmn4	4.0	4.43E-07	0.000133136
1382692_at	similar to Clecsf12 protein (predicted)	RGD1565140_predicted	11.8	4.47E-07	0.000133136
1367664_at	ankyrin repeat domain 1 (cardiac muscle)	Ankrd1	11.0	4.53E-07	0.000133136
1384036_s_at	---	---	6.8	4.65E-07	0.000133136
1373544_at	chemokine (C-X-C motif) ligand 9	Cxcl9	11.6	4.70E-07	0.000133136
1388496_at	filamin C, gamma (actin binding protein 280) (predicted)	Flnc_predicted	6.3	4.78E-07	0.000133136
1390226_at	similar to hypothetical protein LOC340061 (predicted)	RGD1562552_predicted	2.2	5.04E-07	0.000138183
1370177_at	poliovirus receptor	PVR	5.4	5.47E-07	0.000142833
1389553_at	dendritic cell inhibitory receptor 3	Dcir3	8.8	5.50E-07	0.000142833
1394077_at	Rho family GTPase 3	Rnd3	2.9	5.55E-07	0.000142833
1377994_at	Transcribed locus	---	3.5	5.56E-07	0.000142833
1369843_at	cholinergic receptor, nicotinic, alpha polypeptide 1 (muscle)	Chrna1	3.5	5.87E-07	0.00014858
1368420_at	ceruloplasmin	Cp	4.9	6.65E-07	0.000165794
1387029_at	complement component factor H	Cfh	5.9	7.11E-07	0.000173276
1371079_at	Fc receptor, IgG, low affinity IIb	Fcgr2b	6.6	7.16E-07	0.000173276
1373161_at	transmembrane protein 98	Tmem98	2.6	7.66E-07	0.000179505
1374276_at	Similar to liver-specific bHLH-Zip transcription factor	LOC685277	7.4	7.75E-07	0.000179505
1393280_at	lymphocyte antigen 86 (predicted)	Ly86_predicted	3.1	8.59E-07	0.000190087
1379381_at	Transcribed locus	---	5.3	8.66E-07	0.000190087
1394451_at	annexin A1	Anxa1	3.4	8.82E-07	0.000190087
1395389_at	ST3 beta-galactoside alpha-2,3-sialyltransferase 6	St3gal6	3.4	8.83E-07	0.000190087
1383241_at	complement component 1, r subcomponent	C1r	3.5	9.01E-07	0.000190087
1395313_s_at	Annexin A3	Anxa3	3.7	9.01E-07	0.000190087
1370382_at	RT1 class II, locus Bb	RT1-Bb	3.6	9.45E-07	0.00019684
1397958_at	Transcribed locus	---	3.8	1.02E-06	0.000207881
1391605_at	Transcribed locus	---	2.7	1.04E-06	0.000207881
1390649_at	solute carrier family 30 (zinc transporter), member 3	Slc30a3	3.3	1.05E-06	0.000207881
1378700_at	Transcribed locus	---	3.5	1.06E-06	0.000207881
1393038_at	Fc receptor, IgG, high affinity I	Fcgr1	3.7	1.06E-06	0.000207881
1381089_at	Transcribed locus	---	7.6	1.14E-06	0.000211994
1383210_at	Transcribed locus	---	7.6	1.15E-06	0.000211994
1372599_at	microsomal glutathione S-transferase 2 (predicted)	Mgst2_predicted	3.1	1.16E-06	0.000211994
1375633_at	chloride intracellular channel 1	Clic1	2.6	1.16E-06	0.000211994
1393550_at	CAMP responsive element modulator	Crem	2.5	1.19E-06	0.000211994
1377916_at	schlafen 2 (predicted)	Slfn2_predicted	4.3	1.20E-06	0.000211994
1368223_at	a disintegrin-like and metallopeptidse (reprolysin type) with thrombospondin type 1 motif, 1	Adamts1	3.2	1.21E-06	0.000211994
1382868_at	sema domain, transmembrane domain (TM), and cytoplasmic domain, (semaphorin) 6A (predicted)	Sema6a_predicted	10.7	1.21E-06	0.000211994
1368188_at	4-hydroxyphenylpyruvic acid dioxygenase	Hpd	5.5	1.40E-06	0.000236121
1391582_at	Transcribed locus	---	2.4	1.41E-06	0.000236121
1389350_at	apolipoprotein H	Apoh	2.5	1.54E-06	0.000251478
1368590_at	matrix metalloproteinase 16	Mmp16	4.7	1.55E-06	0.000251478
1391637_at	similar to RIKEN cDNA 5830480G12	LOC295635	2.9	1.56E-06	0.000251478
1381070_at	---	---	12.7	1.61E-06	0.000257469
1388451_at	Transcribed locus	---	3.2	1.74E-06	0.0002692
1374529_at	Thrombospondin 1	Thbs1	5.0	1.75E-06	0.0002692
1368406_at	steroidogenic acute regulatory protein	Star	2.4	1.86E-06	0.000281113
1398202_at	Transcribed locus	---	6.3	1.90E-06	0.000281227

1390937_at	similar to chromosome 14 open reading frame 50	RGD1309051	2.1	1.92E-06	0.000282603
1376562_at	similar to Traf2 and NCK interacting kinase, splice variant 4 (predicted)	RGD1561817_predicted	6.6	1.97E-06	0.000286822
1369979_at	src family associated phosphoprotein 2	Scap2	3.6	2.13E-06	0.000292875
1376055_at	minichromosome maintenance deficient 5, cell division cycle 46 (S. cerevisiae) (predicted)	Mcm5_predicted	4.6	2.14E-06	0.000292875
1394568_at	similar to DNA segment, Chr 10, Wayne State University 102, expressed (predicted)	RGD1563365_predicted	2.1	2.14E-06	0.000292875
1390510_at	membrane-spanning 4-domains, subfamily A, member 6B	Ms4a6b	7.7	2.15E-06	0.000292875
1371785_at	tumor necrosis factor receptor superfamily, member 12a	Tnfrsf12a	3.5	2.17E-06	0.000292875
1376693_at	similar to OEF2 (predicted)	RGD1563091_predicted	2.6	2.17E-06	0.000292875
1372516_at	kinesin family member 22	Kif22	4.0	2.19E-06	0.000292875
1370118_at	chemokine (C-C motif) ligand 17	Ccl17	2.5	2.25E-06	0.000297935
1375043_at	FBJ murine osteosarcoma viral oncogene homolog	Fos	6.5	2.30E-06	0.000300604
1393477_at	---	---	5.0	2.33E-06	0.000301977
1393641_at	B-cell linker	Blnk	4.0	2.45E-06	0.000314033
1383786_at	Transcribed locus	---	3.8	2.46E-06	0.000314033
1393722_at	fem-1 homolog c (C.elegans) (predicted)	Fem1c_predicted	2.1	2.53E-06	0.000320146
1398656_at	---	---	2.2	2.62E-06	0.0003285
1375951_at	thrombomodulin	Thbd	4.5	2.70E-06	0.000333693
1387005_at	cathepsin S	Ctss	6.9	2.74E-06	0.000334824
1388742_at	---	---	3.8	2.75E-06	0.000334824
1371913_at	transforming growth factor, beta induced	Tgfbi	3.6	2.78E-06	0.000335206
1369725_at	centaurin, alpha 2	Centa2	2.2	2.79E-06	0.000335206
1376636_at	transforming growth factor, beta receptor 1	Tgfbr1	2.6	2.90E-06	0.000341135
1387154_at	neuropeptide Y	Npy	8.6	2.90E-06	0.000341135
1373513_at	Transcribed locus	---	4.3	2.96E-06	0.000345832
1383372_at	platelet-activating factor receptor	Ptafr	2.2	3.19E-06	0.000361299
1367849_at	syndecan 1	Sdc1	4.7	3.23E-06	0.000361299
1369691_at	sodium channel, voltage-gated, type III, alpha polypeptide	Scn3a	5.5	3.33E-06	0.000370614
1373490_at	glia maturation factor, gamma	Gmfg	4.3	3.45E-06	0.000375797
1371377_at	ribosomal protein S19	Rps19	2.0	3.47E-06	0.000375797
1393679_at	Transcribed locus	---	2.0	3.50E-06	0.000375879
1373373_at	Transcribed locus	---	2.7	3.52E-06	0.000376367
1392946_at	Transcribed locus	---	2.8	3.58E-06	0.000377436
1382960_at	Transcribed locus	---	6.0	3.66E-06	0.000378299
1388419_at	---	---	2.5	3.84E-06	0.00038745
1373403_at	similar to Protein C8orf4 (Thyroid cancer protein 1) (TC-1)	LOC684871	4.5	3.97E-06	0.000396667
1383556_at	Transcribed locus	---	2.2	3.98E-06	0.000396667
1384013_at	cell adhesion molecule with homology to L1CAM	Chl1	3.2	4.03E-06	0.000396667
1383688_at	Transcribed locus	---	2.8	4.04E-06	0.000396667
1372777_at	Transcribed locus	---	2.0	4.10E-06	0.000396667
1380180_at	Transcribed locus	---	3.9	4.10E-06	0.000396667
1374273_at	Coxsackie virus and adenovirus receptor	Cxadr	3.4	4.15E-06	0.000396667
1371450_at	Transcribed locus	---	4.4	4.16E-06	0.000396667
1389659_at	similar to ctla-2-beta protein (141 AA) (predicted)	RGD1565540_predicted	3.1	4.17E-06	0.000396667
1379957_at	schlafen 8	Slfn8	4.2	4.40E-06	0.000411097
1377663_at	Rho family GTPase 3	Rnd3	2.6	4.44E-06	0.000411097
1369029_at	phospholipid scramblase 1	Plscr1	2.8	4.45E-06	0.000411097
1373769_at	phosphoglucomutase 1 (predicted)	Pgm1_predicted	1.8	4.55E-06	0.000411097
1389423_at	Discoidin domain receptor family, member 2	Ddr2	2.3	4.69E-06	0.000418841
1376911_at	Transcribed locus	---	2.9	4.80E-06	0.000427129
1383574_at	mannosidase 1, alpha (predicted)	Man1a_predicted	3.1	4.89E-06	0.000432603
1398871_at	ribosomal protein L17	Rpl17	2.0	4.99E-06	0.000438741
1368321_at	early growth response 1	Egr1	2.4	5.18E-06	0.000450902

1368421_at	protein tyrosine phosphatase, non-receptor type 5	Ptpn5	4.6	5.22E-06	0.000451797
1367939_at	retinol binding protein 1, cellular	Rbp1	4.7	5.30E-06	0.000456509
1373680_at	Transcribed locus	---	3.3	5.44E-06	0.000457863
1373679_at	Transcribed locus	---	1.8	5.47E-06	0.000457863
1369713_at	cholecystokinin B receptor	Cckbr	3.0	5.48E-06	0.000457863
1371988_at	mannosidase 1, alpha (predicted)	Man1a_predicted	2.4	5.48E-06	0.000457863
1374539_at	similar to Probable phospholipid-transporting ATPase VD (ATPVD)	LOC360932	3.4	5.52E-06	0.000458149
1384343_at	---	---	2.0	5.69E-06	0.000468032
1376100_at	tubulin, beta 6	Tubb6	3.2	5.73E-06	0.000468594
1374778_at	---	---	2.5	5.77E-06	0.000469937
1383232_at	---	---	2.0	5.80E-06	0.000470293
1371336_at	hematological and neurological expressed sequence 1	Hn1	2.2	5.85E-06	0.000471627
1373290_at	similar to Enhancer of zeste homolog 2 (ENX-1)	LOC312299	3.9	5.93E-06	0.000475179
1373035_at	---	---	2.3	5.95E-06	0.000475179
1368207_at	FXYD domain-containing ion transport regulator 5	Fxyd5	2.8	6.01E-06	0.000477276
1379967_at	zinc finger protein 367	Zfp367	2.6	6.04E-06	0.000477395
1369182_at	coagulation factor III	F3	3.4	6.10E-06	0.000479668
1382931_at	Transcribed locus	---	3.7	6.26E-06	0.000487648
1391464_at	Transcribed locus	---	4.7	6.44E-06	0.000497832
1372686_at	hypothetical protein LOC682709 /// hypothetical protein LOC689623	LOC682709 /// LOC689623	1.9	6.45E-06	0.000497832
1381311_at	EGF-like module containing, mucin-like, hormone receptor-like sequence 1	Emr1	4.8	6.59E-06	0.000505576
1373025_at	complement component 1, q subcomponent, gamma polypeptide	C1qg	6.0	6.62E-06	0.000505576
1371573_at	ribosomal protein L36a (predicted) /// similar to large subunit ribosomal protein L36a (predicted) /// similar to large subunit ribosomal protein L36a	LOC680395 /// LOC682278 /// LOC682326 /// LOC685642 /// LOC687717 /// LOC691991 /// RGD1566235_predicted /// Rpl36a_predicted	1.9	6.73E-06	0.000505576
1389873_at	PYD and CARD domain containing	Pycard	2.2	6.73E-06	0.000505576
1369131_at	solute carrier family 18 (vesicular monoamine), member 2	Slc18a2	2.7	6.96E-06	0.000519935
1376662_at	Transcribed locus	---	2.0	7.01E-06	0.000519935
1390050_at	similar to Golgi phosphoprotein 2 (Golgi membrane protein GP73)	LOC680692 /// LOC682869	2.3	7.02E-06	0.000519935
1392440_at	Transcribed locus	---	2.9	7.13E-06	0.00052578
1374137_at	Transcribed locus	---	2.6	7.21E-06	0.00052578
1397676_at	---	---	2.9	7.24E-06	0.00052578
1386529_at	Transcribed locus	---	3.9	7.47E-06	0.000536604
1371295_at	ribosomal protein S20	Rps20	1.8	7.70E-06	0.000550312
1370051_at	transglutaminase 1	Tgm1	3.0	7.85E-06	0.000554362
1383355_at	ATP-binding cassette, sub-family A (ABC1), member 1	Abca1	2.7	8.00E-06	0.000556387
1378128_at	Grainyhead-like 1 (Drosophila) (predicted)	Grhl1_predicted	2.9	8.01E-06	0.000556387
1367960_at	ADP-ribosylation factor-like 4A	Arl4a	2.9	8.02E-06	0.000556387
1367847_at	nuclear protein 1	Nupr1	4.6	8.08E-06	0.00055806
1393469_at	---	---	2.3	8.15E-06	0.000561043
1382206_a_at	A kinase (PRKA) anchor protein 2	Akap2	2.5	8.28E-06	0.000567144
1376652_at	complement component 1, q subcomponent, alpha polypeptide	C1qa	6.6	8.31E-06	0.000567144
1398243_at	cysteine and glycine-rich protein 3	Csrp3	16.7	8.93E-06	0.000601954
1367640_at	ribosomal protein S12	Rps12	1.9	9.17E-06	0.000613307
1398407_at	tumor necrosis factor, alpha-induced protein 8 (predicted)	Tnfaip8_predicted	3.2	9.42E-06	0.000627436
1389581_at	similar to RIKEN cDNA 9230117N10	RGD1311155	2.8	9.62E-06	0.000634742
1376319_at	Transcribed locus	---	3.6	9.70E-06	0.000635949
1375661_at	Transcribed locus	---	4.9	9.77E-06	0.0006374

1368295_at	solute carrier organic anion transporter family, member 2b1	Slco2b1	2.4	1.00E-05	0.000644131
1385557_at	---	---	2.3	1.01E-05	0.000645885
1385672_at	Calcium/calmodulin-dependent protein kinase II, delta	Camk2d	2.7	1.02E-05	0.000648767
1372187_at	protein kinase C, nu	Prkcn	2.0	1.03E-05	0.000650391
1389373_at	MAD homolog 1 (Drosophila)	Smad1	2.2	1.03E-05	0.000650391
1368356_a_at	type 1 tumor necrosis factor receptor shedding aminopeptidase regulator	Arts1	1.9	1.05E-05	0.000659603
1382950_at	Transcribed locus	---	5.2	1.05E-05	0.000659603
1367568_a_at	matrix Gla protein	Mgp	3.8	1.07E-05	0.000666305
1388784_at	colony stimulating factor 1 receptor	Csf1r	2.4	1.10E-05	0.000683052
1372031_at	Transcribed locus	---	3.7	1.12E-05	0.000687355
1398727_at	CDNA clone IMAGE:7374368	---	2.7	1.12E-05	0.000688063
1397304_at	interferon gamma induced GTPase	Igtp	4.2	1.13E-05	0.000688063
1388944_at	Transcribed locus	---	3.8	1.18E-05	0.000708701
1370941_at	platelet derived growth factor receptor, alpha polypeptide	Pdgfra	2.6	1.20E-05	0.000713201
1371883_at	monocyte to macrophage differentiation-associated	Mmd	2.5	1.20E-05	0.000712515
1388674_at	cyclin-dependent kinase inhibitor 1A	Cdkn1a	3.6	1.21E-05	0.000715998
1370234_at	fibronectin 1	Fn1	3.4	1.23E-05	0.00072225
1373164_at	Transcribed locus	---	2.3	1.24E-05	0.00072225
1372610_at	procollagen-proline, 2-oxoglutarate 4-dioxygenase (proline 4-hydroxylase), alpha II polypeptide (predicted)	P4ha2_predicted	1.8	1.28E-05	0.000742663
1377146_at	vasoactive intestinal polypeptide	Vip	19.3	1.29E-05	0.000742663
1370613_s_at	UDP glycosyltransferase 1 family, polypeptide A1 /// UDP glycosyltransferase 1 family, polypeptide A6 /// UDP glycosyltransferase 1 family, polypeptide A7 /// UDP glycosyltransferase 1 family, polypeptide A8 /// UDP glycosyltransferase 1 family polypeptide A2 /// UDP glycosyltransferase 1 family polypeptide A3 /// UDP glycosyltransferase 1 family polypeptide A10 /// UDP glycosyltransferase 1 family, polypeptide A5	Ugt1a1 /// Ugt1a10 /// Ugt1a2 /// Ugt1a3 /// Ugt1a5 /// Ugt1a6 /// Ugt1a7 /// Ugt1a8	2.6	1.30E-05	0.000745703
1367791_at	receptor (calcitonin) activity modifying protein 1	Ramp1	3.2	1.31E-05	0.000745703
1388296_at	ribosomal protein S18	Rps18	1.9	1.33E-05	0.000756362
1385440_at	hematopoietic cell signal transducer	Hcst	2.9	1.35E-05	0.000761055
1374113_at	bromodomain adjacent to zinc finger domain, 1A (predicted)	Baz1a_predicted	3.9	1.39E-05	0.000775092
1367712_at	tissue inhibitor of metallopeptidase 1	Timp1	3.2	1.41E-05	0.000776806
1383222_at	FERM-domain-containing protein 163SCII	LOC257646	2.0	1.41E-05	0.000776806
1367874_at	ras homolog gene family, member Q	Rhoq	2.6	1.48E-05	0.000802262
1373829_at	fibroblast growth factor receptor 2	Fgfr2	2.9	1.51E-05	0.000810492
1398882_at	ribosomal protein S5	Rps5	1.7	1.54E-05	0.000820925
1385832_s_at	similar to RIKEN cDNA 1200013B08 (predicted)	RGD1560293_predicted	2.5	1.58E-05	0.000837553
1372642_at	Transcribed locus	---	1.7	1.62E-05	0.000853389
1383486_at	Transcribed locus	---	3.1	1.62E-05	0.000853389
1380318_at	Transcribed locus, strongly similar to XP_579933.1 hypothetical protein XP_579933 [Rattus norvegicus]	---	2.6	1.64E-05	0.000857179
1371015_at	myxovirus (influenza virus) resistance 1	Mx1	8.4	1.67E-05	0.000867383
1388884_at	similar to RIKEN cDNA 1810022C23	RGD1310224	1.9	1.68E-05	0.000867383
1390866_at	Transcribed locus	---	3.7	1.68E-05	0.000867383
1393329_at	Inducible T-cell co-stimulator	Icos	1.7	1.71E-05	0.000873114
1367597_at	ribosomal protein S8	Rps8	1.8	1.72E-05	0.000873114
1373485_at	Similar to ring finger protein 122 homolog (predicted)	RGD1561238_predicted	2.1	1.72E-05	0.000873114

1378032_at	nuclear factor of kappa light polypeptide gene enhancer in B-cells inhibitor, zeta (predicted)	Nfkbiz_predicted	2.2	1.74E-05	0.000878626
1384933_at	Transcribed locus	---	2.2	1.79E-05	0.00089819
1377702_at	similar to purinergic receptor P2Y, G-protein coupled, 5	LOC684663	2.1	1.82E-05	0.000905998
1369202_at	myxovirus (influenza virus) resistance 2	Mx2	7.5	1.83E-05	0.000907641
1389047_at	Similar to Bcl2-associated athanogene 2	LOC690038	1.9	1.83E-05	0.000907641
1371344_at	ribosomal protein L27a (predicted)	Rpl27a_predicted	1.9	1.84E-05	0.000907641
1383401_at	similar to Testis derived transcript	LOC500040	2.0	1.87E-05	0.000918829
1394940_at	similar to hypothetical protein FLJ20037 (predicted)	RGD1311381_predicted	5.3	1.97E-05	0.000951114
1376750_at	Transcribed locus	---	6.9	2.00E-05	0.000961769
1398347_at	Axl receptor tyrosine kinase /// similar to AXL receptor tyrosine kinase	Axl /// LOC687188	2.2	2.02E-05	0.000968411
1389351_at	leucine rich repeat (in FLII) interacting protein 1	Lrrfip1	2.5	2.03E-05	0.000968411
1368482_at	B-cell leukemia/lymphoma 2 related protein A1	Bcl2a1	4.2	2.05E-05	0.000972323
1376805_at	Transcribed locus	---	2.2	2.06E-05	0.000972323
1387074_at	regulator of G-protein signaling 2	Rgs2	1.8	2.12E-05	0.000988764
1396144_at	similar to Traf2 and NCK interacting kinase, splice variant 4 (predicted)	RGD1561817_predicted	3.3	2.15E-05	0.000997067
1376579_at	leucine aminopeptidase 3	Lap3	1.6	2.17E-05	0.001002135
1390798_at	protein tyrosine phosphatase, receptor type, C	Ptprc	4.3	2.17E-05	0.001002135
1390738_at	bone marrow stromal cell antigen 2	Bst2	3.6	2.19E-05	0.00101105
1383833_at	hypothetical protein LOC500991	LOC500991	2.0	2.21E-05	0.001015979
1389966_at	procollagen, type VI, alpha 3 (predicted)	Col6a3_predicted	2.8	2.22E-05	0.001017827
1384548_at	ribosomal protein L32	Rpl32	1.9	2.23E-05	0.001019999
1367610_at	ribosomal protein L19	Rpl19	1.7	2.24E-05	0.00102046
1381985_at	---	---	1.9	2.25E-05	0.001023349
1371302_at	similar to Small EDRK-rich factor 2 (4F5rel)	LOC502663	1.7	2.27E-05	0.001028334
1377766_at	similar to erythroid differentiation-related factor 1 (predicted)	RGD1306820_predicted	1.9	2.30E-05	0.001040935
1390096_at	SH3-domain GRB2-like B1 (endophilin)	Sh3glb1	1.7	2.33E-05	0.001044751
1377713_at	checkpoint suppressor 1 (predicted)	Ches1_predicted	1.7	2.35E-05	0.001044751
1378311_at	---	---	3.7	2.36E-05	0.001044751
1390687_at	pleckstrin	Plek	4.8	2.36E-05	0.001044751
1374734_at	hypothetical protein LOC680254 /// hypothetical protein LOC686282	LOC680254 /// LOC686282	2.0	2.37E-05	0.001044751
1377831_at	monoamine oxidase A	Maoa	2.0	2.37E-05	0.001044751
1367717_at	ribosomal protein S27	Rps27	1.7	2.39E-05	0.001049726
1389496_at	A kinase (PRKA) anchor protein 7	Akap7	2.5	2.44E-05	0.001060703
1392148_at	Similar to H43E16.1	LOC689994	1.7	2.46E-05	0.001064804
1370408_at	putative small membrane protein NID67	Nid67	1.7	2.54E-05	0.001089032
1378009_at	Transcribed locus	---	2.3	2.57E-05	0.001093364
1389228_at	similar to RIKEN cDNA 2010309E21 (predicted)	RGD1304825_predicted	1.7	2.57E-05	0.001093364
1392578_at	Matrix Gla protein	Mgp	3.6	2.58E-05	0.001094871
1380811_at	Transcribed locus	---	1.6	2.66E-05	0.001119838
1372256_at	similar to Cysteine-rich protein 1 (Cysteine-rich intestinal protein) (CRIP)	LOC686293 /// LOC691657	2.0	2.67E-05	0.001119838
1367850_at	Fc receptor, IgG, low affinity III	Fcgr3	5.8	2.73E-05	0.001133504
1373052_at	phosducin-like 3	Pdcl3	1.9	2.75E-05	0.001133504
1382108_at	Transcribed locus	---	3.9	2.76E-05	0.001133504
1376151_a_at	---	---	4.0	2.86E-05	0.001171025
1383946_at	claudin 1	Cldn1	5.6	2.86E-05	0.001171025
1371057_at	gamma-aminobutyric acid (GABA-A) receptor, subunit alpha 5	Gabra5	2.8	2.88E-05	0.001171025
1389368_at	Cnksr family member 3	Cnksr3	2.0	2.92E-05	0.001176405
1368464_at	macrophage galactose N-acetyl-galactosamine specific lectin 1	Mgl1	4.0	2.95E-05	0.001181019
1398775_at	ribosomal protein S15a	Rps15a	1.7	2.97E-05	0.001184622
1373575_at	similar to NADH dehydrogenase (ubiquinone) Fe-S protein 2	LOC498279	5.3	3.04E-05	0.001199774
1387891_at	peroxiredoxin 4	Prdx4	1.7	3.04E-05	0.001199774

1392557_at	Bicaudal C homolog 1 (Drosophila) (predicted)	Bicc1_predicted	2.4	3.07E-05	0.001207012
1388138_at	thrombospondin 4	Thbs4	3.8	3.08E-05	0.001210702
1372064_at	similar to chemokine (C-X-C motif) ligand 16	Cxcl16	2.6	3.17E-05	0.001234846
1378413_at	Transcribed locus	---	3.2	3.18E-05	0.001234846
1397808_at	Cathepsin C	Ctsc	3.0	3.23E-05	0.00124752
1387101_at	acyl-CoA synthetase long-chain family member 4	Acsl4	2.1	3.25E-05	0.00124936
1367938_at	UDP-glucose dehydrogenase	Ugdh	1.9	3.26E-05	0.00124936
1372685_at	cyclin-dependent kinase inhibitor 3 (predicted)	Cdkn3_predicted	3.6	3.26E-05	0.00124936
1373657_at	solute carrier family 31, member 2	Slc31a2	2.0	3.29E-05	0.001254225
1381341_at	---	---	3.7	3.31E-05	0.001259286
1372930_at	SP110 nuclear body protein	Sp110	3.7	3.38E-05	0.001284507
1398481_at	Transcribed locus	---	2.0	3.39E-05	0.001284592
1387032_at	cholecystokinin	Cck	1.7	3.42E-05	0.001293545
1377779_at	phosducin-like 3	Pdcl3	1.9	3.44E-05	0.001297397
1377334_at	RT1 class II, locus Ba	RT1-Ba	3.9	3.46E-05	0.001302935
1367912_at	latent transforming growth factor beta binding protein 1	Ltbp1	3.0	3.48E-05	0.001302935
1376645_at	Similar to RIKEN cDNA 6330406I15 (predicted)	RGD1307396_predicted	2.9	3.50E-05	0.001303798
1376708_at	Hypothetical protein LOC680422	LOC680422	2.7	3.50E-05	0.001303798
1367946_at	PDZ and LIM domain 1 (elfin)	Pdlim1	2.1	3.56E-05	0.001321305
1368742_at	complement component 5, receptor 1	C5r1	2.0	3.58E-05	0.001325845
1388694_at	---	---	4.0	3.59E-05	0.001325845
1380110_at	---	---	2.0	3.64E-05	0.001336019
1387893_at	complement component 1, s subcomponent /// similar to complement component 1, s subcomponent (predicted)	C1s /// RGD1561715_predicted	3.8	3.64E-05	0.001336019
1392515_at	Ly49 inhibitory receptor 9 /// immunoreceptor Ly49si1 /// immunoreceptor Ly49si2 /// immunoreceptor Ly49si3 /// Ly49 inhibitory receptor 5 /// similar to immunoreceptor Ly49si3 (predicted) /// similar to immunoreceptor Ly49si1 /// similar to immunoreceptor Ly49si3	LOC502907 /// LOC684013 /// LOC684059 /// LOC684068 /// LOC690045 /// LOC690097 /// Ly49i5 /// Ly49i9 /// Ly49si1 /// Ly49si2 /// Ly49si3 /// RGD1561306_predicted /// RGD1563110_predicted	6.8	3.65E-05	0.001336019
1368834_at	calcium/calmodulin-dependent protein kinase II, delta	Camk2d	1.8	3.68E-05	0.001345088
1395184_at	---	---	3.7	3.70E-05	0.001347652
1383659_a_at	---	---	2.5	3.77E-05	0.001364509
1392092_at	RAB33A, member of RAS oncogene family (predicted)	Rab33a_predicted	2.1	3.78E-05	0.001365013
1368488_at	nuclear factor, interleukin 3 regulated	Nfil3	2.0	3.79E-05	0.001366103
1369973_at	xanthine dehydrogenase	Xdh	2.4	3.80E-05	0.00136867
1385029_at	Transcribed locus	---	2.5	3.86E-05	0.001380567
1371951_at	---	---	2.5	3.89E-05	0.001389675
1367930_at	growth associated protein 43	Gap43	1.9	3.94E-05	0.001398645
1374422_at	similar to Erbb2 interacting protein isoform 2 (predicted)	RGD1562952_predicted	1.9	4.02E-05	0.001421549
1388372_at	ribosomal protein L35	Rpl35	1.8	4.03E-05	0.001422271
1367954_at	glial cell line derived neurotrophic factor family receptor alpha 1	Gfra1	2.5	4.06E-05	0.001431682
1383269_at	ring finger protein 2	Rnf2	1.8	4.12E-05	0.001447847
1379477_at	Similar to Programmed cell death 6 interacting protein (ALG-2 interacting protein X) (predicted)	RGD1561176_predicted	2.6	4.20E-05	0.001474299
1374615_at	---	---	2.0	4.29E-05	0.001502529
1397540_at	Transcribed locus	---	3.1	4.36E-05	0.001516999
1387134_at	schlafen 3	Slfn3	9.3	4.37E-05	0.001516999
1371037_at	protein S (alpha)	Pros1	1.9	4.42E-05	0.001526634

1379043_at	Transcribed locus	---	1.7	4.48E-05	0.001543115
1389006_at	Transcribed locus	---	8.5	4.50E-05	0.001543776
1389443_at	---	---	2.1	4.52E-05	0.001549915
1388303_at	ribosomal protein L26	Rpl26	2.2	4.57E-05	0.001557006
1391660_at	Transcribed locus	---	2.8	4.71E-05	0.0015857
1374941_at	Transcribed locus	---	2.5	4.75E-05	0.001594456
1371002_at	programmed cell death 2	Pdcd2	2.3	4.77E-05	0.001598008
1393213_at	cyclin L2	Ccnl2	1.9	4.78E-05	0.001598008
1382739_at	G protein-coupled receptor 19	Gpr19	1.8	4.82E-05	0.001602731
1367614_at	annexin A1	Anxa1	2.0	5.07E-05	0.001662315
1373504_at	GLI pathogenesis-related 1 (glioma)	Glipr1	2.6	5.07E-05	0.001662315
1397004_at	Nuclear receptor subfamily 3, group C, member 1	Nr3c1	2.0	5.18E-05	0.001689257
1393111_at	---	---	3.1	5.29E-05	0.001715654
1376973_at	syndecan binding protein (syntenin) 2	Sdcbp2	2.3	5.33E-05	0.001724485
1376996_at	Transcribed locus	---	1.7	5.34E-05	0.001724485
1370418_s_at	brain and kidney protein	Bk	2.3	5.35E-05	0.001724485
1369268_at	activating transcription factor 3	Atf3	8.4	5.36E-05	0.001724485
1368224_at	serine (or cysteine) peptidase inhibitor, clade A, member 3N	Serpina3n	6.8	5.45E-05	0.001745623
1368106_at	polo-like kinase 2 (Drosophila)	Plk2	1.8	5.48E-05	0.001745623
1371308_at	ribosomal protein S4, X-linked	Rps4x	1.6	5.48E-05	0.001745623
1388879_at	similar to ABI gene family, member 3 (NESH) binding protein (predicted)	RGD1562717_predicted	2.6	5.48E-05	0.001745623
1391719_at	---	---	1.8	5.48E-05	0.001745623
1383708_at	similar to integrin, beta-like 1	LOC498564	3.9	5.51E-05	0.001745623
1373838_at	fucosyltransferase 4	Fut4	2.4	5.53E-05	0.001745623
1382442_at	similar to Septin-6	LOC682750	1.9	5.56E-05	0.001748159
1377674_at	similar to PHF21A protein (predicted)	RGD1560612_predicted	1.7	5.59E-05	0.001753636
1373424_at	DiGeorge syndrome critical region gene 6 (predicted)	Dgcr6_predicted	1.7	5.67E-05	0.001767498
1368211_at	ribosomal protein S14	Rps14	1.8	5.72E-05	0.001769703
1388700_at	CDNA clone IMAGE:7317367	---	1.8	5.73E-05	0.001769703
1374643_at	similar to FAT tumor suppressor homolog 4 (predicted)	RGD1564291_predicted	1.9	5.85E-05	0.001795884
1376763_at	Transcribed locus	---	2.6	5.85E-05	0.001795884
1391812_at	Similar to RIKEN cDNA E330026B02 (predicted)	RGD1309172_predicted	4.1	5.92E-05	0.001809363
1388986_at	Transcribed locus	---	1.7	5.98E-05	0.00181593
1393639_at	myosin X (predicted)	Myo10_predicted	2.1	6.12E-05	0.001854453
1392655_at	Transcribed locus	---	3.3	6.18E-05	0.001860461
1373499_at	growth arrest specific 5	Gas5	2.6	6.21E-05	0.001863871
1367516_at	distrobrevin binding protein 1	Dtnbp1	2.4	6.36E-05	0.001899804
1383986_at	tropomyosin 3, gamma	Tpm3	1.6	6.45E-05	0.001921205
1373106_at	zinc finger protein 36, C3H type-like 2	Zfp36l2	2.5	6.46E-05	0.001921205
1379835_at	Transcribed locus	---	1.8	6.70E-05	0.001967953
1376664_at	liver regeneration-related protein	LOC246187	3.3	6.75E-05	0.001978936
1376649_at	Transcribed locus	---	2.2	6.81E-05	0.001988424
1373823_at	similar to Cyclin-dependent kinases regulatory subunit 2 (CKS-2) (predicted)	RGD1562047_predicted	3.3	6.82E-05	0.001988424
1382492_a_at	hydroxysteroid (17-beta) dehydrogenase 8	Hsd17b8	2.7	6.82E-05	0.001988424
1367940_at	chemokine orphan receptor 1	Cmkor1	2.6	6.88E-05	0.002000394
1375181_at	similar to 60S ribosomal protein L12 (predicted) /// similar to 60S ribosomal protein L12 /// hypothetical LOC500136 /// similar to similar to 60S ribosomal protein L12	LOC499782 /// LOC500136 /// LOC679192 /// LOC685320 /// LOC688511 /// RGD1564883_predicted	1.5	6.94E-05	0.002010193
1367973_at	chemokine (C-C motif) ligand 2	Ccl2	2.4	6.95E-05	0.002010193
1371940_at	similar to microfilament and actin filament cross-linker protein isoform b	LOC362587	1.6	6.97E-05	0.002010193
1370194_at	synaptosomal-associated protein 23	Snap23	1.8	6.99E-05	0.002010193
1368361_a_at	protein tyrosine phosphatase, non-receptor type 2	Ptpn2	2.9	7.00E-05	0.002010193
1383910_at	Transcribed locus	---	2.2	7.01E-05	0.002010193
1371307_at	ribosomal protein, large, P1	Rplp1	1.6	7.06E-05	0.002016211

1377950_at	similar to interferon-inducible GTPase	RGD1309362	9.3	7.10E-05	0.002025095
1388754_at	---	---	2.6	7.20E-05	0.002046425
1380219_at	---	---	1.6	7.30E-05	0.002069202
1370442_at	thymosin beta-like protein 1	Tmsbl1	1.8	7.33E-05	0.002071863
1380297_at	---	---	1.7	7.42E-05	0.002090952
1390024_at	---	---	2.6	7.56E-05	0.002123916
1378753_at	occludin	Ocln	3.4	7.71E-05	0.002157244
1388449_at	eukaryotic translation elongation factor 1 beta 2 (predicted)	Eef1b2_predicted	1.8	7.90E-05	0.00220097
1393499_at	phosphatidylinositol 3-kinase, catalytic, alpha polypeptide	Pik3ca	2.5	7.97E-05	0.002213494
1399062_at	CDNA clone IMAGE:7317308	---	2.0	8.01E-05	0.002215606
1393144_at	N-myc (and STAT) interactor	Nmi	2.3	8.08E-05	0.002229334
1377198_at	Transcribed locus	---	2.2	8.19E-05	0.002251458
1380665_at	CGI-58-like protein	LOC316122	1.6	8.32E-05	0.002267697
1367998_at	secretory leukocyte peptidase inhibitor /// similar to secretory leukocyte protease inhibitor (predicted)	RGD1563818_predicted /// Slpi	8.3	8.34E-05	0.002267697
1374029_at	---	---	1.9	8.34E-05	0.002267697
1383294_at	prodynorphin	Pdyn	2.7	8.52E-05	0.002298812
1383658_at	lysosomal-associated protein transmembrane 5	Laptm5	2.2	8.52E-05	0.002298812
1373421_at	TG interacting factor	Tgif	2.1	8.56E-05	0.002298812
1382452_at	serum deprivation response protein	Sdpr	2.2	8.57E-05	0.002298812
1377064_at	dual specificity phosphatase 6	Dusp6	2.4	8.62E-05	0.002306719
1367679_at	CD74 antigen (invariant polypeptide of major histocompatibility complex, class II antigen-associated)	Cd74	6.0	8.64E-05	0.002306719
1391341_at	ADP-ribosylation factor-like 12 (predicted)	Arl12_predicted	1.8	8.64E-05	0.002306719
1374544_at	hypothetical protein LOC679150 /// hypothetical protein LOC690899	LOC679150 /// MGC112715	1.8	8.69E-05	0.002315223
1372204_at	---	---	1.7	8.78E-05	0.002332378
1389295_at	olfactomedin-like 2B (predicted)	Olfml2b_predicted	1.9	8.84E-05	0.002342208
1398324_at	similar to 60S ribosomal protein L18a	MGC72957	1.6	8.84E-05	0.002342208
1376700_at	Clone ZX36 unknown mRNA	---	2.3	8.90E-05	0.002348947
1368754_at	pyrimidinergic receptor P2Y, G-protein coupled, 6	P2ry6	1.9	9.02E-05	0.002362838
1387887_at	ribosomal protein L14	Rpl14	1.6	9.04E-05	0.002362838
1375011_at	Transcribed locus	---	1.8	9.15E-05	0.002377389
1373368_at	similar to procollagen C-endopeptidase enhancer 2	LOC684050	2.0	9.16E-05	0.002377389
1379631_at	colony stimulating factor 1 (macrophage)	Csf1	2.3	9.16E-05	0.002377389
1381145_at	Transcribed locus	---	3.9	9.20E-05	0.002378591
1387186_at	RAB9, member RAS oncogene family	Rab9	1.9	9.29E-05	0.00239305
1372112_at	similar to 9230105E10Rik protein	RGD1304579	2.3	9.30E-05	0.00239305
1373897_at	---	---	3.7	9.32E-05	0.002394446
1368202_a_at	disabled homolog 2 (Drosophila)	Dab2	2.7	9.35E-05	0.002398608
1387221_at	GTP cyclohydrolase 1	Gch	5.4	9.54E-05	0.002436801
1390909_at	Transcribed locus	---	2.5	9.57E-05	0.002439176
1392274_at	Platelet-derived growth factor, C polypeptide	Pdgfc	2.3	9.65E-05	0.002451996
1397173_at	similar to Serine/threonine-protein kinase WNK3 (Protein kinase, lysine-deficient 3) (predicted)	RGD1563131_predicted	1.9	9.66E-05	0.002451996
1398315_at	ribosomal protein L15	Rpl15	1.6	9.80E-05	0.002477476
1388673_at	lymphocyte specific 1	Lsp1	1.8	9.91E-05	0.002488416
1388662_at	Transcribed locus	---	1.7	9.94E-05	0.002488416
1370678_s_at	monoamine oxidase A	Maoa	2.1	0.000100263	0.002498677
1386879_at	lectin, galactose binding, soluble 3	Lgals3	1.7	0.000100683	0.002505356
1382043_at	unc-93 homolog B1 (C. elegans)	Unc93b1	1.8	0.000101854	0.002519242
1370281_at	fatty acid binding protein 5, epidermal	Fabp5	1.6	0.00010355	0.002549682
1368728_at	purinergic receptor P2Y, G-protein coupled 12	P2ry12	1.8	0.000106623	0.002595861
1389408_at	ribonucleotide reductase M2	Rrm2	4.4	0.000106688	0.002595861
1393492_at	Transcribed locus	---	2.2	0.000107618	0.002613317
1367596_at	ribosomal protein S26	Rps26	1.7	0.000107723	0.002613317
1398854_at	ribosomal protein L24	Rpl24	1.9	0.000107896	0.002613647

1378654_at	Transcribed locus	---	1.7	0.000108462	0.002623515
1389143_at	phosphatidylinositol 3-kinase, catalytic, alpha polypeptide	Pik3ca	1.9	0.00010929	0.002633336
1370892_at	complement component 4a /// complement component 4, gene 2	C4-2 /// C4a	4.3	0.000109509	0.002633336
1384093_at	similar to Complement C1q-like protein 3 precursor (Gliacolin)	LOC684921	3.3	0.000110084	0.00263908
1374794_at	kinesin family member 15	Kif15	3.3	0.00011055	0.00263908
1383146_at	similar to neurobeachin (predicted)	RGD1562629_predicted	1.7	0.000110792	0.002641016
1369959_at	zinc finger protein 36, C3H type-like 1	Zfp36l1	2.4	0.000111825	0.002656412
1372564_at	v-ets erythroblastosis virus E26 oncogene homolog 2 (avian)	Ets2	1.7	0.000112222	0.002656412
1388519_at	Sec61 beta subunit (predicted)	Sec61b_predicted	1.7	0.000112345	0.002656412
1387169_at	transducin-like enhancer of split 3, E(spl) homolog (Drosophila)	Tle3	2.1	0.00011273	0.002656412
1382749_at	RNA binding motif protein 5	Rbm5	1.7	0.000113838	0.00267869
1367645_at	ribosomal protein S17	Rps17	1.6	0.000114446	0.002682757
1370882_at	major histocompatibility complex, class II, DM beta	Hla-dmb	3.2	0.000115789	0.002709097
1384195_at	similar to transmembrane protein 45a	LOC680866 /// LOC682422	2.7	0.000116772	0.002718281
1393653_at	similar to putative protein product of HMFN2073 (predicted)	RGD1560766_predicted	1.9	0.000118195	0.002735019
1367916_at	phospholipase C, delta 1	Plcd1	1.8	0.000118511	0.002737737
1384707_at	Transcribed locus	---	3.3	0.000119352	0.002745607
1379397_at	RAR-related orphan receptor alpha (predicted)	Rora_predicted	1.6	0.000123413	0.002823226
1396835_at	Transcribed locus	---	2.0	0.000124351	0.002829627
1373433_at	nucleosome binding protein 1 (predicted) /// similar to Nucleosome binding protein 1 (Nucleosome binding protein 45) (NBP-45) (GARP45 protein)	LOC680182 /// LOC681284 /// LOC682212 /// Nsbp1_predicted	2.4	0.000125096	0.002838054
1392238_at	Transcribed locus	---	2.2	0.000125167	0.002838054
1374650_at	neural precursor cell expressed, developmentally down-regulated gene 9	Nedd9	1.9	0.000125269	0.002838054
1390042_at	similar to 1110007F12Rik protein	MGC109491	3.1	0.000129218	0.002887736
1393460_at	leucine rich repeat containing 33	Lrrc33	2.0	0.000130594	0.002910576
1368073_at	interferon regulatory factor 1	Irf1	2.2	0.000133201	0.002949341
1376575_at	Transcribed locus	---	2.0	0.00013323	0.002949341
1383238_at	queuine tRNA-ribosyltransferase 1	Qtrt1	1.8	0.000133823	0.002956634
1374065_at	Met proto-oncogene	Met	2.5	0.000134497	0.002964903
1385572_at	Transcribed locus	---	2.2	0.000134654	0.002964903
1374691_at	sulfotransferase family 5A, member 1 (predicted)	Sult5a1_predicted	2.1	0.000135358	0.002974878
1388900_at	RGD1566118 (predicted)	RGD1566118_predicted	1.9	0.000135468	0.002974878
1397692_at	cytotoxic granule-associated RNA binding protein 1	Tia1	2.1	0.000136249	0.002984051
1387313_at	myocilin	Myoc	2.5	0.000136637	0.002988566
1386912_at	procollagen C-endopeptidase enhancer protein	Pcolce	1.8	0.000137678	0.003000657
1398809_at	nuclear distribution gene E homolog 1 (A nidulans)	Nde1	1.5	0.000137737	0.003000657
1370075_at	dihydrofolate reductase	Dhfr	2.1	0.000139177	0.003024029
1367671_at	proliferating cell nuclear antigen	Pcna	1.6	0.000140339	0.003041241
1372734_at	small cell adhesion glycoprotein	Smagp	3.7	0.000141507	0.00304339
1378440_at	Transcribed locus	---	2.2	0.000141625	0.00304339
1373992_at	similar to interferon-inducible GTPase /// similar to MGC108823 protein (predicted)	MGC108823 /// RGD1559715_predicted	10.2	0.000141777	0.00304339
1376673_at	transcription factor 8	Tcf8	1.6	0.000141894	0.00304339
1373072_at	Transcribed locus	---	1.5	0.000141919	0.00304339
1372167_at	similar to small nuclear ribonucleoprotein polypeptide G	LOC681031 /// LOC686174 /// LOC687679	2.0	0.000142106	0.003043441
1373754_at	Similar to Golgi phosphoprotein 2 (Golgi membrane protein GP73)	LOC680692	2.6	0.000144114	0.003078434

1369649_at	calcium channel, voltage-dependent, alpha2/delta subunit 1	Cacna2d1	2.7	0.000144569	0.003084136
1387276_at	activity and neurotransmitter-induced early gene protein 4 (ania-4)	Ania4	2.0	0.000145138	0.003092277
1379742_at	---	---	2.6	0.000147111	0.003110839
1375010_at	CD68 antigen	Cd68	6.4	0.000147208	0.003110839
1375640_at	FK506 binding protein 9	Fkbp9	1.7	0.000147473	0.003110839
1368947_at	growth arrest and DNA-damage-inducible 45 alpha	Gadd45a	2.8	0.000148056	0.003118079
1372282_at	similar to RIKEN cDNA 2310016C16 (predicted)	RGD1307506_predicted	1.9	0.000149963	0.003142146
1379369_at	prickle-like 1 (Drosophila)	Prickle1	2.0	0.000150363	0.0031449
1372404_at	RAS-related C3 botulinum substrate 2	Rac2	2.9	0.000150659	0.0031449
1383179_at	Transcribed locus	---	2.2	0.000150668	0.0031449
1368829_at	fibrillin 1	Fbn1	2.9	0.000151764	0.003159775
1368323_at	tissue factor pathway inhibitor	Tfpi	1.9	0.000153399	0.003185736
1373975_at	similar to indolethylamine N-methyltransferase	LOC368066	2.4	0.000156344	0.003226539
1373386_at	gap junction membrane channel protein beta 2	Gjb2	4.4	0.000159726	0.003272732
1372500_at	tropomodulin 3	Tmod3	2.2	0.000160073	0.003274722
1381850_at	protein phosphatase 1, regulatory (inhibitor) subunit 12A	Ppp1r12a	5.6	0.000163329	0.00333305
1392579_at	similar to 5830411E10Rik protein	RGD1306658	1.8	0.000165169	0.003366411
1388142_at	chondroitin sulfate proteoglycan 2	Cspg2	3.7	0.000167016	0.003395647
1367606_at	ribosomal protein S3a	Rps3a	1.7	0.000167236	0.00339593
1388711_at	interleukin 13 receptor, alpha 1	Il13ra1	2.2	0.000167782	0.003398625
1398774_at	ribosomal protein L30	Rpl30	1.6	0.000168799	0.003406638
1371475_at	ribonuclease, RNase A family 4	Rnase4	2.6	0.000172093	0.003458009
1373065_at	protein tyrosine phosphatase, non-receptor type 18	Ptpn18	2.1	0.000172186	0.003458009
1393249_at	Transcribed locus	---	2.5	0.000173496	0.003469941
1371349_at	procollagen, type VI, alpha 1 (predicted)	Col6a1_predicted	2.6	0.000173624	0.003469941
1399158_a_at	nucleophosmin 1	Npm1	1.6	0.000174658	0.003482143
1371998_at	MOB1, Mps One Binder kinase activator-like 1B (yeast)	Mobk1b	1.6	0.000176237	0.003500911
1368474_at	vascular cell adhesion molecule 1	Vcam1	2.1	0.00017895	0.003541958
1372725_at	similar to phospholipid scramblase 2	LOC315883	3.0	0.000179773	0.003553526
1367902_at	guanine nucleotide binding protein (G protein), gamma 11	Gng11	2.5	0.000180471	0.003553526
1392842_at	Transcribed locus	---	3.9	0.000180533	0.003553526
1388340_at	NS5A (hepatitis C virus) transactivated protein 9	Ns5atp9	3.7	0.000181832	0.003571466
1389170_at	caspase 7	Casp7	3.1	0.000182922	0.003581795
1372042_at	CKLF-like MARVEL transmembrane domain containing 3 (predicted)	Cmtm3_predicted	2.7	0.000183485	0.00358853
1376685_at	Transcribed locus	---	1.8	0.000184682	0.003599975
1377092_at	Transcribed locus	---	3.1	0.000185199	0.003599975
1375363_at	Transcribed locus	---	1.5	0.000185724	0.003599975
1371447_at	placenta-specific 8 (predicted)	Plac8_predicted	4.0	0.000185821	0.003599975
1388114_at	myosin light chain, regulatory B	Mrlcb	2.2	0.000186724	0.003604234
1370928_at	LPS-induced TN factor	Litaf	2.2	0.000186933	0.003604234
1379285_at	receptor transporter protein 4 (predicted)	Rtp4_predicted	6.3	0.000186994	0.003604234
1367634_at	ribosomal protein L31	Rpl31	1.6	0.000189275	0.003625074
1371644_at	protein tyrosine kinase 9	Ptk9	1.6	0.00018932	0.003625074
1387113_at	C-terminal binding protein 2	Ctbp2	3.1	0.000190005	0.003633959
1392965_a_at	SPARC related modular calcium binding 2 (predicted)	Smoc2_predicted	2.7	0.000190244	0.003634299
1389796_at	Transcribed locus	---	1.8	0.000192694	0.003662697
1372585_at	RGD1566254 (predicted)	RGD1566254_predicted	2.7	0.000197451	0.003711618
1395755_at	---	---	2.0	0.000199402	0.003739752
1383169_at	Transcribed locus	---	1.6	0.000201783	0.003775794
1393122_at	---	---	1.7	0.000205147	0.003811252
1368171_at	lysyl oxidase	Lox	3.2	0.00020542	0.003811252
1376606_a_at	similar to e(y)2 protein	LOC682575 /// LOC685258	1.8	0.00020555	0.003811252
1384087_at	---	---	1.8	0.000205693	0.003811252

1393956_at	serine/threonine kinase 17b (apoptosis-inducing)	Stk17b	1.5	0.000206115	0.003811252
1371691_at	retinoic acid receptor responder (tazarotene induced) 2	Rarres2	1.7	0.000206227	0.003811252
1368144_at	regulator of G-protein signaling 2	Rgs2	1.9	0.000206546	0.003812865
1382727_at	---	---	1.7	0.000207035	0.003817618
1389554_at	Transcribed locus	---	2.0	0.000207633	0.003819078
1377243_at	---	---	2.1	0.000207756	0.003819078
1385638_at	Transcribed locus	---	2.0	0.000207811	0.003819078
1377572_at	---	---	1.9	0.00021153	0.003883083
1373846_at	similar to RIKEN cDNA 5730434I03 gene (predicted) /// similar to basic transcription factor 3-like 4	LOC683767 /// RGD1311774_predicted	1.8	0.000213548	0.003915763
1379295_at	similar to guanine nucleotide binding protein (G protein), gamma transducing activity polypeptide 2	LOC688154 /// LOC690825	1.9	0.000214662	0.003931797
1379345_at	procollagen, type XV	Col15a1	2.4	0.000216915	0.003964248
1392452_at	similar to PHD finger protein 14 isoform 1 (predicted)	RGD1563764_predicted	2.5	0.000218213	0.003983538
1394490_at	ATP-binding cassette, sub-family A (ABC1), member 1	Abca1	3.3	0.000221753	0.004025815
1387144_at	integrin alpha 1	Itga1	1.9	0.000223388	0.004048591
1384254_at	similar to OTU domain containing 1 (predicted)	RGD1563344_predicted	2.6	0.000224376	0.004056321
1387817_at	neuron specific gene family member 1	Nsg1	2.3	0.00022442	0.004056321
1383912_at	similar to Protein C6orf115	LOC684848 /// LOC685045	1.8	0.000225629	0.004069235
1389832_at	glutathione S-transferase omega 1	Gsto1	1.5	0.000226808	0.004084059
1372343_at	exosome component 8 (predicted)	Exosc8_predicted	1.8	0.000226947	0.004084059
1374927_at	Transcribed locus	---	2.2	0.000236328	0.004180784
1382778_at	Dual specificity phosphatase 6	Dusp6	2.6	0.000236874	0.004180784
1393795_at	zinc finger homeobox 1b	Zfhx1b	3.4	0.000240519	0.004235611
1374854_at	similar to Pin2-interacting protein X1 (predicted)	RGD1566025_predicted	1.9	0.000242703	0.004260391
1368419_at	ceruloplasmin	Cp	3.0	0.000243773	0.004272382
1386882_at	t-complex testis expressed 1	Tctex1	2.0	0.000244172	0.004272488
1393796_at	centrosomal protein 152 (predicted)	Cep152_predicted	1.6	0.00024513	0.004283997
1377221_at	Transcribed locus	---	1.6	0.000245351	0.004283997
1373439_at	Transcribed locus	---	1.8	0.000245814	0.00428753
1373610_at	SEC24 related gene family, member D (S. cerevisiae) (predicted)	Sec24d_predicted	2.0	0.00024643	0.004293734
1389732_at	Similar to CG4025-PA	LOC679937	3.1	0.00024695	0.004298236
1367786_at	proteosome (prosome, macropain) subunit, beta type 8	Psmb8	3.2	0.000248111	0.004309324
1388455_at	guanine nucleotide binding protein (G protein), gamma 10	Gng10	1.5	0.000250256	0.004332845
1388431_at	synovial sarcoma translocation, Chromosome 18	Ss18	1.7	0.000251221	0.00434498
1383936_at	Elastin microfibril interfacer 2 (predicted)	Emilin2_predicted	2.1	0.000255821	0.004410634
1389857_at	WW domain binding protein 5 (predicted)	Wbp5_predicted	2.1	0.000256138	0.004411472
1383501_at	Transcribed locus	---	2.4	0.000256768	0.004415913
1368990_at	cytochrome P450, family 1, subfamily b, polypeptide 1	Cyp1b1	2.0	0.000256933	0.004415913
1369788_s_at	Jun oncogene	Jun	2.7	0.000259589	0.004452263
1398907_at	ORM1-like 2 (S. cerevisiae) (predicted)	Ormdl2_predicted	1.8	0.000260118	0.004454917
1377114_at	---	---	3.3	0.000261576	0.00446308
1367925_at	major vault protein	Mvp	1.7	0.000262467	0.004468034
1390373_at	Transcribed locus	---	1.9	0.000263307	0.004473593
1379739_at	Transcribed locus	---	1.9	0.000263553	0.004473593
1391094_at	Transcribed locus	---	1.9	0.000264045	0.00447733
1378551_at	cytochrome P450, family 20, subfamily A, polypeptide 1	Cyp20a1	1.7	0.000264455	0.004479667
1374718_at	Transcribed locus	---	2.4	0.000268873	0.004540484
1367749_at	lumican	Lum	2.7	0.000269775	0.004541834
1369720_at	myosin Ib	Myo1b	2.0	0.000269782	0.004541834
1369947_at	cathepsin K	Ctsk	3.0	0.000270665	0.004549291
1372365_at	Ras and Rab interactor 2 (predicted)	Rin2_predicted	1.7	0.000270778	0.004549291

1373148_at	carboxypeptidase X 2 (M14 family) (predicted)	Cpxm2_predicted	2.8	0.000274711	0.004596585
1391489_at	immunity-related GTPase family, M	Irgm	3.2	0.000275915	0.004600963
1388745_at	sema domain, immunoglobulin domain (Ig), transmembrane domain (TM) and short cytoplasmic domain, (semaphorin) 4A	Sema4a	1.7	0.000276371	0.004600963
1386881_at	insulin-like growth factor binding protein 3	Igfbp3	3.0	0.000277548	0.004608172
1380079_at	similar to mKIAA2005 protein	LOC500015	2.0	0.000277569	0.004608172
1388511_at	centaurin, delta 2	Centd2	1.6	0.000280001	0.004633263
1381190_at	---	---	3.6	0.000281458	0.004643352
1371504_at	RGD1561181 (predicted) /// similar to 60S ribosomal protein L37a	LOC679823 /// LOC687187 /// LOC688094 /// LOC689401 /// RGD1561181_predicted	1.5	0.000285384	0.004693989
1375714_at	similar to Erbb2 interacting protein isoform 2 (predicted)	RGD1562952_predicted	2.5	0.000285976	0.004695834
1372070_at	interferon gamma inducible protein 30	Ifi30	3.0	0.000287001	0.004697113
1377974_at	Integrin beta 8 (predicted)	Itgb8_predicted	1.9	0.000290944	0.004756898
1385426_at	similar to hypothetical protein FLJ20647 (predicted)	RGD1305326_predicted	2.1	0.000292121	0.004770133
1367777_at	2,4-dienoyl CoA reductase 1, mitochondrial	Decr1	1.5	0.000293313	0.004781377
1377794_at	Transcribed locus	---	1.6	0.000294054	0.00478871
1378453_at	---	---	2.1	0.000294591	0.004792714
1377161_at	Transcribed locus	---	3.0	0.000295256	0.00479878
1373923_at	Transcribed locus	---	3.4	0.000298521	0.004836983
1389229_at	acid phosphatase-like 2	Acpl2	1.7	0.000298783	0.004836983
1383164_at	Transcribed locus	---	2.4	0.0003017	0.0048665
1372234_at	similar to Lix1 homolog (mouse) like	LOC499677	2.0	0.000301723	0.0048665
1375877_at	synaptotagmin IV	Syt4	1.7	0.000303617	0.004878719
1373959_at	protein phosphatase 2 (formerly 2A), regulatory subunit A (PR 65), beta isoform	Ppp2r1b	1.9	0.000303733	0.004878719
1374468_at	myeloid differentiation primary response gene 88	Myd88	2.1	0.000308703	0.004948877
1389568_at	similar to RIKEN cDNA 2810048G17 gene	RGD1308276	1.6	0.000310128	0.004966875
1376204_at	Transcribed locus	---	2.7	0.000311608	0.004985605
1383947_at	---	---	1.5	0.00031315	0.004995824
1382181_at	similar to RIKEN cDNA A530088I07 gene	LOC311984	1.9	0.00031416	0.005007076
1392180_at	Sp1 transcription factor	Sp1	2.0	0.000314495	0.005007571
1392648_at	mannose receptor, C type 1 (predicted)	Mrc1_predicted	3.1	0.000315326	0.005015945
1392234_at	Transcribed locus	---	2.6	0.000316778	0.005029307
1388666_at	ectodermal-neural cortex 1	Enc1	2.0	0.000320983	0.005081353
1387195_at	suppression of tumorigenicity 14 (colon carcinoma)	St14	1.8	0.00032147	0.005084166
1391181_at	Transcribed locus	---	1.6	0.000322026	0.005086271
1382366_at	phosphatidylinositol 3-kinase, catalytic, alpha polypeptide /// hypothetical gene supported by AF395897	LOC367779 /// Pik3ca	1.6	0.000322389	0.005086271
1367723_a_at	linker of T-cell receptor pathways	Lnk	2.6	0.00032286	0.005086271
1376891_at	Transcribed locus	---	2.0	0.000322953	0.005086271
1393458_s_at	similar to PHD finger protein 14 isoform 1 (predicted)	RGD1563764_predicted	1.8	0.000323344	0.005086271
1398600_at	Transcribed locus	---	1.9	0.000323458	0.005086271
1399096_at	Transcribed locus	---	1.8	0.000326372	0.00511254
1398751_at	ribosomal protein S7 /// similar to ribosomal protein S7	LOC497813 /// Rps7	2.0	0.00032713	0.005119537
1387675_at	---	---	2.8	0.000328054	0.005129125
1398872_at	ribosomal protein S13 /// similar to ribosomal protein S13	LOC683961 /// LOC684988 /// Rps13	1.9	0.000329901	0.005148206
1397917_at	---	---	1.9	0.000331907	0.005163352
1382522_at	matrin 3	Matr3	1.7	0.000333705	0.005173216

1377826_at	Transcribed locus	---	2.3	0.000337642	0.005196847
1398383_at	cytochrome b-561 (predicted)	Cyb561_predicted	1.8	0.000337812	0.005196847
1369960_at	FXYD domain-containing ion transport regulator 1	Fxyd1	1.7	0.000338289	0.005196847
1373997_at	Similar to Drctnnb1a (predicted)	RGD1562906_predicted	1.6	0.000344001	0.005262579
1374535_at	similar to expressed sequence AU041783 (predicted)	RGD1310668_predicted	2.0	0.000344269	0.005262579
1379425_at	similar to RIKEN cDNA 3110037I16 (predicted)	RGD1309802_predicted	2.4	0.000346231	0.005285857
1388199_at	tumor-associated calcium signal transducer 1	Tacstd1	2.8	0.000346434	0.005285857
1376599_at	ATPase family, AAA domain containing 2 (predicted)	Atad2_predicted	1.8	0.000349161	0.005302858
1392074_at	similar to hypothetical protein FLJ21986	LOC500046	2.2	0.000349888	0.005306176
1396411_at	phosphatidylinositol 3-kinase, catalytic, alpha polypeptide /// hypothetical gene supported by AF395897 /// similar to Phosphatidylinositol-4,5-bisphosphate 3-kinase catalytic subunit alpha isoform (PI3-kinase p110 subunit alpha) (PtdIns-3-kinase p110) (PI3K)	LOC367779 /// LOC685590 /// Pik3ca	2.0	0.000353748	0.00534292
1373866_at	similar to hypothetical protein FLJ13448	RGD1359509	1.5	0.000357063	0.005388042
1398789_at	ribosomal protein L37	Rpl37	1.5	0.00035767	0.005392265
1389034_at	ubiquitin specific peptidase 18	Usp18	8.2	0.000359748	0.005408714
1379495_at	Plexin domain containing 2 (predicted)	Plxdc2_predicted	1.9	0.000363183	0.005445433
1377705_at	Transcribed locus, moderately similar to XP_574280.1 similar to Ab2-143 [Rattus norvegicus]	---	1.7	0.000365179	0.005460426
1372027_at	Transcribed locus	---	1.7	0.000374065	0.005552902
1387351_at	fibrillin 1	Fbn1	2.0	0.000375879	0.005568157
1390425_at	Transcribed locus	---	1.6	0.000376108	0.005568157
1367901_at	glucuronidase, beta	Gusb	1.6	0.000378009	0.005586242
1393128_at	similar to RIKEN cDNA 2610029K21	RGD1311086	1.9	0.000380633	0.005615439
1373210_at	laminin, beta 1 (predicted)	Lamb1_predicted	1.9	0.00038135	0.005615439
1384068_at	cytoskeleton associated protein 2 (predicted)	Ckap2_predicted	3.5	0.000382473	0.005626932
1385155_at	leucine rich repeat (in FLII) interacting protein 1	Lrrfip1	1.9	0.000384075	0.005645462
1377117_at	---	---	1.8	0.000386489	0.005670797
1368921_a_at	CD44 antigen	Cd44	2.1	0.000390691	0.005715792
1392899_at	protein regulator of cytokinesis 1 (predicted)	Prc1_predicted	2.4	0.000390853	0.005715792
1378110_at	Transcribed locus	---	2.0	0.000390945	0.005715792
1389477_at	Transcribed locus	---	1.4	0.000395919	0.005769901
1390907_at	similar to zinc finger protein 84 (HPF2)	LOC687219 /// LOC691170	1.6	0.000396049	0.005769901
1398852_at	ribosomal protein S21	Rps21	1.5	0.000398023	0.005793531
1390443_at	Transcribed locus	---	1.9	0.000399735	0.005813304
1371498_at	JTV1	MGC125271	1.5	0.000402264	0.005832432
1373646_at	RAB15, member RAS onocogene family	Rab15	2.5	0.000403335	0.005833935
1388880_at	Transcribed locus	---	3.8	0.000403992	0.005833935
1377706_x_at	Transcribed locus, moderately similar to XP_574280.1 similar to Ab2-143 [Rattus norvegicus]	---	1.7	0.000404539	0.005836713
1388678_at	---	---	1.7	0.000407531	0.005864456
1389618_at	Transcribed locus	---	2.1	0.000408216	0.005869181
1383131_at	integrin beta 2	Itgb2	2.3	0.00040859	0.005869417
1381382_at	similar to Vacuolar ATP synthase subunit S1 precursor (V-ATPase S1 subunit) (predicted)	RGD1564936_predicted	1.8	0.00041382	0.005929016
1375523_at	similar to Myristoylated alanine-rich C-kinase substrate (MARCKS) (ACAMP-81) /// similar to Myristoylated alanine-rich C-kinase substrate (MARCKS) (Protein kinase C substrate 80 kDa protein)	LOC294446 /// LOC681252	1.8	0.00041473	0.005936882
1393347_at	integrin alpha L	Itgal	2.7	0.000416734	0.005944877
1367764_at	cyclin G1	Ccng1	1.7	0.000420241	0.005976639

1382094_at	---	---	2.0	0.000421177	0.005982314
1385051_at	Guanylate nucleotide binding protein 4 (predicted)	Gbp4_predicted	3.6	0.000423922	0.006005749
1390109_at	---	---	1.7	0.000425135	0.006017745
1384032_at	Transcribed locus	---	1.7	0.000426197	0.006027584
1398796_at	transmembrane emp24-like trafficking protein 10 (yeast)	Tmed10	1.5	0.000429391	0.006062331
1393352_at	similar to hypothetical protein MGC38960 (predicted)	RGD1310552_predicted	1.7	0.000431436	0.006076409
1371989_at	high mobility group nucleosomal binding domain 3	Hmgn3	1.8	0.000436718	0.006134195
1393853_at	---	---	3.4	0.000439689	0.0061549
1382277_at	---	---	2.6	0.000441443	0.006174191
1396803_at	THO complex 2 (predicted)	Thoc2_predicted	1.9	0.000444323	0.006203924
1377414_at	similar to DNA segment, Chr 19, ERATO Doi 386, expressed (predicted)	RGD1307934_predicted	1.7	0.000445811	0.006219418
1392386_at	CASP8 and FADD-like apoptosis regulator	Cflar	1.9	0.000451186	0.006278439
1386987_at	interleukin 6 receptor, alpha	Il6ra	2.5	0.000456333	0.006339329
1370956_at	decorin	Dcn	2.2	0.000459781	0.006360372
1383075_at	cyclin D1	Ccnd1	2.9	0.000461178	0.006373485
1388930_at	transmembrane protein 123	Tmem123	1.9	0.000462627	0.006383626
1368650_at	Kruppel-like factor 10	Klf10	1.9	0.000467748	0.006438091
1368412_a_at	protein tyrosine phosphatase, receptor type, O	Ptpro	2.5	0.000468433	0.006442127
1395042_at	EH domain binding protein 1 (predicted)	Ehbp1_predicted	1.6	0.000471334	0.006469437
1367676_at	high mobility group box 2 /// similar to High mobility group protein 2 (HMG-2) (predicted) /// similar to High mobility group protein 2 (HMG-2)	Hmgb2 /// LOC680704 /// LOC682545 /// RGD1559962_predicted /// RGD1561694_predicted /// RGD1564519_predicted	3.6	0.00047138	0.006469437
1376799_a_at	cytokine receptor-like factor 1 (predicted)	Crlf1_predicted	4.8	0.000471599	0.006469437
1370531_a_at	phospholipase D1	Pld1	1.9	0.000472361	0.006474489
1384380_at	Zinc finger, CCHC domain containing 7 (predicted)	Zcchc7_predicted	1.6	0.000475958	0.006507527
1372815_at	mago-nashi homolog, proliferation-associated (Drosophila) (predicted)	Magoh_predicted	1.8	0.00047786	0.00652811
1369670_at	Cd200 antigen	Cd200	1.5	0.000480091	0.006547713
1370810_at	cyclin D2	Ccnd2	2.7	0.000480576	0.006548889
1373843_at	Transcribed locus	---	1.9	0.000481353	0.006554052
1379604_at	apolipoprotein L, 3 /// similar to apolipoprotein L, 3 (predicted)	Apol3 /// LOC681351	1.5	0.000482039	0.006557965
1384139_at	gap junction membrane channel protein alpha 7	Gja7	3.1	0.000483709	0.006569808
1383753_at	---	---	1.7	0.000486808	0.006590133
1388816_at	Discs, large homolog 1 (Drosophila)	Dlgh1	1.4	0.000489532	0.006615438
1382009_at	chromodomain helicase DNA binding protein 1 (predicted)	Chd1_predicted	1.6	0.000490193	0.006615438
1383392_at	complement component 2	C2	2.0	0.000490909	0.006615438
1398886_at	similar to Selenoprotein H (predicted)	RGD1563348_predicted	1.6	0.000493864	0.006647357
1367710_at	proteasome (prosome, macropain) 28 subunit, beta	Psme2	1.4	0.000499684	0.006709233
1393227_at	---	---	2.9	0.000505543	0.006771508
1386874_at	ribosomal protein S15	Rps15	1.5	0.000509827	0.006817585
1380063_at	cholesterol 25-hydroxylase	Ch25h	2.0	0.000512372	0.00684606
1379982_at	Transcribed locus	---	1.9	0.000516492	0.006873191
1385458_a_at	similar to C11orf17 protein (predicted)	RGD1306959_predicted	2.2	0.000516931	0.006873472
1384889_at	Transcribed locus	---	2.1	0.000523575	0.006942942
1388947_at	eukaryotic translation initiation factor 5B /// similar to Eukaryotic translation initiation factor 5B (eIF-5B) (Translation initiation factor IF-2)	Eif5b /// LOC686553 /// LOC689581	1.5	0.000524353	0.006942942

1368533_at	hephaestin	Heph	1.9	0.000524808	0.006942942
1373463_at	procollagen, type V, alpha 2	Col5a2	2.8	0.00052502	0.006942942
1389791_at	ceroid-lipofuscinosis, neuronal 8	Cln8	1.6	0.000531775	0.007000008
1379400_at	Transcribed locus	---	1.9	0.000532197	0.007000008
1368331_at	chitobiase, di-N-acetyl-	Ctbs	1.5	0.000532406	0.007000008
1388827_at	H2A histone family, member V (predicted) /// similar to H2A histone family, member V isoform 1 (predicted) /// similar to H2A histone family, member V isoform 1	H2afv_predicted /// LOC685909 /// RGD1560813_predicted	1.9	0.000534068	0.007007317
1377761_at	glutamine-fructose-6-phosphate transaminase 2	Gfpt2	1.8	0.000534646	0.007007317
1371973_at	eukaryotic translation initiation factor 3, subunit 6	Eif3s6	1.6	0.000535074	0.007007317
1375796_at	interferon gamma induced GTPase	Igtp	5.2	0.000535092	0.007007317
1389226_at	stromal antigen 1 (predicted)	Stag1_predicted	1.5	0.000536052	0.007010127
1369342_at	ATPase, Cu++ transporting, alpha polypeptide	Atp7a	1.6	0.000536159	0.007010127
1381859_at	similar to F33H2.2	LOC684063 /// LOC689997	1.8	0.000545478	0.007103739
1373644_at	nuclear factor I/B	Nfib	1.6	0.000547093	0.007113503
1384163_at	Transcribed locus	---	1.6	0.000548679	0.007122869
1372029_at	similar to mannose-6-phosphate receptor binding protein 1	LOC316130	1.5	0.000549172	0.007123646
1371330_at	ribosomal protein L11	Rpl11	1.7	0.000552062	0.007144225
1367623_at	ribosomal protein L18	Rpl18	1.5	0.000553874	0.007153232
1372818_at	collectin sub-family member 12	Colec12	3.5	0.000553924	0.007153232
1393832_at	similar to RIKEN cDNA 0610033I05	RGD1306676	2.2	0.000555245	0.007162885
1375215_x_at	pyroglutamyl-peptidase I	Pgpep1	1.6	0.000559626	0.007213733
1367767_at	3-hydroxy-3-methylglutaryl-Coenzyme A lyase	Hmgcl	1.5	0.000562274	0.007242189
1387875_at	PTK2 protein tyrosine kinase 2	Ptk2	1.6	0.000565413	0.007271242
1387932_at	solute carrier family 1 (neuronal/epithelial high affinity glutamate transporter, system Xag), member 1	Slc1a1	1.5	0.000566015	0.007273295
1388866_at	Transcribed locus	---	1.6	0.000567649	0.007288593
1374222_at	solute carrier family 22 (organic cation transporter), member 18	Slc22a18	1.8	0.000568357	0.00729108
1380416_at	Transcribed locus	---	1.8	0.000569436	0.007294461
1371527_at	epithelial membrane protein 1	Emp1	2.6	0.000571376	0.007313614
1370253_at	ribosomal protein L22	Rpl22	2.2	0.000578094	0.007362322
1389761_at	Transcribed locus	---	2.3	0.000578605	0.007362322
1367846_at	S100 calcium-binding protein A4	S100a4	2.2	0.000581895	0.0073796
1375170_at	S100 calcium binding protein A11 (calizzarin)	S100a11	1.6	0.000584978	0.007394963
1390312_at	similar to mKIAA2005 protein (predicted) /// similar to sterile alpha motif domain containing 9-like /// similar to mKIAA2005 protein	LOC500013 /// LOC500015 /// RGD1561472_predicted	2.1	0.000585616	0.007394963
1367880_at	laminin, beta 2	Lamb2	1.9	0.000597288	0.007510856
1374298_at	Transcribed locus	---	1.9	0.000599343	0.007530935
1391630_at	Transcribed locus	---	4.0	0.000600004	0.007533482
1374292_at	similar to RIKEN cDNA 1110031I02	RGD1305007	1.6	0.000600906	0.007539052
1378671_at	CREB binding protein	Crebbp	1.5	0.00060919	0.007625543
1388622_at	nucleolar protein 5A	Nol5a	1.7	0.000611912	0.007652108
1384875_at	Transcribed locus	---	1.7	0.000612349	0.007652108
1373186_at	similar to RIKEN cDNA 5033405K12 (predicted)	RGD1311593_predicted	2.0	0.000613464	0.007655729
1378754_at	Transcribed locus	---	2.1	0.000616949	0.007681744
1372693_at	heterogeneous nuclear ribonucleoprotein A1	Hnrpa1	1.7	0.000623087	0.007736935
1379703_at	hypothetical LOC315055 (predicted)	RGD1311559_predicted	1.7	0.000624951	0.007752033
1378172_at	Phosphatase and actin regulator 2	Phactr2	1.6	0.000626928	0.007770698
1388408_at	similar to RIKEN cDNA 1110020C13	RGD1307129	1.6	0.000628987	0.007790346
1395204_at	Transcribed locus	---	2.7	0.000630956	0.007808467

1374449_at	cell division cycle associated 3 /// similar to cell division cycle associated 3 (predicted)	Cdca3 /// RGD1562067_predicted	4.6	0.000634025	0.007823282
1377168_at	copine family member IX	Cpne9	2.5	0.000636173	0.00783217
1396494_at	Patched homolog 1 (Drosophila)	Ptch1	2.2	0.000638324	0.007852768
1374390_at	similar to Protein UNQ655/PRO1286 homolog precursor	LOC686324 /// LOC690344	1.9	0.000639092	0.007856347
1387917_at	torsin A interacting protein 1	Tor1aip1	1.6	0.000640641	0.007859516
1398319_at	Nclone10 mRNA	---	2.1	0.000640784	0.007859516
1370866_at	ribosomal protein L41	Rpl41	1.5	0.00064506	0.007898088
1384948_at	trichorhinophalangeal syndrome I (predicted)	Trps1_predicted	2.3	0.000645369	0.007898088
1377262_at	similar to KIAA2010 protein (predicted)	RGD1309450_predicted	1.8	0.000656648	0.008000403
1384507_at	similar to hypothetical protein FLJ11127	LOC310190	2.1	0.000657594	0.008006005
1368835_at	signal transducer and activator of transcription 1	Stat1	2.0	0.000658623	0.008012591
1390348_at	folate receptor 2 (fetal) (predicted)	Folr2_predicted	2.9	0.000660538	0.008024028
1373699_at	Transcribed locus	---	2.7	0.000663272	0.008045758
1370605_s_at	leptin receptor	Lepr	2.0	0.000668895	0.00809021
1368901_at	thrombomodulin	Thbd	3.0	0.00066999	0.008091041
1383576_at	Transcribed locus	---	1.9	0.000674911	0.008126605
1382493_at	Transcribed locus, strongly similar to XP_223060.4 similar to centromere protein F (350/400kD) [Rattus norvegicus]	---	2.6	0.000678252	0.008160849
1391435_at	phospholipid transfer protein (predicted)	Pltp_predicted	3.9	0.000687709	0.008238487
1382008_at	similar to hypothetical protein FLJ11218	RGD1309804	2.5	0.0006917	0.008256224
1388555_at	thioredoxin-like 5 (predicted)	Txnl5_predicted	1.4	0.000697215	0.00829197
1388169_at	jumonji domain containing 1C	Jmjd1c	2.1	0.000700206	0.008314928
1389422_at	Transcribed locus	---	1.6	0.000700662	0.008314928
1374518_at	transmembrane protein 77	Tmem77	1.4	0.000702183	0.008326963
1379598_at	Transcribed locus	---	1.7	0.000707541	0.008372402
1385430_at	similar to LIM and senescent cell antigen-like domains 1 (predicted)	RGD1560732_predicted	1.6	0.000709729	0.008386223
1385682_at	similar to vitrin (predicted)	RGD1564128_predicted	2.9	0.000710865	0.008393615
1371695_at	translocated promoter region	Tpr	1.6	0.000711861	0.008399346
1398568_at	Synaptosomal-associated protein, 91kDa homolog (mouse)	Snap91	2.4	0.000713958	0.008418045
1392536_at	Transcribed locus	---	1.5	0.000715902	0.008428891
1374325_at	Transcribed locus	---	2.1	0.000717301	0.00843932
1372473_at	tight junction protein 1 (predicted)	Tjp1_predicted	1.5	0.000722452	0.008475214
1382311_at	TRAF2 binding protein	LOC310877	1.9	0.000723184	0.008475214
1391412_at	5'-3' exoribonuclease 2 (predicted)	Xrn2_predicted	1.9	0.000723444	0.008475214
1388999_at	Transcribed locus	---	2.0	0.000735548	0.008586444
1389083_at	similar to KIAA2010 protein (predicted)	RGD1309450_predicted	1.6	0.000737732	0.00859581
1397844_at	Rho GTPase activating protein 20	Arhgap20	1.8	0.000738481	0.00859581
1382307_at	protein phosphatase 1, regulatory (inhibitor) subunit 12A	Ppp1r12a	2.3	0.000746259	0.008662292
1383519_at	Hexokinase 2	Hk2	1.9	0.000749908	0.008698504
1388535_at	similar to erythroid differentiation-related factor 1 (predicted)	RGD1306820_predicted	1.7	0.000752217	0.00871914
1377671_at	Transcribed locus	---	1.6	0.000757351	0.008760136
1371040_at	solute carrier family 1 (neutral amino acid transporter), member 5	Slc1a5	1.7	0.000762792	0.008812896
1383589_at	---	---	2.2	0.000765061	0.008826747
1381980_at	Torsin A interacting protein 2	Tor1aip2	1.7	0.000765257	0.008826747
1374494_at	Transcribed locus	---	1.9	0.000770951	0.008873765
1383469_at	Transcribed locus	---	3.1	0.000772395	0.008884166
1375249_at	eukaryotic translation initiation factor 2C, 1 (predicted)	Eif2c1_predicted	2.1	0.000775497	0.008907387
1398761_at	ribosomal protein L5	Rpl5	1.6	0.000777933	0.008922897
1373092_at	Transcribed locus	---	1.6	0.000779691	0.008925406
1383226_at	zinc finger protein X-linked (predicted)	Zfx_predicted	1.8	0.000784952	0.008972121
1395325_s_at	transmembrane protein 32 (predicted)	Tmem32_predicted	1.6	0.000785882	0.008976522
1379882_a_at	Transcribed locus	---	2.6	0.000787731	0.00899139

1382314_at	interferon, alpha-inducible protein (clone IFI-15K) (predicted)	G1p2_predicted	8.7	0.000792394	0.009025155
1372845_at	ribonuclease P 21 subunit (human)	Rpp21	1.7	0.000796163	0.009039611
1375186_at	similar to zinc finger, CSL domain containing 2	LOC680594 /// LOC682119	1.8	0.000796802	0.009039611
1368172_a_at	lysyl oxidase	Lox	3.1	0.000796901	0.009039611
1385948_at	Transcribed locus	---	1.6	0.000800293	0.00906034
1370186_at	proteosome (prosome, macropain) subunit, beta type 9	Psmb9	3.4	0.000804509	0.009094546
1377627_at	similar to down-regulated in metastasis (predicted)	RGD1560606_predicted	1.9	0.000805873	0.009095741
1392717_at	Kinesin family member 1B	Kif1b	1.6	0.000806901	0.009096579
1383998_at	similar to KIAA2010 protein (predicted)	RGD1309450_predicted	1.6	0.000811328	0.009133964
1372604_at	similar to apolipoprotein L2; apolipoprotein L-II (predicted) /// similar to apolipoprotein L, 3 (predicted)	LOC686810 /// LOC688281 /// RGD1309808_predicted	2.5	0.000813534	0.009152539
1374108_at	low density lipoprotein-related protein 12 (predicted)	Lrp12_predicted	1.5	0.0008144	0.009153576
1391551_at	---	---	1.5	0.000814739	0.009153576
1383516_at	fibrinogen-like 2	Fgl2	2.2	0.000818064	0.009176249
1381228_at	Transcribed locus	---	4.3	0.000818104	0.009176249
1367794_at	alpha-2-macroglobulin	A2m	2.6	0.000818954	0.009176249
1377353_a_at	tumor necrosis factor (ligand) superfamily, member 13	Tnfsf13	1.6	0.000820858	0.009184672
1398401_at	tudor domain containing 3	Tdrd3	1.8	0.000822875	0.009187546
1375559_at	similar to thyroid receptor-interacting protein 6	LOC686323	1.5	0.000823395	0.009187546
1390051_at	similar to mKIAA0774 protein (predicted)	RGD1562533_predicted	1.9	0.000823753	0.009187546
1398448_at	Transcribed locus	---	1.5	0.000831779	0.009237741
1367582_at	ribosomal protein L29	Rpl29	1.6	0.000837111	0.00928442
1368127_at	neuraminidase 2	Neu2	1.7	0.000838644	0.009295159
1393048_at	Adrenergic receptor, alpha 2a	Adra2a	2.7	0.000841943	0.009312892
1382387_at	Transmembrane protein 16A (predicted)	Tmem16a_predicted	2.1	0.000842844	0.009316594
1383383_at	Transcribed locus	---	2.1	0.000843473	0.009317287
1385804_x_at	WD repeat domain 36 (predicted)	Wdr36_predicted	1.8	0.000844143	0.009318432
1367826_at	nuclear factor, erythroid derived 2, like 2	Nfe2l2	1.5	0.000850419	0.009362576
1387294_at	SH3-domain binding protein 5 (BTK-associated)	Sh3bp5	1.8	0.000862822	0.009463654
1389091_at	ubiquitin specific peptidase 3	Usp3	1.7	0.000863627	0.009463654
1377622_at	hepatitis B virus x associated protein (predicted)	Hbxap_predicted	1.9	0.000865754	0.009480637
1374151_at	Transmembrane 6 superfamily member 1 (predicted)	Tm6sf1_predicted	2.0	0.000866864	0.009486482
1387031_at	endoplasmic reticulum protein 29	Erp29	1.5	0.000870861	0.009508976
1398432_at	similar to Ankyrin repeat domain protein 28 (predicted)	RGD1559931_predicted	1.7	0.000870927	0.009508976
1384310_at	Transcribed locus	---	2.1	0.000872211	0.009508976
1395413_at	low density lipoprotein receptor-related protein 6 (predicted)	Lrp6_predicted	1.6	0.000872388	0.009508976
1388762_at	IQ motif containing GTPase activating protein 1 (predicted)	Iqgap1_predicted	2.1	0.000882657	0.009595865
1383176_at	similar to C11orf17 protein (predicted)	RGD1306959_predicted	1.9	0.000882694	0.009595865
1389254_at	Glutamine and serine rich 1 (predicted)	Qser1_predicted	1.8	0.000886088	0.009626406
1393322_at	TAF15 RNA polymerase II, TATA box binding protein (TBP)-associated factor (predicted)	Taf15_predicted	1.7	0.00089038	0.009666648
1385006_at	alpha thalassemia/mental retardation syndrome X-linked homolog (human)	Atrx	1.4	0.000895393	0.009708254
1382268_at	A kinase (PRKA) anchor protein 13	Akap13	1.6	0.000898403	0.009728063
1367618_a_at	guanine nucleotide binding protein (G protein), beta polypeptide 2 like 1	Gnb2l1	1.5	0.000902335	0.009755149
1371412_a_at	Neuronal regeneration related protein	Nrep	1.5	0.000902683	0.009755149
1384124_at	Transcribed locus	---	2.3	0.000903366	0.009756116
1390264_at	similar to RIKEN cDNA 1200014J11 (predicted)	RGD1308139_predicted	1.7	0.00090708	0.009777575

1390709_at	triple functional domain (PTPRF interacting)	Trio	1.6	0.00091024	0.009804598
1378264_at	Nuclear autoantigenic sperm protein (histone-binding)	Nasp	1.7	0.000915264	0.009852268
1389293_at	Transcribed locus	---	1.4	0.000920738	0.009904704
1368708_a_at	dopamine receptor 2	Drd2	1.9	0.000924024	0.009914126
1375362_at	similar to RIKEN cDNA 2010106G01 (predicted)	RGD1563001_predicted	1.5	0.000925603	0.009922602
1383051_at	Synaptotagmin binding, cytoplasmic RNA interacting protein	Syncrip	1.6	0.000926021	0.009922602
1396850_at	---	---	2.3	0.000929226	0.009950458
1381014_at	interferon-induced protein 44	Ifi44	1.9	0.000930406	0.009956616
1372729_at	protein C receptor, endothelial	Procr	1.8	0.00093185	0.009965585
1372030_at	zinc finger, FYVE domain containing 21 (predicted)	Zfyve21_predicted	1.5	0.00093718	0.01000634
1370201_at	calbindin 1	Calb1	2.5	0.000938009	0.01000634
1382691_at	---	---	2.0	0.000938094	0.01000634
1367685_at	ribosomal protein S27a	Rps27a	1.6	0.000939391	0.010013681
1370260_at	adducin 3 (gamma)	Add3	1.5	0.000948318	0.010090655
1367776_at	cell division cycle 2 homolog A (S. pombe)	Cdc2a	3.8	0.000953465	0.010130872
1388481_at	ribosomal protein S28 /// similar to 40S ribosomal protein S28 (predicted) /// similar to 40S ribosomal protein S28	LOC684733 /// LOC691531 /// RGD1564194_predicted /// Rps28	1.7	0.000954269	0.010132871
1383603_at	Transcribed locus	---	1.5	0.000963243	0.010205275
1371313_at	ribosomal protein L23a	Rpl23a	1.7	0.00096355	0.010205275
1370904_at	major histocompatibility complex, class II, DM alpha	Hla-dma	2.7	0.000964796	0.010210808
1398760_at	ribosomal protein L35a	Rpl35a	1.6	0.000965954	0.010210808
1368671_at	sushi-repeat-containing protein	Srpx	2.1	0.000968945	0.010229273
1383655_at	TNFAIP3 interacting protein 2	Tnip2	2.0	0.000973011	0.010252455
1367934_at	ribosomal protein L39	Rpl39	1.7	0.000984398	0.010339318
1390460_at	Transcribed locus	---	1.7	0.000989996	0.010384853
1391145_at	Transcribed locus	---	1.5	0.000996164	0.010436248
1389836_a_at	Tissue inhibitor of metalloproteinase 3 (Sorsby fundus dystrophy, pseudoinflammatory)	Timp3	1.8	0.001003941	0.010499503
1367630_at	ribosomal protein S11	Rps11	1.5	0.001004117	0.010499503
1370195_at	synaptosomal-associated protein 23	Snap23	1.8	0.001005276	0.010504938
1370883_at	RT1 class II, locus Da	RT1-Da	4.8	0.00100697	0.010515968
1391613_at	Similar to hypothetical protein DKFZp761N1114 (predicted)	RGD1566090_predicted	1.9	0.001015141	0.010587682
1373732_at	acid phosphatase 6, lysophosphatidic	Acp6	1.5	0.001023737	0.010623617
1399018_at	similar to cisplatin resistance-associated overexpressed protein (predicted)	RGD1307981_predicted	1.5	0.001025999	0.010633664
1373250_at	similar to Anillin (predicted)	RGD1566097_predicted	4.0	0.001028156	0.010633707
1370113_at	baculoviral IAP repeat-containing 3	Birc3	1.4	0.001028854	0.010633707
1381410_a_at	---	---	2.3	0.001029235	0.010633707
1397867_at	---	---	1.7	0.001030857	0.01064378
1373357_at	Transcribed locus	---	3.4	0.001034214	0.01067174
1372175_at	Transcribed locus	---	1.6	0.001035724	0.010680618
1372417_at	SERTA domain containing 1	Sertad1	1.4	0.001038993	0.010701542
1371761_at	ribosomal protein L34 (predicted) /// similar to ribosomal protein L34	LOC680170 /// LOC684829 /// Rpl34_predicted	1.5	0.001039054	0.010701542
1389611_at	---	---	1.6	0.001039846	0.010702468
1372259_at	DEK oncogene (DNA binding)	Dek	1.9	0.001040549	0.010702468
1383954_at	Transcribed locus	---	1.8	0.001041336	0.010702468
1385380_at	Transcribed locus	---	3.1	0.001044503	0.010704212
1389500_at	similar to ras homolog gene family, member U	LOC678766	1.8	0.00104452	0.010704212
1372137_at	biogenesis of lysosome-related organelles complex-1, subunit 1 (predicted)	Bloc1s1_predicted	1.4	0.001052286	0.010756992
1383520_at	---	---	1.4	0.001057285	0.010789283

1387343_at	CCAAT/enhancer binding protein (C/EBP), delta	Cebpd	2.2	0.00105894	0.010791478
1379694_at	enhancer of polycomb homolog 2 (Drosophila) (predicted)	Epc2_predicted	2.0	0.001065705	0.010826474
1383062_at	Glutaredoxin 2 (thioltransferase)	Glrx2	1.5	0.001066361	0.010826474
1390177_at	Transcribed locus	---	1.9	0.001066781	0.010826474
1372493_at	coiled-coil domain containing 23	Ccdc23	1.6	0.001069115	0.010834755
1397767_at	Transcribed locus	---	1.7	0.00107541	0.010871145
1383251_at	poly (ADP-ribose) polymerase family, member 2 (predicted)	Parp2_predicted	1.6	0.001085923	0.010937702
1398888_at	H3 histone, family 3B	H3f3b	1.5	0.001088344	0.010955379
1377999_at	Transcribed locus	---	2.2	0.001092842	0.010993922
1376096_a_at	Transcribed locus	---	1.9	0.001095772	0.011003208
1379217_at	Transcribed locus	---	1.5	0.001100578	0.011037375
1399025_at	similar to mesoderm induction early response 1 (MI-ER1) (predicted)	RGD1562337_predicted	1.5	0.001104569	0.011057795
1388830_at	protein kinase N2	Pkn2	1.4	0.001106173	0.011067117
1370048_at	endothelial differentiation, lysophosphatidic acid G-protein-coupled receptor, 2	Edg2	1.5	0.001110198	0.011100285
1373182_at	---	---	1.6	0.001111513	0.011100285
1398353_at	SAR1 gene homolog A (S. cerevisiae)	Sar1a	1.5	0.001113111	0.011102756
1388557_at	complement component 7 /// tubulin, beta 2c	C7 /// Tubb2c	3.4	0.001117352	0.011138303
1385397_at	Ab1-219	LOC499991	3.1	0.001120018	0.011156156
1393596_at	alpha thalassemia/mental retardation syndrome X-linked homolog (human)	Atrx	2.0	0.001125019	0.011194385
1395203_at	Transcribed locus	---	1.9	0.001134432	0.01126759
1386984_at	MAD homolog 4 (Drosophila)	Smad4	1.8	0.00114409	0.011356669
1368917_at	nudix (nucleoside diphosphate linked moiety X)-type motif 1	Nudt1	1.4	0.001145256	0.011361381
1389528_s_at	Jun oncogene	Jun	2.8	0.001156075	0.011454889
1392502_at	AT hook containing transcription factor 1 (predicted)	Ahctf1_predicted	1.7	0.001164056	0.011506249
1373776_at	---	---	2.0	0.001167777	0.011529168
1381967_at	RNA-binding region (RNP1, RRM) containing 2	Rnpc2	1.4	0.00116895	0.011533823
1368841_at	transcription factor 4	Tcf4	1.4	0.001172119	0.011558163
1395060_at	Inner membrane protein, mitochondrial	Immt	2.1	0.001176874	0.011578942
1383467_at	enhancer of polycomb homolog 2 (Drosophila) (predicted)	Epc2_predicted	1.9	0.001177042	0.011578942
1393687_at	Transcribed locus	---	1.7	0.001181012	0.011594924
1390019_at	H3 histone, family 3B	H3f3b	1.7	0.001181487	0.011594924
1375637_at	similar to RIKEN cDNA 1110003E01	RGD1311122	1.5	0.00119171	0.01167436
1392971_at	Transcribed locus	---	1.9	0.00119691	0.01171135
1386945_a_at	protein kinase, AMP-activated, beta 1 non-catalytic subunit	Prkab1	1.6	0.001203131	0.011758231
1385751_at	thrombospondin 2	Thbs2	3.1	0.001206407	0.011769264
1371078_at	RT1 class Ib, locus Aw2	RT1-Aw2	1.7	0.001208551	0.011778151
1369414_at	syntaxin binding protein 3	Stxbp3	1.4	0.001210182	0.011778151
1367663_at	proteasome (prosome, macropain) 28 subunit, alpha	Psme1	1.9	0.001211066	0.011779787
1395654_at	Transcribed locus	---	2.0	0.001222606	0.011849986
1387890_at	ribosomal protein S29	Rps29	1.4	0.001225384	0.011862932
1373937_at	FYVE and coiled-coil domain containing 1 (predicted)	Fyco1_predicted	1.6	0.001226484	0.011866595
1392471_at	Transcribed locus	---	1.5	0.001228589	0.011872927
1397673_at	CUG triplet repeat, RNA binding protein 2	Cugbp2	1.7	0.001229304	0.011872927
1379683_at	Similar to hypothetical protein MGC17839 (predicted)	RGD1565079_predicted	1.5	0.001235415	0.011898489
1381979_at	sulfatase modifying factor 2	Sumf2	1.6	0.00124006	0.011932825
1372995_at	protein kinase D2	Prkd2	1.5	0.001240584	0.011932825
1378645_at	Transcribed locus	---	2.5	0.001249416	0.011996726
1388763_at	similar to Calponin-2 (Calponin H2, smooth muscle) (Neutral calponin)	LOC690976	1.5	0.001253493	0.012003577
1374134_at	Transcribed locus	---	1.9	0.001253521	0.012003577
1371044_at	phosphodiesterase 7A	Pde7a	1.6	0.001253778	0.012003577
1392888_at	glypican 4	Gpc4	1.7	0.001261573	0.012056527

1381755_x_at	similar to RIKEN cDNA 4831426I19 (predicted)	RGD1304563_predicted	2.0	0.001262241	0.012056527
1372097_at	---	---	2.6	0.001266322	0.012081474
1374474_at	copine VIII (predicted)	Cpne8_predicted	1.5	0.001273134	0.012132396
1372811_at	kinectin 1 (predicted)	Ktn1_predicted	1.7	0.001275666	0.012149484
1374540_at	cell division cycle associated 7	Cdca7	1.6	0.001284405	0.012218558
1389968_at	eukaryotic translation initiation factor 3, subunit 10 (theta)	Eif3s10	1.6	0.00128756	0.012234423
1384792_at	pre-mRNA processing factor 40 homolog A (yeast) (predicted)	Prpf40a_predicted	1.6	0.001290319	0.012246487
1374463_at	similar to quaking homolog, KH domain RNA binding isoform HQK-6 /// similar to quaking homolog, KH domain RNA binding isoform HQK-5	LOC499022 /// LOC684079	1.6	0.001297999	0.012312273
1399046_at	---	---	1.4	0.001304832	0.012362832
1373054_at	CDW92 antigen	Cdw92	1.8	0.001318551	0.012471266
1374232_at	phosphatidylinositol 3-kinase, catalytic, alpha polypeptide	Pik3ca	1.9	0.001320747	0.012478539
1387342_at	guanine nucleotide binding protein (G protein), gamma 5 subunit	Gng5	2.1	0.00132366	0.012478539
1371428_at	similar to Protein transport protein SEC61 gamma subunit	LOC681549 /// LOC689134	2.0	0.001326005	0.012491485
1380369_at	Transcribed locus	---	1.5	0.001327827	0.012501488
1372466_at	Transcribed locus	---	1.5	0.001333008	0.01253853
1367922_at	a disintegrin and metalloproteinase domain 17 (tumor necrosis factor, alpha, converting enzyme)	Adam17	1.8	0.001333286	0.01253853
1379271_at	similar to Suppressor of cytokine signaling 5 (predicted)	RGD1564914_predicted	1.6	0.001339154	0.012579332
1389306_at	---	---	1.8	0.001348758	0.012655093
1394419_at	similar to RIKEN cDNA 6530401L14 gene	RGD1309107	1.8	0.001361154	0.012715638
1384795_at	zinc finger, matrin-like (predicted)	Zfml_predicted	1.5	0.001361396	0.012715638
1396339_at	Transcribed locus	---	1.5	0.001365151	0.012743471
1369174_at	MAD homolog 1 (Drosophila)	Smad1	1.8	0.001367178	0.012748378
1376411_at	similar to ADP-ribosylation factor-like 2-like 1	LOC304037	1.7	0.001368749	0.012748378
1397929_at	Transcribed locus	---	2.7	0.001369863	0.012748378
1390386_at	caspase 3, apoptosis related cysteine protease	Casp3	2.4	0.001369944	0.012748378
1378586_at	Cytokine inducible SH2-containing protein	Cish	1.6	0.001372311	0.012748378
1384280_at	Transcribed locus	---	2.7	0.001372652	0.012748378
1377130_at	Transcribed locus	---	2.2	0.001374925	0.012762288
1382680_at	---	---	1.5	0.001378533	0.012788558
1393252_at	Transcribed locus	---	3.8	0.00138294	0.012821275
1376098_a_at	myosin IG	Myo1g	1.9	0.001383619	0.012821275
1380449_at	Transcribed locus	---	1.5	0.001387173	0.012826724
1383564_at	interferon regulatory factor 7	Irf7	3.4	0.001387815	0.012826724
1367576_at	glutathione peroxidase 1	Gpx1	1.4	0.001388106	0.012826724
1379344_at	Cytochrome b-245, beta polypeptide	Cybb	5.4	0.001391455	0.012845219
1384200_at	similar to RIKEN cDNA 4931400A14 (predicted)	RGD1307234_predicted	1.4	0.00139167	0.012845219
1399005_at	protein phosphatase 2, regulatory subunit B (B56), alpha isoform (predicted)	Ppp2r5a_predicted	1.6	0.00139584	0.012876484
1372449_at	Transcribed locus	---	1.4	0.001397802	0.012879943
1371033_at	RT1 class II, locus Bb	RT1-Bb	6.9	0.001399155	0.012879943
1387131_at	serine (or cysteine) peptidase inhibitor, clade I, member 1	Serpini1	1.8	0.001399347	0.012879943
1369996_at	polymerase (RNA) II (DNA directed) polypeptide F	Polr2f	1.5	0.001407267	0.012923912
1374171_at	Transcribed locus	---	2.6	0.001410665	0.012947889
1397719_at	Transcribed locus	---	1.4	0.001417314	0.012975695
1375006_at	CDNA clone IMAGE:7318427	---	1.7	0.001419249	0.012975993
1394493_at	Casein kinase 1, alpha 1	Csnk1a1	2.2	0.001423846	0.013003655
1388485_at	chemokine (C-X-C motif) ligand 14	Cxcl14	5.1	0.001423856	0.013003655
1382923_at	synaptotagmin binding, cytoplasmic RNA interacting protein	Syncrip	1.7	0.001426443	0.013020053

1374404_at	Jun oncogene	Jun	2.4	0.00144241	0.013143911
1376629_at	---	---	1.4	0.001445583	0.013158248
1373376_at	Transcribed locus	---	1.4	0.001446572	0.013159961
1374337_at	Transcribed locus	---	2.7	0.001449123	0.013168607
1371150_at	cyclin D1	Ccnd1	1.9	0.001455329	0.013186526
1398256_at	interleukin 1 beta	Il1b	1.9	0.001463576	0.013215173
1383240_at	integrin, alpha 6	Itga6	1.6	0.001463889	0.013215173
1377102_at	transmembrane protein 63a (predicted)	Tmem63a_predicted	1.4	0.001466996	0.013235956
1390788_a_at	WD repeat domain 36 (predicted)	Wdr36_predicted	2.0	0.001470785	0.013255605
1383115_at	Protein kinase, cGMP-dependent, type 1 (mapped)	Prkg1	1.7	0.001472773	0.013266244
1387157_at	polyamine modulated factor 1 binding protein 1	Pmfbp1	1.5	0.001485127	0.013350227
1386899_at	cathepsin H	Ctsh	1.6	0.001485307	0.013350227
1368006_at	lysosomal-associated protein transmembrane 5	Laptm5	2.3	0.001486207	0.013350227
1392542_at	similar to CDC42 small effector 2 (predicted) /// hypothetical protein LOC686503 /// hypothetical protein LOC691031	LOC686503 /// LOC691031 /// RGD1563924_predicted	1.5	0.001487812	0.013350227
1392489_at	Transcribed locus	---	1.7	0.001489044	0.013350227
1376194_at	Transcribed locus	---	1.6	0.001494806	0.013370578
1387995_a_at	interferon induced transmembrane protein 3	Ifitm3	2.4	0.001494923	0.013370578
1367675_at	calcium and integrin binding 1 (calmyrin)	Cib1	1.4	0.001504151	0.013438502
1390189_at	similar to zinc finger protein 277 isoform 1	LOC298977	1.4	0.00150928	0.013476442
1387969_at	chemokine (C-X-C motif) ligand 10	Cxcl10	7.5	0.001513096	0.013496422
1392660_at	Transcribed locus	---	1.4	0.001522041	0.013561498
1388244_s_at	ribosomal protein SA	Rpsa	1.8	0.001523763	0.013562281
1376045_at	Transcribed locus	---	1.6	0.001524602	0.013562281
1372463_at	FCH domain only 2 (predicted)	Fcho2_predicted	1.9	0.001533724	0.013636053
1369160_a_at	solute carrier family 4, sodium bicarbonate cotransporter, member 7	Slc4a7	2.0	0.001545634	0.013727024
1368633_at	cysteine-rich secretory protein 1	Crisp1	24.0	0.001547295	0.013727024
1397584_at	Transcribed locus	---	1.8	0.001555081	0.013758992
1395010_at	Transcribed locus	---	1.6	0.00156068	0.013793693
1376144_at	poly (ADP-ribose) polymerase family, member 9 (predicted)	Parp9_predicted	2.4	0.001571657	0.0138758
1392838_at	similar to CG13957-PA (predicted)	RGD1309995_predicted	1.6	0.001573322	0.013883047
1393351_at	Transcribed locus	---	2.6	0.001592687	0.013989624
1398917_at	ribosomal protein L7	Rpl7	1.5	0.001593094	0.013989624
1384063_at	collagen triple helix repeat containing 1	Cthrc1	4.2	0.001593169	0.013989624
1398265_at	ATP-binding cassette, sub-family C (CFTR/MRP), member 9	Abcc9	1.7	0.001593337	0.013989624
1383396_at	fibronectin type III domain containing 3a (predicted)	Fndc3a_predicted	1.4	0.001594756	0.013989624
1370054_at	cyclin-dependent kinase inhibitor 2C (p18, inhibits CDK4)	Cdkn2c	1.9	0.001600381	0.014024011
1382252_at	Transcribed locus	---	1.9	0.001602937	0.014031712
1367588_a_at	ribosomal protein L13A	Rpl13a	1.5	0.001603819	0.014031712
1392669_at	similar to CG10084-PA	RGD1308297	1.5	0.001623625	0.014162022
1368050_at	cyclin L1	Ccnl1	1.7	0.001637484	0.014257989
1373143_at	similar to hypothetical protein FLJ10652	RGD1309621	1.6	0.001639266	0.014265949
1375449_at	Zinc finger homeobox 1b	Zfhx1b	2.0	0.001649276	0.014307647
1374063_at	splicing factor, arginine/serine-rich 3 (SRp20) (predicted)	Sfrs3_predicted	1.4	0.001672163	0.014438933
1378831_at	Similar to KIAA0456 protein (predicted)	RGD1566016_predicted	2.2	0.001676253	0.014465379
1368305_at	caspase 6	Casp6	1.7	0.001678012	0.01447296
1388710_at	ras responsive element binding protein 1 (predicted)	Rreb1_predicted	2.1	0.001681419	0.014492598
1370907_at	beta galactoside alpha 2,6 sialyltransferase 1	St6gal1	1.5	0.001682506	0.014492598
1382568_at	Transcribed locus	---	1.5	0.001684476	0.014492598
1368344_at	glutamic acid decarboxylase 1	Gad1	2.1	0.00168498	0.014492598
1373847_at	transmembrane 4 superfamily member 1 (predicted)	Tm4sf1_predicted	1.8	0.001692553	0.014533875

1376848_at	Transcribed locus	---	2.5	0.001693027	0.014533875
1380690_at	Transcribed locus, strongly similar to NP_075544.1 zipper- and sterile alpha motif-containing kinase isoform 1 [Mus musculus]	---	1.6	0.001695107	0.014544145
1399106_at	similar to CG17059-PA	LOC681389 /// LOC684000	1.4	0.001696572	0.014549123
1374221_at	Solute carrier family 29 (nucleoside transporters), member 3	Slc29a3	2.1	0.001699416	0.014558327
1376465_at	Sim ilar to glucocorticoid induced gene 1 (predicted)	RGD1563869_predicted	2.0	0.00170314	0.014575053
1375857_at	similar to Myoferlin (Fer-1 like protein 3) (predicted)	RGD1564216_predicted	2.5	0.00170555	0.014588086
1397866_at	serine (or cysteine) proteinase inhibitor, clade B, member 6b	Serpinb6b	1.6	0.001708922	0.014609332
1398764_at	ribosomal protein L21	Rpl21	1.4	0.001711091	0.014614638
1385068_at	Transcribed locus	---	1.5	0.001714148	0.014631191
1387775_at	general transcription factor IIa 2	Gtf2a2	2.0	0.001716024	0.014639607
1391480_at	Transcribed locus	---	1.5	0.001717342	0.014643259
1371557_at	THAP domain containing 4	Thap4	1.4	0.00172731	0.014713
1382489_at	similar to WD repeat domain 11 protein (predicted)	RGD1564964_predicted	2.6	0.001728704	0.014717252
1393585_at	Transcribed locus	---	1.5	0.001738342	0.014776356
1377299_at	nuclear autoantigenic sperm protein (histone-binding)	Nasp	1.8	0.001741342	0.014780346
1389742_at	Transcribed locus	---	1.8	0.001741702	0.014780346
1396009_at	---	---	2.5	0.001743304	0.014780346
1383484_at	male-specific lethal 2-like 1 (Drosophila) (predicted)	Msl2l1_predicted	1.7	0.001744841	0.01478575
1368806_at	selenoprotein P, plasma, 1	Sepp1	1.4	0.001748354	0.014807888
1384692_at	---	---	1.7	0.001758483	0.014878357
1379676_a_at	deoxyribonuclease 1-like 1	Dnase1l1	1.4	0.001764536	0.014921897
1398366_at	Transcribed locus, moderately similar to XP_579763.1 hypothetical protein XP_579763 [Rattus norvegicus]	---	1.6	0.001770689	0.014958547
1377400_at	HIV-1 Rev binding protein 2 (predicted)	Hrb2_predicted	1.4	0.001772575	0.014964264
1375538_at	Vinculin (predicted)	Vcl_predicted	2.3	0.001773185	0.014964264
1372668_at	LIM domains containing 1 (predicted)	Limd1_predicted	1.8	0.001781494	0.015011275
1391830_at	copine VIII (predicted)	Cpne8_predicted	2.8	0.001790174	0.015058775
1373036_at	similar to Ras GTPase-activating-like protein IQGAP2 (predicted)	RGD1561455_predicted	1.6	0.001790793	0.015058775
1393702_at	5'-3' exoribonuclease 2 (predicted)	Xrn2_predicted	1.4	0.001795881	0.015093841
1396053_at	neural precursor cell expressed, developmentally down-regulated gene 9	Nedd9	2.2	0.001801089	0.015120853
1387084_at	dipeptidylpeptidase 4	Dpp4	2.0	0.001801657	0.015120853
1392484_at	---	---	1.5	0.001807764	0.015147273
1370449_at	purinergic receptor P2Y, G-protein coupled, 14	P2ry14	2.0	0.001811296	0.015168972
1389840_at	splicing factor 3b, subunit 1	Sf3b1	1.6	0.001812198	0.015168972
1389867_at	Transcribed locus	---	1.5	0.001818039	0.015210121
1383175_a_at	similar to C11orf17 protein (predicted)	RGD1306959_predicted	2.2	0.001824177	0.015245967
1395485_s_at	myosin Ib	Myo1b	2.2	0.00182599	0.015250877
1385637_at	Similar to sushi, von Willebrand factor type A, EGF and pentraxin domain containing 1	LOC685899	1.7	0.001827404	0.015250877
1393029_at	CUG triplet repeat, RNA binding protein 1	Cugbp1	1.7	0.00182764	0.015250877
1383889_at	Transcribed locus	---	1.6	0.001828957	0.015250877
1389397_at	Transcribed locus	---	1.7	0.001829401	0.015250877
1388212_a_at	RT1 class Ib, locus S3	RT1-S3	2.8	0.001845306	0.015360119
1380336_at	Transcribed locus	---	2.6	0.001856495	0.015429828
1375358_at	Transcribed locus	---	1.8	0.001859962	0.015443038
1393159_at	Glutaredoxin 2 (thioltransferase)	Glrx2	1.4	0.001867329	0.015496383
1399004_at	similar to CG6878-PA	LOC679572 /// LOC683563	1.8	0.001872184	0.015513191
1374448_at	reversion-inducing-cysteine-rich protein with kazal motifs (predicted)	Reck_predicted	1.4	0.001876819	0.015528128
1390945_at	zinc finger protein 292	Znf292	1.7	0.001878162	0.015528563

1371824_at	adenylate kinase 3-like 1	Ak3l1	1.6	0.00187876	0.015528563
1371924_at	olfactomedin-like 3 (predicted)	Olfml3_predicted	1.9	0.001887598	0.015593777
1376578_at	euchromatic histone methyltransferase 1 (predicted)	Ehmt1_predicted	1.8	0.001895463	0.015643039
1396305_at	Transcribed locus	---	1.9	0.001913991	0.015772205
1385346_at	WW domain binding protein 4	Wbp4	1.9	0.001916816	0.015787579
1389130_at	E74-like factor 2	Elf2	1.8	0.001920473	0.015809779
1371659_at	ras homolog gene family, member C (predicted)	Rhoc_predicted	2.3	0.001928346	0.015858716
1371544_at	similar to Enhancer of rudimentary homolog	LOC678886 /// LOC681415	1.4	0.001935673	0.015911017
1396529_at	Transcribed locus	---	2.1	0.001940072	0.015932299
1378543_at	heterogeneous nuclear ribonucleoprotein A2/B1 (predicted)	Hnrpa2b1_predicted	1.5	0.001943189	0.015932991
1393210_at	---	---	2.9	0.001949368	0.015967731
1380071_at	poly (ADP-ribose) polymerase family, member 12 (predicted)	Parp12_predicted	3.2	0.001951481	0.015970267
1388772_at	LSM8 homolog, U6 small nuclear RNA associated (S. cerevisiae) (predicted)	Lsm8_predicted	1.8	0.001952761	0.015971392
1377833_at	similar to Rap1-interacting factor 1 (predicted)	RGD1562474_predicted	1.9	0.001953699	0.015971392
1383940_at	cell division cycle associated 1	Cdca1	2.3	0.001963441	0.016014877
1382482_at	Transcribed locus	---	1.9	0.001963887	0.016014877
1375957_at	Transcribed locus	---	1.7	0.001967287	0.016026718
1388143_at	procollagen, type XVIII, alpha 1	Col18a1	1.6	0.001986441	0.016142781
1385585_at	Transcribed locus	---	2.1	0.001988488	0.016143461
1368303_at	period homolog 2 (Drosophila)	Per2	2.6	0.002000994	0.016220971
1384292_at	docking protein 1	Dok1	2.1	0.002012471	0.016273904
1371928_at	cell division cycle associated 8	Cdca8	1.4	0.002019056	0.016303109
1398246_s_at	Fc receptor, IgG, low affinity III	Fcgr3	2.9	0.002024905	0.016342318
1370422_at	receptor-interacting serine-threonine kinase 3	Ripk3	1.7	0.00202938	0.016354373
1388054_a_at	chondroitin sulfate proteoglycan 2	Cspg2	2.1	0.002029382	0.016354373
1385533_at	Transcribed locus	---	1.4	0.002030772	0.016357563
1385227_at	trichorhinophalangeal syndrome I (predicted)	Trps1_predicted	1.8	0.00204404	0.016443272
1392938_s_at	similar to C11orf17 protein (predicted)	RGD1306959_predicted	2.1	0.002045412	0.016443272
1377103_at	midnolin (predicted)	Midn_predicted	1.6	0.002048769	0.016458219
1390229_at	Transcribed locus	---	1.6	0.002052251	0.016466059
1373405_at	CDNA clone IMAGE:7373898	---	1.4	0.002075045	0.01663272
1390034_at	Transcribed locus	---	1.5	0.002079299	0.01665059
1383712_at	---	---	1.6	0.002094657	0.016753818
1379260_at	Transcribed locus	---	1.4	0.002105041	0.016802032
1388932_at	laminin, alpha 5	Lama5	1.8	0.002105362	0.016802032
1373432_at	similar to Myristoylated alanine-rich C-kinase substrate (MARCKS) (ACAMP-81) /// similar to Myristoylated alanine-rich C-kinase substrate (MARCKS) (Protein kinase C substrate 80 kDa protein)	LOC294446 /// LOC681252	2.3	0.002116796	0.016876907
1373217_at	EH domain binding protein 1 (predicted)	Ehbp1_predicted	1.4	0.002118376	0.016881321
1392249_at	Similar to protein 4.1G (predicted)	RGD1563977_predicted	1.8	0.002120652	0.016883939
1392118_at	Transcribed locus	---	2.4	0.002126749	0.016915265
1397630_at	similar to HECT, C2 and WW domain containing E3 ubiquitin protein ligase 2 isoform 1	LOC316395	1.8	0.002128951	0.016917156
1373534_at	similar to SR rich protein	RGD1307395	1.7	0.002131071	0.016917156
1389287_at	CDNA clone IMAGE:7193711	---	2.1	0.002136173	0.016932901
1388488_at	LSM3 homolog, U6 small nuclear RNA associated (S. cerevisiae) (predicted)	Lsm3_predicted	1.6	0.002143652	0.016984002
1370963_at	growth arrest specific 7	Gas7	2.1	0.002150001	0.017017906
1383284_at	Transcribed locus	---	2.2	0.002153807	0.017039834
1374780_at	Transcribed locus	---	2.1	0.002165935	0.017111097
1370235_at	diazepam binding inhibitor	Dbi	1.4	0.002176853	0.0171726
1372758_at	Transcribed locus	---	1.7	0.00217922	0.017183037
1380425_at	Ribonuclease L (2',5'-oligoisoadenylate synthetase-dependent)	Rnasel	2.0	0.002186469	0.01722368

1370895_at	procollagen, type V, alpha 2	Col5a2	2.3	0.002211985	0.017341621
1382189_at	syndecan 2	Sdc2	1.6	0.002214754	0.017355061
1392770_at	neogenin	Neo1	1.4	0.002216905	0.01735549
1383661_at	Heparan sulfate (glucosamine) 3-O-sulfotransferase 3B1 (predicted)	Hs3st3b1_predicted	1.8	0.002227029	0.017393241
1392385_at	nuclear receptor coactivator 3	Ncoa3	1.8	0.002228677	0.017397503
1387024_at	dual specificity phosphatase 6	Dusp6	1.7	0.002231173	0.017397503
1373176_at	Transcribed locus	---	1.4	0.00223173	0.017397503
1376057_at	---	---	1.7	0.002233904	0.017397503
1368842_at	transcription factor 4	Tcf4	2.1	0.002242049	0.017434087
1375723_at	Transcribed locus	---	1.9	0.002250546	0.017477325
1388943_at	chromatin accessibility complex 1 (predicted)	Chrac1_predicted	1.3	0.002251856	0.017479249
1374976_a_at	Sterol O-acyltransferase 1	Soat1	2.2	0.00225919	0.017500096
1373447_at	similar to HN1-like protein	RGD1305117	1.5	0.002267504	0.017542697
1388459_at	procollagen, type XVIII, alpha 1	Col18a1	2.3	0.002268563	0.017542697
1380265_at	small nuclear RNA activating complex, polypeptide 1 (predicted)	Snapc1_predicted	2.0	0.00227713	0.017600676
1376711_at	claudin 11	Cldn11	3.4	0.002302227	0.01773631
1367969_at	peroxiredoxin 6	Prdx6	1.6	0.002310789	0.017768985
1371944_at	ubiquitin-conjugating enzyme E2L 3 (predicted)	Ube2l3_predicted	1.8	0.002315147	0.017777589
1372352_at	arginine-rich, mutated in early stage tumors (predicted)	Armet_predicted	1.5	0.002318434	0.017794498
1372004_at	heme binding protein 1 (predicted)	Hebp1_predicted	1.8	0.00232282	0.017796757
1391608_at	similar to Parn protein (predicted)	RGD1565449_predicted	1.4	0.002323057	0.017796757
1398464_at	Transcribed locus	---	1.8	0.002324851	0.01780221
1376200_at	hypothetical LOC316976	MGC72974	1.5	0.002333135	0.017844708
1386868_at	ribosomal protein S10	Rps10	1.4	0.002335907	0.017845331
1375849_at	RGM domain family, member A (predicted)	Rgma_predicted	1.7	0.002338275	0.017855131
1383601_at	Transcribed locus	---	1.8	0.002346752	0.017905352
1372333_at	similar to small nuclear ribonucleoprotein E	LOC685587 /// LOC687315	1.4	0.002347793	0.017905352
1376621_at	similar to myocardial ischemic preconditioning upregulated protein 2 (predicted)	RGD1565589_predicted	1.8	0.002350724	0.017916915
1378633_at	leupaxin	Lpxn	1.6	0.00235297	0.017917431
1383578_at	Transcribed locus	---	2.0	0.002360484	0.017957526
1376627_at	Transcribed locus	---	1.8	0.002361511	0.017957526
1386102_at	---	---	1.6	0.002379222	0.018067148
1380544_at	Transcribed locus	---	2.5	0.002401566	0.018216879
1391067_at	---	---	2.4	0.002405478	0.018232857
1390532_at	Transcribed locus	---	1.9	0.002409748	0.01825681
1391003_at	similar to chromosome 10 open reading frame 6 (predicted)	RGD1560300_predicted	1.7	0.002426583	0.018352467
1398919_at	similar to Hypothetical protein CGI-99	RGD1304704	1.6	0.002426837	0.018352467
1374838_at	SP140 nuclear body protein	Sp140	1.5	0.002437269	0.018414426
1382630_at	Similar to 4930506M07Rik protein (predicted)	RGD1311558_predicted	2.2	0.002451416	0.018504306
1383476_at	Inter-alpha trypsin inhibitor, heavy chain 3	Itih3	1.9	0.002459876	0.01855114
1382081_at	Transcribed locus	---	1.7	0.002466976	0.018596158
1389474_at	Myosin regulatory light chain interacting protein (predicted)	Mylip_predicted	2.0	0.00248603	0.018714059
1392569_at	Insulin-like growth factor 1	Igf1	1.9	0.00248999	0.018727628
1371515_at	similar to elongation factor 1 homolog (ELF1, S. cerevisiae)	LOC686668 /// LOC691193	1.3	0.00249011	0.018727628
1389756_at	maternal embryonic leucine zipper kinase (predicted)	Melk_predicted	2.1	0.002501179	0.018782553
1379669_at	---	---	1.4	0.002501217	0.018782553
1398885_at	ribosomal protein L23	Rpl23	1.4	0.002505675	0.018801709
1390846_at	procollagen, type XVI, alpha 1	Col16a1	1.9	0.00250993	0.018816466
1372254_at	serine (or cysteine) peptidase inhibitor, clade G, member 1	Serping1	2.9	0.002531925	0.018946813
1393730_at	Transcribed locus	---	1.9	0.002540312	0.018979133
1373475_at	coiled-coil domain containing 58 (predicted)	Ccdc58_predicted	1.6	0.002540543	0.018979133

1382847_at	ash1 (absent, small, or homeotic)-like (Drosophila) (predicted)	Ash1l_predicted	1.4	0.002541444	0.018979133
1368418_a_at	ceruloplasmin	Cp	2.3	0.002550556	0.019034277
1374247_at	---	---	2.2	0.002554499	0.019055059
1372843_at	LOC363020 (predicted)	RGD1309410_predicted	1.6	0.002564439	0.019115667
1387930_at	regenerating islet-derived 3 alpha	Reg3a	2.2	0.002564949	0.019115667
1384391_at	retinol dehydrogenase 10 (all-trans)	Rdh10	3.3	0.002566738	0.019120337
1371969_at	Transcribed locus	---	1.4	0.002572196	0.01915232
1374487_at	similar to RIKEN cDNA 5730536A07	RGD1307481	1.5	0.002581578	0.019198918
1376920_at	similar to sterile alpha motif domain containing 9-like	LOC500013	1.9	0.002582356	0.019198918
1374016_at	endothelial differentiation, lysophosphatidic acid G-protein-coupled receptor, 2	Edg2	1.6	0.002583091	0.019198918
1374751_at	---	---	1.4	0.00258429	0.019198918
1381099_at	V-raf murine sarcoma 3611 viral oncogene homolog	Araf	1.9	0.002587469	0.019213857
1375056_at	---	---	1.5	0.002588638	0.01921386
1368681_at	parathyroid hormone-like peptide	Pthlh	1.6	0.002590896	0.019221947
1392425_x_at	LUC7-like (S. cerevisiae)	Luc7l	1.4	0.002594811	0.019231443
1368221_at	nuclear receptor subfamily 3, group C, member 1	Nr3c1	1.5	0.002601521	0.019266017
1382255_at	---	---	1.3	0.002619217	0.019362192
1377029_at	RAR-related orphan receptor alpha (predicted)	Rora_predicted	1.5	0.002621591	0.019371041
1398387_at	hypothetical LOC310540	MGC72614	2.3	0.002646451	0.019519651
1379638_at	Transcribed locus	---	2.1	0.00265528	0.019544829
1379245_at	Thioredoxin domain containing 9	Txndc9	1.7	0.002657097	0.019544829
1385252_at	tripartite motif protein 34 (predicted)	Trim34_predicted	2.1	0.002668582	0.019569344
1385849_at	Transcribed locus	---	1.6	0.002669912	0.019569836
1392894_at	fibrinogen-like 2	Fgl2	1.5	0.002675727	0.019594998
1377345_at	Bobby sox homolog (Drosophila) (predicted)	Bbx_predicted	1.5	0.002680692	0.01962262
1390021_at	histone cluster 1, H2bh	Hist1h2bh	1.5	0.002685969	0.019635032
1376947_at	retinoblastoma binding protein 6	Rbbp6	1.9	0.002694952	0.019674463
1385291_a_at	---	---	3.3	0.002699964	0.019702312
1388420_at	similar to topoisomerase (DNA) II beta	LOC361100	1.6	0.002704554	0.019727056
1382633_at	Transcribed locus	---	2.2	0.002726531	0.019808298
1377212_at	Similar to Serologically defined colon cancer antigen 13 (predicted)	RGD1564816_predicted	2.1	0.002731299	0.019816675
1377380_at	---	---	1.4	0.00273391	0.019826877
1383001_at	RGD1560812 (predicted)	RGD1560812_predicted	1.7	0.002738213	0.019849329
1371875_at	mannosidase, beta A, lysosomal	Manba	1.5	0.002743991	0.019875482
1383129_at	CG6210-like	LOC362065	1.6	0.002745169	0.019875482
1372062_at	similar to cyclin-dependent kinase 2-interacting protein (predicted)	RGD1563395_predicted	1.5	0.002750704	0.019896035
1390382_at	similar to RIKEN cDNA 2310003F16 (predicted)	RGD1311457_predicted	1.9	0.002754405	0.019914047
1384815_at	Protein phosphatase 3, catalytic subunit, alpha isoform	Ppp3ca	2.0	0.002761835	0.019955885
1381838_at	Transcribed locus	---	1.5	0.002766488	0.019975062
1398553_at	transmembrane emp24 protein transport domain containing 5	Tmed5	2.5	0.002772344	0.019980855
1371477_at	Transcribed locus	---	1.4	0.0027727	0.019980855
1397335_at	sema domain, immunoglobulin domain (Ig), short basic domain, secreted, (semaphorin) 3D	Sema3d	2.1	0.002773283	0.019980855
1388805_at	Protein phosphatase 2 (formerly 2A), catalytic subunit, alpha isoform	Ppp2ca	1.6	0.002776773	0.019996654
1384407_at	similar to RIKEN cDNA 1110025F24 (predicted)	RGD1311451_predicted	1.3	0.002784708	0.020014078
1377597_at	AT rich interactive domain 2 (Arid-rfx like) (predicted)	Arid2_predicted	1.5	0.002785276	0.020014078
1396532_at	Transcribed locus	---	1.6	0.002788404	0.020023475
1385660_at	similar to KIAA2010 protein (predicted)	RGD1309059_predicted	1.6	0.002790188	0.020023475
1368105_at	tetraspanin 2	Tspan2	1.5	0.002790334	0.020023475

1385472_at	Transcribed locus	---	1.8	0.002791969	0.020023475
1388709_at	similar to WD-repeat protein 43	LOC362703	1.3	0.002798516	0.02005664
1376839_at	Transcribed locus	---	1.5	0.002803497	0.020074842
1398258_at	apolipoprotein D	Apod	3.2	0.002816038	0.02015587
1383632_at	Transcribed locus	---	2.1	0.002831269	0.020256073
1392504_at	similar to RIKEN cDNA 1600012F09 (predicted)	RGD1306613_predicted	1.5	0.002838922	0.020301997
1389510_at	Ly1 antibody reactive clone	Lyar	1.6	0.002846791	0.02034655
1383953_at	excision repair cross-complementing rodent repair deficiency, complementation group 5	Ercc5	2.0	0.002847626	0.02034655
1376201_at	similar to Hypothetical UPF0049 protein ZK1128.2 in chromosome III	LOC360568	1.4	0.002875744	0.020485162
1373408_at	tubulin cofactor a	Tbca	1.6	0.002891525	0.020570847
1377653_at	similar to mKIAA1011 protein	LOC366669	2.2	0.002896643	0.020585101
1370340_x_at	tropomyosin 3, gamma	Tpm3	1.5	0.002896648	0.020585101
1379390_at	ST6 (alpha-N-acetyl-neuraminyl-2,3-beta-galactosyl-1,3)-N-acetylgalactosaminide alpha-2,6-sialyltransferase 2	St6galnac2	1.9	0.002897283	0.020585101
1376634_a_at	similar to Protein C12orf11 (Sarcoma antigen NY-SAR-95)	LOC686480 /// LOC690728	1.6	0.002900425	0.020598526
1379404_at	Transcribed locus	---	2.6	0.002915068	0.020666823
1378202_at	Transcribed locus	---	1.7	0.002951593	0.020880778
1382903_at	similar to zinc finger, ZZ domain containing 3	LOC310958	1.4	0.002964356	0.020960609
1372314_at	Transcribed locus	---	1.4	0.002965427	0.020960609
1388353_at	proliferation-associated 2G4	Pa2g4	1.7	0.002969325	0.020979145
1373950_at	zinc finger protein 496 (predicted)	Zfp496_predicted	1.7	0.002976223	0.021018858
1377827_at	serum response factor binding protein 1	Srfbp1	1.3	0.002993203	0.021129699
1398837_at	transcription elongation factor B (SIII), polypeptide 2	Tceb2	1.4	0.002995941	0.021130037
1379914_at	transcription factor CP2-like 2	Tcfcp2l2	1.7	0.003000904	0.021130037
1372945_at	inhibitor of growth family, member 3	Ing3	1.5	0.003006669	0.021152135
1374261_at	Transcribed locus	---	1.7	0.003011679	0.021178321
1370063_at	nuclear receptor subfamily 2, group F, member 2	Nr2f2	2.4	0.003014631	0.021190021
1398620_at	Transcribed locus	---	2.3	0.003016829	0.021196411
1374193_at	Transcribed locus	---	2.2	0.003023262	0.021232539
1393239_at	Mitochondrial fission regulator 1 (predicted)	Mtfr1_predicted	1.6	0.003028292	0.021234478
1389790_at	Transcribed locus	---	2.3	0.003030283	0.021236509
1391884_at	coagulation factor XII (Hageman factor)	F12	1.4	0.003047164	0.021312877
1373596_at	similar to hypothetical protein FLJ31737	RGD1310423	1.6	0.003050178	0.021321433
1392044_at	Transcribed locus	---	2.1	0.003059747	0.021369369
1368382_at	S100 calcium binding protein A3	S100a3	2.0	0.003060934	0.021369369
1389980_at	similar to Protein HSPC163 (predicted)	RGD1559740_predicted	2.1	0.003070277	0.021415848
1398849_at	H3 histone, family 3B	H3f3b	1.5	0.003088843	0.021502283
1376005_at	Kinesin family member 1B	Kif1b	1.5	0.003089439	0.021502283
1375638_at	serum deprivation response protein	Sdpr	2.0	0.003089888	0.021502283
1382468_at	similar to CG9752-PA	RGD1311345	1.4	0.003091642	0.021502283
1376260_at	similar to KIAA1627 protein (predicted)	RGD1304822_predicted	1.7	0.00309267	0.021502283
1378389_at	Transcribed locus, strongly similar to XP_225713.3 similar to nuclear factor of activated T-cells, cytoplasmic, calcineurin-dependent 1 [Rattus norvegicus]	---	2.4	0.003093045	0.021502283
1389617_at	ELK3, member of ETS oncogene family (predicted)	Elk3_predicted	2.1	0.003094555	0.02150369
1393608_at	Transcribed locus	---	1.3	0.003108689	0.021583672
1379376_at	Transcribed locus	---	2.1	0.003114119	0.021612251
1390480_at	Transcribed locus	---	1.4	0.003120845	0.021640669
1391218_at	similar to RIKEN cDNA 2810485I05 (predicted)	RGD1311077_predicted	1.5	0.003127134	0.021675139
1398441_at	non-catalytic region of tyrosine kinase adaptor protein 2 (predicted)	Nck2_predicted	1.8	0.003155892	0.021837654
1382603_at	Similar to PD-1-ligand precursor (predicted)	RGD1566211_predicted	1.7	0.003163528	0.021881289

1397341_at	PDZ binding kinase (predicted)	Pbk_predicted	2.7	0.00317821	0.021964371
1390450_a_at	osteoglycin (predicted)	Ogn_predicted	2.5	0.003198983	0.022080095
1373595_at	transmembrane protein 43	Tmem43	1.7	0.003218239	0.022166498
1375632_at	similar to 60S ribosomal protein L38 (predicted) /// similar to 60S ribosomal protein L38 /// ribosomal protein L38 /// 60S ribosomal protein L38 pseudogene	LOC681221 /// LOC681502 /// LOC682065 /// LOC682793 /// LOC682969 /// LOC685963 /// LOC686435 /// LOC688248 /// LOC689671 /// LOC690468 /// LOC690833 /// RGD1561636_predicted /// Rpl38	1.3	0.003221628	0.022179565
1376906_at	Transcribed locus	---	1.6	0.00322493	0.022184716
1383408_at	Transcribed locus	---	1.6	0.003248143	0.022282817
1391550_at	Transcribed locus	---	1.6	0.003251845	0.022282817
1382865_at	testis specific, 14	Tsga14	1.6	0.003259062	0.022307553
1371298_at	CDNA clone IMAGE:7460165	---	3.4	0.003262985	0.022325116
1383385_at	Transcribed locus	---	1.8	0.003272547	0.022371259
1371965_at	similar to RIKEN cDNA 2010311D03	RGD1303272	1.4	0.00327381	0.022371259
1368256_at	serine (or cysteine) peptidase inhibitor, clade I, member 1	Serpini1	1.7	0.003275411	0.022371396
1393017_at	rhophilin, Rho GTPase binding protein 1 (predicted)	Rhpn1_predicted	1.6	0.003300987	0.02248255
1373818_at	Potassium channel tetramerisation domain containing 12 (predicted)	Kctd12_predicted	1.4	0.003311998	0.022519944
1388345_at	---	---	1.3	0.00331524	0.022532671
1378362_at	similar to Complement C1q-like protein 3 precursor (Gliacolin)	LOC680404 /// LOC684921	2.7	0.003324073	0.022574052
1398405_at	similar to septin 6	LOC691335	1.5	0.003327837	0.02257564
1375853_at	similar to CG13957-PA (predicted)	RGD1309995_predicted	1.3	0.00333873	0.02260825
1388328_at	similar to Eukaryotic translation initiation factor 3 subunit 2 (eIF-3 beta) (eIF3 p36) (eIF3i) (TGF-beta receptor-interacting protein 1) (TRIP-1)	LOC682390	1.3	0.003340216	0.022608999
1380148_at	Transcribed locus	---	1.5	0.003344018	0.022625428
1375912_a_at	UDP-N-acetyl-alpha-D-galactosamine:polypeptide N-acetylgalactosaminyltransferase 2 (predicted)	Galnt2_predicted	1.9	0.003363515	0.02271539
1390112_at	epidermal growth factor-containing fibulin-like extracellular matrix protein 1	Efemp1	1.5	0.003368312	0.02271539
1391994_at	Transcribed locus	---	1.5	0.003369413	0.02271539
1377990_at	Transcribed locus	---	2.1	0.003369744	0.02271539
1373970_at	similar to RIKEN cDNA 9230117N10	RGD1311155	1.9	0.003371587	0.022718504
1384330_at	similar to hypothetical protein FLJ20546	LOC303067	1.5	0.003377077	0.022721768
1370526_at	integrin, alpha E, epithelial-associated	Itgae	1.8	0.003377451	0.022721768
1382109_at	nuclear NF-kappaB activating protein	2610020o08rik	1.3	0.003379445	0.022721768
1372360_at	---	---	1.4	0.003379534	0.022721768
1399125_at	inositol polyphosphate-1-phosphatase	Inpp1	1.4	0.00338036	0.022721768
1382275_at	similar to PAK/PLC-interacting protein 1	MGC125015	1.6	0.003390396	0.022761318
1372543_at	similar to RIKEN cDNA 2610029G23 (predicted)	RGD1562502_predicted	2.4	0.003417887	0.022908475
1371119_at	MHC class I RT1.O type 149 processed pseudogene	LOC360231	1.5	0.003437604	0.023016353
1382614_at	similar to Zinc finger, FYVE domain containing 16 (predicted)	RGD1564784_predicted	1.5	0.00343818	0.023016353
1386145_at	---	---	1.5	0.003443271	0.023041061
1378296_at	similar to Condensin complex subunit 2 (Barren homolog protein 1) (Chromosome-associated protein H) (mCAP-H) (XCAP-H homolog)	LOC680089 /// LOC688088	1.5	0.00346283	0.023162515
1372918_at	---	---	1.7	0.003464536	0.023164507
1376836_at	---	---	1.9	0.003468444	0.023181212

1383250_at	UTP14, U3 small nucleolar ribonucleoprotein, homolog A (yeast)	Utp14a	1.5	0.00347102	0.02318901
1368195_at	Hspb associated protein 1	Hspbap1	1.4	0.00347537	0.023208644
1370916_at	tec protein tyrosine kinase	Tec	1.6	0.003489589	0.023275246
1393020_at	---	---	1.6	0.003493835	0.02329412
1386477_at	Transcribed locus	---	1.6	0.003497677	0.023305989
1392541_at	similar to RIKEN cDNA A030007L17; EST AA673177 (predicted) /// similar to C44B7.7	LOC685702 /// RGD1304876_predicted	1.8	0.003524132	0.023420174
1386979_at	developmentally regulated protein TPO1	Tpo1	1.6	0.003526175	0.023424184
1389483_at	Dpy-19-like 1 (C. elegans) (predicted)	Dpy19l1_predicted	1.6	0.003527584	0.023424184
1387644_at	betacellulin	Btc	1.5	0.003559193	0.023586925
1377787_at	similar to Rbm6 protein (predicted)	RGD1560367_predicted	1.5	0.003560427	0.023586925
1367602_at	Cbp/p300-interacting transactivator, with Glu/Asp-rich carboxy-terminal domain, 2	Cited2	2.8	0.003561077	0.023586925
1374942_at	carboxypeptidase X 2 (M14 family) (predicted)	Cpxm2_predicted	1.7	0.003564998	0.023586925
1373670_at	Signal transducer and activator of transcription 2	Stat2	1.6	0.003575846	0.023620688
1382904_at	similar to hypothetical protein DKFZp434K1421	RGD1309863	1.6	0.003594236	0.023705986
1387854_at	procollagen, type I, alpha 2	Col1a2	1.7	0.003598018	0.023719519
1377611_at	Transcribed locus	---	1.7	0.003613667	0.02377472
1367936_at	serine/threonine kinase 10	Stk10	1.6	0.003615442	0.02377472
1370219_at	cytochrome b-245, alpha polypeptide	Cyba	1.7	0.003615548	0.02377472
1398549_at	---	---	1.4	0.003615948	0.02377472
1387080_at	chondroitin sulfate proteoglycan 6	Cspg6	1.4	0.00361651	0.02377472
1378536_at	Transcribed locus	---	1.4	0.003636033	0.023874438
1373159_at	Transcribed locus	---	1.7	0.003645664	0.023920789
1372297_at	glutathione S-transferase, alpha 4	Gsta4	1.7	0.003650515	0.023920789
1372101_at	phosphatidic acid phosphatase type 2B	Ppap2b	1.4	0.003650547	0.023920789
1373674_at	microfibrillar associated protein 5 (predicted)	Mfap5_predicted	2.1	0.003660661	0.02395963
1385238_at	Transcribed locus	---	1.3	0.003674807	0.024033091
1383095_at	Transcribed locus	---	1.6	0.003682293	0.024072475
1372019_at	similar to DNA segment, Chr 13, Wayne State University 50, expressed (predicted)	RGD1310128_predicted	1.4	0.003715607	0.024190595
1369113_at	gremlin 1 homolog, cysteine knot superfamily (Xenopus laevis)	Grem1	1.5	0.003715792	0.024190595
1390933_a_at	RNA (guanine-9-) methyltransferase domain containing 3	Rg9mtd3	1.8	0.003733484	0.02428167
1384451_at	Transcribed locus	---	1.6	0.003737561	0.02429858
1368824_at	caldesmon 1	Cald1	1.8	0.003742481	0.024317716
1378395_at	Transcribed locus	---	1.3	0.00374494	0.024317716
1380206_at	Kinesin family member 5C (predicted)	Kif5c_predicted	1.4	0.003758126	0.024391345
1390574_at	Transcribed locus	---	1.4	0.003759245	0.024391345
1389394_at	Transcribed locus	---	1.6	0.003762594	0.024403447
1390093_at	Similar to Ribulose-5-phosphate-3-epimerase	MGC124653	1.6	0.003783366	0.024509178
1393972_at	metal response element binding transcription factor 2	Mtf2	1.4	0.003811491	0.024652538
1378740_at	RAS protein activator like 2 (predicted)	Rasal2_predicted	2.1	0.003813382	0.024655073
1377516_at	Odd Oz/ten-m homolog 2 (Drosophila)	Odz2	2.2	0.003818527	0.024668945
1367769_at	polymerase (RNA) II (DNA directed) polypeptide G	Polr2g	1.4	0.003820469	0.024671797
1379450_at	CTTNBP2 N-terminal like (predicted)	Cttnbp2nl_predicted	1.5	0.003843565	0.024773335
1377595_at	sperm specific antigen 2 (predicted)	Ssfa2_predicted	1.3	0.003845229	0.024773335
1373869_at	sterol O-acyltransferase 1	Soat1	1.6	0.003849835	0.024788306
1395316_at	melanoma antigen, family H, 1	Mageh1	1.6	0.003852175	0.024788306
1378843_at	DnaJ (Hsp40) homolog, subfamily B, member 6	Dnajb6	2.1	0.003858101	0.024788306
1376483_at	LSM5 homolog, U6 small nuclear RNA associated (S. cerevisiae) (predicted)	Lsm5_predicted	2.0	0.003859609	0.024788306
1371862_at	ribonucleotide reductase M1	Rrm1	1.5	0.003863838	0.02479751
1368589_at	protein tyrosine phosphatase, receptor type, J	Ptprj	1.7	0.003876299	0.024817968
1382746_s_at	Alpha-kinase 1 (predicted)	Alpk1_predicted	1.5	0.003881474	0.024835097

1389188_at	G protein-coupled receptor 108	Gpr108	1.4	0.003881994	0.024835097
1372286_at	tetraspanin 6	Tspan6	1.4	0.003898334	0.024910565
1383531_at	---	---	1.4	0.003903714	0.024925577
1367509_at	similar to general transcription factor IIH, polypeptide 5 (predicted)	RGD1560991_predicted	1.4	0.00391065	0.024960177
1388015_at	protein tyrosine phosphatase, receptor-type, Z polypeptide 1	Ptprz1	1.4	0.003918169	0.024980262
1389204_at	---	---	1.7	0.003918353	0.024980262
1371130_at	solute carrier family 1 (glial high affinity glutamate transporter), member 3	Slc1a3	2.2	0.00393915	0.025073969
1383339_at	similar to NNX3 (predicted)	RGD1310358_predicted	1.6	0.003947158	0.025090615
1389918_at	---	---	2.3	0.003947867	0.025090615
1368422_at	mesenchyme homeobox 2	Meox2	2.9	0.003959473	0.025144948
1397387_at	Transcribed locus	---	1.4	0.003984066	0.025271851
1396383_at	aldehyde dehydrogenase 3 family, member B1	Aldh3b1	1.4	0.003990293	0.025293855
1394925_at	similar to RIKEN cDNA 2310035C23 (predicted)	RGD1307235_predicted	1.6	0.00399061	0.025293855
1398386_at	Similar to Zinc finger CCHC domain-containing protein 6	LOC501515	1.3	0.004004535	0.025333308
1373905_at	heterogeneous nuclear ribonucleoprotein R	Hnrpr	1.4	0.004013791	0.025382095
1383644_at	Transcribed locus	---	2.2	0.004015389	0.025382442
1376101_at	low density lipoprotein receptor-related protein 6 (predicted)	Lrp6_predicted	1.4	0.004021975	0.025406468
1389372_at	similar to hypothetical protein (predicted)	RGD1308489_predicted	1.4	0.004022279	0.025406468
1393336_at	SWAP complex protein (predicted)	Swap70_predicted	1.4	0.004024268	0.025409274
1388939_at	procollagen, type XV	Col15a1	1.9	0.004029928	0.025426364
1377728_at	RGD1565641 (predicted)	RGD1565641_predicted	2.2	0.004031811	0.025427618
1372414_at	Transcribed locus	---	1.4	0.004037025	0.025450743
1377072_at	Transcribed locus, strongly similar to XP_001077488.1 similar to ankyrin repeat domain 25 [Rattus norvegicus]	---	1.8	0.004047246	0.025498764
1372877_at	procollagen-lysine, 2-oxoglutarate 5-dioxygenase 3	Plod3	1.4	0.004075643	0.025654869
1369633_at	chemokine (C-X-C motif) ligand 12	Cxcl12	1.8	0.004086538	0.025713607
1375205_at	p300/CBP-associated factor	Pcaf	1.6	0.004090636	0.02572955
1384392_at	cytochrome P450, family 26, subfamily b, polypeptide 1	Cyp26b1	2.6	0.004113795	0.025855445
1392653_at	Transcribed locus	---	1.9	0.004151053	0.026029936
1380594_at	signal peptide, CUB domain, EGF-like 1	Scube1	1.4	0.00416521	0.026100051
1395130_at	---	---	2.1	0.00417207	0.026113384
1389636_at	similar to KIAA0833 protein /// similar to calmodulin-binding transcription activator 1	LOC362665 /// LOC500591	1.7	0.004173887	0.026113384
1377992_at	Dual specificity phosphatase 7	Dusp7	2.8	0.004187415	0.026170965
1374689_at	phosphatidylinositol 4-kinase, catalytic, beta polypeptide	Pik4cb	1.3	0.004189355	0.026170965
1387300_at	Crn, crooked neck-like 1 (Drosophila)	Crnkl1	1.5	0.004189455	0.026170965
1396323_at	similar to Protein C10orf11 homolog	LOC681383	1.4	0.004198498	0.0262175
1369161_at	ATP-binding cassette, sub-family B (MDR/TAP), member 4	Abcb4	1.4	0.004205732	0.026242748
1373310_at	Transcribed locus	---	1.4	0.004208877	0.02625241
1382509_at	similar to CCR4	RGD1310783	1.6	0.004215747	0.02626538
1378291_at	FK506 binding protein 3 (predicted)	Fkbp3_predicted	1.4	0.004261096	0.026507756
1378133_at	Transcribed locus	---	1.9	0.004268968	0.026536651
1373413_at	similar to RIKEN cDNA 2810021O14 (predicted)	RGD1310481_predicted	1.4	0.004270806	0.026537094
1370959_at	procollagen, type III, alpha 1	Col3a1	1.8	0.004277392	0.026548885
1375553_at	similar to RIKEN cDNA 5133400G04	RGD1311742	1.4	0.004300551	0.0266725
1378038_at	Polypyrimidine tract binding protein 2	Ptbp2	2.9	0.0043119	0.026732806
1380926_at	Transcribed locus	---	1.8	0.004341647	0.026846393
1381259_at	stromal antigen 1 (predicted)	Stag1_predicted	1.5	0.004344886	0.026856329
1374208_at	zinc finger CCCH type containing 7 A (predicted)	Zc3h7a_predicted	1.5	0.004347651	0.026861534

1389358_at	Similar to lysophosphatidylglycerol acyltransferase 1	LOC679692	1.7	0.004352443	0.026869213
1370190_at	H3 histone, family 3B	H3f3b	1.5	0.004353505	0.026869213
1383705_at	CDNA clone IMAGE:7317308	---	1.5	0.004378221	0.027002346
1398756_at	nucleophosmin 1 /// similar to Nucleophosmin (NPM) (Nucleolar phosphoprotein B23) (Numatrin) (Nucleolar protein NO38)	LOC300303 /// Npm1	1.4	0.004381533	0.027002346
1390171_at	similar to hypothetical protein BC008163 (predicted)	RGD1305162_predicted	1.4	0.004381571	0.027002346
1382370_at	cyclin F	Ccnf	1.3	0.004381643	0.027002346
1383902_at	leucine-rich repeats and IQ motif containing 2 (predicted)	Lrriq2_predicted	1.4	0.004395541	0.02704502
1372647_at	---	---	1.9	0.004397329	0.02704502
1389208_at	---	---	1.7	0.004398433	0.02704502
1383334_at	Transcribed locus	---	1.4	0.004447283	0.027296144
1371316_at	Finkel-Biskis-Reilly murine sarcoma virus (FBR-MuSV) ubiquitously expressed (fox derived) protein	Fau	1.4	0.004451654	0.027300819
1394814_at	translocated promoter region	Tpr	2.3	0.004453927	0.027304582
1383894_at	Rap guanine nucleotide exchange factor (GEF) 6 (predicted)	Rapgef6_predicted	1.8	0.004457355	0.027313439
1398356_at	cleavage and polyadenylation specific factor 5	Cpsf5	1.3	0.004458693	0.027313439
1393451_at	similar to RIKEN cDNA 2610510J17	RGD1310953	1.4	0.004472672	0.027360564
1371512_at	---	---	1.4	0.00447304	0.027360564
1390931_at	Transcribed locus	---	1.4	0.004485026	0.027410884
1377939_at	Similar to APG4 autophagy 4 homolog C	LOC313391	1.4	0.004491265	0.027410884
1378074_at	Transcribed locus	---	1.5	0.004498116	0.027425227
1398839_at	thioredoxin 1	Txn1	1.3	0.004498618	0.027425227
1382154_at	Transcribed locus, moderately similar to XP_519168.2 protein tyrosine phosphatase, non-receptor type 12 [Pan troglodytes]	---	1.5	0.004507842	0.027461108
1382058_at	related RAS viral (r-ras) oncogene homolog 2	Rras2	2.0	0.004517904	0.027502031
1372778_at	Transcribed locus	---	1.9	0.004521754	0.027515285
1393365_at	chromodomain helicase DNA binding protein 1 (predicted)	Chd1_predicted	1.7	0.004535327	0.027587668
1375841_at	Transcribed locus	---	1.5	0.004538074	0.027590404
1387018_at	Arg/Abl-interacting protein ArgBP2	Argbp2	1.5	0.004541761	0.027596189
1369970_at	vesicle-associated membrane protein 8	Vamp8	1.5	0.004548855	0.02761779
1376159_at	similar to zinc finger protein 336 (predicted)	RGD1562321_predicted	1.5	0.004553936	0.027619152
1385088_at	hypothetical LOC304650 (predicted)	RGD1310262_predicted	1.8	0.004579477	0.027739051
1375504_at	polymerase (DNA directed), gamma 2, accessory subunit (predicted)	Polg2_predicted	1.3	0.004580451	0.027739051
1389299_at	---	---	1.3	0.004604349	0.027839625
1367696_at	interferon induced transmembrane protein 2 (1-8D)	Ifitm2	1.5	0.004605282	0.027839625
1380908_at	Cd200 antigen	Cd200	1.6	0.004607181	0.027839625
1391759_at	SH3-domain GRB2-like B1 (endophilin)	Sh3glb1	1.6	0.004607214	0.027839625
1390514_at	similar to hypothetical protein from BCRA2 region	MGC94223	1.3	0.004622874	0.027893263
1377806_at	---	---	1.6	0.004629889	0.027914124
1388650_at	topoisomerase (DNA) 2 alpha	Top2a	2.3	0.004631423	0.027914124
1374428_at	kinesin family member 3B (predicted)	Kif3b_predicted	1.4	0.00463555	0.027921888
1372481_at	Transcribed locus, strongly similar to XP_223083.3 similar to CD34 antigen [Rattus norvegicus]	---	1.5	0.004636106	0.027921888
1377369_at	cytochrome b reductase 1	Cybrd1	1.3	0.004638019	0.027923182
1393779_x_at	---	---	2.4	0.004644861	0.027954145
1373868_at	---	---	1.5	0.004666696	0.028023421
1381117_at	Ribosomal protein S5	Rps5	1.4	0.004674479	0.028050287
1383949_at	thyroid hormone receptor associated protein 2 (predicted)	Thrap2_predicted	1.5	0.004685413	0.028085946

1388792_at	growth arrest and DNA-damage-inducible 45 gamma	Gadd45g	2.2	0.004687994	0.028085946
1385595_at	similar to antigenic determinant of rec-A protein	LOC683353 /// LOC689197	1.6	0.004689965	0.028085946
1384262_at	protein phosphatase 1, regulatory (inhibitor) subunit 3B	Ppp1r3b	1.5	0.004719753	0.028229273
1375061_at	Transcribed locus	---	1.4	0.004723595	0.028231717
1380088_at	Transcribed locus	---	1.8	0.004729281	0.028247074
1372558_at	NMDA receptor-regulated gene 1 (predicted)	Narg1_predicted	1.3	0.004732669	0.028247074
1372081_at	Transcribed locus	---	1.3	0.004732779	0.028247074
1392915_at	procollagen, type XI, alpha 1	Col11a1	1.7	0.004733033	0.028247074
1392723_at	Similar to ankyrin repeat domain protein 17 isoform b (predicted)	RGD1562348_predicted	1.3	0.004737747	0.028264946
1378574_at	CDNA clone MGC:105770 IMAGE:7319366	---	1.8	0.004742132	0.028280852
1374411_at	mitochondrial ribosomal protein L52 (predicted)	Mrpl52_predicted	1.8	0.004751495	0.028316154
1391023_at	Transcribed locus	---	1.4	0.0047626	0.028341259
1381525_at	---	---	1.3	0.004766195	0.028346613
1372750_at	Transcribed locus	---	2.7	0.004769899	0.028353921
1391871_at	Transcribed locus	---	1.8	0.004772695	0.028355541
1376324_at	Transcribed locus, weakly similar to NP_055333.2 finger protein 229 [Homo sapiens]	---	1.6	0.004773619	0.028355541
1383152_at	similar to Cc1-9	RGD1308955	1.4	0.004783663	0.028388205
1389569_at	brix domain containing 2	Bxdc2	1.5	0.004784296	0.028388205
1373600_at	Similar to CG9996-PA	LOC300173	1.9	0.004794676	0.028439536
1374427_at	Synaptotagmin XIII	Syt13	1.7	0.004798767	0.02844338
1385608_at	Transcribed locus	---	1.7	0.004798783	0.02844338
1368304_at	flavin containing monooxygenase 3	Fmo3	2.6	0.004813283	0.028498515
1382305_at	---	---	3.0	0.004834928	0.028595789
1389281_at	Transcribed locus, weakly similar to XP_001055725.1 similar to ankyrin repeat domain 15 [Rattus norvegicus]	---	1.6	0.004868467	0.028732164
1369712_at	serine/threonine kinase 3 (STE20 homolog, yeast)	Stk3	1.5	0.004879789	0.02876802
1395561_at	heterogeneous nuclear ribonucleoprotein A2/B1 (predicted)	Hnrpa2b1_predicted	2.7	0.004882717	0.028774966
1371521_at	similar to Paired amphipathic helix protein Sin3b (Transcriptional corepressor Sin3b) (Histone deacetylase complex subunit Sin3b)	LOC683381	1.4	0.004886002	0.028775782
1382238_at	similar to cell division cycle and apoptosis regulator 1 (predicted)	RGD1560358_predicted	1.6	0.004895644	0.028820171
1373777_at	Transcribed locus	---	1.6	0.004905445	0.028865082
1397610_at	Transcribed locus	---	1.6	0.004910945	0.028868912
1381175_at	similar to modulator of estrogen induced transcription	RGD1307526	2.5	0.004917415	0.028896622
1391474_at	similar to cofactor required for Sp1 transcriptional activation, subunit 2, 150kDa (predicted)	RGD1560170_predicted	1.5	0.004934523	0.028955775
1382072_at	Similar to cis-Golgi matrix protein GM130	LOC690485	1.8	0.004945408	0.028998958
1374579_at	similar to RIKEN cDNA E230015L20 gene (predicted)	RGD1560873_predicted	1.7	0.004975198	0.029163242
1390722_at	---	---	2.0	0.004982131	0.029163972
1383336_at	similar to Pinin	LOC368070	1.7	0.004982415	0.029163972
1370383_s_at	RT1 class II, locus Db1	RT1-Db1	5.5	0.004987761	0.029174502
1383981_at	---	---	1.5	0.004991308	0.029184866
1375270_at	Transcribed locus	---	2.1	0.00499707	0.029208176
1388794_at	RNA binding motif protein, X chromosome retrogene (predicted) /// similar to Heterogeneous nuclear ribonucleoprotein G (hnRNP G) (RNA-binding motif protein, X chromosome)	LOC679457 /// LOC680944 /// Rbmxrt_predicted	1.5	0.005009345	0.029269523
1381016_at	Inositol 1,3,4,5,6-pentakisphosphate 2-kinase	Ippk	1.4	0.005014017	0.029286419
1385248_a_at	osteoglycin (predicted)	Ogn_predicted	2.5	0.005050114	0.029444975

1386097_at	---	---	1.7	0.005055327	0.029464928
1384240_at	angiotensin II receptor, type 1 (AT1A)	Agtr1a	1.4	0.005061369	0.029479248
1377321_at	Myeloid/lymphoid or mixed-lineage leukemia 5 (trithorax homolog, Drosophila)	Mll5	2.1	0.005069446	0.029496544
1379652_at	AT hook containing transcription factor 1 (predicted)	Ahctf1_predicted	1.4	0.005071741	0.029496544
1383829_at	bobby sox homolog (Drosophila) (predicted)	Bbx_predicted	1.4	0.005072936	0.029496544
1388368_at	mediator of RNA polymerase II transcription, subunit 28 homolog (yeast) (predicted)	Med28_predicted	1.8	0.005076814	0.029496544
1395372_at	Integrin beta 8 (predicted)	Itgb8_predicted	1.8	0.005078685	0.029496544
1392840_at	Transcribed locus	---	2.3	0.005091671	0.029561528
1388704_at	---	---	1.7	0.005099207	0.029591365
1393109_at	CDNA clone IMAGE:7302574	---	2.2	0.005124813	0.02971199
1377194_a_at	similar to RIKEN cDNA 2310015N07	RGD1308637	1.4	0.005132485	0.029735513
1393431_at	similar to Splicing factor U2AF 35 kDa subunit (U2 auxiliary factor 35 kDa subunit) (U2 snRNP auxiliary factor small subunit)	LOC687575	2.0	0.005142611	0.029783688
1374405_at	heterogeneous nuclear ribonucleoprotein A3	Hnrpa3	1.3	0.005149477	0.029812953
1398450_at	Transcribed locus	---	1.4	0.005152086	0.029817559
1373497_at	Transcribed locus	---	1.6	0.005166591	0.029880447
1397096_at	Similar to 4933407C03Rik protein (predicted)	RGD1306101_predicted	1.5	0.005174727	0.029906503
1390165_at	PR domain containing 15 (predicted)	Prdm15_predicted	1.7	0.005197574	0.030017449
1382521_at	glutaminase	Gls	1.8	0.005207924	0.030045576
1397508_at	DEAD (Asp-Glu-Ala-Asp) box polypeptide 18	Ddx18	1.8	0.005211228	0.030054097
1383687_at	Transcribed locus	---	1.8	0.00521939	0.030073361
1387306_a_at	early growth response 2	Egr2	1.7	0.005219396	0.030073361
1372946_at	similar to RIKEN cDNA 1110012L19 (predicted)	RGD1562747_predicted	1.4	0.005222034	0.030073361
1372389_at	immediate early response 2	Ier2	1.8	0.005223711	0.030073361
1372301_at	AE binding protein 1 (predicted)	Aebp1_predicted	1.9	0.005227121	0.030082464
1368145_at	Purkinje cell protein 4	Pcp4	1.5	0.005236576	0.030118075
1392465_at	sin3-associated polypeptide, 18kDa	Sap18	1.6	0.005270659	0.030265409
1388876_at	Transcribed locus	---	1.5	0.00527363	0.030265409
1382745_at	Alpha-kinase 1 (predicted)	Alpk1_predicted	1.7	0.005286622	0.030326594
1373979_at	Cadherin 23 (otocadherin)	Cdh23	1.4	0.005287979	0.030326594
1383296_a_at	exportin 4 (predicted)	Xpo4_predicted	1.7	0.005295509	0.030338045
1380445_at	---	---	1.8	0.005305764	0.03036636
1376594_at	vascular endothelial zinc finger 1 (predicted)	Vezf1_predicted	1.6	0.005308981	0.03036636
1378714_at	similar to hypothetical protein BC010003 (predicted)	RGD1309873_predicted	2.0	0.005314549	0.030373074
1387039_at	glypican 1	Gpc1	1.7	0.005329721	0.030428063
1371251_at	galactose-1-phosphate uridyl transferase	Galt	1.4	0.005337908	0.030464229
1376419_at	Similar to expressed sequence AI317237 (predicted)	RGD1305671_predicted	1.5	0.005343368	0.030484813
1389179_at	cell death-inducing DNA fragmentation factor, alpha subunit-like effector A (predicted)	Cidea_predicted	1.9	0.005355841	0.030534793
1370706_a_at	cytochrome P450, family 2, subfamily j, polypeptide 9	Cyp2j9	1.5	0.005363862	0.030559333
1373908_at	---	---	1.5	0.005371361	0.030591463
1379594_at	Abelson helper integration site 1	Ahi1	1.8	0.005375651	0.030605298
1381798_at	LIM domain only protein 7	LMO7	2.6	0.005400889	0.030727716
1379377_at	ganglioside-induced differentiation-associated-protein 2	Gdap2	1.4	0.005418699	0.030797084
1368518_at	CD53 antigen	Cd53	2.4	0.005425908	0.030816761
1377463_at	Mannosidase 2, alpha 2 (predicted)	Man2a2_predicted	1.4	0.005439334	0.030865912
1379995_at	Tubulin, beta 2b	Tubb2b	2.6	0.005440192	0.030865912
1389459_at	Similar to RIKEN cDNA 1600012F09 (predicted)	RGD1306613_predicted	1.3	0.005479043	0.031054206
1373738_at	similar to M phase phosphoprotein 6	LOC686999	1.4	0.005503394	0.031149284

1373574_at	similar to hypothetical protein MGC40499 (predicted)	RGD1307636_predicted	1.4	0.00550852	0.03116621
1398617_at	Transcribed locus, strongly similar to XP_001060577.1 similar to ribosomal protein S27a [Rattus norvegicus]	---	1.6	0.005521624	0.031188074
1373262_at	similar to 2310014H01Rik protein (predicted)	RGD1309543_predicted	1.7	0.005532344	0.031227175
1388149_at	transporter 1, ATP-binding cassette, sub-family B (MDR/TAP)	Tap1	1.6	0.00554369	0.031248323
1380824_at	hook homolog 3 (Drosophila)	Hook3	1.4	0.005546493	0.031253413
1376374_at	Transcribed locus	---	1.5	0.005555044	0.031290879
1370333_a_at	insulin-like growth factor 1	Igf1	1.9	0.005567933	0.031320576
1377501_at	similar to zinc finger protein 75 (predicted)	RGD1565419_predicted	1.4	0.005574013	0.031337489
1399142_at	Transcribed locus	---	1.4	0.005576185	0.031337489
1386641_at	Transcribed locus	---	2.8	0.005576656	0.031337489
1387414_at	dual oxidase 2	Duox2	1.7	0.005580397	0.031338792
1387002_at	maternal G10 transcript	G10	1.3	0.005582603	0.031338792
1383610_at	---	---	1.8	0.00558569	0.03134216
1375019_at	heterogeneous nuclear ribonucleoprotein H3 (2H9) (predicted)	Hnrph3_predicted	1.5	0.005587014	0.03134216
1389413_at	similar to ecotropic viral integration site 2A	LOC685433 /// LOC688044	2.1	0.005600554	0.031398667
1367905_at	ectonucleotide pyrophosphatase/phosphodiesterase 3	Enpp3	1.5	0.005607832	0.031409251
1395968_at	similar to Myosin-15 (Myosin XV) (Unconventional myosin-15)	LOC688264	1.5	0.005623759	0.031462445
1391635_at	CTD (carboxy-terminal domain, RNA polymerase II, polypeptide A) small phosphatase-like (predicted)	Ctdspl_predicted	1.5	0.005632248	0.031487121
1382101_at	heparan sulfate 2-O-sulfotransferase 1	Hs2st1	1.7	0.005633929	0.031487121
1376155_at	similar to hypothetical protein 2BE2121 - Chinese hamster (fragment) (predicted)	RGD1561110_predicted	1.3	0.005635827	0.031487121
1379282_at	leucine rich repeat (in FLII) interacting protein 2	Lrrfip2	1.5	0.005647458	0.031519981
1376864_at	similar to cell division cycle and apoptosis regulator 1 (predicted)	RGD1560358_predicted	2.2	0.00565171	0.031522324
1376687_at	ubiquitin specific peptdiase 1	Usp1	2.3	0.00566697	0.031584079
1368205_at	complement factor I	Cfi	1.6	0.005678207	0.031595111
1389380_at	similar to Folylpolyglutamate synthase, mitochondrial precursor (Folylpoly-gamma-glutamate synthetase) (FPGS) (Tetrahydrofolate synthase) (Tetrahydrofolylpolyglutamate synthase)	LOC687266	1.5	0.005687821	0.031616517
1372938_at	similar to RIKEN cDNA 6330509G02	RGD1304726	1.4	0.00570946	0.031720729
1398434_at	death associated transcription factor 1 (predicted)	Datf1_predicted	1.5	0.005727133	0.031792063
1381244_at	Cut-like 1 (Drosophila)	Cutl1	1.5	0.005731041	0.031803024
1372653_at	FK506 binding protein 11	Fkbp11	1.5	0.005754543	0.031884731
1398770_at	large subunit ribosomal protein L36a	Rpl36a	1.5	0.005765089	0.031927332
1371438_at	---	---	1.6	0.005774005	0.031955993
1382415_at	LOC360807	LOC360807	1.6	0.005789486	0.032008559
1370972_x_at	RT1 class I, CE5	RT1-CE5	1.6	0.005792578	0.032014891
1393224_at	Transcribed locus	---	1.4	0.005815851	0.032121934
1384452_at	zinc finger, CCHC domain containing 7 (predicted)	Zcchc7_predicted	2.6	0.005821433	0.032141723
1389546_at	---	---	1.6	0.005829124	0.032162844
1375526_at	similar to novel protein of unknown function (DUF423) family member (predicted)	RGD1563438_predicted	1.9	0.005893589	0.032464083
1389020_at	similar to immunoglobulin superfamily containing leucine-rich repeat	LOC686539	1.7	0.005899024	0.032472269
1374694_at	similar to Ankyrin repeat domain protein 28 (predicted)	RGD1559931_predicted	1.4	0.005906012	0.032498842
1383239_at	zinc finger, CCHC domain containing 7 (predicted)	Zcchc7_predicted	1.6	0.005908572	0.032498842
1397473_at	distrobrevin binding protein 1	Dtnbp1	1.6	0.005911106	0.032498842
1382005_at	Transcribed locus	---	1.3	0.005919601	0.032531103
1381584_at	Calsyntenin 2	Clstn2	1.8	0.005927608	0.032550322

1373676_at	Transcribed locus	---	1.5	0.005980361	0.032777398
1382330_at	development and differentiation enhancing factor 2 (predicted)	Ddef2_predicted	2.0	0.005995144	0.03280805
1372748_at	TBC1 domain family, member 2B	Tbc1d2b	1.3	0.005995926	0.03280805
1370822_at	RT1 class II, locus Ba	RT1-Ba	2.8	0.006000791	0.032822259
1370965_at	transcription factor 8	Tcf8	1.5	0.006002514	0.032822259
1379715_at	similar to CG9346-PA (predicted)	RGD1307882_predicted	1.6	0.006028363	0.032926481
1388439_at	similar to 65kDa FK506-binding protein	LOC360627	1.6	0.006029582	0.032926481
1394714_at	ATPase, Ca++ transporting, plasma membrane 1	Atp2b1	1.9	0.006038886	0.032961143
1378062_at	Rho guanine nucleotide exchange factor (GEF) 12	Arhgef12	1.7	0.006041385	0.032961143
1375378_at	similar to quaking homolog, KH domain RNA binding isoform HQK-6 /// similar to quaking homolog, KH domain RNA binding isoform HQK-5	LOC499022 /// LOC684079	1.6	0.006047101	0.032967372
1373200_at	eukaryotic translation elongation factor 1 epsilon 1 (predicted)	Eef1e1_predicted	1.4	0.006047398	0.032967372
1382695_at	Hypothetical protein LOC690329	LOC690329	1.7	0.006049096	0.032967372
1387666_at	G protein-coupled receptor 85	Gpr85	2.0	0.006094306	0.033154016
1392905_at	Guanine nucleotide binding protein, gamma 2	Gng2	1.3	0.006095437	0.033154016
1374938_at	ZW10 homolog, centromere/kinetochore protein (Drosophila)	Zw10	1.3	0.006128465	0.033278637
1397881_at	similar to hypothetical protein (predicted)	RGD1306694_predicted	1.8	0.006147736	0.033360263
1375054_at	Far upstream element (FUSE) binding protein 1	Fubp1	1.7	0.006149581	0.033360263
1397203_at	PRP4 pre-mRNA processing factor 4 homolog B (yeast)	Prpf4b	2.2	0.00615349	0.033363957
1371815_at	microfibrillar-associated protein 2 (predicted)	Mfap2_predicted	1.3	0.006154319	0.033363957
1393134_at	Transcribed locus	---	1.4	0.006172435	0.033440121
1377670_at	cleavage and polyadenylation factor subunit homolog (S. cerevisiae) (predicted)	Pcf11_predicted	1.6	0.006177056	0.033454136
1387348_at	insulin-like growth factor binding protein 5	Igfbp5	1.8	0.006210865	0.033626173
1388725_at	leptin receptor overlapping transcript	Leprot	1.4	0.006216844	0.033647465
1371301_at	ribosomal protein L9	Rpl9	1.4	0.006229657	0.033705726
1373864_at	mitogen-activated protein kinase kinase kinase kinase 4 (predicted)	Map4k4_predicted	1.7	0.006233462	0.033709733
1389064_at	Transcribed locus	---	1.6	0.006234497	0.033709733
1370875_at	villin 2	Vil2	1.5	0.006253388	0.033800764
1393091_at	---	---	1.4	0.006259463	0.033820643
1373998_at	Transcribed locus	---	1.4	0.006271142	0.033861157
1381847_at	Similar to BC021442 protein (predicted)	RGD1561413_predicted	1.5	0.006302884	0.033978968
1370301_at	matrix metallopeptidase 2	Mmp2	2.3	0.006307191	0.033980487
1379902_at	Transcribed locus	---	2.1	0.006308997	0.033980487
1371879_at	leucine rich repeat containing 42	Lrrc42	1.3	0.006327014	0.03405325
1392025_x_at	Transcribed locus	---	1.4	0.006333071	0.034063557
1393231_at	protein phosphatase 4, regulatory subunit 2 (predicted)	Ppp4r2_predicted	1.4	0.006337136	0.034065854
1379748_at	Similar to histocompatibility 28	LOC310968	3.6	0.006343413	0.034066009
1367515_at	CCR4-NOT transcription complex, subunit 7 (predicted)	Cnot7_predicted	1.7	0.006343883	0.034066009
1374458_at	Transcribed locus, weakly similar to XP_342571.3 similar to Serine/threonine-protein kinase Sgk2 (Serum/glucocorticoid-regulated kinase 2) [Rattus norvegicus]	---	1.5	0.006353615	0.034107135
1371688_at	translocation associated membrane protein 1	Tram1	1.4	0.006377263	0.034210938
1370042_at	stathmin-like 2	Stmn2	1.5	0.006394006	0.034262561
1391333_at	---	---	1.5	0.006395067	0.034262561
1384743_at	Transcribed locus	---	1.5	0.006450273	0.034493089
1389352_at	Transcribed locus	---	1.4	0.006450677	0.034493089

1388684_at	formin binding protein 4	Fnbp4	1.4	0.006456182	0.034500089
1390406_at	Rho GTPase activating protein 18 (predicted)	Arhgap18_predicted	1.4	0.006463079	0.034505269
1395376_at	DEAD/H (Asp-Glu-Ala-Asp/His) box polypeptide 11 (CHL1-like helicase homolog, S. cerevisiae) (predicted)	Ddx11_predicted	1.4	0.006463444	0.034505269
1398840_at	vesicle-associated membrane protein 5	Vamp5	1.6	0.00647299	0.034511885
1381902_at	Transcribed locus	---	1.7	0.006473077	0.034511885
1374548_at	AF4/FMR2 family, member 4 (predicted)	Aff4_predicted	1.4	0.006476791	0.034515186
1372615_at	amine oxidase, copper containing 3	Aoc3	1.9	0.006477893	0.034515186
1371926_at	Interleukin 6 signal transducer	Il6st	1.3	0.006493615	0.034576555
1373668_at	polymerase (RNA) II (DNA directed) polypeptide I (predicted)	Polr2i_predicted	1.3	0.006518392	0.034674809
1375269_at	similar to Putative deoxyribose-phosphate aldolase (Phosphodeoxyriboaldolase) (Deoxyriboaldolase) (DERA)	LOC686184	1.4	0.006526407	0.034691842
1399114_at	general transcription factor II E, polypeptide 2 (beta subunit) (predicted)	Gtf2e2_predicted	1.4	0.006528835	0.034691842
1387296_at	cytochrome P450, family 2, subfamily J, polypeptide 4	Cyp2j4	1.4	0.006530031	0.034691842
1389700_at	---	---	1.9	0.006534931	0.03470388
1373085_at	carbonyl reductase 3 (predicted)	Cbr3_predicted	1.7	0.00653733	0.03470388
1386895_at	melanoma antigen, family D, 1	Maged1	1.4	0.006538626	0.03470388
1389405_at	similar to chromodomain helicase DNA binding protein 9	LOC680231 /// LOC682690	1.4	0.006542287	0.034712109
1390432_at	transmembrane and coiled-coil domains 3 (predicted)	Tmco3_predicted	1.3	0.006547117	0.034726533
1398308_at	replication protein A3 (predicted)	Rpa3_predicted	1.6	0.006560251	0.034768473
1383127_at	a disintegrin and metalloprotease domain 10	Adam10	1.5	0.006562492	0.034768473
1389634_at	methionyl aminopeptidase 1 (predicted)	Metap1_predicted	1.3	0.006563479	0.034768473
1397349_at	UBX domain containing 6 (predicted)	Ubxd6_predicted	1.4	0.00657678	0.034801664
1391862_at	Transcribed locus	---	1.6	0.006581801	0.034809476
1380130_at	---	---	1.7	0.006598558	0.034867387
1370357_at	solute carrier family 30 (zinc transporter), member 4	Slc30a4	1.4	0.006600237	0.034867387
1371880_at	Sp1 transcription factor	Sp1	1.3	0.006601231	0.034867387
1385475_a_at	stimulated by retinoic acid gene 6 homolog (mouse)	Stra6	2.6	0.006605591	0.034879217
1396422_at	Transcribed locus	---	2.4	0.006609512	0.034888721
1393162_at	solute carrier family 39 (metal ion transporter), member 6	Slc39a6	1.5	0.006654032	0.035078692
1393735_at	Glutamine and serine rich 1 (predicted)	Qser1_predicted	1.8	0.006660433	0.035089007
1371340_at	ribosomal protein, large P2 /// hypothetical gene supported by X15098 /// similar to 60S acidic ribosomal protein P2	LOC363929 /// LOC498555 /// Rplp2	1.3	0.006662389	0.035089007
1389989_at	alpha thalassemia/mental retardation syndrome X-linked homolog (human)	Atrx	2.0	0.006696867	0.0352417
1390147_at	similar to mKIAA1429 protein (predicted)	RGD1559904_predicted	1.3	0.006697809	0.0352417
1373764_at	zinc finger, matrin-like (predicted)	Zfml_predicted	1.5	0.006737149	0.035403397
1377946_at	Transcribed locus	---	2.1	0.006746843	0.035431694
1372510_at	Sulfiredoxin 1 homolog (S. cerevisiae)	Srxn1	1.4	0.006752641	0.03544132
1377322_at	Transcribed locus	---	1.4	0.006752985	0.03544132
1392813_at	SRY-box containing gene 9	Sox9	1.6	0.006769915	0.035507516
1367652_at	insulin-like growth factor binding protein 3	Igfbp3	3.8	0.006779999	0.035549069
1373443_a_at	ankyrin repeat, family A (RFXANK-like), 2	Ankra2	1.4	0.006790541	0.035570324
1393236_at	Transcribed locus	---	1.4	0.006799188	0.035581624
1382199_at	Microtubule-associated protein 1 light chain 3 beta	Map1lc3b	1.9	0.006808458	0.035605435
1383452_at	Transcribed locus	---	1.5	0.006810232	0.035605435
1378668_at	Transcribed locus	---	1.6	0.006816029	0.035621401
1384699_at	Transcribed locus	---	1.9	0.006817617	0.035621401
1371908_at	NTF2-related export protein 1 (predicted)	Nxt1_predicted	1.3	0.006822659	0.035631654

1390028_at	dual-specificity tyrosine-(Y)-phosphorylation regulated kinase 2 (predicted)	Dyrk2_predicted	1.4	0.006827982	0.035631654
1389202_at	similar to Ribulose-5-phosphate-3-epimerase	MGC124653	1.4	0.006832392	0.035631654
1376896_at	similar to RIKEN cDNA 1810074P20 (predicted)	RGD1309308_predicted	1.8	0.006832578	0.035631654
1377619_at	WD repeat domain 36 (predicted)	Wdr36_predicted	1.9	0.006842871	0.035670832
1383112_at	trichorhinophalangeal syndrome I (predicted)	Trps1_predicted	2.3	0.006850161	0.035678102
1388416_at	low density lipoprotein receptor-related protein 1	Lrp1	1.5	0.006859695	0.03571645
1384186_at	similar to mKIAA0212 protein (predicted)	RGD1563633_predicted	1.4	0.006908293	0.035923995
1374812_at	similar to protein Tyr phosphatase	LOC498331	1.7	0.006911417	0.035927296
1372757_at	signal transducer and activator of transcription 1	Stat1	1.6	0.00691548	0.035927296
1377042_at	similar to polycomb group ring finger 5	LOC681178 /// LOC687730	1.7	0.006918665	0.035932494
1371623_at	similar to CG7224-PA, isoform A /// hypothetical protein LOC685888	LOC684207 /// LOC685888	1.6	0.006925883	0.035952069
1374225_at	similar to chromosome 14 open reading frame 145	LOC500700	1.5	0.006926806	0.035952069
1387247_at	proprotein convertase subtilisin/kexin type 1	Pcsk1	1.5	0.006939485	0.035983648
1398588_at	similar to step II splicing factor SLU7; DNA segment, Chr 11, ERATO Doi 730, expressed; DNA segment, Chr 3, Brigham & Womens Genetics 0878 expressed	LOC303057	1.7	0.00694345	0.035983648
1387227_at	Wiskott-Aldrich syndrome protein interacting protein	Waspip	1.3	0.006981869	0.036157992
1382905_at	Collagen-binding factor Endo180	Endo180	1.5	0.007000575	0.036230857
1392898_at	Transcribed locus	---	1.5	0.007004556	0.036230857
1373206_at	fibronectin type III domain containing 3B (predicted)	Fndc3b_predicted	1.3	0.00700475	0.036230857
1373430_at	bromodomain adjacent to zinc finger domain, 2B (predicted)	Baz2b_predicted	1.5	0.00702912	0.036334057
1371518_at	Nidogen 1	Nid1	1.6	0.007052263	0.036394374
1371411_at	plexin B2	Plxnb2	1.3	0.007052667	0.036394374
1370387_at	cytochrome P450, family 3, subfamily a, polypeptide 13	Cyp3a13	1.8	0.007056278	0.036394374
1377726_at	tripartite motif protein 25	Trim25	1.6	0.007061599	0.03639899
1375726_at	LIM domain only protein 7	LMO7	1.5	0.00706847	0.036422993
1393240_at	EGF-containing fibulin-like extracellular matrix protein 2	Efemp2	1.6	0.007090561	0.036513949
1378973_at	Transcribed locus	---	1.4	0.007106642	0.036585303
1367682_at	midkine	Mdk	1.4	0.007110313	0.036592751
1389907_at	zinc finger and BTB domain containing 8 opposite strand (predicted)	Zbtb8os_predicted	1.4	0.00713388	0.036691077
1389527_at	promethin	LOC378467	1.8	0.007140092	0.036709105
1399034_at	pecanex homolog (Drosophila)	Pcnx	1.6	0.007151553	0.036716103
1383110_at	kelch-like 24 (Drosophila)	Klhl24	2.1	0.007177156	0.036798584
1378012_at	synaptonemal complex protein SC65	Sc65	2.0	0.0071803	0.036803233
1397380_at	PX domain containing serine/threonine kinase	Pxk	1.7	0.007184628	0.036813944
1370000_at	nucleobindin 2	Nucb2	1.7	0.007206928	0.036882251
1386911_at	ATPase, Na+/K+ transporting, alpha 2 polypeptide	Atp1a2	1.4	0.007231008	0.036993971
1388752_at	CDNA clone IMAGE:7370517	---	1.7	0.007239754	0.037023131
1383005_at	Transcribed locus	---	1.5	0.007241209	0.037023131
1370189_at	splicing factor, arginine/serine-rich 10 (transformer 2 homolog, Drosophila)	Sfrs10	1.3	0.007251893	0.03706623
1376597_at	zinc finger, CCHC domain containing 10	Zcchc10	1.6	0.007273578	0.03715398
1372274_at	myeloid/lymphoid or mixed-lineage leukemia 5 (trithorax homolog, Drosophila)	Mll5	2.0	0.007279088	0.03717058

1385118_at	eukaryotic translation initiation factor 2, subunit 1 alpha /// hypothetical gene supported by NM_019356	Eif2s1 /// LOC364604 /// LOC364984	1.4	0.007308514	0.037274554
1378040_at	similar to Carbohydrate sulfotransferase D4ST1 (Dermatan 4-sulfotransferase 1) (D4ST-1)	LOC687718 /// LOC691394	1.5	0.00732885	0.037361399
1393891_at	procollagen, type VIII, alpha 1 (predicted)	Col8a1_predicted	2.7	0.007334501	0.037372329
1395966_at	potassium channel tetramerisation domain containing 14 (predicted)	Kctd14_predicted	1.6	0.007373579	0.037524947
1376782_at	CDC14 cell division cycle 14 homolog A (S. cerevisiae) (predicted)	Cdc14a_predicted	1.4	0.007392815	0.037610968
1374738_at	serologically defined colon cancer antigen 10	Sdccag10	1.5	0.007395055	0.037610968
1382194_at	Coagulation factor VIII	F8	2.3	0.007420449	0.037705144
1398695_at	Amyloid beta (A4) precursor protein	App	2.2	0.007445836	0.03780489
1383625_a_at	similar to zinc finger protein (predicted)	RGD1562173_predicted	1.5	0.007446373	0.03780489
1372478_at	similar to chemokine-like factor super family 7	LOC501065	1.5	0.007451069	0.037814003
1393242_at	similar to chromosome 16 open reading frame 33; minus -99 protein (predicted)	RGD1310922_predicted	1.5	0.007463096	0.037828355
1368817_at	proteasome (prosome, macropain) activator subunit 4	Psme4	1.4	0.007470742	0.037843783
1396775_at	Transcribed locus	---	2.5	0.007499311	0.037950782
1370445_at	phosphatidylserine-specific phospholipase A1	Pspla1	1.8	0.00750197	0.037950782
1374362_at	similar to 4930566A11Rik protein (predicted)	RGD1306674_predicted	1.4	0.007503608	0.037950782
1375898_at	similar to RNA binding protein gene with multiple splicing (predicted)	RGD1561067_predicted	1.8	0.007504725	0.037950782
1371529_at	receptor (TNFRSF)-interacting serine-threonine kinase 1 (predicted)	Ripk1_predicted	1.6	0.007505709	0.037950782
1390723_at	Catenin (cadherin-associated protein), alpha 1	Catna1	1.6	0.007565248	0.038228328
1372109_at	---	---	1.4	0.007569284	0.038230053
1388931_at	mitochondrial ribosomal protein L13	Mrpl13	1.3	0.007570239	0.038230053
1384840_at	Paired related homeobox 1	Prrx1	1.6	0.007606922	0.038379955
1374156_at	membrane protein, palmitoylated 5 (MAGUK p55 subfamily member 5) (predicted)	Mpp5_predicted	1.7	0.007615478	0.038411143
1376047_at	3'-phosphoadenosine 5'-phosphosulfate synthase 2 (predicted)	Papss2_predicted	1.7	0.007617774	0.038411143
1376339_at	---	---	1.3	0.007620803	0.038414641
1371813_at	HIRA interacting protein 3	Hirip3	1.6	0.007627544	0.038428111
1383853_at	dual-specificity tyrosine-(Y)-phosphorylation regulated kinase 3	Dyrk3	1.6	0.007628148	0.038428111
1377846_a_at	---	---	1.4	0.007651122	0.038501439
1391463_at	DEAD (Asp-Glu-Ala-Asp) box polypeptide 58 (predicted)	Ddx58_predicted	4.1	0.007653158	0.038501439
1385408_at	similar to mKIAA0518 protein (predicted)	RGD1561597_predicted	1.5	0.007678549	0.038587466
1372013_at	interferon induced transmembrane protein 1 (predicted)	Ifitm1_predicted	2.0	0.007685538	0.038607162
1395351_at	Dynein-like protein 2	DLP2	1.7	0.007703138	0.038663782
1384327_at	differentially expressed in FDCP 8	Def8	1.4	0.007764001	0.038921761
1390543_at	Transcribed locus	---	1.6	0.007794104	0.039036975
1391643_at	---	---	1.5	0.007799096	0.039044564
1378210_at	myeloid/lymphoid or mixed-lineage leukemia	Mll	1.4	0.007802741	0.039044564
1379485_at	eukaryotic translation initiation factor 3, subunit 10 (theta)	Eif3s10	1.5	0.007821188	0.039124972
1384282_at	zinc finger proliferation 1	Zipro1	1.4	0.007824498	0.039129626
1368186_a_at	spleen tyrosine kinase	Syk	1.4	0.007830213	0.039142381
1381757_at	hypothetical LOC305552 (predicted)	RGD1309501_predicted	1.4	0.007835692	0.039142381
1388313_at	ribosomal protein s25	Rps25	2.2	0.007837362	0.039142381
1373540_at	heterogeneous nuclear ribonucleoprotein A2/B1 (predicted)	Hnrpa2b1_predicted	1.5	0.007838947	0.039142381

1393798_at	alpha thalassemia/mental retardation syndrome X-linked homolog (human)	Atrx	2.9	0.007890653	0.039352776
1382077_at	Transcribed locus	---	1.3	0.007909848	0.039424599
1381619_at	A kinase (PRKA) anchor protein 5	Akap5	2.2	0.007912814	0.039427435
1399028_at	CDNA clone IMAGE:7367808	---	1.3	0.007918863	0.039445624
1390659_at	Transcribed locus	---	1.6	0.007938827	0.039521134
1373372_at	similar to lymphocyte antigen 6 complex, locus E ligand	LOC501282	1.3	0.007980612	0.039657132
1392605_at	Transcribed locus	---	2.0	0.008001009	0.039720606
1389005_at	---	---	1.3	0.008004426	0.039720606
1381510_at	Transcribed locus	---	1.5	0.008009884	0.039730572
1375246_at	hypothetical protein LOC679388 /// hypothetical protein LOC679747 /// hypothetical protein LOC685117 /// hypothetical protein LOC686398	LOC679388 /// LOC679747 /// LOC685117 /// LOC686398	1.3	0.008021276	0.039763092
1372293_at	Transcribed locus	---	1.4	0.008032946	0.039806117
1398754_at	ubiquitin A-52 residue ribosomal protein fusion product 1	Uba52	1.3	0.008037077	0.039806117
1371871_at	RAB12, member RAS oncogene family	Rab12	1.9	0.008037215	0.039806117
1370062_at	hypoxia induced gene 1	Hig1	1.3	0.008052502	0.039869822
1374519_at	---	---	1.4	0.008085838	0.039974712
1373576_at	---	---	1.4	0.008097486	0.040010982
1398595_at	RNA binding motif protein 5	Rbm5	1.7	0.008106689	0.040041687
1398884_at	prefoldin 5 (predicted)	Pfdn5_predicted	1.7	0.008149913	0.040202018
1394784_at	similar to chromosome 20 open reading frame 6	RGD1306067	1.8	0.00815137	0.040202018
1385177_at	Similar to sorbin and SH3 domain containing 1 isoform 3	LOC686098	1.7	0.008178444	0.040311007
1382135_at	similar to CG9346-PA (predicted)	RGD1307882_predicted	1.5	0.008201725	0.040377359
1383137_at	SRY-box containing gene 4 (predicted)	Sox4_predicted	1.6	0.00821188	0.04040353
1370339_at	---	---	1.4	0.008260769	0.040534945
1375308_at	Transcribed locus	---	1.5	0.008297124	0.040664807
1376937_at	similar to 4631422O05Rik protein (predicted)	RGD1565927_predicted	1.5	0.008301508	0.040666938
1383311_at	similar to cisplatin resistance associated	LOC682257 /// LOC689613	1.5	0.008307656	0.04068006
1395557_at	Transcribed locus	---	1.7	0.008379701	0.04098806
1381489_at	Transcribed locus	---	1.8	0.008387405	0.041009525
1372059_at	similar to RIKEN cDNA 2610528E23	RGD1309437	1.4	0.008395961	0.041039159
1393706_at	six transmembrane epithelial antigen of the prostate 1 (predicted)	Steap1_predicted	1.8	0.008415883	0.041075502
1388667_at	mannosidase 2, alpha 1	Man2a1	1.6	0.008435882	0.041132289
1389632_at	Transcribed locus, strongly similar to XP_228131.4 similar to Rho-related BTB domain-containing protein 1 [Rattus norvegicus]	---	1.7	0.008440347	0.041133873
1384180_at	interferon-induced protein with tetratricopeptide repeats 2	Ifit2	4.0	0.008469098	0.041225133
1393214_at	Similar to zinc finger protein 458	LOC685865	1.7	0.008477232	0.041252516
1385164_at	Transcribed locus	---	1.7	0.008488187	0.041293612
1388615_at	RAS-related protein 1a	Rap1a	1.5	0.0085005	0.041330212
1388492_at	TNFAIP3 interacting protein 1 (predicted)	Tnip1_predicted	1.5	0.008501563	0.041330212
1380568_a_at	---	---	1.4	0.008520355	0.041401121
1395770_at	Transcribed locus	---	1.7	0.008544056	0.041504028
1370968_at	nuclear factor of kappa light chain gene enhancer in B-cells 1, p105	Nfkb1	1.5	0.008549385	0.041517652
1392330_at	HemK methyltransferase family member 2 (predicted)	Hemk2_predicted	1.7	0.008560413	0.041554015
1391911_at	---	---	1.7	0.008561926	0.041554015
1368593_at	CD1d1 antigen	Cd1d1	1.3	0.008567906	0.041570772
1368358_a_at	protein tyrosine phosphatase, receptor type, R	Ptprr	1.5	0.008584267	0.041625594
1383237_at	Transcribed locus	---	1.8	0.008598768	0.041643841
1377755_at	activating signal cointegrator 1 complex subunit 3 (predicted)	Ascc3_predicted	1.4	0.00861586	0.041705023
1391431_at	Transcribed locus	---	1.7	0.008629981	0.041761083
1373311_at	PHD finger protein 3 (predicted)	Phf3_predicted	1.3	0.008641688	0.041793147

1381065_at	hypothetical protein LOC499120	LOC499120	1.4	0.008658373	0.041860901
1398397_at	Transcribed locus	---	1.4	0.008677896	0.041920647
1388771_at	CGG triplet repeat binding protein 1 (predicted)	Cggbp1_predicted	1.4	0.008681796	0.041925478
1372273_at	glycophorin C (Gerbich blood group)	Gypc	2.0	0.008694299	0.041961216
1384562_at	Transcribed locus	---	2.5	0.008701688	0.041984558
1391876_at	---	---	1.7	0.008712918	0.042026416
1372829_at	RNA binding motif protein 8 (predicted)	Rbm8_predicted	1.6	0.008728158	0.042075245
1390885_at	Transcribed locus	---	1.7	0.008752246	0.042129627
1374567_at	proline/serine-rich coiled-coil 2	Psrc2	1.3	0.008758806	0.042146595
1370282_at	cysteine and glycine-rich protein 2	Csrp2	3.0	0.008760895	0.042146595
1370167_at	syndecan 2	Sdc2	1.4	0.008765269	0.042155306
1373100_at	similar to RIKEN cDNA 2900010J23	LOC499779	1.4	0.00878855	0.042217895
1372250_at	SCF apoptosis response protein 1	LOC499941	1.2	0.008794067	0.04222337
1389258_at	ring finger protein 138	Rnf138	1.4	0.008794824	0.04222337
1372372_at	similar to Ab2-225	RGD1306952	1.3	0.008822423	0.042318814
1374876_at	leptin receptor overlapping transcript-like 1	Leprotl1	1.3	0.008848246	0.042405526
1382512_at	Transcribed locus	---	1.4	0.008850813	0.042405526
1377657_at	inhibitor of Bruton agammaglobulinemia tyrosine kinase (predicted)	Ibtk_predicted	1.4	0.008869024	0.042468038
1381447_at	similar to RIKEN cDNA 9430015G10	RGD1311517	1.5	0.008872697	0.042472133
1377883_at	Transcribed locus	---	1.7	0.008890361	0.042520692
1382913_at	cortactin binding protein 2	Cttnbp2	1.6	0.008893338	0.042522566
1389468_at	ribose 5-phosphate isomerase A (predicted)	Rpia_predicted	1.4	0.008899946	0.042541794
AFFX_ratb1/X12957_a	---	---	1.3	0.008905327	0.042555148
1379942_at	similar to SOX2 protein (predicted)	RGD1565646_predicted	1.8	0.008951679	0.042751807
1377118_at	similar to KIAA1712 protein	RGD1308517	1.4	0.008963658	0.04278195
1380489_at	Similar to RIKEN cDNA G430041M01 (predicted)	RGD1562563_predicted	1.8	0.008965794	0.04278195
1374578_at	Transcribed locus	---	1.3	0.008978225	0.042812374
1373086_at	similar to RIKEN cDNA F730014I05 (predicted)	RGD1310800_predicted	1.6	0.00898114	0.042812374
1391150_at	Wingless-related MMTV integration site 16	Wnt16	2.0	0.008986369	0.042818018
1394654_at	---	---	1.6	0.009037297	0.043006154
1377778_at	ischemia related factor vof-16	Vof16	2.5	0.009038927	0.043006154
1392056_at	PHD finger protein 3 (predicted)	Phf3_predicted	1.4	0.009071606	0.043099302
1378322_at	Similar to APG4-D protein	LOC686505	1.5	0.009093067	0.043163855
1392049_at	A kinase (PRKA) anchor protein 11	Akap11	1.4	0.009117463	0.043208808
1372964_at	AT rich interactive domain 5B (Mrf1 like) (predicted)	Arid5b_predicted	1.8	0.009118298	0.043208808
1393987_s_at	G protein-coupled receptor kinase 6	Gprk6	1.5	0.009131619	0.043247011
1392729_at	Transcribed locus	---	1.3	0.00917186	0.043373062
1382939_at	translocated promoter region	Tpr	1.8	0.009174057	0.043373062
1379542_at	similar to Small nuclear ribonucleoprotein F (snRNP-F) (Sm protein F) (Sm-F) (SmF)	LOC680737 /// LOC687845	1.8	0.009185933	0.043394069
1386525_at	THO complex 2 (predicted)	Thoc2_predicted	1.9	0.009193168	0.043413504
1373878_at	Rho-associated coiled-coil forming kinase 1	Rock1	1.3	0.009219816	0.043514355
1382312_at	AT rich interactive domain 5B (Mrf1 like) (predicted)	Arid5b_predicted	2.2	0.009248392	0.043618941
1371186_at	integrin, alpha 6	Itga6	1.7	0.009264019	0.043663038
1368173_at	nucleolar protein 5	Nol5	2.3	0.009269193	0.043663038
1368012_at	telomerase associated protein 1	Tep1	1.4	0.009271969	0.043663038
1393191_at	similar to RIKEN cDNA 2610200G18 (predicted)	RGD1561205_predicted	1.9	0.009308301	0.043793746
1383228_at	---	---	2.1	0.009335044	0.043869372
1389379_at	Transcribed locus	---	1.4	0.009354962	0.04395042
1391415_at	putatative 28 kDa protein	LOC289809	1.4	0.009369522	0.04399369
1372168_s_at	insulin-like growth factor binding protein 6	Igfbp6	1.6	0.00937755	0.04401882
1379420_at	similar to Dehydrogenase/reductase SDR family member 7 precursor (Retinal short-chain dehydrogenase/reductase 4) (predicted)	RGD1565002_predicted	1.4	0.009397379	0.044086734

1399167_a_at	Transcribed locus, strongly similar to XP_341668.3 similar to growth factor receptor bound protein 2-associated protein 1 [Rattus norvegicus]	---	1.3	0.009432986	0.044193866
1395781_at	v-myc myelocytomatosis viral oncogene homolog 1, lung carcinoma derived (avian)	Mycl1	1.3	0.009435182	0.044193866
1380250_at	insulin responsive sequence DNA binding protein-1	Sned1	1.7	0.009502028	0.044422794
1391345_at	BMP-binding endothelial regulator (predicted)	Bmper_predicted	2.6	0.009531637	0.044500815
1389479_at	Kruppel-like factor 3 (basic) (mapped)	Klf3	1.4	0.009538577	0.044520579
1373343_at	Transcribed locus	---	1.6	0.009578262	0.044638549
1371910_at	Transcribed locus	---	1.6	0.009580136	0.044638549
1371232_a_at	chondroitin sulfate proteoglycan 2	Cspg2	1.9	0.00958425	0.044645071
1379150_at	Cytoplasmic FMR1 interacting protein 2 (predicted)	Cyfip2_predicted	2.6	0.009589039	0.044654734
1367485_at	transcription elongation factor A (SII) 1	Tcea1	1.3	0.009606746	0.044705033
1372934_at	similar to 1700019E19Rik protein (predicted)	RGD1307392_predicted	1.4	0.009607994	0.044705033
1377333_at	Transcribed locus	---	1.6	0.009636672	0.044825784
1383528_at	similar to mKIAA0215 protein (predicted)	RGD1563945_predicted	1.9	0.009648458	0.044867919
1391481_at	Calpastatin	Cast	2.0	0.009686575	0.044998439
1381981_at	Transcribed locus	---	1.7	0.009690164	0.044998439
1385275_at	wingless-related MMTV integration site 16	Wnt16	1.6	0.009712599	0.045064269
1374966_at	doublecortin	Dcx	1.5	0.009733259	0.045083819
1398947_at	pumilio 2 (Drosophila)	Pum2	1.3	0.009745841	0.045108804
1389126_at	coiled-coil-helix-coiled-coil-helix domain containing 1 (predicted)	Chchd1_predicted	1.4	0.00974688	0.045108804
1372835_at	ras homolog gene family, member J	Rhoj	1.6	0.009762548	0.045155901
1372430_at	similar to RIKEN cDNA 1700037H04	RGD1311739	1.4	0.009798606	0.045297211
1374531_at	Transcribed locus	---	2.8	0.009801941	0.045299894
1388131_at	tubulin, beta 2b	Tubb2b	1.5	0.009827821	0.045388756
1379384_at	Sp1 transcription factor	Sp1	1.7	0.00985504	0.045481395
1384242_at	Transcribed locus	---	1.4	0.009866902	0.045523368
1382023_at	Transcribed locus	---	1.7	0.009872984	0.045531176
1388448_at	Hypothetical protein LOC691031	LOC691031	1.7	0.009874131	0.045531176
1376206_at	Transcribed locus	---	1.4	0.009883901	0.045551032
1383218_at	---	---	1.5	0.009883976	0.045551032
1373059_at	ankyrin repeat domain 13	Ankrd13	1.5	0.009935465	0.045724265
1371977_at	actin related protein 2/3 complex, subunit 3 (predicted)	Arpc3_predicted	1.4	0.009972372	0.045842812
1379583_at	AFG3(ATPase family gene 3)-like 1 (yeast) (predicted)	Afg3l1_predicted	1.5	0.010013776	0.045981739
1387907_at	inositol 1,4,5-triphosphate receptor 1	Itpr1	1.4	0.010029197	0.046037067
1390372_at	Mitogen activated protein kinase kinase kinase 12	Map3k12	1.4	0.010031423	0.046037067
1389913_at	---	---	1.5	0.010039274	0.046060245
1395359_at	Spectrin beta 2	Spnb2	1.5	0.010059492	0.046140133
1374564_at	deltex 2 homolog (Drosophila)	Dtx2	1.4	0.010087751	0.046218199
1390914_at	Friend leukemia integration 1	Fli1	1.8	0.010091652	0.046223196
1381381_at	Membrane associated DNA binding protein (predicted)	Mnab_predicted	1.7	0.010098906	0.04623828
1371720_at	similar to mitochondrial ribosomal protein L20	LOC680747 /// LOC687253	1.4	0.010135387	0.046358965
1373628_at	Transcribed locus	---	3.1	0.010153252	0.046427768
1374432_at	---	---	1.3	0.010167593	0.046477415
1391689_at	similar to Retinoblastoma-binding protein 2 (RBBP-2)	LOC312678	1.5	0.01016976	0.046477415
1395819_at	Transcribed locus	---	3.0	0.01018666	0.046528792
1374709_at	Transcribed locus	---	1.6	0.010226231	0.046657713
1373160_at	similar to phosphatidic acid phosphatase type 2 domain containing 1A	LOC680466 /// LOC683534	1.4	0.010268697	0.046824736
1368712_at	zinc finger protein 386 (Kruppel-like)	Znf386	1.7	0.010271379	0.046824736
1367978_at	adenylate cyclase 2	Adcy2	1.4	0.010276343	0.046826362
1369943_at	transglutaminase 2, C polypeptide	Tgm2	1.7	0.01027743	0.046826362

1388737_at	similar to polybromo-1 (predicted)	RGD1565549_predicted	1.5	0.01028754	0.046859445
1372808_at	similar to Bifunctional methylenetetrahydrofolate dehydrogenase/cyclohydrolase, mitochondrial precursor	LOC680308 /// LOC684490	1.4	0.010324305	0.046964517
1383263_at	osteoglycin (predicted)	Ogn_predicted	2.1	0.010324884	0.046964517
1384901_at	Zinc finger protein 451	Zfp451	1.3	0.010335441	0.046999541
1372549_at	similar to RIKEN cDNA 2600017H02 (predicted)	RGD1561781_predicted	1.6	0.010338643	0.047001107
1398131_at	Glycoprotein m6b	Gpm6b	2.6	0.010348681	0.047033742
1395264_at	similar to Rap1-interacting factor 1 (predicted)	RGD1562474_predicted	2.1	0.010394668	0.047216652
1374036_at	minichromosome maintenance deficient 2 mitotin (S. cerevisiae) (predicted)	Mcm2_predicted	1.6	0.010429494	0.047328547
1396463_at	similar to Pinin	LOC368070	3.4	0.010433312	0.047328547
1374896_at	Transcribed locus	---	1.3	0.010433689	0.047328547
1371536_at	calcium regulated heat stable protein 1	Carhsp1	1.4	0.01044765	0.047378811
1383602_at	pumilio 1 (Drosophila) (predicted)	Pum1_predicted	1.7	0.010465701	0.047434511
1385199_a_at	RNA binding motif protein 34	Rbm34	1.4	0.010506868	0.047594869
1372681_at	---	---	1.5	0.010515572	0.047621179
1370458_at	hepatoma-derived growth factor, related protein 3	Hdgfrp3	1.5	0.01052128	0.047633914
1378416_at	Transcribed locus	---	1.6	0.010542404	0.047716416
1390806_at	similar to RIKEN cDNA B230118H07 (predicted)	RGD1309730_predicted	1.6	0.010550075	0.047725196
1373632_at	TAF9 RNA polymerase II, TATA box binding protein (TBP)-associated factor	Taf9	1.4	0.010550147	0.047725196
1374183_at	similar to RIKEN cDNA 1810011O16 (predicted)	RGD1309624_predicted	1.5	0.010594059	0.047835009
1395974_at	similar to hypothetical protein (predicted)	RGD1311334_predicted	1.4	0.010596014	0.047835009
1388843_at	Transcribed locus	---	1.4	0.010597417	0.047835009
1377237_at	Transcribed locus	---	1.3	0.010623084	0.0479244
1383455_at	glutamyl-prolyl-tRNA synthetase	Eprs	1.4	0.010628086	0.047925883
1395944_at	Transcribed locus	---	1.7	0.010658563	0.048035559
1374695_at	chromobox homolog 1 (Drosophila HP1 beta) (predicted)	Cbx1_predicted	1.5	0.010672787	0.048068456
1383534_at	Transcribed locus, weakly similar to XP_522567.1 similar to zinc finger protein 605 [Pan troglodytes]	---	1.4	0.010679338	0.048084796
1375266_at	---	---	1.8	0.01069241	0.048130476
1391383_at	cartilage acidic protein 1	Crtac1	2.0	0.010699491	0.048149174
1384268_at	similar to hypothetical protein FLJ40432 (predicted)	RGD1310683_predicted	2.0	0.010710544	0.048159383
1378154_at	Transcribed locus	---	1.4	0.010729341	0.048217541
1397286_at	Transcription factor 4	Tcf4	1.7	0.010737185	0.048226438
1390698_at	Transcribed locus	---	1.3	0.010743836	0.048235169
1377310_at	Transcribed locus	---	2.1	0.010744994	0.048235169
1374007_at	similar to step II splicing factor SLU7; DNA segment, Chr 11, ERATO Doi 730, expressed; DNA segment, Chr 3, Brigham & Womens Genetics 0878 expressed	LOC303057	1.6	0.010759017	0.048284943
1380101_at	poly(A) polymerase gamma (predicted)	Papolg_predicted	1.4	0.010780796	0.048365525
1378347_at	Transcribed locus	---	1.7	0.010782854	0.048365525
1390414_at	similar to hypothetical protein (predicted)	RGD1308489_predicted	1.3	0.010787832	0.048374662
1398757_at	nucleophosmin 1	Npm1	1.3	0.010804671	0.048436968
1394042_at	---	---	1.4	0.010823422	0.048494593
1371324_at	similar to Splicing factor 3B subunit 5 (SF3b5) (Pre-mRNA splicing factor SF3b 10 kDa subunit)	LOC680891 /// LOC684491	1.5	0.010832363	0.048521436
1368885_at	ectonucleoside triphosphate diphosphohydrolase 1	Entpd1	1.6	0.010850492	0.048589408
1382613_at	Transcribed locus	---	1.5	0.010859191	0.048615127
1373612_at	Transcribed locus	---	1.3	0.010879918	0.04865916
1383040_a_at	---	---	2.4	0.010885854	0.048668261
1390234_at	splicing factor 3b, subunit 1	Sf3b1	1.3	0.01091001	0.048763

1377949_s_at	---	---	1.6	0.010923792	0.048806649
1389485_at	similar to transformed mouse 3T3 cell double minute 1	LOC314859	1.4	0.010927417	0.048806649
1387264_at	potassium channel, subfamily K, member 6	Kcnk6	2.1	0.010928678	0.048806649
1388616_at	similar to RIKEN cDNA 2310042G06	MGC72955	1.4	0.010958197	0.048911922
1387543_at	solute carrier family 5 (choline transporter), member 7	Slc5a7	1.8	0.010976131	0.048937584
1392487_at	Transcribed locus	---	1.3	0.010976238	0.048937584
1388392_at	Tax1 (human T-cell leukemia virus type I) binding protein 3	Tax1bp3	1.5	0.010978823	0.048937584
1383409_at	---	---	1.6	0.010995152	0.048983817
1371352_at	high mobility group nucleosomal binding domain 2	Hmgn2	1.5	0.011004787	0.049000199
1374498_at	Similar to CG13379-PA (predicted)	RGD1311493_predicted	1.3	0.011047804	0.049138527
1374484_at	transmembrane protein 39a	Tmem39a	1.3	0.011071202	0.049229286
1373986_at	Transcribed locus	---	1.7	0.01111274	0.049360034
1393803_at	caspase 8 associated protein 2 (predicted)	Casp8ap2_predicted	1.3	0.011113188	0.049360034
1373961_at	similar to 4930453N24Rik protein	MGC95208	1.4	0.011129896	0.049396798
1371996_at	AE binding protein 2 (predicted)	Aebp2_predicted	1.5	0.011150426	0.049445444
1392223_at	epsin 2	Epn2	1.4	0.011150728	0.049445444
1392446_at	kelch-like 2, Mayven (Drosophila) (predicted)	Klhl2_predicted	1.5	0.011179797	0.049538066
1389789_at	Transcribed locus	---	1.3	0.011211142	0.049663577
1380001_at	similar to Pinin	LOC368070	2.4	0.01121802	0.049680665
1382205_at	Transcribed locus	---	2.6	0.011235718	0.049745649
1390298_at	sorting nexin associated golgi protein 1 (predicted)	Snag1_predicted	1.3	0.011242804	0.04976363
1393107_at	Transcribed locus	---	1.4	0.011275153	0.049839752
1378373_at	Transcribed locus	---	1.5	0.011286751	0.049864217
1376729_at	similar to DIP13 alpha (predicted)	RGD1309388_predicted	1.3	0.011302106	0.049918648
1374803_at	---	---	1.5	0.011318153	0.049934837
1383470_at	Transcribed locus	---	1.5	0.011321345	0.049934837
1390941_at	retinoblastoma binding protein 6	Rbbp6	1.5	0.011323987	0.049934837
1385261_s_at	Transcribed locus	---	1.4	0.011330625	0.049942099
1379605_at	Transcribed locus	---	1.4	0.011336591	0.049950239
1397251_at	Similar to hypothetical protein MGC32132 (predicted)	RGD1307915_predicted	1.3	0.011411773	0.050214245
1375634_at	coiled-coil domain containing 53 (predicted)	Ccdc53_predicted	1.7	0.011528333	0.050700006
1379164_at	Zinc finger protein 142 (clone pHZ-49) (predicted)	Znf142_predicted	1.5	0.011580312	0.050872282
1376701_a_at	similar to 5930416I19Rik protein	MGC94282	1.3	0.011592001	0.05087813
1374632_at	phosphatidylserine receptor	Ptdsr	1.4	0.011598153	0.05087813
1374943_at	Transcribed locus	---	1.5	0.011602861	0.05087813
1392961_at	MOB1, Mps One Binder kinase activator-like 1B (yeast)	Mobk1b	1.5	0.011612182	0.050891865
1385869_at	Zinc finger protein 281	Zfp281	1.8	0.011647786	0.051020716
1399020_at	similar to family with sequence similarity 40, member A	LOC362012	1.4	0.011715799	0.051237453
1388164_at	RT1 class Ib, locus S3	RT1-S3	2.0	0.011715957	0.051237453
1372356_at	ubiquitin specific protease 54	Usp54	1.5	0.011730568	0.051260464
1394761_at	Transcribed locus	---	1.5	0.011740163	0.051288763
1371803_at	GM2 ganglioside activator protein	Gm2a	1.5	0.011748456	0.051289708
1373808_at	CDNA clone IMAGE:7193711	---	1.9	0.011775975	0.051376986
1390688_at	DEAD (Asp-Glu-Ala-Asp) box polypeptide 50	Ddx50	1.3	0.011790148	0.051411546
1379352_at	phosphatidylinositol-4-phosphate 5-kinase, type 1, beta	Pip5k1b	1.8	0.011804158	0.051443188
1383637_at	Transcribed locus	---	1.6	0.011807173	0.051443188
1377723_at	Transcribed locus	---	1.9	0.011809915	0.051443188
1379435_at	deoxyguanosine kinase (predicted)	Dguok_predicted	1.5	0.011831719	0.051497253
1383962_at	Cd27 binding protein (Hindu God of destruction) (predicted)	Siva_predicted	1.4	0.011864709	0.051599877
1376595_at	similar to peroxisome biogenesis factor 1 (predicted)	RGD1559939_predicted	1.5	0.01187655	0.051637719

1383945_at	uridine-cytidine kinase 2 /// uridine monophosphate kinase (predicted)	Uck2 /// Umpk_predicted	1.7	0.011881895	0.051647309
1368359_a_at	VGF nerve growth factor inducible	Vgf	3.5	0.011885523	0.051649428
1377080_at	neurotrophin receptor associated death domain	Nradd	1.7	0.011893701	0.051671313
1367935_at	smu-1 suppressor of mec-8 and unc-52 homolog (C. elegans)	Smu1	1.3	0.01195471	0.05190894
1379098_at	---	---	1.3	0.01197212	0.05197082
1388141_at	centrin 3	Cetn3	1.4	0.011990325	0.052036114
1382735_at	autophagy-related 12 (yeast)	Atg12	1.6	0.012009867	0.052093432
1374549_at	zinc responsive protein ZD7	LOC474154	1.4	0.012023233	0.052137658
1386683_at	Transcribed locus	---	1.6	0.012051336	0.052245748
1391026_at	immediate early response 5-like	Ier5l	2.5	0.012077717	0.052332533
1388618_at	nidogen 2	Nid2	1.9	0.012098281	0.052407829
1394795_at	Transcribed locus	---	1.9	0.012132795	0.052515847
1385761_s_at	Transcribed locus	---	1.4	0.01213966	0.052531736
1388330_at	vitamin K epoxide reductase complex, subunit 1	Vkorc1	1.4	0.012149674	0.052533607
1383253_at	Transcribed locus	---	2.0	0.012171287	0.052602044
1379379_at	Transcribed locus	---	1.5	0.012171898	0.052602044
1373087_at	Membrane-associated ring finger (C3HC4) 7	Mar-07	1.4	0.012177297	0.052611553
1394591_at	zinc finger protein 207	Zfp207	1.3	0.012197837	0.052658235
1380293_at	similar to chromosome 18 open reading frame 54	LOC361346	1.4	0.012199572	0.052658235
1374830_at	Transcribed locus, strongly similar to XP_574081.1 similar to Coiled-coil domain containing protein 3 precursor [Rattus norvegicus]	---	1.8	0.012243913	0.052813739
1389780_at	tissue factor pathway inhibitor	Tfpi	1.5	0.012246571	0.052813739
1370193_at	protein tyrosine phosphatase 4a1	Ptp4a1	1.4	0.012284318	0.05294876
1372531_at	protein tyrosine phosphatase, receptor-type, F interacting protein, binding protein 2	Ppfibp2	1.3	0.012319687	0.053087297
1379130_at	CDW92 antigen	Cdw92	1.5	0.012342413	0.053171298
1371322_at	laminin, gamma 1	Lamc1	1.6	0.012346302	0.053174121
1386611_at	kelch-like 24 (Drosophila)	Klhl24	1.4	0.012366668	0.053233959
1377071_at	Transcribed locus	---	1.4	0.012387228	0.053294568
1396356_at	Transcribed locus	---	1.4	0.012396526	0.053319146
1398367_at	speckle-type POZ protein	Spop	1.5	0.012433247	0.053436648
1376121_at	PHD finger protein 10	Phf10	1.6	0.012456835	0.053523705
1392054_at	similar to polybromo-1 (predicted)	RGD1565549_predicted	1.4	0.01246239	0.053523705
1389442_at	Transcribed locus	---	1.3	0.012466323	0.053523705
1383308_a_at	similar to hypothetical protein FLJ34389 (predicted)	RGD1305243_predicted	1.4	0.012469774	0.053523705
1371715_at	LOC500651	MGC112883	1.3	0.0124894	0.053593959
1371832_at	Leo1, Paf1/RNA polymerase II complex component, homolog (S. cerevisiae)	Leo1	1.5	0.012515054	0.053662045
1389576_at	U2 small nuclear ribonucleoprotein B (predicted)	Snrpb2_predicted	1.5	0.012521827	0.053663107
1374061_at	CD302 antigen	Cd302	1.5	0.012616363	0.054026019
1394511_at	myb-like, SWIRM and MPN domains 1 (predicted)	Mysm1_predicted	1.5	0.01264552	0.054114399
1385265_a_at	similar to Protein C12orf11 (Sarcoma antigen NY-SAR-95)	LOC686480 /// LOC690728	1.5	0.012646872	0.054114399
1383332_at	Transcribed locus	---	1.5	0.01265397	0.05412403
1367561_at	ribosomal protein L27	Rpl27	1.5	0.012682969	0.054198358
1379370_at	Transcribed locus	---	1.5	0.012693648	0.054229903
1374263_at	Similar to sorbin and SH3 domain containing 1 isoform 3	LOC686098	1.8	0.012702135	0.054242784
1385697_at	cyclin T2 (predicted)	Ccnt2_predicted	1.4	0.012748225	0.054389137
1371643_at	cyclin D1	Ccnd1	1.6	0.012749534	0.054389137
1388870_at	similar to RNA-binding protein Musashi2-S (predicted)	RGD1560397_predicted	1.4	0.012750761	0.054389137
1382283_at	Transcribed locus	---	2.4	0.012767315	0.054417051
1396877_at	Laminin, gamma 1	Lamc1	1.8	0.01278336	0.054417051

1390154_at	similar to Interferon-alpha/beta receptor beta chain precursor (IFN-alpha-REC) (Type I interferon receptor) (IFN-R) (Interferon alpha/beta receptor-2)	LOC686326	1.6	0.012788469	0.054417051
1373875_at	similar to RIKEN cDNA 1190005P17 (predicted) /// hypothetical protein LOC690089	LOC690089 /// RGD1308261_predicted	1.4	0.01278883	0.054417051
1390527_at	RGD1562114 (predicted)	RGD1562114_predicted	1.4	0.012790467	0.054417051
1376610_a_at	similar to TPR repeat-containing protein KIAA1043	LOC304558	1.8	0.012890541	0.054701657
1368770_at	glucosaminyl (N-acetyl) transferase 1, core 2	Gcnt1	1.6	0.01294079	0.05488657
1382161_at	M-phase phosphoprotein 10 (U3 small nucleolar ribonucleoprotein) (predicted)	Mphosph10_predicted	1.3	0.012957925	0.054930915
1389053_at	similar to hypothetical protein FLJ20627 (predicted)	RGD1309546_predicted	1.3	0.012987701	0.055028776
1390579_at	similar to RIKEN cDNA 1810029B16 (predicted)	RGD1305222_predicted	1.5	0.012991424	0.055030376
1383213_at	epidermal growth factor receptor pathway substrate 8 (predicted)	Eps8_predicted	1.3	0.013006577	0.055071668
1377138_at	Ataxin 10	Atxn10	1.6	0.013007869	0.055071668
1380186_at	Transcribed locus	---	1.3	0.013021882	0.055116807
1388167_at	nuclear factor I/B	Nfib	1.9	0.013040179	0.055164743
1373066_at	Transcribed locus	---	1.3	0.013042569	0.055164743
1370250_at	ubiquitin-conjugating enzyme E2I /// similar to Chain A, Human Ubiquitin-Conjugating Enzyme Ubc9 (predicted) /// similar to RIKEN cDNA A930001M12 gene (predicted) /// similar to Ubiquitin-conjugating enzyme E2 I (Ubiquitin-protein ligase I) (Ubiquitin carrier protein I) (SUMO-1-protein ligase) (SUMO-1-conjugating enzyme) (Ubiquitin-conjugating enzyme UbcE2A)	LOC681942 /// LOC685390 /// RGD1562567_predicted /// RGD1566292_predicted /// Ube2i	1.3	0.01312142	0.055363937
1370242_at	ribosomal protein S23	Rps23	1.3	0.013148661	0.055425212
1371202_a_at	nuclear factor I/B	Nfib	1.3	0.013159313	0.0554559
1372207_at	bromodomain containing 8	Brd8	1.3	0.013165998	0.055469859
1398830_at	ribosomal protein L28	Rpl28	1.3	0.013198788	0.055578936
1379732_at	syntaxin 11	Stx11	1.7	0.013208783	0.055578936
1384024_at	bromodomain containing 1 (predicted)	Brd1_predicted	1.4	0.013228071	0.05561741
1391773_at	Transcribed locus	---	1.4	0.01323231	0.055621015
1376786_a_at	Transcribed locus	---	1.3	0.013250419	0.055668681
1391935_at	similar to eukaryotic translation initiation factor 4E member 3	LOC297481	1.8	0.013266634	0.055712564
1389705_at	recombination activating gene 1 activating protein 1 (predicted)	Rag1ap1_predicted	1.4	0.013287596	0.055753668
1395241_at	ADP-ribosylation factor 1	Arf1	1.4	0.013302356	0.055801364
1374197_at	Transcribed locus	---	1.3	0.013313704	0.055834727
1376703_at	nucleoporin 37 (predicted)	Nup37_predicted	1.3	0.013325119	0.055868356
1391981_at	Poliovirus receptor-related 3 (predicted)	Pvrl3_predicted	1.3	0.013351974	0.055952424
1367780_at	pituitary tumor-transforming 1	Pttg1	1.3	0.0133583	0.05596171
1385755_at	Transcribed locus	---	1.4	0.013360994	0.05596171
1372859_at	GDP-mannose pyrophosphorylase A	Gmppa	1.2	0.013369989	0.055982998
1390445_at	similar to mbt domain containing 1	LOC688133	1.3	0.013372884	0.055982998
1377116_at	Ribonuclease L (2',5'-oligoisoadenylate synthetase-dependent)	Rnasel	1.3	0.013378326	0.055991528
1372096_at	oxidative-stress responsive 1 (predicted)	Oxsr1_predicted	1.4	0.013385547	0.055995838
1370213_at	Y box protein 1 /// similar to Nuclease sensitive element-binding protein 1 (Y-box-binding protein 1) (Y-box transcription factor) (YB-1) (CCAAT-binding transcription factor I subunit A) (CBF-A) (Enhancer factor I subunit A) (EFI-A) (DNA-binding protein B) (DBPB)...	LOC686530 /// Ybx1	1.9	0.013386164	0.055995838
1373895_at	---	---	1.3	0.013431715	0.056102551

1396054_at	Membrane protein, palmitoylated 6 (MAGUK p55 subfamily member 6) (predicted)	Mpp6_predicted	1.6	0.013432141	0.056102551
1392454_at	similar to hypothetical protein FLJ20729 (predicted)	RGD1308723_predicted	1.5	0.013455164	0.056170189
1377757_at	SNF2 histone linker PHD RING helicase (predicted)	Shprh_predicted	1.3	0.013478616	0.056225279
1369631_at	---	---	1.3	0.01349802	0.056291946
1379824_at	thymocyte selection-associated HMG box gene (predicted)	Tox_predicted	1.5	0.01353944	0.056436063
1374878_at	Fms interacting protein	Fmip	1.5	0.013608502	0.056695197
1368087_a_at	protein tyrosine phosphatase, non-receptor type 21	Ptpn21	1.4	0.013612404	0.056697095
1378557_at	Similar to TAFA4 protein	LOC689043	1.6	0.013619746	0.056713313
1379341_at	USP6 N-terminal like (predicted)	Usp6nl_predicted	1.7	0.013637988	0.056774898
1375432_at	similar to exosome component 1	LOC679140	1.4	0.013641958	0.056777055
1375217_at	Meningioma expressed antigen 5 (hyaluronidase)	Mgea5	1.4	0.013655373	0.05678595
1392362_at	Transcribed locus	---	1.5	0.013655701	0.05678595
1379188_at	AT rich interactive domain 2 (Arid-rfx like) (predicted)	Arid2_predicted	1.6	0.01368766	0.056844472
1395686_at	Activating transcription factor 7 (predicted)	Atf7_predicted	1.3	0.013693219	0.056844472
1393389_at	Nuclear receptor subfamily 4, group A, member 3	Nr4a3	1.4	0.013694952	0.056844472
1385054_at	Transcribed locus	---	1.5	0.013703551	0.056844472
1385253_at	Transcribed locus	---	1.4	0.013709224	0.056844472
1389470_at	complement factor B	Cfb	1.9	0.013709996	0.056844472
1388180_at	phosphorylated adaptor for RNA export	Phax	1.4	0.013725862	0.056895914
1394964_at	similar to KIAA1096 protein (predicted) /// similar to HBxAg transactivated protein 2	LOC684194 /// LOC691387 /// RGD1566064_predicted	1.5	0.013745286	0.056947723
1395771_at	Activating transcription factor 2	Atf2	1.7	0.013769716	0.056980884
1374636_at	PHD finger protein 17 (predicted)	Phf17_predicted	1.8	0.013774076	0.056980884
1398926_at	prefoldin 1 (predicted)	Pfdn1_predicted	1.6	0.013779469	0.056988862
1392204_at	similar to NK10	LOC498945	1.5	0.013787947	0.05700959
1374652_at	Transcribed locus	---	1.6	0.013814107	0.057103403
1387197_at	osteomodulin	Omd	2.6	0.013853877	0.057218045
1385876_at	similar to Gpc6 protein (predicted)	RGD1563063_predicted	1.7	0.013866192	0.057218045
1389142_at	similar to Sulfide:quinone oxidoreductase, mitochondrial precursor	LOC691966	1.4	0.013889872	0.057287016
1377747_at	Transcribed locus	---	1.4	0.013902334	0.057309671
1368545_at	CASP8 and FADD-like apoptosis regulator	Cflar	1.5	0.013923179	0.05734821
1379316_at	---	---	1.5	0.01393462	0.057370873
1375932_at	Transcribed locus	---	1.4	0.013949737	0.057385309
1385859_at	Glutaredoxin 2 (thioltransferase)	Glrx2	1.5	0.013954397	0.057385309
1396062_at	Transcribed locus	---	1.2	0.013970106	0.05743072
1374188_at	translocation protein 1	Tloc1	1.8	0.014119963	0.057930943
1385953_at	Myeloid/lymphoid or mixed-lineage leukemia,translocated to, 3 (trithorax homolog, Drosophila)	Mllt3	1.6	0.014147662	0.058001183
1370166_at	syndecan 2	Sdc2	1.5	0.014154385	0.058014286
1391309_at	similar to Complement C1q-like protein 3 precursor (Gliacolin)	LOC680404 /// LOC684921	1.6	0.014158364	0.058016138
1395436_at	Protein phosphatase 3, catalytic subunit, alpha isoform	Ppp3ca	1.9	0.014194172	0.058143689
1382436_at	similar to RIKEN cDNA 2610002M06 (predicted)	RGD1566265_predicted	1.5	0.014250515	0.058321093
1387318_at	potassium large conductance calcium-activated channel, subfamily M, beta member 4	Kcnmb4	1.9	0.014265725	0.058339791
1370243_a_at	prothymosin alpha	Ptma	1.9	0.014317809	0.058523689

1371189_x_at	ribosomal protein SA /// similar to 40S ribosomal protein SA (p40) (34/67 kDa laminin receptor) (predicted) /// similar to 40S ribosomal protein SA (p40) (34/67 kDa laminin receptor)	LOC367035 /// LOC679411 /// LOC683125 /// LOC688911 /// RGD1563757_predicted /// Rpsa	1.5	0.014340365	0.058598432
1397583_at	Transcribed locus	---	1.4	0.01434322	0.058598432
1389555_at	transcription factor 19	Tcf19	1.9	0.014404277	0.058804058
1375901_at	DEAD (Asp-Glu-Ala-Asp) box polypeptide 21a	Ddx21a	1.3	0.014427307	0.058868852
1371848_at	similar to small nuclear ribonucleoprotein D3	LOC687711	1.4	0.014485486	0.059003614
1382466_at	similar to RIKEN cDNA 6530403A03	RGD1309020	2.0	0.014494547	0.059003614
1397362_at	Hippyragranin	LOC257650	1.6	0.014497872	0.059003614
1371237_a_at	metallothionein 1a	Mt1a	1.8	0.014499794	0.059003614
1387077_at	cAMP-regulated phosphoprotein 19	Arpp19	1.5	0.014509665	0.059029178
1367724_a_at	ATPase, H+ transporting, V0 subunit E isoform 1	Atp6v0e1	1.3	0.014529817	0.059096544
1374601_at	interferon gamma receptor 2 (predicted)	Ifngr2_predicted	1.5	0.014569642	0.059214597
1373315_at	Aryl hydrocarbon receptor nuclear translocator 2	Arnt2	1.4	0.014634743	0.059449801
1368162_at	cystatin E/M	Cst6	1.5	0.01465096	0.059486296
1378745_at	period homolog 3 (Drosophila)	Per3	1.7	0.014737701	0.059749991
1397904_at	Transcribed locus	---	1.4	0.014811455	0.059975087
1396208_at	gamma-glutamyltransferase-like activity 1	Ggtla1	1.5	0.014859604	0.060110861
1374205_at	transcription factor Pur-beta	pur-beta	1.4	0.014868138	0.060130594
1377908_at	Transcribed locus	---	1.3	0.014885141	0.06017462
1374178_at	Transcribed locus	---	1.4	0.014886341	0.06017462
1373651_at	Transcribed locus	---	1.9	0.014894497	0.060192797
1388681_at	SAR1 gene homolog B (S. cerevisiae)	Sar1b	1.3	0.014912955	0.060247945
1376990_at	Similar to HCDI protein (predicted)	RGD1309307_predicted	1.3	0.014918528	0.060247945
1368004_at	mitochondrial ribosomal protein L23	Mrpl23	1.3	0.01491935	0.060247945
1367837_at	proteasome (prosome, macropain) subunit, alpha type 4	Psma4	1.3	0.014938048	0.060279935
1385887_at	Cut-like 1 (Drosophila)	Cutl1	1.6	0.01494461	0.060291621
1398905_at	ATPase, H transporting, lysosomal V1 subunit G1 (predicted)	Atp6v1g1_predicted	1.3	0.015021467	0.060525868
1375185_at	importin 7 (predicted)	Ipo7_predicted	1.4	0.015025527	0.060525868
1389191_at	vacuolar protein sorting 36 (yeast) (predicted)	Vps36_predicted	1.3	0.015026586	0.060525868
1390513_at	similar to ras homolog gene family, member f	LOC687609	1.5	0.015031132	0.060525868
1387883_a_at	thymosin, beta 4	Tmsb4x	1.5	0.015078731	0.060683866
1381162_at	Similar to septin 10 isoform 1	LOC309891	1.3	0.015137753	0.060891602
1376067_at	---	---	1.2	0.015157532	0.060942572
1374280_at	cerebellin 2 precursor protein	Cbln2	1.8	0.015157835	0.060942572
1367580_at	ribosomal protein L10A	Rpl10a	1.3	0.01518527	0.061023045
1373415_at	Transcribed locus	---	1.3	0.015189764	0.061023737
1382198_at	pumilio 2 (Drosophila)	Pum2	1.4	0.015202938	0.061049296
1375859_a_at	Transcribed locus	---	1.2	0.015247019	0.061196431
1372714_at	similar to RIKEN cDNA 8430437G11	RGD1307778	1.4	0.015274619	0.0612773
1389996_at	NIMA (never in mitosis gene a)-related expressed kinase 1 (predicted)	Nek1_predicted	1.8	0.015312799	0.061400516
1397602_at	Similar to RIKEN cDNA 5033405K12 (predicted)	RGD1311593_predicted	1.6	0.015363012	0.061541845
1391701_at	Transcribed locus	---	1.8	0.01539041	0.061612448
1375938_at	coiled-coil domain containing 12 (predicted)	Ccdc12_predicted	1.4	0.015391874	0.061612448
1397949_at	Membrane protein, palmitoylated 6 (MAGUK p55 subfamily member 6) (predicted)	Mpp6_predicted	2.1	0.015408921	0.061641742
1373214_at	KDEL (Lys-Asp-Glu-Leu) containing 1	Kdelc1	1.4	0.015410436	0.061641742
1374421_at	bromodomain adjacent to zinc finger domain protein 1B	Baz1b	1.3	0.015532817	0.062070887
1391649_a_at	Transcribed locus	---	1.8	0.01554372	0.062099369
1385334_at	Transcribed locus	---	2.2	0.015619461	0.062356526
1393938_at	Transcribed locus	---	1.4	0.015628229	0.062374082

1390896_at	Transcribed locus	---	1.5	0.015639027	0.062374082
1368083_at	cyclin H	Ccnh	1.4	0.015693448	0.062539676
1393512_at	Transcribed locus	---	1.5	0.015719167	0.062602627
1384497_at	Kruppel-like factor 7 (ubiquitous) (predicted)	Klf7_predicted	1.7	0.015729661	0.062620792
1388630_at	Transcribed locus	---	1.4	0.015735149	0.062620792
1377640_at	Transcribed locus	---	1.6	0.015739492	0.062622922
1398349_at	adenylate kinase 2	Ak2	1.4	0.015781958	0.06274635
1382031_at	Transcribed locus	---	2.3	0.015795281	0.062784143
1389000_at	similar to jumonji domain containing 1B	LOC682469	1.3	0.01582535	0.062888467
1382921_at	SDA1 domain containing 1	Sdad1	1.5	0.015846671	0.062947401
1371900_at	CUG triplet repeat, RNA binding protein 1	Cugbp1	1.3	0.015847835	0.062947401
1393015_at	similar to hypothetical protein FLJ14146	RGD1310587	1.6	0.015854465	0.062958531
1390886_at	Coiled-coil domain containing 39 (predicted)	Ccdc39_predicted	1.7	0.015904115	0.063140448
1370927_at	procollagen, type XII, alpha 1	Col12a1	1.6	0.015952003	0.063269484
1385038_at	similar to hedgehog-interacting protein (predicted)	RGD1564108_predicted	1.5	0.015962794	0.063272185
1377551_at	Transcribed locus	---	2.2	0.015963802	0.063272185
1383685_at	HEAT repeat containing 1 (predicted)	Heatr1_predicted	1.3	0.015964225	0.063272185
1383347_at	REV1-like (S. cerevisiae) (predicted)	Rev1l_predicted	1.5	0.016004695	0.063417304
1375369_at	similar to hypothetical protein FLJ20154	RGD1305793	1.3	0.016022432	0.063460875
1393145_at	follicular lymphoma variant translocation 1 (predicted)	Fvt1_predicted	1.3	0.016027266	0.063460875
1374034_at	cysteinyl-tRNA synthetase (predicted)	Cars_predicted	1.4	0.016071622	0.063590578
1368063_a_at	splicing factor YT521-B	Yt521	1.3	0.016106519	0.063709748
1393330_at	---	---	1.5	0.016109487	0.063709748
1382363_at	membrane protein, palmitoylated 5 (MAGUK p55 subfamily member 5) (predicted)	Mpp5_predicted	1.4	0.016122688	0.063723786
1371664_at	paxillin	Pxn	1.4	0.016134366	0.063746829
1377607_at	mitogen activated protein kinase kinase kinase 3 (predicted)	Map3k3_predicted	1.5	0.0161558	0.063789202
1381610_at	Transcribed locus	---	1.8	0.016156256	0.063789202
1383295_at	---	---	1.4	0.016160418	0.063789202
1370088_at	sperm autoantigenic protein 17	Spa17	1.6	0.01619029	0.063854403
1377810_at	Ral GEF with PH domain and SH3 binding motif 2	Ralgps2	1.3	0.016196533	0.063854403
1377835_at	similar to dedicator of cytokinesis 8	LOC499337	1.7	0.016205971	0.063870855
1378114_at	Wolf-Hirschhorn syndrome candidate 1-like 1 (predicted)	Whsc1l1_predicted	1.2	0.016208472	0.063870855
1395121_at	Transcribed locus	---	1.8	0.016270141	0.064053944
1376744_at	Similar to RNA-binding region (RNP1, RRM) containing 3	LOC686263	1.6	0.016271864	0.064053944
1372621_at	similar to 2610027C15Rik protein (predicted)	RGD1308876_predicted	1.9	0.016304499	0.064141689
1390803_at	HIV TAT specific factor 1 (predicted)	Htatsf1_predicted	1.4	0.016351526	0.06428057
1372870_at	KDEL (Lys-Asp-Glu-Leu) endoplasmic reticulum protein retention receptor 3 (predicted)	Kdelr3_predicted	1.5	0.016356749	0.064284549
1389263_at	retinoic acid induced 14	Rai14	1.7	0.016371999	0.064284549
1397574_at	Transcribed locus	---	1.3	0.016373129	0.064284549
1383539_at	Transcribed locus	---	1.3	0.016390979	0.064284549
1383041_x_at	---	---	2.3	0.016394852	0.064284549
1389778_a_at	transcription elongation factor B (SIII), polypeptide 3	Tceb3	1.4	0.016400306	0.064284549
1380214_at	Transcribed locus	---	1.6	0.016403347	0.064284549
1380347_at	Transcribed locus	---	1.5	0.01646853	0.064493899
1388344_at	similar to RIKEN cDNA 1110005A23	RGD1305692	1.6	0.016493781	0.064562044
1373152_at	protease, serine, 23	Prss23	1.6	0.016526742	0.064656133
1384558_at	placenta-specific 9 (predicted)	Plac9_predicted	1.5	0.016529611	0.064656133
1367547_at	similar to zinc finger protein 198	LOC305913	1.3	0.01654938	0.064710212
1382814_at	odd Oz/ten-m homolog 3 (Drosophila) (predicted)	Odz3_predicted	1.7	0.01657188	0.06476437
1373770_at	---	---	1.3	0.016573032	0.06476437
1396654_at	CUG triplet repeat, RNA binding protein 2	Cugbp2	1.8	0.016672256	0.065112926
1376879_at	RWD domain containing 4A	Rwdd4a	1.8	0.016687718	0.065119714

1372355_at	Transcribed locus	---	1.3	0.016733866	0.065222424
1374508_at	---	---	1.4	0.016741068	0.065235035
1390115_at	SEC63-like (S. cerevisiae) (predicted)	Sec63_predicted	1.3	0.016760803	0.065281007
1378257_at	---	---	1.3	0.016768307	0.065287709
1385471_at	DEAD (Asp-Glu-Ala-Asp) box polypeptide 27	Ddx27	1.4	0.016774432	0.065287709
1382021_at	Transcribed locus	---	1.7	0.016801241	0.065376581
1382040_at	glutamyl-prolyl-tRNA synthetase	Eprs	1.6	0.016829752	0.065451434
1388897_at	WD repeat domain 5	Wdr5	1.4	0.016831023	0.065451434
1384062_at	Phosphatidylinositol 4-kinase, catalytic, beta polypeptide	Pik4cb	1.7	0.016836038	0.065451434
1389482_at	similar to EST AA792894	RGD1306495	1.2	0.016863428	0.06551203
1374333_at	similar to RIKEN cDNA 1110007C09 (predicted)	RGD1306058_predicted	1.3	0.016879826	0.065558294
1393743_at	Transcribed locus	---	1.4	0.016886527	0.065568839
1375059_at	similar to zinc finger protein 652 (predicted)	RGD1566329_predicted	1.7	0.016911886	0.065651806
1389194_at	Transcribed locus, moderately similar to XP_001153858.1 hypothetical protein isoform 3 [Pan troglodytes]	---	2.1	0.016926911	0.065679129
1373015_at	ring finger protein 11 (predicted) /// similar to RING finger protein 11 (NEDD4 WW domain-binding protein 2) (Sid 1669)	LOC683844 /// Rnf11_predicted	1.4	0.016940692	0.065713213
1395645_at	---	---	1.8	0.017034242	0.06595556
1380940_at	Similar to sorbin and SH3 domain containing 1 isoform 3	LOC686098	2.0	0.017137121	0.066275891
1383435_at	sodium channel, voltage-gated, type III, beta	Scn3b	1.7	0.01714904	0.066279578
1392523_at	Son cell proliferation protein	Son	1.8	0.01715699	0.066290386
1386901_at	similar to fatty acid translocase/CD36 (predicted)	RGD1562323_predicted	1.4	0.017196821	0.06641308
1383907_at	Transcribed locus	---	1.4	0.017231382	0.066488708
1373669_at	glucosamine-6-phosphate deaminase 2 (predicted)	Gnpda2_predicted	1.4	0.017239038	0.066498055
1390380_at	similar to Ski protein (predicted)	RGD1565591_predicted	2.0	0.017395176	0.067012556
1379347_at	Similar to RIKEN cDNA 1700023M03	RGD1305457	1.4	0.01739789	0.067012556
1399081_at	pellino homolog 1 (Drosophila) /// similar to pellino protein (predicted)	Peli1 /// RGD1564594_predicted	1.9	0.017400938	0.067012556
1386818_at	Transcribed locus	---	1.6	0.017432723	0.067103539
1371763_at	similar to RIKEN cDNA 4931406C07	RGD1309534	1.3	0.017438436	0.067105598
1398373_at	UDP-Gal:betaGlcNAc beta 1,3-galactosyltransferase, polypeptide 3	B3galt3	1.4	0.017441417	0.067105598
1389355_at	immediate early response 5	Ier5	3.4	0.017530121	0.067415345
1373020_at	similar to mitochondria-associated granulocyte macrophage CSF signaling molecule	LOC679907 /// LOC687579	1.2	0.017581275	0.067573984
1374154_at	Similar to splicing factor, arginine/serine-rich 2, interacting protein	LOC312030	1.3	0.017581947	0.067573984
1371552_at	Transcribed locus	---	1.3	0.017587329	0.067573984
1374587_at	Transcribed locus	---	1.4	0.017587806	0.067573984
1383271_at	coiled-coil domain containing 59 (predicted)	Ccdc59_predicted	1.7	0.017631561	0.06769466
1372133_at	related RAS viral (r-ras) oncogene homolog 2	Rras2	1.6	0.017643551	0.067724883
1387606_at	fibroblast growth factor 2	Fgf2	1.5	0.017668693	0.06778975
1387022_at	aldehyde dehydrogenase family 1, member A1	Aldh1a1	2.0	0.017687194	0.067829089
1383167_at	Transcribed locus	---	1.9	0.017794623	0.06817749
1372438_at	nitrilase family, member 2	Nit2	1.3	0.017860945	0.068399726
1397549_at	centaurin, gamma 1	Centg1	1.3	0.017875751	0.068408718
1387870_at	zinc finger protein 36	Zfp36	1.5	0.017890392	0.06841692
1383400_at	similar to TBP-associated factor 4	LOC682097	1.7	0.017899344	0.068420423
1382720_at	Transcribed locus	---	1.3	0.017904285	0.068420423
1372987_at	Transcribed locus, weakly similar to NP_002218.2 kinase 1 [Homo sapiens]	---	1.3	0.01791258	0.068422228
1372371_at	Transcribed locus	---	1.3	0.017983234	0.068602268

1394849_at	zinc finger and BTB domain containing 20 (predicted)	Zbtb20_predicted	1.9	0.017990097	0.068606799
1372456_at	mitochondrial ribosomal protein S31 (predicted)	Mrps31_predicted	1.4	0.018012194	0.068659229
1371539_at	nucleolar protein family A, member 2 (predicted)	Nola2_predicted	1.3	0.018022696	0.06868334
1371782_at	nipsnap homolog 3A (C. elegans)	Nipsnap3a	1.3	0.018036211	0.068718925
1373131_at	Pbx/knotted 1 homeobox	Pknox1	1.6	0.018056619	0.068773766
1389294_at	cytoplasmic FMR1 interacting protein 1 (predicted)	Cyfip1_predicted	1.3	0.018063149	0.068773766
1396063_at	DEK oncogene (DNA binding)	Dek	3.0	0.018117879	0.068950227
1382130_at	protocadherin 19 (predicted)	Pcdh19_predicted	1.9	0.018193519	0.069174065
1367958_at	abl-interactor 1	Abi1	1.3	0.018273383	0.06942957
1374418_at	---	---	1.3	0.018285137	0.069446364
1378178_at	SMC6 structural maintenance of chromosomes 6-like 1 (yeast) (predicted)	Smc6l1_predicted	1.4	0.018289189	0.069446364
1376834_at	ataxia telangiectasia mutated homolog (human)	Atm	1.3	0.01829047	0.069446364
1378595_at	---	---	1.5	0.018296204	0.069452104
1367893_a_at	chloride channel CLIC-like 1	Clcc1	1.6	0.018308119	0.069481298
1380701_at	Sperm specific antigen 2 (predicted)	Ssfa2_predicted	1.6	0.018336546	0.069573127
1395327_at	Transcribed locus	---	1.6	0.018361639	0.069620156
1393922_at	similar to CDNA sequence BC020077 (predicted)	RGD1561825_predicted	1.5	0.01838209	0.069681634
1388530_at	CDC42 small effector 1	Cdc42se1	1.4	0.018451922	0.069881928
1390586_at	similar to Jumonji/ARID domain-containing protein 1C (SmcX protein) (predicted)	RGD1560601_predicted	1.7	0.018481442	0.06994541
1373473_a_at	nucleosome assembly protein 1-like 1	Nap1l1	1.3	0.018491584	0.069967697
1367494_at	similar to CGI-35 protein (predicted)	RGD1310899_predicted	1.4	0.018526984	0.070033329
1380323_at	Transcribed locus	---	1.7	0.018528353	0.070033329
1394756_at	Similar to CG10084-PA	RGD1308297	1.9	0.018530219	0.070033329
1369966_a_at	ribosomal protein S24	Rps24	1.7	0.018582788	0.070215873
1383821_at	Interleukin enhancer binding factor 3	Ilf3	1.2	0.018666342	0.070402201
1379062_at	---	---	2.2	0.018686645	0.070462619
1373867_at	similar to Retinoblastoma-binding protein 2 (RBBP-2)	LOC312678	1.3	0.01869219	0.07046737
1390387_at	Transcribed locus	---	1.4	0.018700555	0.070482752
1398382_at	Transcribed locus	---	1.4	0.018715324	0.070507459
1383320_at	lymphocyte protein tyrosine kinase	Lck	1.3	0.018794578	0.070756059
1374415_at	polymerase (RNA) III (DNA directed) polypeptide E (predicted)	Polr3e_predicted	1.3	0.018812058	0.070789459
1383999_at	Transcribed locus	---	1.4	0.018830482	0.070825455
1379719_at	Transcribed locus	---	1.6	0.018834541	0.070825455
1378313_at	Similar to methylenetetrahydrofolate dehydrogenase (NAD) (EC 1.5.1.15) / methenyltetrahydrofolate cyclohydrolase (EC 3.5.4.9) precursor - mouse (predicted)	RGD1310879_predicted	1.9	0.018840266	0.070830787
1379793_at	similar to RIKEN cDNA 3110031B13	RGD1305356	2.0	0.018874164	0.070925044
1369304_at	6-pyruvoyl-tetrahydropterin synthase	Pts	1.4	0.018883689	0.070925044
1373341_at	similar to RIKEN cDNA 2410018C17 (predicted)	RGD1563315_predicted	1.4	0.018901735	0.070925044
1384483_at	RGD1561157 (predicted)	RGD1561157_predicted	1.7	0.018904412	0.070925044
1368014_at	prostaglandin E synthase	Ptges	1.3	0.018904782	0.070925044
1370414_at	Rab38, member of RAS oncogene family	Rab38	1.5	0.018919171	0.070932877
1375872_at	Transcribed locus	---	1.4	0.018939634	0.070993415
1389862_at	similar to hypothetical protein MGC3731	RGD1305327	1.3	0.018976322	0.071114727
1379538_at	centromere protein J (predicted)	Cenpj_predicted	1.5	0.019030112	0.071218949
1374100_at	---	---	1.2	0.019059946	0.071314376
1380041_at	Transcribed locus	---	1.4	0.019078685	0.071335808
1372706_at	hexosaminidase B	Hexb	1.5	0.019109755	0.071435739
1373763_at	zinc finger protein 297B	Zfp297b	1.3	0.01920178	0.071698269
1398768_at	retinoblastoma binding protein 7	Rbbp7	1.3	0.019242248	0.0718139

1382955_at	G protein-coupled receptor 126 (predicted)	Gpr126_predicted	1.3	0.019245846	0.0718139
1372006_at	Lysyl oxidase-like 2 (predicted)	Loxl2_predicted	1.6	0.019262523	0.071859827
1374705_at	collagen, type IV, alpha 5 (predicted)	Col4a5_predicted	2.2	0.019305376	0.07198342
1386861_at	H2A histone family, member Z	H2afz	1.3	0.019313158	0.07198342
1377121_at	discs, large homolog 5 (Drosophila) (predicted)	Dlg5_predicted	1.5	0.019343237	0.072079193
1388351_at	similar to chromosome 13 open reading frame 12 (predicted)	RGD1305831_predicted	1.3	0.019395685	0.072193421
1376533_at	bromodomain adjacent to zinc finger domain, 2B (predicted)	Baz2b_predicted	1.5	0.019395837	0.072193421
1375892_at	ELAV (embryonic lethal, abnormal vision, Drosophila)-like 1 (Hu antigen R) (predicted)	Elavl1_predicted	1.4	0.01942715	0.07227726
1379023_at	Thioredoxin domain containing 12 (endoplasmic reticulum)	Txndc12	2.2	0.019433844	0.072284537
1371480_at	RGD1561797 (predicted) /// hypothetical protein LOC686524	LOC686524 /// RGD1561797_predicted	1.4	0.019443161	0.072284537
1396947_at	Leucine-rich repeat-containing G protein-coupled receptor 4	Lgr4	1.7	0.019456197	0.072303552
1389014_at	pre-B-cell colony enhancing factor 1	Pbef1	1.4	0.019468845	0.072320773
1389289_at	Ewing sarcoma breakpoint region 1	Ewsr1	1.4	0.019478207	0.072336319
1389011_at	similar to RIKEN cDNA 1110014D18	RGD1305156	1.4	0.019498502	0.072379004
1374323_at	BRCA2 and CDKN1A interacting protein (predicted)	Bccip_predicted	1.4	0.019504131	0.072380212
1389563_at	transmembrane protein 1 (predicted)	Tmem1_predicted	1.3	0.019516611	0.072380924
1383357_a_at	Transcribed locus	---	1.3	0.019565096	0.072501148
1389896_at	Transcribed locus	---	1.3	0.019613328	0.072592264
1378094_at	Similar to pleckstrin homology-like domain, family B, member 2	LOC685611	1.6	0.019640395	0.072659744
1378146_at	Transcribed locus	---	1.4	0.019653442	0.072679573
1391901_at	Transcribed locus	---	1.3	0.019668657	0.072715233
1393101_at	F-box and leucine-rich repeat protein 10	Fbxl10	1.5	0.019676758	0.072716122
1375034_at	lysophospholipase 3	Lypla3	1.4	0.01967774	0.072716122
1374027_at	Transcribed locus	---	1.3	0.019689221	0.072742209
1380744_at	Transcribed locus	---	1.6	0.019724589	0.072803649
1394779_at	Transcribed locus	---	1.4	0.019749803	0.072818822
1372556_at	hypothetical protein LOC502374	LOC502374	1.4	0.019784865	0.072899074
1390972_at	Tp53rk binding protein	Tprkb	1.5	0.019817348	0.072986058
1376492_at	similar to chromosome 16 open reading frame 28 (predicted)	RGD1565247_predicted	1.3	0.019874739	0.073131927
1384355_at	plexin A2 (predicted)	Plxna2_predicted	1.4	0.019907375	0.073219253
1389454_at	programmed cell death 5 (predicted)	Pdcd5_predicted	1.6	0.019949588	0.07329856
1394243_at	spermine synthase /// similar to spermine synthase (predicted) /// similar to spermine synthase	LOC683008 /// LOC690027 /// RGD1559938_predicted /// Sms	2.5	0.019950036	0.07329856
1385476_x_at	stimulated by retinoic acid gene 6 homolog (mouse)	Stra6	1.4	0.019950078	0.07329856
1370432_at	POU domain, class 3, transcription factor 1	Pou3f1	4.0	0.019952306	0.07329856
1373101_at	phosphatidylinositol glycan, class K	Pigk	1.3	0.019966105	0.073304129
1391370_at	Transcribed locus	---	1.5	0.020040354	0.073518654
1384295_at	heat shock 70kDa protein 14	Hspa14	1.3	0.020042415	0.073518654
1372820_at	Transcribed locus	---	1.4	0.020064622	0.073523058
1394160_at	transmembrane protein 2 (predicted)	Tmem2_predicted	1.8	0.020069375	0.073523058
1376611_at	polymerase (DNA directed), alpha 1	Pola1	1.4	0.020096174	0.073600951
1393879_at	Transcribed locus	---	1.6	0.020103548	0.073611565
1373348_at	similar to Protein deltex-1 (Deltex-1) (Deltex1) (mDTX1) (FXI-T1)	LOC687424	1.3	0.020185908	0.073798136
1378720_at	GLI-Kruppel family member GLI1	Gli1	1.4	0.020225189	0.073925312
1381840_at	Electron-transfer-flavoprotein, beta polypeptide	Etfb	1.8	0.020253585	0.073996217
1387202_at	intercellular adhesion molecule 1	Icam1	1.5	0.02029263	0.074073055
1397179_at	similar to DIP13 alpha (predicted)	RGD1309388_predicted	1.3	0.020306653	0.074107795
1386893_at	granulin	Grn	1.5	0.020322227	0.074130875
1371583_at	RNA binding motif protein 3	Rbm3	1.6	0.020326498	0.074130875

1378578_at	Transcribed locus	---	1.6	0.020386576	0.074300556
1371766_at	Transcribed locus	---	1.3	0.020464243	0.074457056
1382258_at	Rho-guanine nucleotide exchange factor (predicted)	Rgnef_predicted	1.6	0.020470258	0.074457056
1383202_at	similar to U6 snRNA-associated Sm-like protein LSm6 (Sm protein F) (predicted) /// similar to U6 snRNA-associated Sm-like protein LSm6	LOC684624 /// RGD1561937_predicted	1.5	0.020494379	0.074528308
1394630_at	LIM domain only 4	Lmo4	1.6	0.020526931	0.074613693
1367476_at	signal recognition particle 14 (predicted)	Srp14_predicted	1.3	0.020543548	0.074657596
1368252_at	kelch repeat and BTB (POZ) domain containing 10	Kbtbd10	3.1	0.020552218	0.074672603
1370585_a_at	protein kinase C, beta 1	Prkcb1	1.3	0.020583852	0.074725507
1391211_at	Atpase, class VI, type 11C (predicted)	Atp11c_predicted	1.4	0.020584234	0.074725507
1383905_at	zinc finger protein 216 (predicted)	Zfp216_predicted	1.4	0.020589494	0.074725507
1373927_at	high mobility group box 2-like 1 (predicted)	Hmgb2l1_predicted	1.4	0.02059669	0.074735131
1371420_at	92Aa-Protein	LOC494529	1.4	0.020703427	0.075072742
1370826_at	nucleosome assembly protein 1-like 1	Nap1l1	1.3	0.020773858	0.075278346
1386394_at	---	---	1.7	0.020822412	0.075430429
1389580_at	SWI/SNF related, matrix associated, actin dependent regulator of chromatin, subfamily a, member 3 (predicted)	Smarca3_predicted	1.3	0.020988191	0.075887836
1372814_at	similar to RIKEN cDNA 2010005O13	RGD1310623	1.3	0.021007439	0.075940739
1393811_at	similar to putative repair and recombination helicase RAD26L (predicted)	RGD1561537_predicted	1.4	0.021100155	0.076208904
1383841_at	similar to PWWP domain containing 2	LOC683932	2.0	0.021126705	0.076288044
1392231_at	serine/threonine kinase 4 (predicted)	Stk4_predicted	1.3	0.021131537	0.076288746
1382045_at	TBC1 domain family, member 15	Tbc1d15	1.4	0.021150634	0.076310277
1380202_at	---	---	2.1	0.021155793	0.076310277
1392832_at	similar to angiopoietin-like 1	LOC679942 /// LOC684489	2.1	0.021190216	0.076399972
1374687_at	---	---	1.4	0.02119615	0.076404619
1374111_at	similar to Pinin	LOC368070	1.3	0.021236309	0.076501139
1380195_at	ATPase, Ca++ transporting, plasma membrane 1	Atp2b1	1.5	0.021262603	0.076577028
1393085_at	similar to hypothetical protein BC018453	RGD1307700	1.5	0.021333712	0.076763701
1388874_at	metastasis suppressor 1 (predicted)	Mtss1_predicted	1.2	0.02133777	0.076763701
1382642_at	zinc finger protein 281	Zfp281	1.7	0.021384427	0.076881106
1382789_at	Transcribed locus	---	1.5	0.021394792	0.076899044
1383245_at	MAP3K12 binding inhibitory protein 1 (predicted)	Mbip_predicted	1.3	0.021427903	0.076953306
1376102_at	transmembrane BAX inhibitor motif containing 1	Tmbim1	1.4	0.021433412	0.076956289
1375367_at	PDZ and LIM domain 2	Pdlim2	1.5	0.021439389	0.076960951
1373957_at	reelin	Reln	1.6	0.02145507	0.077000436
1393046_at	similar to Protein C20orf20	RGD1308612	1.7	0.021501276	0.077115784
1390404_at	laminin, alpha 2 (predicted)	Lama2_predicted	1.6	0.021507951	0.077122909
1375652_at	signal sequence receptor, gamma	Ssr3	1.2	0.021530166	0.077185738
1382447_at	---	---	1.4	0.021546261	0.077226609
1373079_at	Transcribed locus	---	1.2	0.021572225	0.077282279
1397855_at	Transcribed locus	---	1.8	0.021575889	0.077282279
1395633_at	Similar to F-box only protein 3 isoform 1	LOC690634	1.2	0.021592536	0.077325068
1372718_at	similar to anaphase promoting complex subunit 13	LOC683931 /// LOC685029	1.5	0.021617515	0.077397667
1378275_at	UPF3 regulator of nonsense transcripts homolog A (yeast)	Upf3a	1.5	0.021629122	0.077422371
1395871_at	similar to topoisomerase (DNA) II beta	LOC361100	1.4	0.021744358	0.077733363
1395306_at	similar to polybromo-1 (predicted)	RGD1565549_predicted	1.2	0.021755601	0.077749115
1373453_at	similar to myocardial ischemic preconditioning upregulated protein 2 (predicted)	RGD1565589_predicted	1.3	0.021758219	0.077749115
1368703_at	PDZ and LIM domain 5	Pdlim5	1.4	0.021775171	0.077775898
1373944_at	Transcribed locus	---	1.4	0.021798971	0.077844001
1381124_at	Transcribed locus	---	2.0	0.021814853	0.077866906

1382139_at	similar to hypothetical protein FLJ12994 (predicted)	RGD1308795_predicted	1.3	0.021822835	0.077876934
1379915_at	Similar to sorbin and SH3 domain containing 1 isoform 3	LOC686098	1.5	0.021827132	0.077876934
1371792_at	---	---	1.4	0.02183403	0.07788465
1378103_at	similar to DNA (cytosine-5)-methyltransferase 3A (Dnmt3a) (DNA methyltransferase MmuIIIA) (DNA MTase MmuIIIA) (M.MmuIIIA) (predicted)	RGD1305891_predicted	2.0	0.021840087	0.077889364
1398989_at	Son cell proliferation protein	Son	1.3	0.021850832	0.07789178
1391466_at	---	---	1.3	0.021867216	0.077901662
1379987_at	Similar to RIKEN cDNA 6330416G13 gene (predicted)	RGD1304595_predicted	1.2	0.021882499	0.077939226
1381683_at	chromodomain helicase DNA binding protein 2 (predicted)	Chd2_predicted	1.5	0.021911453	0.078008567
1374281_at	similar to KIAA1259 protein (predicted)	RGD1310969_predicted	1.3	0.021960977	0.078106477
1377156_at	similar to transcription factor 7-like 2, T-cell specific, HMG-box /// similar to Transcription factor 7-like 2 (HMG box transcription factor 4) (T-cell-specific transcription factor 4) (TCF-4) (hTCF-4)	LOC679869 /// LOC683733	1.5	0.021992744	0.078179523
1379195_s_at	ligase I, DNA, ATP-dependent	Lig1	1.7	0.02202177	0.078265791
1393836_at	microphthalmia-associated transcription factor	Mitf	1.8	0.022039952	0.078297781
1370817_at	Sec11-like 3 (S. cerevisiae)	Sec11l3	1.4	0.022052876	0.078297781
1368702_at	PRKC, apoptosis, WT1, regulator	Pawr	2.7	0.022057596	0.078297781
1371986_at	Transcribed locus	---	1.4	0.022059333	0.078297781
1376860_at	ion transporter protein	Nritp	1.5	0.022082061	0.078337105
1393110_at	Mpv17 transgene, kidney disease mutant-like (predicted)	Mpv17l_predicted	1.3	0.022128295	0.078457901
1394200_at	heat shock 70kDa protein 2	Hspa2	1.4	0.022135306	0.078465845
1371856_at	CDNA clone MGC:93828 IMAGE:7110880	---	1.5	0.022217402	0.078704529
1389255_at	cadherin 5 (predicted)	Cdh5_predicted	1.5	0.022218192	0.078704529
1392692_at	Transcribed locus	---	1.9	0.02222178	0.078704529
1373822_at	similar to Chain A, Solution Structure Of The Bola-Like Protein From Mus Musculus (predicted) /// similar to RIKEN cDNA 1110025L05 (predicted)	RGD1309998_predicted /// RGD1565363_predicted	1.5	0.022321634	0.079024156
1380874_at	Dullard homolog (Xenopus laevis)	Dullard	1.8	0.02235833	0.079102991
1384154_at	WW domain binding protein 4	Wbp4	2.3	0.022369648	0.079126016
1375024_at	similar to RIKEN cDNA 5230400J09 (predicted)	RGD1559623_predicted	1.3	0.022381917	0.079152392
1376369_at	trophinin associated protein (tastin) (predicted)	Troap_predicted	1.2	0.022475609	0.079398371
1385739_at	Transcribed locus	---	1.4	0.022523115	0.079514959
1392995_at	similar to zinc finger protein 322a	LOC680201 /// LOC684943	1.5	0.022534384	0.079537673
1372620_at	acidic (leucine-rich) nuclear phosphoprotein 32 family, member E	Anp32e	1.4	0.022548383	0.079570006
1382550_at	---	---	1.3	0.022568839	0.079608035
1376195_at	spindlin /// similar to spindlin	LOC682571 /// Spin	1.4	0.022579637	0.079629047
1396249_at	Transcribed locus	---	1.4	0.022586056	0.079634609
1368226_at	Nucleoside 2-deoxyribosyltransferase domain containing protein RGD620382	RGD620382	1.4	0.02259464	0.079634717
1390661_at	WD repeat domain 20	Wdr20	1.3	0.022648355	0.07973193
1396036_at	Ral GEF with PH domain and SH3 binding motif 2	Ralgps2	2.1	0.022736061	0.079974921
1373017_at	succinate-Coenzyme A ligase, GDP-forming, beta subunit	Suclg2	1.3	0.022741456	0.079976795
1388591_at	DNA cross-link repair 1B, PSO2 homolog (S. cerevisiae)	Dclre1b	1.4	0.02281602	0.080175526
1375958_at	similar to TSC22 domain family protein 4 (TSC22-related-inducible leucine zipper protein 2)	LOC686226	2.1	0.022816374	0.080175526
1368655_at	proteoglycan peptide core protein	Pgsg	1.5	0.022820369	0.080175526
1373611_at	interleukin 17 receptor (predicted)	Il17r_predicted	1.3	0.022822338	0.080175526
1384501_at	Transcribed locus	---	1.7	0.02284429	0.080235507

1372760_at	CDNA clone IMAGE:7321089	---	1.4	0.022851908	0.080245128
1370827_at	cytochrome b5 reductase 4	Cyb5r4	1.5	0.022861945	0.080263238
1382001_at	similar to Myeloid/lymphoid or mixed-lineage leukemia protein 3 homolog (Histone-lysine N-methyltransferase, H3 lysine-4 specific MLL3)	LOC502710 /// LOC679252	1.4	0.022884516	0.080325333
1390757_at	similar to cell division cycle 2-like 1 (predicted)	RGD1566355_predicted	2.1	0.022950996	0.080489976
1397217_at	CUG triplet repeat, RNA binding protein 2	Cugbp2	1.6	0.022972764	0.08054914
1376398_at	Transcribed locus	---	1.8	0.022985979	0.080552003
1383573_at	serologically defined colon cancer antigen 33 (predicted)	Sdccag33_predicted	2.0	0.022986043	0.080552003
1379810_at	small inducible cytokine subfamily E, member 1	Scye1	2.0	0.022990762	0.080552003
1376193_at	Kohjirin	Chrdl1	1.6	0.023139891	0.080930287
1398734_at	Ankyrin 3, epithelial	Ank3	1.5	0.023140512	0.080930287
1396581_at	SCY1-like 2 (S. cerevisiae) (predicted)	Scyl2_predicted	1.4	0.023204406	0.081107783
1370176_at	trafficking protein, kinesin binding 2	Trak2	1.4	0.02320841	0.081107783
1369705_at	X transporter protein 3	Xtrp3	2.1	0.023238911	0.081153631
1373193_at	Hypothetical protein LOC690089	LOC690089	1.6	0.023252693	0.081184525
1375020_at	Ras and Rab interactor 3 (predicted)	Rin3_predicted	1.3	0.023272166	0.081218031
1392493_at	Potassium channel tetramerisation domain containing 1	Kctd1	1.3	0.023279279	0.081218341
1367491_at	apoptosis inhibitor 5 (predicted)	Api5_predicted	1.3	0.02328213	0.081218341
1377263_at	hepatitis A virus cellular receptor 2	Havcr2	1.5	0.023305076	0.081238142
1382891_at	similar to ubiquitin specific protease 27, X chromosome (predicted)	RGD1564628_predicted	1.7	0.023308912	0.081238142
1367657_at	B-cell translocation gene 1, anti-proliferative	Btg1	1.8	0.023312502	0.081238142
1379909_at	G kinase anchoring protein 1	Gkap1	1.7	0.023361516	0.081357234
1388607_at	SMAD specific E3 ubiquitin protein ligase 2 (predicted)	Smurf2_predicted	1.7	0.023467894	0.081624005
1376760_at	similar to GC-rich sequence DNA-binding factor homolog	LOC681004 /// LOC683880	2.1	0.023516283	0.081740454
1372034_at	similar to hypothetical protein MGC29390 (predicted)	RGD1310490_predicted	2.1	0.023528144	0.081764401
1369967_at	citrate synthase	Cs	1.3	0.023544612	0.081789697
1399001_at	hypothetical LOC294030	RGD1305481	1.4	0.023578758	0.08188839
1376710_at	Hypothetical protein LOC691783	LOC691783	1.5	0.023645606	0.082085884
1383939_at	Transcribed locus	---	1.6	0.023670922	0.082134846
1374609_at	RAB3 GTPase activating protein subunit 2	Rab3gap2	1.4	0.02367469	0.082134846
1368212_at	similar to splicing factor p54	LOC502603	1.6	0.023684335	0.082150978
1370073_at	DnaJ (Hsp40) homolog, subfamily C, member 3	Dnajc3	1.6	0.023720464	0.082214732
1380713_at	Transcribed locus	---	1.6	0.023727207	0.082214732
1393097_at	---	---	1.4	0.023757025	0.082298977
1376935_at	---	---	1.3	0.023789468	0.082376666
1375981_a_at	suppressor of variegation 3-9 homolog 1 (Drosophila) (predicted)	Suv39h1_predicted	1.5	0.023819344	0.082445405
1394961_at	Transcribed locus	---	1.4	0.023825895	0.08245073
1376182_at	Transcribed locus, weakly similar to NP_066564.1 general control of amino-acid synthesis 5-like 2 [Homo sapiens]	---	1.8	0.023998316	0.082960132
1388581_at	hematological and neurological expressed sequence 1	Hn1	1.4	0.024010002	0.082969013
1388672_at	Transcribed locus	---	1.2	0.024012687	0.082969013
1398317_at	bisphosphate 3'-nucleotidase 1	Bpnt1	1.3	0.024025385	0.082983943
1380191_s_at	thioredoxin-like 1	Txnl1	1.3	0.024051362	0.08303879
1377148_at	---	---	1.4	0.024065253	0.083069314
1379386_at	similar to B99 protein (predicted)	RGD1563164_predicted	1.2	0.024100245	0.083131787
1373881_at	Rho, GDP dissociation inhibitor (GDI) beta	Arhgdib	1.6	0.024103568	0.083131787
1399141_at	CDC like kinase 4	Clk4	1.4	0.024115868	0.083156769
1383500_at	ribosome binding protein 1 homolog 180kDa (dog) (predicted)	Rrbp1_predicted	1.8	0.024149806	0.083238896
1395110_at	Transcribed locus	---	1.9	0.024166233	0.083260619

1377909_at	Transcribed locus	---	1.9	0.024184879	0.083307411
1371867_at	Transcribed locus	---	1.3	0.024199809	0.083341385
1391654_at	---	---	1.4	0.024212676	0.083350794
1392846_at	Zinc finger, MYND domain containing 11	Zmynd11	1.9	0.024221874	0.083365009
1378691_at	---	---	1.4	0.024283492	0.08354212
1368204_at	ligase I, DNA, ATP-dependent	Lig1	1.2	0.024335411	0.083650751
1374667_at	CDNA clone IMAGE:7302289	---	1.3	0.024357059	0.083674378
1391904_at	Transcribed locus	---	1.6	0.024357546	0.083674378
1389716_at	centrosomal protein 27 (predicted)	Cep27_predicted	1.3	0.024420236	0.083854705
1397652_at	Transcribed locus	---	1.9	0.024463888	0.083921194
1382582_at	Transcribed locus	---	1.4	0.02446511	0.083921194
1372785_at	ash1 (absent, small, or homeotic)-like (Drosophila) (predicted)	Ash1l_predicted	1.8	0.024478338	0.083949064
1370940_at	tight junction protein 2	Tjp2	1.6	0.024531528	0.084078886
1372622_at	similar to RIKEN cDNA 6530418L21 (predicted)	RGD1560778_predicted	1.5	0.024640959	0.084366047
1383724_at	Heparan sulfate 2-O-sulfotransferase 1	Hs2st1	1.4	0.024676995	0.084454269
1399077_at	similar to Metaxin 1, isoform 2	LOC295241	1.2	0.024696201	0.084502417
1382146_at	tetraspanin 6	Tspan6	1.3	0.024760561	0.084687402
1373683_at	fyn proto-oncogene	Fyn	1.4	0.024780379	0.084737561
1398309_at	phosphatidylinositol glycan, class L	Pigl	1.3	0.024881044	0.085033005
1394585_at	Similar to RIKEN cDNA G430041M01 (predicted)	RGD1562563_predicted	1.8	0.025022184	0.08544005
1383951_at	similar to hypothetical protein FLJ31951 (predicted)	RGD1309561_predicted	1.3	0.025024095	0.08544005
1376321_at	family with sequence similarity 38, member A (predicted)	Fam38a_predicted	1.7	0.025026953	0.08544005
1372298_at	thioredoxin domain containing 4 (endoplasmic reticulum)	Txndc4	1.3	0.025032563	0.08544005
1383491_at	interferon stimulated exonuclease gene 20-like 1 (predicted)	Isg20l1_predicted	1.3	0.025044018	0.085461413
1384523_at	similar to postmeiotic segregation increased 1	MGC72584	1.6	0.025090619	0.085575671
1371864_at	Transcribed locus	---	1.5	0.025093109	0.085575671
1376437_at	similar to Der1-like domain family, member 2	LOC687738 /// LOC691956	1.5	0.025149217	0.085749238
1372338_at	similar to CG9240-PA	LOC687395	1.3	0.025165958	0.085760187
1382056_at	similar to splicing factor p54	LOC502603	1.7	0.025191007	0.085785028
1379362_at	Transcribed locus	---	1.6	0.025199794	0.085797186
1395940_at	1-acylglycerol-3-phosphate O-acyltransferase 5 (lysophosphatidic acid acyltransferase, epsilon) (predicted)	Agpat5_predicted	1.8	0.025218084	0.085841688
1385931_at	hook homolog 3 (Drosophila)	Hook3	1.6	0.025358714	0.08619851
1397874_at	Similar to U7 snRNA-associated Sm-like protein Lsm11 (predicted)	RGD1565523_predicted	1.3	0.025385843	0.086238626
1373648_at	similar to Protein C6orf142 homolog	LOC681849	1.5	0.025433415	0.086344709
1397197_at	Serine incorporator 3	Serinc3	1.5	0.025436341	0.086344709
1392341_at	Calpain, small subunit 1	Capns1	1.2	0.025443059	0.086344709
1398573_at	zinc finger protein 307	Zfp307	1.3	0.025489478	0.086479155
1370913_at	Best5 protein	Best5	2.3	0.025565364	0.086667457
1372538_at	solute carrier family 25 (mitochondrial carrier, peroxisomal membrane protein), member 17 (predicted)	Slc25a17_predicted	1.3	0.025566057	0.086667457
1383880_at	integrin alpha V (predicted)	Itgav_predicted	1.3	0.025636332	0.086869877
1374870_at	Procollagen, type XXVII, alpha 1	Col27a1	1.6	0.025675591	0.086971889
1371379_at	similar to Brain protein 44 (predicted)	RGD1563422_predicted	1.4	0.02567758	0.086971889
1377844_at	zinc binding alcohol dehydrogenase, domain containing 1	Zadh1	1.3	0.0256823	0.086971889
1385101_a_at	similar to RIKEN cDNA 0610011N22 gene	RGD735106	1.4	0.025711276	0.086998315
1398992_at	Transcribed locus	---	1.4	0.025714868	0.086998315
1382190_at	MAS-related GPR, member F	Mrgprf	1.4	0.025727082	0.08701605
1368199_at	nucleoporin 88	Nup88	1.3	0.02580275	0.087200254
1388828_at	SID1 transmembrane family, member 2 (predicted)	Sidt2_predicted	1.4	0.02583502	0.087291374
1381262_at	Pre-B-cell leukemia transcription factor interacting protein 1	Pbxip1	1.6	0.025879885	0.087405679

1379460_at	similar to RIKEN cDNA 3110007P09 (predicted)	RGD1564957_predicted	1.4	0.025885387	0.087405679
1377282_at	Protein phosphatase 3, catalytic subunit, alpha isoform	Ppp3ca	1.3	0.025890106	0.087405679
1383621_at	Membrane-bound transcription factor peptidase, site 2	Mbtps2	1.3	0.025926281	0.087465891
1384882_at	HECT domain containing 2 (predicted)	Hectd2_predicted	1.4	0.025977447	0.087573024
1385856_at	RIO kinase 1 (yeast)	Riok1	1.4	0.025982269	0.087573024
1383138_at	slingshot homolog 3 (Drosophila)	Ssh3	1.4	0.026028321	0.08771027
1375343_at	Transcribed locus	---	1.5	0.026069093	0.087776875
1397545_at	Transcribed locus	---	1.2	0.026069433	0.087776875
1384505_at	Transcribed locus	---	1.4	0.026121637	0.087862717
1393008_at	CAMP-regulated phosphoprotein 19	Arpp19	1.3	0.026146941	0.087911873
1376966_at	Transcribed locus	---	1.3	0.026184308	0.088019516
1374809_at	Transcribed locus	---	1.3	0.026199678	0.088045102
1383989_at	SRY-box containing gene 4 (predicted)	Sox4_predicted	1.2	0.02622027	0.088086398
1377704_at	male-specific lethal 2-like 1 (Drosophila) (predicted)	Msl2l1_predicted	1.7	0.02624403	0.088148213
1373225_at	Transcribed locus	---	1.5	0.026379817	0.088526481
1367500_at	similar to 26 proteasome complex subunit DSS1 (Deleted in split hand/split foot protein 1) (Split hand/foot deleted protein 1 homolog)	LOC679014 /// LOC680532	1.5	0.026383361	0.088526481
1385033_at	Transcribed locus	---	1.4	0.026383561	0.088526481
1379444_at	Transcribed locus	---	2.2	0.026426245	0.088614797
1389668_at	spindle pole body component 25 homolog (S. cerevisiae)	Spbc25	1.4	0.026472038	0.088678633
1368273_at	mitogen-activated protein kinase 6	Mapk6	1.2	0.026573885	0.088965449
1373002_at	mitochondrial ribosomal protein S9	Mrps9	1.3	0.026585219	0.088985282
1367709_at	CD63 antigen	Cd63	1.7	0.026611352	0.089054631
1376087_at	similar to RIKEN cDNA 1300010M03 (predicted)	RGD1561189_predicted	1.5	0.026649983	0.089165771
1368042_a_at	high mobility group box 1 /// similar to High mobility group protein 1 (HMG-1) (predicted) /// similar to Hmgb1 protein (predicted) /// hypothetical protein LOC678705 /// similar to High mobility group protein 1 (HMG-1) (High mobility group protein B1) (Amphoterin) (Heparin-binding protein p30)	Hmgb1 /// LOC678705 /// LOC679451 /// LOC679571 /// LOC680054 /// LOC680765 /// LOC680968 /// LOC681718 /// LOC685520 /// RGD1562312_predicted /// RGD1563012_predicted /// RGD1563786_predicted	1.7	0.026674123	0.08922597
1383965_at	similar to SMP3 mannosyltransferase /// similar to nuclear cap binding protein subunit 2	LOC684506 /// LOC689116	1.2	0.026683586	0.08922597
1395836_at	Odd Oz/ten-m homolog 2 (Drosophila)	Odz2	1.7	0.026722072	0.089316121
1391372_at	Dolichol-phosphate (beta-D) mannosyltransferase 1 (predicted)	Dpm1_predicted	1.7	0.026762799	0.089434075
1371395_at	chromobox homolog 3 (HP1 gamma homolog, Drosophila)	Cbx3	1.2	0.026835894	0.089660124
1373768_at	BMS1-like, ribosome assembly protein (yeast)	Bms1l	1.3	0.026917152	0.089859698
1396737_at	similar to RIKEN cDNA 4931400A14 (predicted)	RGD1307234_predicted	2.2	0.026917481	0.089859698
1368353_at	glial fibrillary acidic protein	Gfap	2.6	0.026964025	0.0899603
1373810_at	phospholipase A2, group XIIA (predicted)	Pla2g12a_predicted	1.5	0.026990312	0.09002974
1390848_at	RNA binding motif protein 19 (predicted) /// similar to RNA binding motif protein 19	LOC687727 /// Rbm19_predicted	1.4	0.027042594	0.090167562
1395699_at	RIO kinase 3 (yeast) (predicted)	Riok3_predicted	1.3	0.027114494	0.090315756
1375067_at	Transcribed locus	---	1.2	0.027171754	0.090416693
1386938_at	alanyl (membrane) aminopeptidase	Anpep	1.5	0.027172781	0.090416693

1392550_at	similar to dapper 1 (predicted)	RGD1564008_predicted	1.7	0.02717778	0.090416693
1397944_at	Transcribed locus	---	2.4	0.027204112	0.090463047
1371745_at	hypothetical LOC300361	RGD1310669	1.3	0.02722079	0.090468288
1389110_at	---	---	1.5	0.027245026	0.090517115
1396641_at	---	---	1.4	0.027251991	0.090517115
1388571_at	Synaptogyrin 2	Syngr2	1.4	0.027311892	0.090629923
1387897_at	cyclic nucleotide phosphodiesterase 1	Cnp1	1.5	0.027344985	0.090697785
1377998_at	coproporphyrinogen oxidase	Cpox	1.5	0.027377284	0.090786606
1385349_at	similar to centrin 4	LOC684095	1.3	0.027398951	0.090840142
1392548_at	similar to mKIAA1604 protein (predicted)	RGD1565385_predicted	1.4	0.027459516	0.090959828
1375630_at	similar to NHP2-like protein 1 (High mobility group-like nuclear protein 2 homolog 1) ([U4/U6.U5] tri-snRNP 15.5 kDa protein) (OTK27)	MGC72932	1.2	0.027462701	0.090959828
1373804_at	forkhead box P1	Foxp1	1.6	0.027496188	0.091015758
1388316_at	RGD1566320 (predicted)	RGD1566320_predicted	1.3	0.027514126	0.091052814
1368332_at	guanylate nucleotide binding protein 2	Gbp2	3.5	0.02752776	0.091052814
1376438_at	Ubiquitin-conjugating enzyme E2E 2 (UBC4/5 homolog, yeast)	Ube2e2	1.7	0.0275327	0.091052814
1370284_at	ATP synthase, H+ transporting, mitochondrial F1 complex, epsilon subunit	Atp5e	1.3	0.027543847	0.091054542
1392853_at	---	---	1.3	0.027616813	0.09119481
1392488_at	similar to hypothetical protein MGC32132 (predicted)	RGD1307915_predicted	1.3	0.02764146	0.091257874
1387446_at	core 1 UDP-galactose:N-acetylgalactosamine-alpha-R beta 1,3-galactosyltransferase	C1galt1	1.5	0.027675188	0.091332563
1368279_at	myeloid/lymphoid or mixed-lineage leukemia,translocated to, 3 (trithorax homolog, Drosophila)	Mllt3	1.6	0.027718409	0.091420172
1384519_at	SEC63-like (S. cerevisiae) (predicted)	Sec63_predicted	1.3	0.027790676	0.091592188
1374465_at	ubiquitously expressed transcript	Uxt	1.4	0.027794017	0.091592188
1394109_at	Thrombospondin 1	Thbs1	2.5	0.027807484	0.091592188
1396150_at	claudin 1	Cldn1	1.9	0.027809545	0.091592188
1388038_at	attractin	Atrn	1.4	0.02785715	0.091730612
1373696_at	---	---	1.4	0.027873394	0.091747364
1383214_at	USP6 N-terminal like (predicted)	Usp6nl_predicted	1.7	0.027912842	0.09185883
1372901_at	similar to RIKEN cDNA 8030451K01 (predicted)	RGD1565414_predicted	1.4	0.028032357	0.092119875
1376457_at	Transcribed locus	---	1.6	0.028041908	0.092119875
1378620_at	Transcribed locus	---	1.3	0.028048172	0.092119875
1385909_at	Transcribed locus	---	1.3	0.028058313	0.092134785
1390492_a_at	ubiquitin specific protease 40	Usp40	1.3	0.028065687	0.092135453
1375068_at	similar to surfeit 5 isoform b (predicted)	RGD1564893_predicted	1.7	0.02812371	0.092239039
1370262_at	metadherin	Mtdh	1.9	0.028136493	0.092262568
1391915_at	heat shock 70kDa protein 9A (predicted) /// similar to Stress-70 protein, mitochondrial precursor (75 kDa glucose-regulated protein) (GRP 75) (Peptide-binding protein 74) (PBP74) (P66 MOT) (Mortalin) /// similar to Stress-70 protein, mitochondrial precursor (75 kDa glucose regulated protein) (GRP 75) (Peptide-binding protein 74) (PBP74) (MTHSP70) (Mortalin)	Hspa9a_predicted /// LOC295778 /// LOC500372 /// LOC502646	1.8	0.028157609	0.092305275
1372068_at	zinc finger, MYND domain containing 11	Zmynd11	1.3	0.028160741	0.092305275
1382092_at	similar to PHD finger protein 20-like 1 isoform 1	LOC314964	1.5	0.028181639	0.092352942
1376591_a_at	hypothetical protein LOC686471 /// hypothetical protein LOC690263	LOC686471 /// LOC690263	1.3	0.028193379	0.09235704
1372309_at	LUC7-like 2 (S. cerevisiae) (predicted)	Luc7l2_predicted	1.4	0.028250781	0.09248982
1394086_at	SUMO1/sentrin specific protease 7 (predicted)	Senp7_predicted	1.3	0.028274009	0.092536112
1387905_at	J domain protein 1	LOC619393	1.2	0.028276173	0.092536112

1391014_at	zinc finger, MYND domain containing 19	Zmynd19	1.5	0.028309359	0.092626285
1379868_at	pre-B-cell leukemia transcription factor interacting protein 1	Pbxip1	1.3	0.0283188	0.092638747
1379941_at	similar to vacuolar protein sorting 13C protein (predicted)	RGD1560364_predicted	1.3	0.028352283	0.092692973
1367686_at	ribosome associated membrane protein 4	RAMP4	1.4	0.02835826	0.092694089
1375442_at	M-phase phosphoprotein 10 (U3 small nucleolar ribonucleoprotein) (predicted)	Mphosph10_predicted	1.9	0.028375571	0.092732245
1378361_at	chromodomain helicase DNA binding protein 7 (predicted)	Chd7_predicted	2.2	0.02839183	0.092766949
1372533_at	similar to mKIAA0212 protein (predicted)	RGD1563633_predicted	1.2	0.028409501	0.092806252
1379059_at	---	---	1.6	0.02846218	0.092852485
1387262_at	Sjogren syndrome antigen B	Ssb	1.3	0.028496019	0.092904365
1398354_at	catenin (cadherin associated protein), alpha-like 1 (predicted)	Ctnnal1_predicted	1.4	0.028514883	0.092917696
1375396_at	pumilio 1 (Drosophila) (predicted)	Pum1_predicted	1.3	0.028518405	0.092917696
1384323_at	proteasome (prosome, macropain) 26S subunit, ATPase, 6	Psmc6	1.4	0.028522705	0.092917696
1380192_at	similar to F-box protein FBL2	RGD1308119	1.6	0.028579786	0.093048362
1369588_a_at	ATPase inhibitory factor 1	Atpif1	1.5	0.028585839	0.093049649
1380327_at	Glutamate receptor interacting protein 1	Grip1	1.3	0.028594731	0.093060179
1385641_at	---	---	1.3	0.028620193	0.093124616
1377069_at	---	---	2.0	0.028626637	0.09312716
1373445_at	nucleolar protein 8 (predicted)	Nol8_predicted	1.7	0.028653825	0.093197175
1374397_at	eukaryotic translation initiation factor 2, subunit 2 (beta)	Eif2s2	1.4	0.02866343	0.093209982
1371921_at	catenin (cadherin-associated protein), alpha 1	Catna1	1.3	0.028715275	0.09336012
1390305_at	protein kinase C binding protein 1	Prkcbp1	1.2	0.028746536	0.093432381
1375887_at	Transcribed locus	---	1.2	0.028748862	0.093432381
1372873_at	F-box protein 38 (predicted)	Fbxo38_predicted	1.3	0.028760503	0.09343329
1367590_at	RAN, member RAS oncogene family	Ran	1.2	0.028822347	0.093578753
1393821_at	similar to CG9047-PA, isoform A	LOC681715 /// LOC689576	1.4	0.028853624	0.093650055
1373011_at	hypothetical protein LOC619558	LOC619558	1.3	0.028857146	0.093650055
1373608_at	Transcribed locus	---	1.7	0.028868015	0.093653079
1384349_at	pogo transposable element with ZNF domain (predicted)	Pogz_predicted	1.3	0.028891313	0.093691704
1393390_at	Similar to src homology 2 domain-containing transforming protein D	LOC316507	1.4	0.028970699	0.093893609
1396425_at	Transcribed locus	---	1.6	0.028986065	0.093906399
1371771_at	perlecan	LOC313641	1.4	0.02904467	0.094022186
1399119_at	Transcribed locus	---	1.4	0.029164039	0.094334339
1374376_at	Rho-guanine nucleotide exchange factor (predicted)	Rgnef_predicted	1.6	0.029219609	0.094469205
1384214_a_at	cyclin T2 (predicted)	Ccnt2_predicted	1.4	0.02923911	0.094469205
1388397_at	EBNA1 binding protein 2	Ebna1bp2	1.4	0.029240195	0.094469205
1389136_at	protein-kinase, interferon-inducible double stranded RNA dependent inhibitor, repressor of (P58 repressor) (predicted)	Prkrir_predicted	1.4	0.029264443	0.094510419
1367993_at	restin (Reed-Steinberg cell-expressed intermediate filament-associated protein)	Rsn	1.5	0.029289577	0.094554458
1383948_at	similar to TPR repeat-containing protein KIAA1043	LOC304558	1.7	0.029300856	0.094572306
1383579_at	Transcribed locus, moderately similar to XP_577744.1 similar to IKEN cDNA 6720480D16 [Rattus norvegicus]	---	1.4	0.029372667	0.094766895
1381696_at	Similar to hypothetical protein FLJ31606 (predicted)	RGD1561507_predicted	1.5	0.029435606	0.094858317
1372162_at	acyl-CoA synthetase short-chain family member 1 (predicted) /// similar to acetyl-CoA synthetase 2-like	Acss1_predicted /// LOC681886	1.3	0.029605236	0.095222012
1388553_at	hypothetical LOC290577	LOC290577	1.2	0.029606357	0.095222012
1383929_at	Transcribed locus	---	1.3	0.029621748	0.095252884

1379235_x_at	similar to cell division cycle 45 homolog (S. cerevisiae)-like	LOC287961	1.3	0.029654252	0.095282895
1389092_at	interleukin 2 receptor, gamma (severe combined immunodeficiency)	Il2rg	1.6	0.029667439	0.095306648
1385765_at	similar to lin-9 homolog (C. elegans)	LOC360888	1.4	0.029690728	0.095332366
1376832_at	---	---	1.2	0.029692833	0.095332366
1380938_at	receptor interacting protein kinase 5	Ripk5	1.7	0.029719548	0.095399516
1368393_at	complement component 1, q subcomponent, receptor 1	C1qr1	1.3	0.029766982	0.095495869
1376447_at	similar to hypothetical protein A430110N23 (predicted)	RGD1565772_predicted	1.2	0.029809493	0.095576323
1384751_at	RNA binding motif protein 8 (predicted)	Rbm8_predicted	1.2	0.029835858	0.095642212
1392046_at	Transcribed locus	---	1.8	0.029903313	0.095810226
1391347_at	RAB8B, member RAS oncogene family	Rab8b	1.3	0.029917374	0.095819958
1389150_at	Transcribed locus	---	1.3	0.029931422	0.095819958
1393815_at	similar to NMDA receptor-regulated gene 2	LOC679306 /// LOC691379	1.4	0.029939987	0.095826602
1383842_at	Transcribed locus, moderately similar to NP_060559.2 repeat and zinc finger domain containing 1 [Homo sapiens]	---	1.3	0.029949192	0.095837414
1393053_at	mannosidase, alpha, class 1C, member 1 (predicted)	Man1c1_predicted	1.2	0.029969138	0.095882589

Supplementary table 4: Probe sets identified as down-regulated 10% FDR (p<0.03) and >1.2 fold in the L5 DRGs of SNT animals

Probe set ID	Gene Title	Gene Symbol	Fold increase	p value	FDR value
1383571_at	hypothetical protein LOC303515	LOC303515	0.3	5.12E-09	1.68E-05
1382965_at	Amphoterin induced gene and ORF 3	Amigo3	0.3	7.53E-08	5.44E-05
1376225_at	Calsenilin, presenilin binding protein, EF hand transcription factor	Csen	0.3	1.41E-07	5.82E-05
1374785_at	similar to CD69 antigen (p60, early T-cell activation antigen) (predicted)	RGD1565373_predicted	0.3	1.70E-07	6.64E-05
1377095_at	similar to ataxin 2-binding protein 1 isoform 2 (predicted)	RGD1560070_predicted	0.2	7.30E-07	0.000174055
1393980_at	Transcribed locus	---	0.3	8.38E-07	0.000190087
1388543_at	similar to HTPAP protein (predicted)	RGD1306289_predicted	0.3	1.09E-06	0.000210194
1390815_at	cyclin M1 (predicted)	Cnnm1_predicted	0.4	1.17E-06	0.000211994
1374659_at	similar to cyclic AMP-regulated phosphoprotein, 21 isoform 2 /// similar to protein phosphatase 1, regulatory (inhibitory) subunit 1C; thymocyte ARPP; DNA segment, Chr 9, Brigham & Womens Genetics 1012 expressed	LOC363153 /// RGD1307215	0.3	1.24E-06	0.000215529
1376203_at	similar to Rho guanine nucleotide exchange factor 4 isoform a	LOC301334	0.5	1.30E-06	0.000222668
1393933_at	sortilin-related receptor, LDLR class A repeats-containing	Sorl1	0.3	1.55E-06	0.000251478
1393404_at	reticulon 4 receptor-like 1	Rtn4rl1	0.2	1.64E-06	0.000258784
1391037_at	RGD1564491 (predicted)	RGD1564491_predicted	0.5	1.73E-06	0.0002692
1396265_at	Transcribed locus	---	0.4	1.79E-06	0.000272962
1374096_at	Transcribed locus	---	0.3	1.89E-06	0.000281227
1393793_at	Transcribed locus	---	0.5	2.10E-06	0.000292875
1388012_at	paired related homeobox protein-like 1	Prrxl1	0.3	2.12E-06	0.000292875
1394609_at	actin-binding LIM protein 2	Ablim2	0.5	2.13E-06	0.000292875
1377084_at	Transcribed locus	---	0.4	2.28E-06	0.000299931
1374386_at	Transcribed locus	---	0.3	2.67E-06	0.000333128
1383582_at	transmembrane protein 54	Tmem54	0.3	2.85E-06	0.000339959
1390070_at	---	---	0.4	3.02E-06	0.000349747
1379300_at	carbohydrate sulfotransferase 2 (predicted)	Chst2_predicted	0.5	3.04E-06	0.000349747
1383757_at	Transcribed locus	---	0.2	3.14E-06	0.000358774
1374271_at	---	---	0.4	3.22E-06	0.000361299
1368749_at	K+ voltage-gated channel, subfamily S, 1	Kcns1	0.2	3.45E-06	0.000375797
1392710_at	WD repeat and FYVE domain containing 1	Wdfy1	0.4	3.47E-06	0.000375797
1384741_at	Phospholipase A2, group III (predicted)	Pla2g3_predicted	0.3	3.58E-06	0.000377436
1376460_at	similar to ataxin 2-binding protein 1 isoform 2 (predicted)	RGD1560070_predicted	0.2	3.60E-06	0.000377436
1378136_at	---	---	0.4	3.65E-06	0.000378299
1393626_at	sortilin-related receptor, LDLR class A repeats-containing	Sorl1	0.2	3.72E-06	0.00038152
1391200_at	Transcribed locus	---	0.2	3.73E-06	0.00038152
1369155_at	contactin 4	Cntn4	0.3	3.79E-06	0.000384571
1398520_at	Transcribed locus	---	0.4	4.15E-06	0.000396667
1383370_at	BMP/retinoic acid-inducible neural-specific protein 2	Brinp2	0.5	4.42E-06	0.000411097
1380107_a_at	N-acetyltransferase 8-like (predicted)	Nat8l _predicted	0.3	4.47E-06	0.000411097
1372690_at	reticulon 4 receptor-like 1	Rtn4rl1	0.3	4.48E-06	0.000411097
1373740_at	---	---	0.4	4.51E-06	0.000411097
1374046_at	heparan sulfate (glucosamine) 3-O-sulfotransferase 2	Hs3st2	0.3	4.53E-06	0.000411097
1389511_s_at	---	---	0.5	4.65E-06	0.000417868
1371793_at	Acetylcholinesterase	Ache	0.3	5.08E-06	0.000444873
1376311_at	similar to netrin G1 (predicted)	RGD1563465_predicted	0.3	5.34E-06	0.000457557
1390652_at	Transcribed locus	---	0.4	5.40E-06	0.000457863
1391575_at	---	---	0.1	5.65E-06	0.000466955
1379517_at	regulator of G-protein signaling 6	Rgs6	0.5	6.22E-06	0.000486989
1385562_at	junctophilin 3 (predicted)	Jph3_predicted	0.4	6.65E-06	0.000505576
1376944_at	Prolactin receptor	Prlr	0.2	6.68E-06	0.000505576
1393022_at	ankyrin repeat domain 13 family, member D (predicted)	Ankrd13d_predicted	0.4	7.20E-06	0.00052578
1384771_at	---	---	0.3	7.26E-06	0.00052578

1378800_at	Rho GTPase activating protein 8	Arhgap8	0.3	7.44E-06	0.000536604
1382123_at	---	---	0.2	7.75E-06	0.0005521
1372884_at	like-glycosyltransferase (predicted)	Large_predicted	0.5	7.85E-06	0.000554362
1373312_at	Transcribed locus	---	0.5	8.00E-06	0.000556387
1390675_at	Transcribed locus	---	0.4	8.50E-06	0.000576069
1376486_at	SH3-domain GRB2-like endophilin B2	Sh3glb2	0.5	8.51E-06	0.000576069
1383418_at	a disintegrin and metallopeptidase domain 11 (predicted)	Adam11_predicted	0.4	9.05E-06	0.000607804
1394463_at	Transcribed locus, strongly similar to XP_507983.1 similar to PRO1933 [Pan troglodytes]	---	0.3	9.64E-06	0.000634742
1384165_at	Coagulation factor II (thrombin) receptor-like 2	F2rl2	0.3	9.65E-06	0.000634742
1382915_at	Transcribed locus	---	0.4	9.90E-06	0.000642629
1390710_x_at	sortilin-related receptor, LDLR class A repeats-containing	Sorl1	0.3	9.92E-06	0.000642629
1394522_at	Transcribed locus	---	0.4	1.00E-05	0.000644131
1385684_x_at	---	---	0.3	1.02E-05	0.00064801
1367745_at	diacylglycerol kinase zeta	Dgkz	0.4	1.09E-05	0.000675432
1368688_at	neurotensin receptor 2	Ntsr2	0.5	1.14E-05	0.000692122
1375053_at	Transcribed locus, strongly similar to XP_001061250.1 similar to Pbx/knotted 1 homeobox 2 [Rattus norvegicus]	---	0.5	1.14E-05	0.000692192
1377767_at	Transcribed locus	---	0.5	1.14E-05	0.000692192
1398645_at	Transcribed locus	---	0.5	1.17E-05	0.000706273
1384756_at	Solute carrier family 43, member 2 (predicted)	Slc43a2_predicted	0.4	1.19E-05	0.000710566
1390674_at	Transcribed locus	---	0.5	1.23E-05	0.00072225
1369144_a_at	potassium voltage gated channel, Shal-related family, member 3	Kcnd3	0.2	1.24E-05	0.00072225
1377457_a_at	sortilin-related receptor, LDLR class A repeats-containing	Sorl1	0.2	1.28E-05	0.000742663
1374510_at	Transcribed locus, moderately similar to XP_521059.2 hypothetical protein [Pan troglodytes]	---	0.5	1.29E-05	0.000742663
1394727_at	---	---	0.2	1.32E-05	0.000749734
1382439_at	integrin, beta 6	Itgb6	0.2	1.35E-05	0.000761055
1373693_at	G protein-coupled receptor, family C, group 5, member C	Gprc5c	0.5	1.39E-05	0.000775092
1376417_at	Transcribed locus	---	0.3	1.39E-05	0.000775092
1379776_at	Transcribed locus	---	0.3	1.40E-05	0.000776806
1390362_at	Transcribed locus	---	0.2	1.41E-05	0.000776806
1376156_at	hypothetical LOC316091	RGD1304827	0.5	1.43E-05	0.000782073
1377458_at	sortilin-related receptor, LDLR class A repeats-containing	Sorl1	0.4	1.45E-05	0.000791275
1388253_at	Stearoyl-Coenzyme A desaturase 2	Scd2	0.3	1.46E-05	0.000796096
1394786_at	sortilin-related receptor, LDLR class A repeats-containing	Sorl1	0.2	1.48E-05	0.000800821
1378624_at	Transcribed locus	---	0.3	1.49E-05	0.000804262
1385041_at	sortilin-related VPS10 domain containing receptor 3 (predicted)	Sorcs3_predicted	0.4	1.53E-05	0.000820233
1397016_at	Transcribed locus	---	0.5	1.56E-05	0.000830731
1376000_at	Transcribed locus	---	0.4	1.58E-05	0.000836801
1387934_at	brevican	Bcan	0.5	1.66E-05	0.000866701
1375667_at	Transcribed locus	---	0.5	1.67E-05	0.000867383
1370111_at	potassium intermediate/small conductance calcium-activated channel, subfamily N, member 2	Kcnn2	0.3	1.69E-05	0.000867383
1381499_at	Transcribed locus	---	0.2	1.70E-05	0.000872971
1378197_at	kinesin family member C2	KIFC2	0.4	1.76E-05	0.000886376
1371049_at	dihydropyrimidinase-like 4	Dpysl4	0.4	1.79E-05	0.00089819
1374370_at	---	---	0.3	1.80E-05	0.00089819
1377693_at	FERM and PDZ domain containing 1 (predicted)	Frmpd1_predicted	0.3	1.84E-05	0.000907641
1378163_at	Transcribed locus	---	0.3	1.88E-05	0.000918829
1368343_at	potassium voltage-gated channel, subfamily H (eag-related), member 2	Kcnh2	0.4	1.91E-05	0.000930303
1390477_at	Transcribed locus	---	0.2	1.93E-05	0.000939813
1373298_at	Transcribed locus	---	0.4	1.95E-05	0.000944836
1384648_at	Transcribed locus	---	0.3	1.95E-05	0.000944836

1376495_at	Transcribed locus	---	0.5	2.03E-05	0.000968411
1368351_at	sodium channel, voltage-gated, type 10, alpha polypeptide	Scn10a	0.2	2.06E-05	0.000972323
1373255_at	Transcribed locus	---	0.6	2.07E-05	0.000972323
1374363_at	similar to hypothetical protein FLJ30473	RGD1306028	0.4	2.07E-05	0.000972323
1378462_at	Transcribed locus	---	0.5	2.10E-05	0.000982555
1383994_at	---	---	0.4	2.14E-05	0.000996081
1391764_at	Transcribed locus	---	0.2	2.32E-05	0.001044751
1375044_at	Transcribed locus	---	0.6	2.34E-05	0.001044751
1371954_at	tensin /// similar to tensin	LOC301509 /// Tns	0.5	2.35E-05	0.001044751
1385889_at	Similar to hypothetical protein MGC52110 (predicted)	RGD1565095_predicted	0.3	2.39E-05	0.001049726
1392746_x_at	La ribonucleoprotein domain family, member 1 (predicted)	Larp1_predicted	0.5	2.40E-05	0.001049726
1387923_at	zinc finger protein 179	Zfp179	0.2	2.42E-05	0.001053164
1389522_at	pleckstrin homology domain containing, family A member 6 (predicted)	Plekha6_predicted	0.6	2.42E-05	0.001053164
1374787_at	Transcribed locus	---	0.2	2.47E-05	0.001064804
1368839_at	Wolfram syndrome 1 homolog (human)	Wfs1	0.5	2.53E-05	0.001089032
1391919_at	similar to transcription elongation regulator 1-like (predicted)	RGD1562437_predicted	0.4	2.56E-05	0.001093364
1383580_at	---	---	0.3	2.58E-05	0.001094871
1379396_at	engulfment and cell motility 1, ced-12 homolog (C. elegans) (predicted)	Elmo1_predicted	0.5	2.64E-05	0.001117143
1390196_at	Synapsin I	Syn1	0.3	2.67E-05	0.001119838
1373322_at	Transcribed locus	---	0.5	2.70E-05	0.001128523
1373751_at	Transcribed locus	---	0.5	2.70E-05	0.001128523
1373656_at	Transcribed locus	---	0.5	2.73E-05	0.001133504
1384996_at	Transcribed locus	---	0.5	2.74E-05	0.001133504
1373331_at	Similar to Leucine rich repeat and sterile alpha motif containing 1 (predicted)	RGD1564403_predicted	0.5	2.76E-05	0.001133504
1394402_at	---	---	0.3	2.87E-05	0.001171025
1393971_at	Transcribed locus	---	0.4	2.89E-05	0.001175409
1368789_at	acid phosphatase, prostate	Acpp	0.4	2.91E-05	0.001176405
1369149_at	LIM motif-containing protein kinase 1	Limk1	0.6	2.92E-05	0.001176405
1376764_at	CDNA clone IMAGE:7319904	---	0.6	2.93E-05	0.001176405
1390227_at	hypothetical protein LOC678810 /// hypothetical protein LOC685448	LOC678810 /// LOC685448	0.3	2.93E-05	0.001176718
1374269_at	Transcribed locus	---	0.4	3.00E-05	0.001193299
1390751_at	---	---	0.4	3.03E-05	0.001199774
1374035_at	Rad and gem related GTP binding protein 2	Rem2	0.2	3.04E-05	0.001199774
1387554_at	UDP-N-acetyl-alpha-D-galactosamine:polypeptide N-acetylgalactosaminyltransferase 5	Galnt5	0.3	3.10E-05	0.001212796
1369159_at	androgen receptor	Ar	0.4	3.13E-05	0.001224223
1373949_at	ATPase type 13A2 (predicted)	Atp13a2_predicted	0.4	3.19E-05	0.001236544
1398298_at	5-hydroxytryptamine (serotonin) receptor 1D	Htr1d	0.3	3.22E-05	0.00124752
1393004_at	similar to mKIAA1045 protein (predicted)	RGD1559864_predicted	0.3	3.29E-05	0.001254225
1370375_at	glutaminase 2 (liver, mitochondrial)	Gls2	0.5	3.47E-05	0.001302935
1393262_at	Transcribed locus	---	0.3	3.54E-05	0.001318357
1373987_at	Kv channel-interacting protein 2	Kcnip2	0.3	3.60E-05	0.001329385
1376111_at	---	---	0.5	3.72E-05	0.00135381
1391902_at	Phosphoglycerate mutase 2	Pgam2	0.6	3.76E-05	0.001364509
1380865_at	Transcribed locus	---	0.4	3.82E-05	0.00137356
1383220_at	Kv channel interacting protein 4	Kcnip4	0.2	3.83E-05	0.001374201
1388735_at	Transcribed locus	---	0.4	3.93E-05	0.001397654
1390334_at	similar to hypothetical protein DKFZp434I2117 (predicted)	RGD1308215_predicted	0.4	3.96E-05	0.001404139
1394575_at	guanine nucleotide binding protein, alpha stimulating, olfactory type	Gnal	0.5	4.31E-05	0.001504327
1371052_at	noggin	Nog	0.3	4.37E-05	0.001516999
1390123_at	transmembrane protein 45b	Tmem45b	0.3	4.42E-05	0.001526634
1373985_at	Similar to KIAA1183 protein (predicted)	RGD1560435_predicted	0.4	4.48E-05	0.001543115
1373125_at	kinesin light chain 2 (predicted)	Klc2_predicted	0.4	4.56E-05	0.001557006
1395014_at	Transcribed locus	---	0.3	4.56E-05	0.001557006

1382824_at	RUN and TBC1 domain containing 2 (predicted)	Rutbc2_predicted	0.5	4.61E-05	0.001567855
1368174_at	EGL nine homolog 3 (C. elegans)	Egln3	0.3	4.64E-05	0.001573247
1387881_at	potassium channel, subfamily V, member 1	Kcnv1	0.2	4.65E-05	0.001575176
1392206_at	similar to solute carrier family 35, member F3 (predicted)	RGD1561109_predicted	0.4	4.69E-05	0.001582928
1395289_at	similar to Vacuolar protein sorting 26 homolog (VPS26 protein homolog)	LOC300472	0.6	4.71E-05	0.0015857
1386333_at	Transcribed locus	---	0.4	4.81E-05	0.001600387
1398182_at	Transcribed locus	---	0.4	4.81E-05	0.001600387
1384080_at	Transcribed locus	---	0.4	4.83E-05	0.001602838
1390358_at	calcium channel, voltage-dependent, alpha 2/delta 3 subunit	Cacna2d3	0.5	4.85E-05	0.001606539
1384865_at	Similar to leucine zipper, down-regulated in cancer 1 (predicted)	RGD1565644_predicted	0.6	4.92E-05	0.001624191
1370306_at	peroxisomal biogenesis factor 14	Pex14	0.4	4.99E-05	0.001646062
1377163_at	inhibin beta-B	Inhbb	0.3	5.05E-05	0.001662145
1369165_at	transient receptor potential cation channel, subfamily C, member 3	Trpc3	0.5	5.15E-05	0.001683622
1382147_at	similar to CG14446-PA	LOC363915	0.3	5.25E-05	0.001707117
1384169_a_at	Vav2 oncogene (predicted)	Vav2_predicted	0.5	5.25E-05	0.001707117
1391728_at	similar to mab-21-like 2	LOC680102 /// LOC683881	0.5	5.51E-05	0.001745623
1375247_at	---	---	0.6	5.52E-05	0.001745623
1382374_at	---	---	0.4	5.54E-05	0.001745623
1391563_at	similar to melanoma associated antigen (mutated) 1-like 1 (predicted)	RGD1565148_predicted	0.3	5.54E-05	0.001745623
1367965_at	solute carrier family 9, member 1	Slc9a1	0.5	5.62E-05	0.001759986
1382842_at	Cyclin H	Ccnh	0.5	5.63E-05	0.001759986
1383931_at	Protein tyrosine phosphatase, receptor type, K, extracellular region	Ptprk	0.4	5.68E-05	0.001767498
1397497_at	Transcribed locus	---	0.4	5.68E-05	0.001767498
1376041_at	epsin 3	Epn3	0.4	5.71E-05	0.001769703
1369000_at	neurotrophic tyrosine kinase, receptor, type 1	Ntrk1	0.3	5.73E-05	0.001769703
1393074_at	Similar to hypothetical protein FLJ38984 (predicted)	RGD1563072_predicted	0.6	5.78E-05	0.001781154
1382821_at	protein tyrosine phosphatase, receptor type, f polypeptide (PTPRF), interacting protein (liprin), alpha 3	Ppfia3	0.5	5.88E-05	0.001801909
1373691_at	Transcribed locus	---	0.6	5.94E-05	0.001811848
1380459_at	BTB (POZ) domain containing 14A	Btbd14a	0.5	5.95E-05	0.001811848
1369722_a_at	xylosyltransferase II	Xylt2	0.6	5.98E-05	0.00181593
1369783_a_at	neuregulin 1	Nrg1	0.4	6.14E-05	0.001855729
1398099_at	---	---	0.5	6.15E-05	0.001855729
1368425_at	CASK interacting protein 1	Caskin1	0.4	6.20E-05	0.001863489
1394446_at	Transcribed locus	---	0.5	6.31E-05	0.001889412
1395461_at	Transcribed locus	---	0.6	6.33E-05	0.001893019
1386439_at	---	---	0.6	6.47E-05	0.001921205
1367957_at	regulator of G-protein signalling 3	Rgs3	0.5	6.54E-05	0.001938239
1377942_at	Transcribed locus	---	0.3	6.55E-05	0.001938864
1373398_at	tripartite motif protein 37 (predicted)	Trim37_predicted	0.5	6.59E-05	0.00194332
1376060_at	Transcribed locus	---	0.6	6.59E-05	0.00194332
1369158_at	calcium-sensing receptor	Casr	0.3	6.67E-05	0.001962866
1383413_at	Gup1, glycerol uptake/transporter homolog (yeast) (predicted)	Gup1_predicted	0.3	6.88E-05	0.002000394
1378198_at	oligophrenin 1 (predicted)	Ophn1_predicted	0.6	6.97E-05	0.002010193
1388173_at	potassium voltage-gated channel, Shal-related family, member 1	Kcnd1	0.4	7.02E-05	0.002010193
1393349_x_at	Vav2 oncogene (predicted)	Vav2_predicted	0.5	7.04E-05	0.002014971
1394545_at	spire homolog 2 (Drosophila) (predicted)	Spire2_predicted	0.4	7.16E-05	0.002038885
1381222_at	---	---	0.5	7.31E-05	0.002069202
1398623_at	cholinergic receptor, nicotinic, beta polypeptide 4	Chrnb4	0.3	7.37E-05	0.002078119
1370517_at	neuronal pentraxin 1	Nptx1	0.2	7.57E-05	0.002123916
1383499_at	---	---	0.2	7.71E-05	0.002157244
1392927_at	progestin and adipoQ receptor family member IV	Paqr4	0.4	7.80E-05	0.002177047
1395255_at	Transcribed locus	---	0.4	7.91E-05	0.00220097

1371522_at	similar to mKIAA0945 protein (predicted)	RGD1563127_predicted	0.6	7.99E-05	0.002215207
1370595_a_at	Kv channel interacting protein 4	Kcnip4	0.5	8.01E-05	0.002215606
1370624_at	coagulation factor II (thrombin) receptor-like 2	F2rl2	0.3	8.18E-05	0.002251458
1368987_at	---	---	0.2	8.23E-05	0.002260751
1376172_at	ATP-binding cassette, sub-family G (WHITE), member 4	Abcg4	0.6	8.30E-05	0.002267697
1374168_at	Transcribed locus	---	0.4	8.31E-05	0.002267697
1382201_at	unc-5 homolog C (C. elegans)	Unc5c	0.5	8.31E-05	0.002267697
1383566_at	Transcribed locus	---	0.2	8.40E-05	0.002279288
1372881_at	Transcribed locus	---	0.5	8.46E-05	0.002293258
1370666_at	dynamin 3	Dnm3	0.3	8.56E-05	0.002298812
1371034_at	one cut domain, family member 1	Onecut1	0.4	8.56E-05	0.002298812
1373435_at	fragile X mental retardation gene 2, autosomal homolog (predicted)	Fxr2h_predicted	0.5	8.70E-05	0.002315223
1388423_at	hypothetical protein MGC:15854	RGD1302996	0.4	8.88E-05	0.002347992
1379336_at	Transcribed locus	---	0.5	8.99E-05	0.002362838
1396276_at	Transcribed locus	---	0.3	9.02E-05	0.002362838
1370962_at	castration induced prostatic apoptosis-related protein 1	Cipar1	0.4	9.03E-05	0.002362838
1383995_at	---	---	0.5	9.03E-05	0.002362838
1397459_at	Transcribed locus	---	0.5	9.05E-05	0.002362838
1395536_at	Transcribed locus	---	0.4	9.17E-05	0.002377389
1384286_at	Transcribed locus	---	0.5	9.18E-05	0.002377389
1370259_a_at	parathyroid hormone receptor 1	Pthr1	0.3	9.24E-05	0.002386892
1375431_at	similar to CG31122-PA (predicted)	RGD1306941_predicted	0.4	9.37E-05	0.002399451
1373110_at	Transcribed locus	---	0.4	9.39E-05	0.002400957
1385912_at	dual specificity phosphatase-like 15 (predicted)	Dusp15_predicted	0.3	9.66E-05	0.002451996
1382569_at	progestin and adipoQ receptor family member IX (predicted)	Paqr9_predicted	0.4	9.76E-05	0.002473373
1368407_at	heparanase	Hpse	0.3	9.81E-05	0.002477476
1381369_at	leucine-rich repeat LGI family, member 3 (predicted)	Lgi3_predicted	0.3	9.86E-05	0.002486336
1396459_at	Insulin related protein 2 (islet 2)	Isl2	0.3	9.87E-05	0.002486479
1370125_at	hyaluronan and proteoglycan link protein 1	Hapln1	0.4	9.92E-05	0.002488416
1372226_at	Transcribed locus, strongly similar to XP_001076245.1 similar to SLIT-ROBO Rho GTPase-activating protein 3 (srGAP3) (srGAP2) (WAVE-associated Rac GTPase-activating protein) (WRP) (Rho-GTPase-activating protein 14) [Rattus norvegicus]	---	0.6	9.94E-05	0.002488416
1373414_at	similar to Brain specific membrane-anchored protein precursor	RGD1305557	0.5	9.97E-05	0.002489015
1393000_at	---	---	0.5	9.97E-05	0.002489015
1398649_at	SLIT and NTRK-like family, member 3 (predicted)	Slitrk3_predicted	0.3	0.000100959	0.002508411
1373559_at	---	---	0.3	0.000101176	0.002510008
1393299_at	dipeptidylpeptidase 10	Dpp10	0.2	0.000101794	0.002519242
1389772_at	Transcribed locus	---	0.6	0.000102161	0.002523031
1368124_at	dual specificity phosphatase 5	Dusp5	0.4	0.000103255	0.002546229
1393712_at	Transcribed locus	---	0.4	0.000104773	0.002575952
1368696_at	FXYD domain-containing ion transport regulator 7	Fxyd7	0.3	0.000105353	0.002585426
1376282_at	Transcribed locus	---	0.6	0.000105473	0.002585426
1378039_at	Transcribed locus	---	0.5	0.000105631	0.002585444
1380688_at	UDP-N-acetyl-alpha-D-galactosamine:polypeptide N-acetylgalactosaminyltransferase-like 4 (predicted)	Galntl4_predicted	0.3	0.000105858	0.002587159
1374164_at	Transcribed locus	---	0.5	0.000106218	0.002592106
1393321_at	kelch domain containing 1 (predicted)	Klhdc1_predicted	0.5	0.000108954	0.002631524
1378252_at	chondrolectin (predicted)	Chodl_predicted	0.3	0.000109469	0.002633336
1387208_at	neuroglobin	Ngb	0.5	0.000110227	0.00263908
1391331_at	Transcribed locus	---	0.4	0.000110253	0.00263908
1379693_at	roundabout homolog 2 (Drosophila)	Robo2	0.4	0.000110521	0.00263908
1374310_at	protein phosphatase 1J	Ppm1j	0.4	0.000111972	0.002656412
1398233_at	Transcribed locus	---	0.5	0.000112098	0.002656412

1393291_at	similar to CG4502-PA, isoform A	LOC679949 /// LOC682677	0.4	0.000112416	0.002656412
1370648_a_at	SH3 domain binding protein CR16	Cr16	0.5	0.000112657	0.002656412
1368759_at	calcium channel, voltage-dependent, gamma subunit 2	Cacng2	0.2	0.000114226	0.002682757
1372443_at	low density lipoprotein receptor-related protein 11 (predicted)	Lrp11_predicted	0.6	0.0001145	0.002682757
1378345_at	Transcribed locus	---	0.5	0.000116161	0.002713934
1372782_a_at	similar to adenosine monophosphate deaminase 2 (isoform L)	LOC362015	0.7	0.000116521	0.002718281
1387309_a_at	glutamate receptor, ionotropic, kainate 1	Grik1	0.3	0.000116842	0.002718281
1372960_a_at	xylosyltransferase II	Xylt2	0.6	0.000117668	0.002733618
1382301_at	Transcribed locus	---	0.5	0.000117921	0.002735019
1390146_at	---	---	0.4	0.000118227	0.002735019
1383526_at	Transcribed locus	---	0.6	0.000118839	0.002738172
1390026_at	Bcl2-associated athanogene 3	Bag3	0.4	0.000118863	0.002738172
1371212_at	neuregulin 1	Nrg1	0.4	0.000120748	0.00277383
1378652_at	Transcribed locus	---	0.4	0.000121347	0.002783702
1369345_at	inositol polyphosphate-4-phosphatase, type II	Inpp4b	0.5	0.000122214	0.002799689
1370964_at	argininosuccinate synthetase	Ass	0.3	0.000123756	0.002824315
1374743_at	similar to channel-interacting PDZ domain protein isoform 1 (predicted)	RGD1565362_predicted	0.3	0.000123804	0.002824315
1368751_at	potassium voltage-gated channel, delayed-rectifier, subfamily S, member 3	Kcns3	0.3	0.000124381	0.002829627
1387556_at	contactin 6	Cntn6	0.4	0.000126139	0.002853825
1374954_at	histone deacetylase 11 (predicted)	Hdac11_predicted	0.5	0.000126316	0.00285392
1385280_at	Nucleotide binding protein 1	Nubp1	0.6	0.000126498	0.002854106
1376661_at	similar to CG11206-PA	LOC313672	0.4	0.000127095	0.002863646
1397840_at	Transcribed locus	---	0.4	0.000128502	0.002885085
1368103_at	ATP-binding cassette, sub-family G (WHITE), member 1	Abcg1	0.6	0.000128536	0.002885085
1380330_at	Transcribed locus	---	0.4	0.000128573	0.002885085
1378288_at	similar to RIKEN cDNA D630035O19 (predicted)	RGD1311100_predicted	0.4	0.000128968	0.002887736
1394491_at	---	---	0.4	0.0001291	0.002887736
1375484_at	Transcribed locus	---	0.6	0.000129839	0.002897684
1389573_at	ChaC, cation transport regulator-like 1 (E. coli) (predicted)	Chac1_predicted	0.5	0.000131098	0.002917866
1385640_at	proprotein convertase subtilisin/kexin type 9	Pcsk9	0.5	0.000131852	0.002930672
1368561_at	ATP-binding cassette, sub-family D (ALD), member 2	Abcd2	0.4	0.00013228	0.00293623
1395223_at	similar to hypothetical protein MGC35097	MGC94736	0.4	0.000133919	0.002956634
1379092_at	Transcribed locus	---	0.5	0.000135885	0.002980043
1381001_at	Kelch repeat and BTB (POZ) domain containing 3 (predicted)	Kbtbd3_predicted	0.5	0.000137448	0.003000657
1397177_at	Transcribed locus	---	0.4	0.000138337	0.003009751
1380570_at	---	---	0.5	0.00013973	0.003032023
1375066_at	similar to RIKEN cDNA 6330512M04 gene (predicted)	RGD1563319_predicted	0.3	0.000141051	0.00304339
1390865_at	similar to Ca2+-dependent activator for secretion protein 2 (predicted)	RGD1559440_predicted	0.4	0.000141636	0.00304339
1368879_a_at	guanine nucleotide binding protein, alpha o	Gnao	0.4	0.00014187	0.00304339
1392880_at	---	---	0.3	0.000143168	0.003062185
1386119_at	Hypothetical protein LOC689147	LOC689147	0.3	0.00014586	0.00310289
1384272_at	Zinc finger protein 365	Zfp365	0.5	0.000146163	0.00310289
1384923_at	Transcribed locus	---	0.4	0.000146203	0.00310289
1394412_at	transmembrane protein 16C (predicted)	Tmem16c_predicted	0.3	0.000146945	0.003110839
1369369_at	potassium voltage gated channel, Shab-related subfamily, member 2	Kcnb2	0.4	0.000147523	0.003110839
1373701_at	Transcribed locus	---	0.6	0.000149212	0.003138407
1367949_at	proenkephalin 1	Penk1	0.4	0.000149843	0.003142146
1383870_at	similar to homeotic protein Hox 2.2 - mouse (predicted)	RGD1562142_predicted	0.4	0.000149899	0.003142146
1385731_at	Transcribed locus	---	0.3	0.000151579	0.003159775
1379505_at	limb expression 1 homolog (chicken) (predicted) /// similar to limb expression 1	Lix1_predicted /// LOC500376	0.4	0.000152925	0.003179921
1385092_at	Eph receptor A6 (predicted)	Epha6_predicted	0.5	0.000154036	0.003194941

1381080_at	Transcribed locus	---	0.3	0.000155275	0.003216568
1377655_at	---	---	0.5	0.000155565	0.003217024
1374808_at	similar to hypothetical protein FLJ21156 (predicted)	RGD1309823_predicted	0.6	0.000155688	0.003217024
1377683_at	similar to hypothetical protein FLJ13045 (predicted)	RGD1307615_predicted	0.5	0.00015683	0.003232509
1371889_at	solute carrier family 22 (organic cation transporter), member 17	Slc22a17	0.5	0.000158135	0.003255315
1372017_at	diablo homolog (Drosophila)	Diablo	0.5	0.000159323	0.003272732
1373502_at	dymeclin (predicted)	Dym_predicted	0.5	0.000159559	0.003272732
1384229_at	guanine nucleotide binding protein, alpha stimulating, olfactory type	Gnal	0.5	0.000159777	0.003272732
1375947_at	Transcribed locus	---	0.6	0.000161207	0.003293839
1367725_at	serine/threonine-protein kinase pim-3	Pim3	0.5	0.000165774	0.003374564
1385321_at	similar to heterogeneous nuclear ribonucleoprotein methyltransferase-like 4 /// similar to Protein arginine N-methyltransferase 4 (Heterogeneous nuclear ribonucleoprotein methyltransferase-like protein 4)	LOC683275 /// LOC688502	0.3	0.000167706	0.003398625
1383367_at	sterile alpha motif domain containing 14	Samd14	0.5	0.000168485	0.003406638
1372179_at	hippocalcin-like 1	Hpcal1	0.6	0.000168795	0.003406638
1376104_at	similar to KIAA0802 protein (predicted)	RGD1308319_predicted	0.4	0.000169731	0.003421237
1394916_at	Transcribed locus	---	0.4	0.00017033	0.003429124
1377828_at	transmembrane protein 16C (predicted)	Tmem16c_predicted	0.3	0.000172934	0.003468805
1376657_at	immunoglobulin superfamily, member 4A	Igsf4a	0.6	0.000173308	0.003469941
1370124_at	metallothionein 3	Mt3	0.4	0.000174109	0.003475417
1369677_at	---	---	0.4	0.000175277	0.003490256
1393833_at	angel homolog 1 (Drosophila) (predicted)	Angel1_predicted	0.6	0.000175655	0.003493561
1393410_at	Transcribed locus	---	0.4	0.000178328	0.003538157
1376800_at	Transcribed locus	---	0.5	0.000178728	0.00354183
1389242_at	---	---	0.5	0.000180316	0.003553526
1389086_at	similar to RIKEN cDNA E430021N18 (predicted)	RGD1564043_predicted	0.3	0.000180615	0.003553526
1368949_at	early B-cell factor 1	Ebf1	0.4	0.000182072	0.003571466
1372602_at	similar to genethonin 1	RGD1311800	0.3	0.000182178	0.003571466
1387387_at	hippocalcin	Hpca	0.6	0.000184642	0.003599975
1376130_a_at	dystrobrevin, beta	Dtnb	0.5	0.000185466	0.003599975
1374262_at	Transcribed locus	---	0.5	0.000185553	0.003599975
1371045_at	amiloride-sensitive cation channel 2, neuronal	Accn2	0.4	0.000185554	0.003599975
1375026_at	similar to calmodulin-like 4	LOC686783 /// LOC691455	0.3	0.000186373	0.003604234
1370572_at	G protein-coupled receptor 149	Gpr149	0.4	0.000187136	0.003604234
1378421_at	Transcribed locus, strongly similar to XP_904500.1 hypothetical protein LOC69784 [Mus musculus]	---	0.3	0.000187731	0.003611462
1397753_at	phosphatidylinositol glycan, class Q	Pigq	0.6	0.00018822	0.003616636
1397899_at	---	---	0.6	0.000188869	0.003624868
1384655_at	kin of IRRE like 3 (Drosophila) (predicted)	Kirrel3_predicted	0.3	0.000190647	0.003637774
1392563_at	Rho GTPase activating protein 8 (predicted)	Arhgap8_predicted	0.5	0.000192333	0.003662697
1384132_at	immunoglobulin superfamily, member 4A	Igsf4a	0.3	0.000192745	0.003662697
1392920_at	Elongation factor RNA polymerase II-like 3	Ell3	0.4	0.000192844	0.003662697
1380828_at	gamma-aminobutyric acid A receptor, alpha 1	Gabra1	0.3	0.000193592	0.003672675
1384942_at	leucine rich repeat and fibronectin type III domain containing 5 (predicted)	Lrfn5_predicted	0.5	0.000194273	0.003681347
1391036_at	IQ motif and Sec7 domain 3	Iqsec3	0.6	0.000194955	0.003684317
1370546_at	unc-13 homolog C (C. elegans)	Unc13c	0.3	0.000195031	0.003684317
1369035_a_at	potassium inwardly-rectifying channel, subfamily J, member 6	Kcnj6	0.5	0.000195445	0.003684317
1396237_at	Transcribed locus	---	0.6	0.000195568	0.003684317
1382632_at	roundabout homolog 2 (Drosophila)	Robo2	0.4	0.000195696	0.003684317
1381904_at	G7c protein	G7c	0.6	0.000195774	0.003684317
1394070_at	---	---	0.5	0.000198465	0.003726432
1398330_at	syntaxin binding protein 1	Stxbp1	0.6	0.000201386	0.003772665
1368120_at	NEL-like 1 (chicken)	Nell1	0.5	0.000202136	0.003778109
1379662_a_at	Transcribed locus	---	0.5	0.000202905	0.003788176
1382924_at	pantothenate kinase 1 (predicted)	Pank1_predicted	0.4	0.00020535	0.003811252

1391092_at	Transcribed locus	---	0.5	0.00020562	0.003811252
1384025_at	Transcribed locus	---	0.5	0.000205939	0.003811252
1379374_at	plasticity related gene 1	Prg1	0.5	0.000214961	0.0039329
1368523_at	Ca2+-dependent secretion activator	Cadps	0.6	0.000220655	0.004023646
1372051_at	rhomboid, veinlet-like 7 (Drosophila) (predicted)	Rhbdl7_predicted	0.5	0.000221014	0.004025739
1383309_at	ST3 beta-galactoside alpha-2,3-sialyltransferase 6	St3gal6	0.5	0.000221499	0.004025815
1384368_at	DNA2 DNA replication helicase 2-like (yeast) (predicted)	Dna2l_predicted	0.4	0.000221699	0.004025815
1376021_at	Transcribed locus, strongly similar to XP_001081813.1 similar to leucine rich repeat containing 45 [Rattus norvegicus]	---	0.6	0.0002235	0.004048591
1382225_at	Transcribed locus	---	0.5	0.000225276	0.00406733
1398412_at	Transcribed locus	---	0.4	0.000228049	0.00409939
1372825_at	Formin binding protein 1	Fnbp1	0.7	0.00022872	0.004106971
1379414_at	similar to CG4502-PA, isoform A	LOC679949 /// LOC682677	0.4	0.000230028	0.004125181
1383493_at	Transcribed locus	---	0.4	0.000230236	0.004125181
1369116_a_at	calcitonin/calcitonin-related polypeptide, alpha	Calca	0.3	0.000230831	0.004129173
1378738_at	potassium voltage-gated channel, shaker-related subfamily, beta member 1	Kcnab1	0.4	0.000230961	0.004129173
1378424_at	tripartite motif protein 46 (predicted)	Trim46_predicted	0.6	0.000231527	0.0041348
1389550_at	SH3-domain GRB2-like 2	Sh3gl2	0.5	0.000234416	0.004170779
1398615_at	Transcribed locus	---	0.4	0.000234417	0.004170779
1372755_at	mal, T-cell differentiation protein 2	Mal2	0.4	0.000234523	0.004170779
1377773_at	Transcribed locus, weakly similar to NP_766020.1 cDNA C030002O17 [Mus musculus]	---	0.5	0.000234556	0.004170779
1378909_at	Transcribed locus	---	0.4	0.000235547	0.004180784
1391765_at	leucine rich repeat containing 48	Lrrc48	0.6	0.000236546	0.004180784
1397232_at	leucine rich repeat neuronal 1	Lrrn1	0.5	0.000236625	0.004180784
1381233_at	Transcribed locus	---	0.3	0.000236809	0.004180784
1373465_at	PQ loop repeat containing 1	Pqlc1	0.3	0.000236898	0.004180784
1368986_at	solute carrier family 17 (sodium-dependent inorganic phosphate cotransporter), member 7	Slc17a7	0.6	0.000239274	0.004218195
1379452_at	similar to growth arrest-specific protein 2 - mouse (predicted)	RGD1563167_predicted	0.6	0.00024137	0.004246043
1378310_at	similar to neuromedin B precursor - rat (predicted)	RGD1562710_predicted	0.3	0.000242038	0.004253247
1389540_at	similar to IQ motif and Sec7 domain 1	LOC686590	0.5	0.000243906	0.004272382
1368572_a_at	glutamate receptor, ionotropic, N-methyl D-aspartate 1	Grin1	0.4	0.000247596	0.004304926
1390244_at	spermatogenesis associated, serine-rich 2 (predicted)	Spats2_predicted	0.6	0.000248714	0.004314461
1368958_at	protein kinase C and casein kinase substrate in neurons 1	Pacsin1	0.2	0.000248931	0.004314461
1389278_at	TSPY-like 2 (predicted)	Tspyl2_predicted	0.4	0.000253089	0.004372698
1382835_at	Transcribed locus	---	0.5	0.000255728	0.004410634
1373734_at	---	---	0.5	0.000257637	0.004423394
1391089_at	---	---	0.5	0.000260285	0.004454917
1368276_at	synaptophysin	Syp	0.4	0.000260616	0.004455937
1377632_at	similar to Metalloproteinase inhibitor 4 precursor (TIMP-4) (Tissue inhibitor of metalloproteinases-4)	LOC678834 /// LOC680130	0.4	0.000261395	0.00446308
1393295_at	similar to RIKEN cDNA 2610019F03	LOC498662	0.6	0.000262388	0.004468034
1374619_at	tumor suppressor candidate 5	Tusc5	0.5	0.000262682	0.004468034
1380011_at	Transcribed locus	---	0.4	0.000266929	0.004516924
1370110_at	potassium channel, subfamily K, member 4	Kcnk4	0.2	0.000267238	0.004517498
1379482_at	transmembrane 6 superfamily member 1 (predicted)	Tm6sf1_predicted	0.5	0.000269437	0.004541834
1373265_at	Transcribed locus	---	0.7	0.000271675	0.004559701
1392644_s_at	similar to RIKEN cDNA 2610019F03	LOC498662	0.4	0.000273325	0.004579388
1396542_at	Transcribed locus	---	0.4	0.000273405	0.004579388
1373689_at	Transcribed locus	---	0.6	0.000275004	0.004596822
1390792_a_at	Transcribed locus	---	0.6	0.000275816	0.004600963
1398433_at	Transcribed locus	---	0.5	0.000276269	0.004600963
1391241_at	Transcribed locus	---	0.5	0.000277686	0.004608172
1378111_at	Transcribed locus	---	0.3	0.000277925	0.004608172

1385043_at	similar to channel-interacting PDZ domain protein isoform 1 (predicted)	RGD1565362_predicted	0.3	0.000279389	0.00462778
1368690_a_at	glutamate receptor, metabotropic 4	Grm4	0.6	0.000281072	0.004643352
1383459_at	leucine-rich repeat LGI family, member 3 (predicted)	Lgi3_predicted	0.3	0.000281247	0.004643352
1380228_at	similar to hypothetical protein MGC47816 (predicted)	RGD1306880_predicted	0.3	0.000284251	0.004683546
1393359_at	adaptor-related protein complex 3, beta 2 subunit (predicted)	Ap3b2_predicted	0.5	0.000284464	0.004683546
1397439_at	similar to diacylglycerol kinase epsilon	LOC497978	0.6	0.000286369	0.004695834
1390555_at	similar to Suppressor of cytokine signaling 5 (predicted)	RGD1564914_predicted	0.6	0.00028643	0.004695834
1383084_at	Transcribed locus	---	0.5	0.000286638	0.004695834
1388462_at	SH3-binding kinase 1	Sbk1	0.4	0.000292333	0.004770133
1393783_at	---	---	0.5	0.000296139	0.004808393
1374644_at	Transcribed locus	---	0.4	0.000298477	0.004836983
1382734_at	jagged 2	Jag2	0.3	0.000300169	0.004854648
1384243_at	Transcribed locus	---	0.6	0.000301789	0.0048665
1373774_at	Ras-like without CAAX 2	Rit2	0.4	0.000302168	0.004867834
1371081_at	Rap guanine nucleotide exchange factor (GEF) 4	Rapgef4	0.3	0.000303558	0.004878719
1387185_at	amyloid beta (A4) precursor protein-binding, family B, member 3	Apbb3	0.6	0.000305864	0.004908146
1392916_at	microtubule-associated protein 7 (predicted)	Mtap7_predicted	0.5	0.000312085	0.004985605
1377712_at	Catenin (cadherin-associated protein), delta 2	Ctnnd2	0.4	0.000312207	0.004985605
1370439_a_at	potassium voltage gated channel, Shaw-related subfamily, member 2	Kcnc2	0.5	0.000316653	0.005029307
1396238_at	UDP-N-acetyl-alpha-D-galactosamine:polypeptide N-acetylgalactosaminyltransferase 14	Galnt14	0.4	0.000317092	0.005029434
1380046_at	Similar to nucleosome assembly protein 1-like 5	MGC105733	0.4	0.000318483	0.00504663
1380345_at	Transcribed locus	---	0.6	0.000324109	0.005091641
1387322_at	sema domain, transmembrane domain (TM), and cytoplasmic domain, (semaphorin) 6B	Sema6b	0.6	0.000324933	0.005096574
1394553_at	Transcribed locus	---	0.5	0.000325043	0.005096574
1392555_at	Ets variant gene 1 (predicted)	Etv1_predicted	0.5	0.000329304	0.005143767
1396313_at	Transcribed locus	---	0.7	0.000330374	0.005150704
1370328_at	dickkopf homolog 3 (Xenopus laevis)	Dkk3	0.6	0.000330971	0.00515512
1371361_at	tensin /// similar to tensin	LOC301509 /// Tns	0.5	0.000332261	0.005163352
1385925_at	Transcribed locus	---	0.5	0.000332441	0.005163352
1373995_at	---	---	0.4	0.000333496	0.005173216
1378107_at	Transcribed locus	---	0.4	0.000334078	0.005174118
1384202_at	similar to Tescalcin (predicted)	RGD1566317_predicted	0.5	0.000335071	0.005184613
1383061_at	Transcribed locus	---	0.6	0.000335728	0.005186804
1390892_at	DEP domain containing 1B (predicted)	Depdc1b_predicted	0.5	0.000335843	0.005186804
1392003_at	similar to KIAA0316 protein (predicted)	RGD1566031_predicted	0.4	0.000337363	0.005196847
1374089_at	Transcribed locus	---	0.5	0.00033817	0.005196847
1373807_at	Transcribed locus	---	0.5	0.000338389	0.005196847
1382914_at	Transcribed locus	---	0.3	0.000340924	0.005230897
1370267_at	glycogen synthase kinase 3 beta	Gsk3b	0.5	0.000341489	0.005234675
1382138_at	similar to Notch-regulated ankyrin repeat protein	LOC499745 /// LOC688240	0.5	0.000343603	0.00526218
1382944_at	---	---	0.5	0.000347425	0.005292591
1372300_at	docking protein 4 (predicted)	Dok4_predicted	0.4	0.000347519	0.005292591
1391547_at	CDNA clone IMAGE:7320555	---	0.5	0.000348149	0.005297269
1367660_at	fatty acid binding protein 3	Fabp3	0.4	0.000348841	0.005302858
1393350_at	---	---	0.5	0.000350024	0.005306176
1380272_at	Transcribed locus	---	0.5	0.000350902	0.005314582
1383340_at	ataxia, cerebellar, Cayman type (caytaxin) (predicted)	Atcay_predicted	0.6	0.000351977	0.005324119
1376726_at	---	---	0.5	0.000352179	0.005324119
1394833_at	Transcribed locus	---	0.3	0.000359028	0.005405027
1384824_at	protocadherin 18 (predicted)	Pcdh18_predicted	0.6	0.000359174	0.005405027

1371578_at	protein kinase, cAMP-dependent, catalytic, alpha	Prkaca	0.4	0.000361608	0.005428713
1392619_at	Transcribed locus	---	0.6	0.000361738	0.005428713
1384877_at	aquaporin 11	Aqp11	0.4	0.000363655	0.005447533
1394455_at	Transcribed locus	---	0.5	0.000365087	0.005460426
1383824_at	LIM domain binding 2 (predicted)	Ldb2_predicted	0.6	0.00036637	0.005473249
1374213_at	ADP-ribosylation factor guanine nucleotide-exchange factor 2 (brefeldin A-inhibited)	Arfgef2	0.5	0.000366726	0.005473605
1391472_at	DAZ interacting protein 1	Dzip1	0.6	0.000367909	0.005483694
1375639_at	Transcribed locus	---	0.5	0.000368676	0.005483694
1379052_at	Transcribed locus	---	0.6	0.00036868	0.005483694
1394297_at	homeo box D1 (predicted)	Hoxd1_predicted	0.2	0.000368736	0.005483694
1372695_at	Fibronectin type III domain containing 5	Fndc5	0.4	0.000372351	0.005532457
1380766_a_at	similar to RIKEN cDNA 8430427H17 gene (predicted)	RGD1563510_predicted	0.6	0.000375661	0.005568157
1389752_at	sortilin-related VPS10 domain containing receptor 3 (predicted)	Sorcs3_predicted	0.5	0.000376624	0.005570788
1394816_at	Transcribed locus	---	0.6	0.000381011	0.005615439
1397666_at	Transcribed locus	---	0.4	0.000381197	0.005615439
1396023_at	Transcribed locus	---	0.3	0.000385981	0.005668405
1383122_at	protein kinase, AMP-activated, gamma 2 non-catalytic subunit	Prkag2	0.5	0.000389756	0.005713642
1369001_at	cholinergic receptor, nicotinic, alpha polypeptide 3	Chrna3	0.2	0.000393964	0.005754813
1377882_at	FMS-like tyrosine kinase 3	Flt3	0.5	0.000395757	0.005769901
1390164_at	Transcribed locus	---	0.6	0.000400357	0.005817204
1373545_at	Transcribed locus	---	0.4	0.000401768	0.005832432
1372423_at	PERP, TP53 apoptosis effector (predicted)	Perp_predicted	0.4	0.000402505	0.005832432
1398525_at	Transcribed locus	---	0.5	0.000402824	0.005832432
1378396_at	Transcribed locus	---	0.6	0.000403966	0.005833935
1377185_at	similar to RIKEN cDNA 6330442E10 gene (predicted)	RGD1562622_predicted	0.5	0.00040526	0.005841989
1371108_a_at	ATPase, Na+/K+ transporting, alpha 1 polypeptide	Atp1a1	0.2	0.000407056	0.005862746
1392045_at	transmembrane protein 22	Tmem22	0.5	0.000409816	0.0058819
1392444_at	similar to N-acetylglucosamine 6-O-sulfotransferase (predicted)	RGD1561144_predicted	0.5	0.00041067	0.00588902
1392944_at	similar to talin 2 (predicted)	RGD1565416_predicted	0.7	0.000415367	0.005937926
1378214_at	N-acetylgalactosamine 4-sulfate 6-O-sulfotransferase	GalNAc4S6ST	0.4	0.000415525	0.005937926
1373336_at	G protein-coupled receptor, family C, group 5, member B (predicted)	Gprc5b_predicted	0.7	0.000416066	0.005940497
1378246_at	similar to T-cell activation protein phosphatase 2C (predicted)	RGD1310383_predicted	0.5	0.000419431	0.005976639
1368153_a_at	nasal embryonic LHRH factor	Nelf	0.6	0.000419979	0.005976639
1383274_at	poly(rC) binding protein 3	Pcbp3	0.5	0.000420414	0.005976639
1388675_at	similar to spinster-like protein	RGD1305613	0.5	0.000421988	0.005988662
1368255_at	neurotrimin	Hnt	0.6	0.000423268	0.006001655
1376980_at	Transcribed locus	---	0.4	0.000426863	0.006031822
1367918_at	fasciculation and elongation protein zeta 1 (zygin I)	Fez1	0.5	0.000431433	0.006076409
1382851_at	Transcribed locus	---	0.6	0.000431496	0.006076409
1376925_at	palmdelphin	Palmd	0.6	0.000433649	0.006101501
1380486_at	similar to SEC22 vesicle trafficking protein-like 3	LOC687022	0.7	0.000435889	0.006127773
1371331_at	follistatin-like 1	Fstl1	0.5	0.000438804	0.0061549
1379632_at	Similar to mitochondrial carrier family protein	RGD1308774	0.6	0.000439383	0.0061549
1374013_at	C1q and tumor necrosis factor related protein 5	C1qtnf5	0.5	0.000439592	0.0061549
1368044_at	secretogranin 2	Scg2	0.3	0.000441981	0.006176473
1372994_at	similar to Fanconi anemia, complementation group E (predicted)	RGD1561045_predicted	0.7	0.000447361	0.006235176
1368768_at	sodium channel, voltage-gated, type XI, alpha	Scn11a	0.2	0.000447698	0.006235176
1383216_at	similar to mKIAA0431 protein	RGD1305781	0.5	0.000452481	0.006291133
1376443_at	Regulator of G-protein signalling 3	Rgs3	0.4	0.000457202	0.006341421
1368152_at	zinc finger, DHHC domain containing 7	Zdhhc7	0.6	0.000457255	0.006341421
1370680_at	staufen, RNA binding protein, homolog 2 (Drosophila)	Stau2	0.5	0.000458718	0.006356358

1390999_at	Transcribed locus	---	0.5	0.000459251	0.006358388
1385016_at	similar to RIKEN cDNA B630019K06 (predicted)	RGD1560784_predicted	0.4	0.000461504	0.006373485
1372482_at	solute carrier family 39 (zinc transporter), member 13	Slc39a13	0.6	0.00046519	0.00641362
1385749_at	Transcribed locus	---	0.5	0.000466206	0.006422247
1391976_at	Transcribed locus	---	0.4	0.000473463	0.006483334
1372629_at	similar to KIAA0925 protein	LOC300768	0.6	0.000473794	0.006483334
1370675_at	transient receptor potential cation channel, subfamily V, member 1	Trpv1	0.3	0.000478459	0.006530871
1395331_at	similar to hypothetical protein CL25084 (predicted)	RGD1306508_predicted	0.4	0.000482969	0.006565181
1383319_at	solute carrier family 4, sodium bicarbonate transporter-like, member 11 (predicted)	Slc4a11_predicted	0.6	0.000485158	0.006584052
1374445_at	G protein-coupled receptor 162 (predicted)	Gpr162_predicted	0.5	0.000485874	0.006588338
1376656_at	ubiquitin specific protease 20 (predicted)	Usp20_predicted	0.6	0.00048628	0.006588412
1397983_at	Transcribed locus	---	0.4	0.000487831	0.006598553
1390799_at	Transcribed locus	---	0.6	0.000490592	0.006615438
1372539_at	Transcribed locus	---	0.4	0.00049109	0.006615438
1389109_at	Transcribed locus	---	0.7	0.000497025	0.006684431
1383219_at	---	---	0.5	0.000498232	0.006695191
1380866_at	similar to adenylate kinase 5 isoform 1	LOC365985	0.4	0.000503684	0.006757419
1392221_at	Similar to GREB1 protein isoform a (predicted)	RGD1562371_predicted	0.5	0.000505558	0.006771508
1394564_at	Transcribed locus	---	0.6	0.000506065	0.006772792
1394392_at	similar to dJ881L22.2 (novel protein) (predicted)	RGD1307696_predicted	0.4	0.000513451	0.006854903
1374062_x_at	microtubule-associated protein, RP/EB family, member 3	Mapre3	0.3	0.000514862	0.00686495
1384780_at	copine IV (predicted)	Cpne4_predicted	0.4	0.000515038	0.00686495
1378763_at	hypothetical gene supported by BC088439	LOC500947	0.5	0.000515572	0.006866497
1372490_at	Transcribed locus	---	0.6	0.000521882	0.006933697
1367956_at	neurochondrin	Ncdn	0.3	0.000523412	0.006942942
1368904_at	calpain 10	Capn10	0.6	0.000525111	0.006942942
1383777_at	Transcribed locus	---	0.6	0.000526214	0.00695194
1384287_at	Transcribed locus	---	0.7	0.000528068	0.006970842
1394577_at	Transcribed locus	---	0.4	0.000529471	0.006983754
1389313_at	Transcribed locus, weakly similar to XP_001170787.1 phosphofurin acidic cluster sorting protein 1 isoform 4 [Pan troglodytes]	---	0.6	0.000530408	0.006990506
1376183_at	Transcribed locus	---	0.3	0.00053495	0.007007317
1391560_at	human immunodeficiency virus type I enhancer binding protein 1	Hivep1	0.3	0.000539248	0.007042452
1369210_at	sodium channel, voltage-gated, type I, alpha	Scn1a	0.4	0.000539487	0.007042452
1393126_at	ubiquitin domain containing 1	Ubtd1	0.6	0.000540982	0.007056363
1368185_at	guanine nucleotide binding protein, alpha z subunit	Gnaz	0.5	0.000543703	0.007086239
1373212_at	mitochondrial ATP synthase regulatory component factor B	Atp5s	0.5	0.000546598	0.007112697
1392739_a_at	similar to RIKEN cDNA 2310005P05	RGD1307401	0.5	0.000548198	0.007122245
1368890_at	glyceronephosphate O-acyltransferase	Gnpat	0.5	0.00055003	0.00712915
1377059_at	Transcribed locus	---	0.7	0.000551853	0.007144225
1369500_at	potassium channel, subfamily K, member 1	Kcnk1	0.5	0.000554062	0.007153232
1374929_at	similar to RIKEN cDNA 6330407D12 (predicted)	RGD1561171_predicted	0.5	0.000565348	0.007271242
1385526_at	similar to autophagy 5-like	LOC365601	0.6	0.000568729	0.00729108
1393502_at	similar to predicted CDS, putative protein of bilaterial origin (4J193)	RGD1306153	0.6	0.000575265	0.007357664
1383887_at	similar to Protein C20orf103 precursor	RGD1306991	0.3	0.000576888	0.007362322
1388998_at	erythrocyte protein band 4.9 (predicted)	Epb4.9_predicted	0.5	0.000577963	0.007362322
1368197_at	opioid receptor, kappa 1	Oprk1	0.4	0.000578485	0.007362322
1374952_at	similar to expressed sequence AI428855	LOC684849 /// LOC689984	0.5	0.00057876	0.007362322
1378102_at	similar to cDNA sequence AF096286; pecanex 1 (predicted)	RGD1305883_predicted	0.5	0.000578778	0.007362322
1387164_at	leukocyte cell derived chemotaxin 1	Lect1	0.6	0.00057921	0.007362322
1391916_at	Transcribed locus	---	0.6	0.000579816	0.00736433
1385643_at	Transcribed locus	---	0.6	0.000582342	0.0073796

1389015_at	similar to FUN14 domain containing 2 (predicted)	RGD1560916_predicted	0.5	0.000582364	0.0073796
1386615_at	carbohydrate sulfotransferase 2 (predicted)	Chst2_predicted	0.4	0.000582986	0.007381795
1372708_at	---	---	0.5	0.000584789	0.007394963
1379997_at	Transcribed locus	---	0.5	0.000585824	0.007394963
1387065_at	phospholipase C, delta 4	Plcd4	0.2	0.000588125	0.007418314
1387004_at	neuroblastoma, suppression of tumorigenicity 1	Nbl1	0.4	0.000592591	0.007468913
1391262_at	similar to SUMO/sentrin specific protease 5 (predicted) /// similar to SUMO/sentrin specific protease 5	LOC686268 /// LOC690251 /// RGD1564247_predicted	0.3	0.00059436	0.007485478
1372275_at	Transcribed locus	---	0.5	0.000597176	0.007510856
1370078_at	lin-7 homolog b (C. elegans)	Lin7b	0.3	0.000602299	0.00755077
1379373_at	Transcribed locus	---	0.4	0.000604308	0.007570184
1383612_at	Transcribed locus	---	0.6	0.000612708	0.007652108
1373931_at	similar to RIKEN cDNA C530028O21 gene	RGD1304952	0.6	0.000615379	0.007673813
1380914_at	A kinase (PRKA) anchor protein 5	Akap5	0.7	0.000615954	0.007675161
1379419_at	transmembrane protein 34	Tmem34	0.6	0.000620738	0.007723067
1376736_at	hypothetical protein LOC683263	LOC683263	0.4	0.000622136	0.007734614
1382593_at	ets variant gene 1 (predicted)	Etv1_predicted	0.6	0.000623264	0.007736935
1373729_at	Transcribed locus	---	0.5	0.000631593	0.007808467
1372398_at	similar to potassium channel tetramerisation domain containing 15	LOC499129	0.5	0.000631973	0.007808467
1368121_at	aldo-keto reductase family 7, member A3 (aflatoxin aldehyde reductase)	Akr7a3	0.5	0.000632349	0.007808467
1390240_at	Transcribed locus	---	0.6	0.000634752	0.007826388
1367994_at	dihydropyrimidine dehydrogenase	Dpyd	0.6	0.000635749	0.00783217
1388246_at	clusterin	Clu	0.5	0.000639996	0.007859516
1384866_at	ectonucleoside triphosphate diphosphohydrolase 3	Entpd3	0.3	0.000643622	0.007888442
1380317_at	cadherin 10	Cdh10	0.5	0.000647075	0.00791308
1372682_at	similar to RIKEN cDNA 2810432L12	RGD1307218	0.5	0.000648027	0.007918837
1370415_at	Ras association (RalGDS/AF-6) domain family 5	Rassf5	0.6	0.000649742	0.007933897
1373832_at	sorting nexin 19 (predicted)	Snx19_predicted	0.7	0.000653351	0.007972045
1392815_at	Centaurin, delta 1 (predicted)	Centd1_predicted	0.4	0.000654037	0.007974504
1377288_at	heat shock transcription factor 4 (predicted)	Hsf4_predicted	0.6	0.000659517	0.00801754
1396117_at	similar to leucine zipper protein 2 (predicted)	RGD1563838_predicted	0.5	0.000663305	0.008045758
1398446_at	similar to chromosome 1 open reading frame 2 (predicted)	RGD1306107_predicted	0.5	0.00066482	0.008058192
1376904_at	similar to hypothetical protein MGC33486 (predicted)	RGD1310680_predicted	0.5	0.000668963	0.00809021
1383346_at	similar to ERCC4_MOUSE (predicted)	RGD1560340_predicted	0.7	0.000669129	0.00809021
1376893_at	vesicular membrane protein p24 (predicted)	Vmp_predicted	0.4	0.000669429	0.00809021
1380380_at	Tubulin tyrosine ligase	Ttl	0.6	0.000672588	0.008114339
1372669_at	Transcribed locus	---	0.4	0.000672906	0.008114339
1383447_at	ets variant gene 5 (ets-related molecule) (predicted)	Etv5_predicted	0.6	0.000674442	0.008126605
1374486_at	Rho guanine nucleotide exchange factor (GEF) 11	Arhgef11	0.6	0.000680437	0.00817706
1389963_at	p55 protein	LOC652956	0.4	0.000680593	0.00817706
1377523_at	similar to dachshund b	LOC686314	0.7	0.000681883	0.008186574
1390068_at	nuclear factor, erythroid derived 2,-like 1 (predicted)	Nfe2l1_predicted	0.7	0.000682917	0.008193014
1383443_at	similar to UPF0308 protein C9orf21	LOC498685	0.5	0.00068714	0.008237671
1392315_at	REV1-like (S. cerevisiae) (predicted)	Rev1l_predicted	0.6	0.00069056	0.008255985
1383861_at	Transcribed locus	---	0.5	0.000690572	0.008255985
1399040_at	glucosidase beta 2	Gba2	0.7	0.000690676	0.008255985
1377044_at	Transcribed locus	---	0.5	0.000691339	0.008256224
1377171_at	leucine zipper, putative tumor suppressor 1	Lzts1	0.6	0.000694589	0.0082779
1393197_at	abhydrolase domain containing 8 (predicted)	Abhd8_predicted	0.4	0.000694662	0.0082779
1374244_at	---	---	0.6	0.000695339	0.0082779
1382054_at	Transcribed locus	---	0.6	0.000695529	0.0082779
1392195_at	Transcribed locus	---	0.4	0.000698376	0.008299777

1397923_at	Similar to erythrocyte membrane protein band 4.1 (elliptocytosis 1, RH-linked) isoform 1 (predicted)	RGD1564762_predicted	0.6	0.000704717	0.008350998
1373581_at	Similar to KIAA0672 gene product	RGD1305664	0.6	0.000707416	0.008372402
1374466_at	Transcribed locus	---	0.5	0.00070969	0.008386223
1393223_a_at	similar to RIKEN cDNA 1110008J03 (predicted)	RGD1306772_predicted	0.7	0.000714637	0.008420019
1373636_at	sparc/osteonectin, cwcv and kazal-like domains proteoglycan 1	Spock1	0.5	0.000721357	0.008475214
1376165_at	solute carrier family 24 (sodium/potassium/calcium exchanger), member 3	Slc24a3	0.6	0.000722748	0.008475214
1380266_at	Transcribed locus	---	0.5	0.000723349	0.008475214
1377266_at	Transcribed locus	---	0.5	0.000725994	0.008499043
1378000_at	Transcribed locus	---	0.6	0.000729433	0.008528495
1373546_at	Putative UA20 protein	Ua20	0.5	0.000729547	0.008528495
1373119_at	potassium channel tetramerisation domain containing 3	Kctd3	0.6	0.000731099	0.008540565
1376222_at	similar to HCDI protein (predicted)	RGD1309307_predicted	0.7	0.000737804	0.00859581
1379437_at	Transcribed locus	---	0.6	0.000738149	0.00859581
1379617_at	Similar to CG33331-PA	LOC362419	0.7	0.000738964	0.00859581
1390839_at	PQ loop repeat containing 3	Pqlc3	0.6	0.000742524	0.008631117
1383468_at	Transcribed locus	---	0.6	0.000745223	0.008656372
1384468_at	Transcribed locus	---	0.4	0.000756399	0.008760136
1388881_at	similar to RIKEN cDNA 1700012G19 gene (predicted)	RGD1307773_predicted	0.7	0.000757109	0.008760136
1375032_at	similar to kinase non-catalytic C-lobe domain (KIND) containing 1 isoform b (predicted)	RGD1565261_predicted	0.6	0.000762984	0.008812896
1393370_at	similar to doublecortin-like kinase 2 (predicted)	Dclk2_predicted	0.7	0.000768408	0.00885688
1384158_at	Transcribed locus	---	0.3	0.000769332	0.00886132
1371754_at	solute carrier family 25 (mitochondrial carrier, phosphate carrier), member 25	Slc25a25	0.6	0.000774402	0.008901019
1392993_at	---	---	0.6	0.000777861	0.008922897
1396428_at	Transcribed locus	---	0.5	0.000779328	0.008925406
1389533_at	fibulin 2	Fbln2	0.5	0.000779779	0.008925406
1396936_at	Zinc finger protein 532 (predicted)	Znf532_predicted	0.7	0.000783637	0.00896332
1368013_at	DNA-damage-inducible transcript 4-like	Ddit4l	0.4	0.000790575	0.009017059
1373154_at	similar to mKIAA0998 protein (predicted)	RGD1563583_predicted	0.6	0.000791076	0.009017059
1368740_at	purinergic receptor P2X-like 1, orphan receptor	P2rxl1	0.2	0.000793335	0.009025155
1373594_at	Transcribed locus	---	0.5	0.000793826	0.009025155
1371077_at	5-hydroxytryptamine (serotonin) receptor 3b	Htr3b	0.2	0.000793981	0.009025155
1394295_at	Transcribed locus	---	0.3	0.000800079	0.00906034
1382274_at	retinoic acid receptor responder (tazarotene induced) 1	Rarres1	0.4	0.000800381	0.00906034
1397953_at	similar to AMSH-family protein	LOC687696	0.5	0.000803178	0.009085743
1390500_at	similar to RIKEN cDNA 9130023F12 gene (predicted)	RGD1311920_predicted	0.6	0.000805996	0.009095741
1370562_at	calcitonin-related polypeptide, beta	Calcb	0.4	0.000806274	0.009095741
1387951_at	decay accelarating factor 1	Daf1	0.4	0.000809915	0.009124301
1385128_at	Transcribed locus	---	0.6	0.000818989	0.009176249
1391157_at	Similar to CG7744-PA	LOC691426	0.6	0.000820775	0.009184672
1367489_at	---	---	0.7	0.00082224	0.009187546
1390033_at	---	---	0.4	0.000823907	0.009187546
1379769_at	Transcribed locus	---	0.4	0.000825056	0.00919412
1370385_at	phospholipase A2, group VI	Pla2g6	0.7	0.000828075	0.009220037
1368939_a_at	neurotrophic tyrosine kinase, receptor, type 3	Ntrk3	0.5	0.000828828	0.009220037
1379801_at	Transcribed locus, strongly similar to XP_580017.1 hypothetical protein XP_580017 [Rattus norvegicus]	---	0.6	0.000829063	0.009220037
1379263_at	fukutin related protein	Fkrp	0.5	0.000830403	0.009228698
1388470_at	F-box only protein 9	Fbxo9	0.6	0.000832444	0.009238889
1368485_at	advillin	Avil	0.4	0.00084074	0.009306809
1396925_at	Transcribed locus	---	0.6	0.000840827	0.009306809
1368886_at	mitogen activated protein kinase kinase kinase 12	Map3k12	0.7	0.000845991	0.009330312

1368524_at	potassium voltage gated channel, Shaw-related subfamily, member 1	Kcnc1	0.4	0.000846354	0.009330312
1398393_at	similar to mitogen-activated protein kinase 11	LOC679007 /// LOC689314	0.5	0.000848922	0.009352359
1380162_at	RDCR-0918-3 protein	LOC246120	0.6	0.000855454	0.009411711
1371635_at	G protein-coupled receptor 175	Gpr175	0.7	0.000858092	0.009434419
1394252_at	sparc/osteonectin, cwcv and kazal-like domains proteoglycan 3 (predicted)	Spock3_predicted	0.5	0.000858868	0.009436646
1392137_at	Transcribed locus	---	0.6	0.000862218	0.009463654
1371570_at	scratch homolog 1, zinc finger protein (Drosophila) (predicted)	Scrt1_predicted	0.3	0.000863186	0.009463654
1377473_at	SWI/SNF related, matrix associated, actin dependent regulator of chromatin, subfamily d, member 3	Smarcd3	0.7	0.000867747	0.009489829
1387130_at	solute carrier family 39 (iron-regulated transporter), member 1	Slc40a1	0.6	0.000871336	0.009508976
1389429_at	---	---	0.7	0.000875713	0.00953889
1393470_at	---	---	0.5	0.000879666	0.009575612
1385343_at	---	---	0.5	0.000894945	0.009708254
1390672_at	candidate mediator of the p53-dependent G2 arrest	Rprm	0.4	0.000896132	0.009709867
1375941_at	BAI1-associated protein 2-like 1	Baiap2l1	0.5	0.000901889	0.009755149
1392877_at	---	---	0.6	0.000905909	0.009777159
1372802_at	similar to mKIAA1805 protein (predicted)	RGD1561526_predicted	0.7	0.000907136	0.009777575
1374835_at	RAB-interacting factor	Rabif	0.6	0.000921608	0.009907591
1377738_a_at	cysteine and histidine rich 1	Cyhr1	0.6	0.000923254	0.009913539
1367585_a_at	ATPase, Na+/K+ transporting, alpha 1 polypeptide	Atp1a1	0.3	0.000923367	0.009913539
1379953_at	similar to T-cell activation protein phosphatase 2C (predicted)	RGD1310383_predicted	0.4	0.000933159	0.009973095
1394110_at	Transcribed locus	---	0.6	0.000943999	0.010056281
1368401_at	glutamate receptor, ionotropic, AMPA2	Gria2	0.4	0.000948453	0.010090655
1392529_at	Transcribed locus	---	0.7	0.000950648	0.010107474
1398352_at	protein inhibitor of activated STAT, 4	Pias4	0.3	0.000957502	0.010160639
1390292_at	transmembrane protein 8 (five membrane-spanning domains) (predicted)	Tmem8_predicted	0.6	0.00096357	0.010205275
1382029_at	similar to CDNA sequence BC017647 (predicted)	RGD1566149_predicted	0.7	0.000965587	0.010210808
1389774_at	Transcribed locus	---	0.5	0.000968861	0.010229273
1385744_at	similar to KIAA1679 protein (predicted)	RGD1306938_predicted	0.4	0.000972227	0.010252455
1388151_at	coronin 7	Coro7	0.7	0.00097256	0.010252455
1383699_at	Ras-like without CAAX 2	Rit2	0.4	0.000975506	0.010272163
1371393_at	calsyntenin 1	Clstn1	0.7	0.000979911	0.010307492
1375341_at	Kua homolog (predicted)	Kua_predicted	0.6	0.000980301	0.010307492
1388718_at	tropomodulin 1	Tmod1	0.6	0.000980741	0.010307492
1387194_at	centaurin, alpha 1	Centa1	0.6	0.000989197	0.010383096
1372658_at	desmuslin	Dmn	0.5	0.000993546	0.010415453
1387133_at	calbindin 2	Calb2	0.4	0.000997101	0.010439415
1389613_at	Transcribed locus	---	0.5	0.001011111	0.01055251
1379022_at	a disintegrin-like and metallopeptidase (reprolysin type) with thrombospondin type 1 motif, 8 (predicted)	Adamts8_predicted	0.5	0.001016074	0.010587682
1370576_at	dynein cytoplasmic 1 light intermediate chain 1	Dync1li1	0.4	0.001016412	0.010587682
1395357_at	microtubule-associated protein 1b	Map1b	0.3	0.001017275	0.010589967
1370205_at	solute carrier organic anion transporter family, member 1c1	Slco1c1	0.4	0.001022642	0.010623617
1368505_at	regulator of G-protein signaling 4	Rgs4	0.4	0.001023028	0.010623617
1368701_at	ATPase, Na+/K+ transporting, alpha 3 polypeptide	Atp1a3	0.4	0.00102321	0.010623617
1395827_at	Transcribed locus	---	0.5	0.001023258	0.010623617
1385442_at	Transcribed locus	---	0.6	0.001025845	0.010633664
1378091_at	Transcribed locus	---	0.6	0.001028233	0.010633707
1378298_at	hypothetical LOC298591 (predicted) /// similar to immunoglobin superfamily, member 21	LOC684776 /// RGD1310117_predicted	0.6	0.001028325	0.010633707

1369043_at	potassium voltage-gated channel, shaker-related subfamily, member 4	Kcna4	0.3	0.001041827	0.010702468
1397619_at	Transcribed locus	---	0.6	0.001042765	0.010702468
1373909_at	similar to transmembrane protein 64	LOC679161	0.6	0.001043048	0.010702468
1393163_at	---	---	0.5	0.00104789	0.010732062
1374300_at	ectonucleoside triphosphate diphosphohydrolase 4 (predicted)	Entpd4_predicted	0.4	0.001049057	0.010737331
1369681_at	ISL1 transcription factor, LIM/homeodomain 1	Isl1	0.4	0.001051136	0.010751919
1372779_at	UDP-GlcNAc:betaGal beta-1,3-N-acetylglucosaminyltransferase 1 (predicted)	B3gnt1_predicted	0.5	0.001057617	0.010789283
1383180_at	protein phosphatase 1, regulatory (inhibitor) subunit 2	Ppp1r2	0.4	0.001057844	0.010789283
1373622_at	hypothetical protein LOC502414	LOC502414	0.7	0.001058069	0.010789283
1378685_at	hypothetical protein LOC689836	LOC689836	0.7	0.00106257	0.010821761
1383511_at	Transcribed locus	---	0.6	0.001065405	0.010826474
1389364_at	Nedd4 family interacting protein 2 (predicted)	Ndfip2_predicted	0.4	0.001066446	0.010826474
1398613_at	Transcribed locus	---	0.4	0.001066982	0.010826474
1371731_at	Mesoderm specific transcript	Mest	0.5	0.001067832	0.010828418
1370455_a_at	olfactomedin 3	Olfm3	0.6	0.001070907	0.010846233
1396676_at	Transcribed locus	---	0.4	0.001072723	0.010857938
1371162_at	nuclear receptor MrgA10 RF-amide G protein-coupled receptor	Mrga10	0.4	0.001075099	0.010871145
1374737_at	similar to tRNA-splicing endonuclease subunit Sen54 (tRNA-intron endonuclease Sen54)	LOC688263 /// LOC690308	0.6	0.001076011	0.010871145
1388756_at	phosphopantothenoylcysteine synthetase	Ppcs	0.5	0.001081993	0.010924872
1371112_at	ret proto-oncogene	Ret	0.4	0.00108302	0.010928531
1387971_a_at	mitogen activated protein kinase 8 interacting protein	Mapk8ip	0.5	0.001085302	0.010937702
1370775_a_at	calcitonin/calcitonin-related polypeptide, alpha	Calca	0.4	0.001085385	0.010937702
1388540_at	---	---	0.6	0.001094077	0.010998666
1374904_at	sine oculis homeobox homolog 1 (Drosophila)	Six1	0.6	0.001094651	0.010998666
1384305_at	solute carrier family 45, member 1	Slc45a1	0.7	0.001098819	0.011027078
1390385_at	similar to D-glucuronyl C5-epimerase (predicted)	RGD1565253_predicted	0.6	0.001101188	0.011037375
1384187_at	adaptor-related protein complex 1, sigma 2 subunit (predicted)	Ap1s2_predicted	0.4	0.001103626	0.01105508
1382212_at	Transcribed locus	---	0.7	0.001111073	0.011100285
1397241_at	similar to hypothetical protein MGC47816 (predicted)	RGD1306880_predicted	0.2	0.001112807	0.011102756
1382226_at	Similar to RNA binding motif, single stranded interacting protein 3 isoform 1	LOC680726	0.5	0.001120499	0.011156156
1382670_at	La ribonucleoprotein domain family, member 1 (predicted)	Larp1_predicted	0.4	0.001128974	0.011226944
1383457_at	Similar to hypothetical protein DKFZp761N1114 (predicted)	RGD1566090_predicted	0.6	0.001129763	0.011227998
1369410_at	---	---	0.5	0.001151904	0.011420447
1381445_at	Estrogen-related receptor gamma	Esrrg	0.4	0.001158907	0.011476036
1383546_at	glycerophosphodiester phosphodiesterase domain containing 2 (predicted)	Gdpd2_predicted	0.6	0.001160774	0.011487611
1391028_at	Similar to nuclear receptor interacting protein 2	LOC689619	0.4	0.001162023	0.01149306
1392987_at	similar to hypothetical protein (predicted)	RGD1561605_predicted	0.6	0.001166896	0.011527387
1391434_at	RAB3A, member RAS oncogene family	Rab3a	0.6	0.001173712	0.011566938
1385589_at	similar to CG10806-PB, isoform B	LOC679958 /// LOC686829	0.7	0.001174675	0.011569492
1389597_at	piggyBac transposable element derived 5 (predicted)	Pgbd5_predicted	0.6	0.001178001	0.011581449
1375861_at	similar to nucleosome assembly protein 1-like 5	LOC682844 /// MGC105733	0.6	0.001180968	0.011594924
1370406_a_at	decay accelarating factor 1	Daf1	0.3	0.001185475	0.011623574
1377001_at	neuron-glia-CAM-related cell adhesion molecule	Nrcam	0.6	0.001185819	0.011623574
1389347_at	phosphatidylinositol transfer protein, membrane-associated 1	Pitpnm1	0.7	0.001195144	0.011701033
1379754_at	Staufen, RNA binding protein, homolog 2 (Drosophila)	Stau2	0.6	0.001199326	0.011728008
1390581_at	---	---	0.5	0.001204399	0.011763629
1379750_at	roundabout homolog 2 (Drosophila)	Robo2	0.6	0.001206285	0.011769264

1396025_at	G protein-coupled receptor 61 (predicted)	Gpr61_predicted	0.6	0.001209465	0.011778151
1379894_at	similar to 3632451O06Rik protein (predicted)	RGD1310110_predicted	0.5	0.001209716	0.011778151
1386929_at	hexokinase 1	Hk1	0.5	0.001214318	0.011797498
1395673_at	Transcribed locus	---	0.5	0.001214322	0.011797498
1382920_at	leucine-rich repeat-containing 8	Lrrc8	0.7	0.001215371	0.011800719
1374841_at	---	---	0.5	0.001216782	0.011807452
1368242_at	potassium voltage gated channel, Shab-related subfamily, member 1	Kcnb1	0.6	0.001221345	0.011844743
1391948_at	B-cell leukemia/lymphoma 11B (predicted)	Bcl11b_predicted	0.6	0.001224964	0.011862932
1371531_at	similar to mammalian retrotransposon derived 8b	LOC678880	0.7	0.001228616	0.011872927
1387998_at	exocyst complex component 8	Exoc8	0.4	0.001231757	0.011889638
1378987_at	Similar to CG18769-PB, isoform B	LOC294560	0.5	0.001233121	0.0118958
1398189_at	Transcribed locus	---	0.4	0.001233842	0.0118958
1395739_at	similar to RIKEN cDNA C920006C10 (predicted)	RGD1305976_predicted	0.6	0.001235568	0.011898489
1373510_at	vesicle-associated membrane protein 1	Vamp1	0.4	0.001247817	0.011994706
1372848_at	Transcribed locus	---	0.4	0.001248476	0.011994706
1370351_at	tudor domain containing 7	Tdrd7	0.7	0.001250683	0.012001889
1373167_at	Transcribed locus	---	0.6	0.001251673	0.012003577
1378320_at	Transcribed locus	---	0.7	0.001255267	0.01200912
1367963_at	G protein beta subunit-like	Gbl	0.7	0.001255817	0.01200912
1378876_at	Transcribed locus	---	0.3	0.00126624	0.012081474
1372561_at	stromal membrane-associated protein 1-like	Smap1l	0.6	0.001272277	0.012131252
1370059_at	neurofilament, light polypeptide	Nefl	0.5	0.001277781	0.012162579
1382325_at	glycine C-acetyltransferase (2-amino-3-ketobutyrate-coenzyme A ligase)	Gcat	0.6	0.001285875	0.012225477
1371359_at	myeloid leukemia factor 2 (predicted)	Mlf2_predicted	0.5	0.001289225	0.012243167
1379405_at	glucocorticoid receptor DNA binding factor 1 (predicted) /// similar to Glucocorticoid receptor DNA-binding factor 1 (GAP-associated protein p190)	Grlf1_predicted /// LOC686520	0.6	0.001302187	0.012344879
1376416_at	similar to KIAA1882 protein	RGD1309461	0.5	0.001305739	0.0123643
1395718_at	Transcribed locus	---	0.7	0.001317664	0.012470042
1376501_at	Rho GTPase activating protein 8	Arhgap8	0.6	0.00132216	0.012478539
1373301_at	protein phosphatase 1, regulatory (inhibitor) subunit 13B (predicted)	Ppp1r13b_predicted	0.6	0.001322837	0.012478539
1380625_at	CCR4-NOT transcription complex, subunit 4	Cnot4	0.7	0.001323723	0.012478539
1394894_at	WD repeat domain 37 (predicted)	Wdr37_predicted	0.6	0.001323872	0.012478539
1372288_at	similar to D11Bwg0280e protein	RGD1309256	0.7	0.001337044	0.012566682
1380561_at	isoleucine-tRNA synthetase 2, mitochondrial (predicted)	Iars2_predicted	0.5	0.00134595	0.012635952
1383362_at	Transcribed locus	---	0.7	0.001355073	0.012707095
1394883_at	Transcribed locus	---	0.5	0.00135619	0.012710321
1396603_at	Transcribed locus	---	0.5	0.001357014	0.012710799
1385954_at	Transcribed locus	---	0.6	0.001358152	0.012714221
1387961_at	opioid binding protein/cell adhesion molecule-like	Opcml	0.5	0.001359155	0.012715638
1367882_at	microtubule-associated protein 1 A	Mtap1a	0.6	0.001359913	0.012715638
1385364_at	Transcribed locus	---	0.5	0.001367459	0.012748378
1395792_at	Poly(rC) binding protein 2	Pcbp2	0.6	0.001372003	0.012748378
1372091_at	MID1 interacting G12-like protein	Mig12	0.5	0.001372042	0.012748378
1398295_at	solute carrier family 29 (nucleoside transporters), member 1	Slc29a1	0.6	0.001386337	0.012826724
1394973_at	Phosphodiesterase 1C	Pde1c	0.6	0.001387953	0.012826724
1386953_at	hydroxysteroid 11-beta dehydrogenase 1	Hsd11b1	0.6	0.001396726	0.012877437
1391601_at	leucine rich repeat protein 2, neuronal (predicted)	Lrrn2_predicted	0.7	0.00140278	0.012904323
1395947_at	Transcribed locus	---	0.6	0.00140407	0.01290897
1390393_at	Transcribed locus	---	0.6	0.001406652	0.012923912
1367499_at	solute carrier family 35, member C1 (predicted)	Slc35c1_predicted	0.7	0.001414986	0.012971904
1394627_at	Sorting nexin 19 (predicted)	Snx19_predicted	0.6	0.001415422	0.012971904
1394448_at	Transcribed locus	---	0.7	0.001415647	0.012971904
1385184_at	Bwk1 leukemia-related gene	Bwk1	0.7	0.001417639	0.012975695
1379899_at	Glycosyltransferase 25 domain containing 2 (predicted)	Glt25d2_predicted	0.5	0.001419238	0.012975993
1369690_at	N-ethylmaleimide sensitive fusion protein	Nsf	0.5	0.001431969	0.013063244

1387235_at	chromogranin A	Chga	0.4	0.00144111	0.013139346
1391405_at	similar to mKIAA0960 protein (predicted)	RGD1566201_predicted	0.6	0.001445359	0.013158248
1377621_at	Transcribed locus	---	0.7	0.001448031	0.013165959
1370568_at	adrenergic receptor, alpha 2c	Adra2c	0.4	0.00145006	0.013169841
1393250_at	---	---	0.6	0.001451493	0.01317486
1370214_at	parvalbumin	Pvalb	0.3	0.001452214	0.01317486
1382846_at	Transcribed locus	---	0.6	0.001453694	0.013181012
1368182_at	acyl-CoA synthetase long-chain family member 6	Acsl6	0.4	0.001455905	0.013186526
1377007_at	Transcribed locus	---	0.6	0.001457579	0.013194418
1370818_at	2-4-dienoyl-Coenzyme A reductase 2, peroxisomal	Decr2	0.6	0.001459786	0.013206469
1388000_at	solute carrier family 24 (sodium/potassium/calcium exchanger), member 2	Slc24a2	0.3	0.001460516	0.013206469
1399059_at	enhancer of zeste homolog 1 (Drosophila) (predicted)	Ezh1_predicted	0.6	0.001461383	0.013207045
1374019_at	RGD1564379 (predicted)	RGD1564379_predicted	0.6	0.001467927	0.013237097
1377366_at	Transcribed locus	---	0.6	0.001474016	0.013270178
1393141_at	Aspartate-beta-hydroxylase (predicted)	Asph_predicted	0.6	0.001487078	0.013350227
1373654_at	annexin A8	Anxa8	0.4	0.001488639	0.013350227
1387042_at	calcium channel, voltage-dependent, beta 3 subunit	Cacnb3	0.5	0.001489401	0.013350227
1388821_at	similar to Tribbles homolog 2 (predicted)	RGD1564451_predicted	0.6	0.001491317	0.013360125
1374630_at	chloride intracellular channel 3	Clic3	0.6	0.001493542	0.013370578
1396034_at	carboxylesterase 615	LOC307660	0.4	0.001500969	0.013417359
1380859_at	Transcribed locus	---	0.5	0.001510037	0.013476442
1381871_at	Transcribed locus	---	0.6	0.00152156	0.013561498
1376424_at	Transcribed locus	---	0.7	0.001524087	0.013562281
1383254_at	---	---	0.5	0.001536926	0.01365714
1368148_at	nerve growth factor receptor (TNFR superfamily, member 16)	Ngfr	0.4	0.001546826	0.013727024
1369972_at	serine (or cysteine) peptidase inhibitor, clade B, member 5	Serpinb5	0.6	0.001551471	0.013750756
1374532_at	prostaglandin E synthase 2 (predicted)	Ptges2_predicted	0.6	0.001552499	0.013750756
1393066_at	Cadherin 1	Cdh1	0.7	0.001552509	0.013750756
1375873_at	Transcribed locus	---	0.7	0.001553314	0.013750756
1388927_at	RAB, member of RAS oncogene family-like 4 (predicted)	Rabl4_predicted	0.7	0.001558126	0.013778521
1393037_at	Transcribed locus	---	0.7	0.001563314	0.013809555
1367773_at	solute carrier family 25, member 1	Slc25a1	0.6	0.001577174	0.013909579
1394821_at	Transcribed locus	---	0.4	0.001588458	0.013989624
1393171_at	Transcribed locus	---	0.5	0.001588648	0.013989624
1388624_at	similar to Hypothetical UPF0184 protein C9orf16 homolog (predicted)	RGD1561113_predicted	0.5	0.00159091	0.013989624
1367707_at	fatty acid synthase	Fasn	0.5	0.001593636	0.013989624
1391437_at	similar to BC002216 protein	MGC94339	0.5	0.001594507	0.013989624
1389787_at	PTK7 protein tyrosine kinase 7 (predicted)	Ptk7_predicted	0.6	0.001598134	0.014011782
1389844_at	FK506 binding protein 4	Fkbp4	0.3	0.001603354	0.014031712
1382567_at	---	---	0.5	0.001607712	0.014058291
1368627_at	regucalcin	Rgn	0.5	0.001611421	0.014083235
1388644_at	---	---	0.7	0.001612774	0.01408758
1374593_at	protein kinase C, epsilon	Prkce	0.5	0.001620321	0.014145991
1368853_at	visinin-like 1	Vsnl1	0.3	0.001624669	0.014162022
1388034_at	kinesin family member 1B	Kif1b	0.5	0.001624741	0.014162022
1374559_at	ring finger protein 26 (predicted)	Rnf26_predicted	0.7	0.001627879	0.014181861
1390077_at	Translocation associated membrane protein 1	Tram1	0.4	0.001641708	0.014279649
1390863_at	solute carrier family 19 (thiamine transporter), member 2	Slc19a2	0.6	0.001645799	0.014306157
1396246_at	Transcribed locus	---	0.7	0.001646559	0.014306157
1387058_at	phosphatidylcholine transfer protein	Pctp	0.5	0.001647865	0.014306157
1383298_at	LOC363015 (predicted)	RGD1310444_predicted	0.6	0.001648235	0.014306157
1389032_at	mucolipin 1 (predicted)	Mcoln1_predicted	0.6	0.001651015	0.014315181

1369993_at	calcium/calmodulin-dependent protein kinase II gamma	Camk2g	0.5	0.001653975	0.014333284
1375307_at	chromobox homolog 6	Cbx6	0.5	0.001657403	0.014355429
1398593_at	similar to RIKEN cDNA 2310003M01	LOC298139	0.6	0.001662112	0.014388637
1367726_at	thyroid hormone receptor alpha	Thra	0.6	0.001667977	0.014431818
1394517_at	CDNA clone IMAGE:7103416	---	0.5	0.00166932	0.014435843
1376669_at	Transcribed locus	---	0.6	0.001671477	0.014438933
1392501_at	Transcribed locus	---	0.7	0.001672311	0.014438933
1373938_at	WD repeat domain 24	Wdr24	0.7	0.001684184	0.014492598
1393699_at	Transcribed locus	---	0.5	0.001685575	0.014492598
1368700_at	phospholipase C-like 1	Plcl1	0.6	0.001692715	0.014533875
1397571_at	Cancer susceptibility candidate 3	Casc3	0.5	0.001699364	0.014558327
1386310_at	Transcribed locus	---	0.3	0.001700491	0.014559957
1395637_at	aspartate beta-hydroxylase domain containing 2	Asphd2	0.6	0.00171132	0.014614638
1375212_at	ankyrin repeat domain 52 (predicted)	Ankrd52_predicted	0.6	0.001724583	0.014697377
1390566_a_at	creatine kinase, mitochondrial 1, ubiquitous	Ckmt1	0.3	0.001731793	0.014735915
1373896_at	synaptotagmin I	Syt1	0.6	0.001733319	0.014741281
1389447_at	similar to prolyl-4-hydroxylase-alpha NE2 CG9720-PA (predicted)	RGD1311848_predicted	0.7	0.001740387	0.014780346
1386379_at	Transcribed locus	---	0.5	0.001743166	0.014780346
1389274_at	dephospho-CoA kinase domain containing	Dcakd	0.7	0.001753949	0.014847636
1375647_at	Transcribed locus	---	0.6	0.00176586	0.014925419
1368261_at	neurexin 3	Nrxn3	0.6	0.001775994	0.014980285
1393547_at	similar to hypothetical protein C630023L15 (predicted)	RGD1560399_predicted	0.4	0.001778199	0.014991192
1393614_at	---	---	0.5	0.001783443	0.015020007
1397579_x_at	Transcribed locus	---	0.6	0.00178506	0.015025934
1393961_at	Transcribed locus	---	0.7	0.001802225	0.015120853
1375048_at	---	---	0.7	0.001802772	0.015120853
1388522_at	similar to T-cell activation protein phosphatase 2C (predicted)	RGD1310383_predicted	0.7	0.001807197	0.015147273
1376788_at	death associated protein kinase 1 (predicted)	Dapk1_predicted	0.6	0.001822105	0.015236394
1385924_at	Transcribed locus	---	0.5	0.001830737	0.015254283
1374934_at	Transcribed locus	---	0.5	0.001841755	0.01533832
1384156_at	oxysterol binding protein-like 7 (predicted)	Osbpl7_predicted	0.7	0.001847267	0.015361988
1376583_at	---	---	0.6	0.001847399	0.015361988
1374414_at	similar to RIKEN cDNA 1300010M03	RGD1306844	0.7	0.001859541	0.015443038
1385219_a_at	---	---	0.4	0.001869618	0.015501603
1374899_at	similar to hypothetical protein MGC20579 (predicted)	RGD1566042_predicted	0.4	0.001869843	0.015501603
1382234_at	Hypothetical protein LOC311254	LOC311254	0.7	0.00187529	0.015528128
1378130_at	---	---	0.5	0.001876718	0.015528128
1387456_at	staufen, RNA binding protein, homolog 2 (Drosophila)	Stau2	0.4	0.001894648	0.015643039
1395429_at	Cholinergic receptor, nicotinic, alpha polypeptide 7	Chrna7	0.5	0.001901316	0.015683473
1377598_at	---	---	0.5	0.001913386	0.015772205
1387878_at	glutamate dehydrogenase 1	Glud1	0.4	0.001924665	0.015836363
1397665_at	Transcribed locus	---	0.6	0.001940199	0.015932299
1385935_at	Transcribed locus	---	0.7	0.001941831	0.015932991
1381526_at	par-6 (partitioning defective 6,) homolog alpha (C. elegans)	Pard6a	0.8	0.001942342	0.015932991
1393559_at	Stanniocalcin 1	Stc1	0.5	0.001946865	0.015955173
1370625_at	Fas apoptotic inhibitory molecule 2	Faim2	0.2	0.001951619	0.015970267
1376924_a_at	palmdelphin	Palmd	0.7	0.001957829	0.015988765
1370887_at	transforming growth factor beta 1 induced transcript 1	Tgfb1i1	0.7	0.001958164	0.015988765
1389955_at	Adaptor-related protein complex 1, sigma 2 subunit (predicted)	Ap1s2_predicted	0.6	0.00195874	0.015988765
1380681_at	similar to ring finger protein 180 /// hypothetical protein LOC685384	LOC683640 /// LOC685384	0.6	0.00196608	0.016024817
1373666_at	Rap guanine nucleotide exchange factor (GEF) 5	Rapgef5	0.6	0.001972024	0.016057348
1370812_at	Bcl2-like 1	Bcl2l1	0.7	0.001977244	0.016091889
1380799_at	Transcribed locus	---	0.4	0.001978592	0.016094895
1371993_at	Copine III (predicted)	Cpne3_predicted	0.6	0.001986188	0.016142781
1389008_at	Sprouty-related, EVH1 domain containing 2	Spred2	0.4	0.001987971	0.016143461

1380028_at	similar to ADP-ribosylation factor-like protein 4C (ADP-ribosylation factor-like 7)	LOC367311	0.7	0.001996421	0.016199871
1397632_at	SLIT and NTRK-like family, member 1 (predicted)	Slitrk1_predicted	0.6	0.002000279	0.016220971
1391097_at	---	---	0.6	0.00200389	0.016236445
1377197_at	Transcribed locus	---	0.6	0.002005824	0.016239676
1368854_at	visinin-like 1	Vsnl1	0.4	0.002006263	0.016239676
1372408_at	golgi associated, gamma adaptin ear containing, ARF binding protein 2	Gga2	0.4	0.002011424	0.016273441
1394986_at	Similar to KIAA1838 protein (predicted)	RGD1310304_predicted	0.5	0.002014249	0.016277518
1372528_at	N-ethylmaleimide sensitive fusion protein	Nsf	0.5	0.002014897	0.016277518
1381457_at	Transcribed locus	---	0.6	0.002027342	0.016353956
1386149_at	---	---	0.6	0.002042353	0.016442795
1374087_at	---	---	0.7	0.002045398	0.016443272
1378665_at	Protocadherin 9 (predicted)	Pcdh9_predicted	0.7	0.002049272	0.016458219
1378470_at	progestin and adipoQ receptor family member V	Paqr5	0.4	0.00205165	0.016466059
1379510_at	Transcribed locus	---	0.5	0.002070496	0.016604346
1372894_at	transmembrane protein 115 (predicted)	Tmem115_predicted	0.5	0.002078553	0.01665059
1391414_at	similar to RIKEN cDNA D930015E06 /// similar to KIAA0922 protein	LOC679811 /// LOC683642	0.7	0.002081704	0.016661733
1382812_at	similar to Protein Njmu-R1 (predicted)	RGD1310429_predicted	0.5	0.002096029	0.016753818
1376640_at	Hypothetical protein LOC680259	LOC680259	0.5	0.002096264	0.016753818
1370045_at	polymerase (DNA directed), gamma	Polg	0.7	0.002100246	0.016777486
1378144_at	Transcribed locus	---	0.6	0.002112569	0.016851372
1379661_at	---	---	0.4	0.002120757	0.016883939
1390441_at	Development and differentiation enhancing (predicted)	Ddef1_predicted	0.6	0.002124983	0.016909399
1396227_at	mitochondrial ribosome recycling factor	Mrrf	0.5	0.002129048	0.016917156
1368081_at	ATP-binding cassette, sub-family A (ABC1), member 2	Abca2	0.6	0.00213162	0.016917156
1381800_at	Transcribed locus	---	0.4	0.002132129	0.016917156
1373396_at	similar to RIKEN cDNA 2610304G08 gene (predicted)	RGD1304782_predicted	0.7	0.002133847	0.016922619
1385002_at	similar to c-myc promoter binding protein (predicted)	RGD1562639_predicted	0.7	0.002147318	0.017004855
1389376_at	Transcribed locus	---	0.6	0.002163098	0.017098206
1396333_at	Transcribed locus	---	0.6	0.002163264	0.017098206
1369530_at	insulin related protein 2 (islet 2)	Isl2	0.3	0.002168699	0.017124704
1372722_at	Transcribed locus	---	0.7	0.002171727	0.017140388
1370146_at	glycine receptor, beta subunit	Glrb	0.3	0.002183697	0.017210082
1389598_at	---	---	0.4	0.002188139	0.01722858
1382566_at	Transcribed locus	---	0.5	0.002190252	0.017236972
1389416_at	RGD1560398 (predicted)	RGD1560398_predicted	0.4	0.002193527	0.017254487
1389465_at	Transcribed locus	---	0.5	0.002196385	0.017268713
1377980_at	similar to chromosome 20 open reading frame 102 (predicted) /// similar to stromal cell derived factor receptor 1	LOC683938 /// RGD1305725_predicted	0.4	0.002200283	0.017291089
1390145_at	Transcribed locus	---	0.6	0.002201616	0.017293304
1390113_a_at	La ribonucleoprotein domain family, member 1 (predicted)	Larp1_predicted	0.7	0.002209987	0.017336284
1392101_s_at	Transcribed locus	---	0.5	0.002210217	0.017336284
1376211_a_at	potassium channel tetramerisation domain containing 6 (predicted)	Kctd6_predicted	0.5	0.00221025	0.017336284
1382183_at	Transcribed locus	---	0.7	0.002217881	0.01735549
1367733_at	carbonic anhydrase 2	Ca2	0.5	0.002217974	0.01735549
1395546_at	Transcribed locus	---	0.6	0.002221536	0.017375092
1374084_at	Transcribed locus	---	0.6	0.002225103	0.017393241
1380314_at	similar to hypothetical protein FLJ38984 (predicted)	RGD1563072_predicted	0.2	0.002226405	0.017393241
1394078_at	---	---	0.3	0.00223284	0.017397503
1398346_at	Transcribed locus	---	0.5	0.002233921	0.017397503
1372618_at	similar to hypothetical protein FLJ13045 (predicted)	RGD1307615_predicted	0.7	0.002238049	0.017421405

1387261_at	protein phosphatase 3, catalytic subunit, beta isoform	Ppp3cb	0.5	0.002242049	0.017434087
1381206_at	Transcribed locus	---	0.6	0.002242858	0.017434087
1384389_at	Transcribed locus	---	0.5	0.002244155	0.017435927
1372555_at	similar to RIKEN cDNA E030034P13 (predicted)	RGD1309385_predicted	0.7	0.002254342	0.017488468
1374235_at	Down syndrome critical region gene 1-like 1	Dscr1l1	0.5	0.00225517	0.017488468
1392778_at	Transcribed locus, weakly similar to XP_001056676.1 similar to Discs large homolog 5 (Placenta and prostate DLG) (Discs large protein P-dlg) [Rattus norvegicus]	---	0.6	0.002259563	0.017500096
1376239_at	ATPase, H+ transporting, V1 subunit C, isoform 2	Atp6v1c2	0.7	0.002259862	0.017500096
1387802_at	discs, large (Drosophila) homolog-associated protein 1	Dlgap1	0.4	0.002267044	0.017542697
1389806_at	Transcribed locus	---	0.6	0.00228081	0.017620839
1379268_at	Transcribed locus	---	0.5	0.002282498	0.017625599
1389164_at	hect domain and RLD 3 (predicted)	Herc3_predicted	0.6	0.002294009	0.017706173
1389066_at	Down syndrome critical region gene 1-like 1	Dscr1l1	0.6	0.002297062	0.017721422
1371006_at	jagged 2	Jag2	0.5	0.002298607	0.017722476
1374859_at	---	---	0.5	0.002299353	0.017722476
1384393_at	Transcribed locus	---	0.4	0.002305602	0.017752701
1374249_at	similar to Hypothetical protein MGC38513	RGD1304580	0.7	0.00230683	0.017752701
1371699_at	Transcribed locus, moderately similar to XP_001136961.1 fibrinogen, beta chain isoform 1 [Pan troglodytes]	---	0.7	0.002307592	0.017752701
1389507_at	neural precursor cell expressed, developmentally down-regulated 4-like	Nedd4l	0.6	0.002313518	0.017777589
1374528_at	Regulator of G-protein signalling 3	Rgs3	0.4	0.002315151	0.017777589
1374496_at	similar to RIKEN cDNA 1200009O22; EST AI316813	RGD1310827	0.6	0.002319565	0.017794875
1375226_at	TBC1 domain family, member 10b (predicted)	Tbc1d10b_predicted	0.6	0.00232265	0.017796757
1382602_at	---	---	0.7	0.002330164	0.017834593
1368821_at	follistatin-like 1	Fstl1	0.3	0.002333656	0.017844708
1390595_at	Transcribed locus	---	0.5	0.002335546	0.017845331
1375242_at	similar to tumor suppressor candidate 5	LOC687797	0.2	0.002348118	0.017905352
1384530_at	similar to RIKEN cDNA 1810048J11 (predicted) /// hypothetical protein LOC687944 /// hypothetical protein LOC690208 /// similar to B0511.12	LOC687944 /// LOC690208 /// LOC690217 /// RGD1305147_predicted	0.7	0.002352257	0.017917431
1376187_at	Solute carrier family 35 (UDP-glucuronic acid/UDP-N-acetylgalactosamine dual transporter), member D1 (predicted)	Slc35d1_predicted	0.7	0.002358444	0.017950803
1384309_at	Transcribed locus	---	0.7	0.002370773	0.018019626
1374761_at	golgi associated, gamma adaptin ear containing, ARF binding protein 1	Gga1	0.7	0.002373969	0.018035586
1389681_at	Poliovirus receptor-related 2 (herpesvirus entry mediator B)	Pvrl2	0.6	0.002388557	0.018129663
1378229_at	Leucine rich repeat and fibronectin type III domain containing 1 (predicted)	Lrfn1_predicted	0.6	0.002402262	0.018216879
1387141_at	dihydropyrimidinase-like 5	Dpysl5	0.6	0.002412869	0.018272038
1370922_at	cortexin	Ctxn	0.4	0.002424525	0.018351856
1384248_at	Transcribed locus	---	0.6	0.00243387	0.018397192
1399039_at	similar to DnaJ homolog subfamily B member 10 (mDJ8) /// similar to DnaJ (Hsp40) homolog, subfamily B, member 10 isoform 2	LOC681650 /// LOC689593	0.6	0.002450584	0.018504306
1378498_at	---	---	0.3	0.002456879	0.018537036
1392865_at	---	---	0.6	0.002479491	0.018681938
1377614_at	similar to hypothetical protein FLJ90652	LOC293494	0.7	0.002483555	0.018703992
1378888_at	Transcribed locus	---	0.5	0.002494384	0.018751203
1372446_at	protein phosphatase 1, regulatory (inhibitor) subunit 2	Ppp1r2	0.5	0.00250198	0.018782553
1391574_at	---	---	0.5	0.002508587	0.018814976
1382057_at	---	---	0.7	0.002512784	0.018829281
1392763_at	1-acylglycerol-3-phosphate O-acyltransferase 1	Agpat1	0.6	0.00251615	0.018845921
1386969_at	neuritin	Nrn1	0.3	0.002524599	0.018900593

1382967_at	G protein-coupled receptor 64	Gpr64	0.6	0.0025358	0.018967186
1373249_at	Transcribed locus	---	0.7	0.002542013	0.018979133
1376706_at	Transcribed locus	---	0.7	0.002584238	0.019198918
1390645_at	Transcribed locus	---	0.6	0.002592422	0.019224599
1376302_at	Coilin	Coil	0.7	0.002595684	0.019231443
1391995_at	tripartite motif protein 37 (predicted)	Trim37_predicted	0.5	0.00260812	0.019306193
1388757_at	Adrenergic receptor kinase, beta 1	Adrbk1	0.6	0.002611823	0.019324905
1379579_at	LEA_4 domain containing protein RGD1359600	RGD1359600	0.6	0.002616481	0.019350664
1372862_at	RAB22A, member RAS oncogene family (predicted)	Rab22a_predicted	0.6	0.002631306	0.019434093
1389800_at	PWWP domain containing 2 (predicted)	Pwwp2_predicted	0.6	0.002635825	0.019458732
1385606_at	brain protein 16	Brp16	0.5	0.002640001	0.019480814
1393040_at	Transcribed locus	---	0.5	0.002648723	0.019527651
1390947_at	similar to RIKEN cDNA D930036F22 gene (predicted)	RGD1561116_predicted	0.7	0.002650383	0.019531135
1393587_a_at	similar to RIKEN cDNA 2310047O13 (predicted)	RGD1309605_predicted	0.6	0.002655056	0.019544829
1390433_at	Transcribed locus	---	0.7	0.002657437	0.019544829
1384043_at	Transcribed locus	---	0.6	0.002658985	0.019544829
1384269_at	Transcribed locus	---	0.4	0.002659371	0.019544829
1390558_at	staufen, RNA binding protein, homolog 2 (Drosophila)	Stau2	0.7	0.002661023	0.019548235
1370440_at	solute carrier family 15, member 4	Slc15a4	0.6	0.002663138	0.019553254
1372345_at	---	---	0.5	0.002664083	0.019553254
1399016_at	MYST histone acetyltransferase 2	Myst2	0.4	0.002668655	0.019569344
1371817_at	similar to myo-inositol 1-phosphate synthase A1	LOC290651	0.5	0.002671534	0.019573003
1373631_at	RAP1, GTPase activating protein 1	Rap1ga1	0.6	0.002682771	0.019623211
1370571_at	solute carrier organic anion transporter family, member 3a1	Slco3a1	0.5	0.002683159	0.019623211
1378591_at	Transcribed locus	---	0.6	0.002693678	0.019674463
1370747_at	fibroblast growth factor 9	Fgf9	0.4	0.002694328	0.019674463
1391653_at	gamma-aminobutyric acid A receptor, gamma 2	Gabrg2	0.4	0.002707252	0.019729617
1393007_at	Transcribed locus	---	0.5	0.002707305	0.019729617
1369167_at	glial cell line derived neurotrophic factor family receptor alpha 2	Gfra2	0.5	0.002711041	0.019748096
1389027_at	lectin, mannose-binding 2-like (predicted)	Lman2l_predicted	0.6	0.002715946	0.019772605
1393659_at	translocation associated membrane protein 1-like 1 (predicted)	Tram1l1_predicted	0.5	0.00271681	0.019772605
1388184_at	isoprenylcysteine carboxyl methyltransferase	Icmt	0.5	0.002720129	0.019788005
1390495_at	Transcribed locus	---	0.5	0.002724003	0.019807424
1374000_at	helicase with zinc finger domain (predicted)	Helz_predicted	0.5	0.002726451	0.019808298
1390501_at	Transcribed locus, moderately similar to XP_001149369.1 secernin 3 isoform 5 [Pan troglodytes]	---	0.5	0.002731187	0.019816675
1383322_at	RAS-like family 11 member B	Rasl11b	0.6	0.002731285	0.019816675
1374196_at	lanC (bacterial lantibiotic synthetase component C)-like 1	Lancl1	0.4	0.002745446	0.019875482
1375435_at	hypothetical LOC308765 (predicted) /// hypothetical protein LOC685841	LOC685841 /// RGD1311021_predicted	0.7	0.00275046	0.019896035
1391229_at	calcium/calmodulin-dependent protein kinase I gamma	Camk1g	0.6	0.002762619	0.019955885
1376869_at	Transcribed locus	---	0.6	0.002771383	0.019980855
1376633_at	Transcribed locus	---	0.6	0.002773364	0.019980855
1384301_at	Transcribed locus	---	0.6	0.002778884	0.020000825
1390141_at	methylenetetrahydrofolate dehydrogenase (NADP+ dependent) 1-like (predicted)	Mthfd1l_predicted	0.5	0.002779784	0.020000825
1390741_at	similar to KIAA1086 protein (predicted)	RGD1304737_predicted	0.7	0.002781756	0.020006259
1372908_at	splA/ryanodine receptor domain and SOCS box containing 3 (predicted)	Spsb3_predicted	0.7	0.002792671	0.020023475
1384197_at	---	---	0.6	0.00280223	0.020074511
1393618_at	Retinoic acid receptor responder (tazarotene induced) 2	Rarres2	0.7	0.00285656	0.020400784
1386569_at	Similar to CG18769-PB, isoform B	LOC294560	0.3	0.002857697	0.020400784

1394605_at	similar to CDNA sequence BC017647 (predicted)	RGD1566149_predicted	0.6	0.002859849	0.020407286
1368115_at	claudin 3	Cldn3	0.7	0.002861388	0.020409418
1373055_at	Transcribed locus	---	0.7	0.002871308	0.020471295
1391595_at	La ribonucleoprotein domain family, member 6 (predicted)	Larp6_predicted	0.6	0.002873039	0.020474757
1389744_at	---	---	0.6	0.002878514	0.020496015
1388349_at	---	---	0.4	0.002889761	0.020567193
1390512_at	Transcribed locus	---	0.5	0.00290785	0.020634763
1370122_at	RAB27B, member RAS oncogene family	Rab27b	0.5	0.002908036	0.020634763
1398902_at	similar to mKIAA0664 protein (predicted)	RGD1307222_predicted	0.5	0.002914477	0.020666823
1383155_at	amyotrophic lateral sclerosis 2 (juvenile) chromosome region, candidate 13 (predicted)	Als2cr13_predicted	0.7	0.002917211	0.020673111
1381906_at	tumor protein D52 (predicted)	Tpd52_predicted	0.6	0.002926728	0.020731617
1395694_at	F-box only protein 9	Fbxo9	0.5	0.002933161	0.020768243
1378215_at	GIY-YIG domain containing 2	Giyd2	0.7	0.00293612	0.020780248
1389594_at	similar to Protein C20orf22 homolog	LOC499913	0.3	0.002995273	0.021130037
1374175_at	similar to porcupine-D (predicted)	RGD1564947_predicted	0.6	0.002998707	0.021130037
1393622_at	---	---	0.6	0.002999281	0.021130037
1390167_at	similar to hypothetical protein FLJ30373 (predicted)	RGD1306256_predicted	0.4	0.003000959	0.021130037
1388208_a_at	ret proto-oncogene	Ret	0.4	0.003005635	0.021152135
1396189_at	Transcribed locus	---	0.6	0.003025739	0.021234478
1374653_at	similar to CG12125-PA (predicted)	RGD1310887_predicted	0.7	0.003027444	0.021234478
1368008_at	prominin 1	Prom1	0.6	0.003028702	0.021234478
1369408_at	deleted in bladder cancer chromosome region candidate 1 (human)	Dbccr1	0.5	0.00303352	0.021250142
1376780_at	---	---	0.6	0.003038232	0.02127409
1387664_at	ATPase, H transporting, lysosomal V1 subunit B2	Atp6v1b2	0.3	0.003041903	0.021290734
1379284_at	similar to RIKEN cDNA 2810457I06 (predicted)	RGD1310357_predicted	0.6	0.003047658	0.021312877
1387793_at	solute carrier family 9 (sodium/hydrogen exchanger), isoform 3 regulator 1	Slc9a3r1	0.6	0.003060173	0.021369369
1376345_at	dopamine receptor D1 interacting protein	Drd1ip	0.5	0.003071264	0.021415848
1373658_at	Rac GTPase-activating protein 1 (predicted)	Racgap1_predicted	0.7	0.003071497	0.021415848
1373339_at	---	---	0.6	0.003075208	0.021432633
1381269_at	Transcribed locus	---	0.6	0.003099728	0.021530544
1368875_a_at	neurexin 2	Nrxn2	0.7	0.003120166	0.021640669
1367740_at	creatine kinase, brain	Ckb	0.5	0.003146361	0.02179922
1383967_at	Transcribed locus	---	0.7	0.0031511	0.021822862
1379318_at	Similar to RIKEN cDNA 2610019F03	LOC498662	0.6	0.003155266	0.021837654
1384360_at	Transcribed locus	---	0.5	0.003174999	0.021951405
1368047_at	solute carrier family 13 (sodium-dependent dicarboxylate transporter), member 3	Slc13a3	0.6	0.003186103	0.022009667
1370802_at	similar to integrin beta-5 (predicted)	RGD1563276_predicted	0.5	0.003196357	0.022071235
1374278_at	Transcribed locus	---	0.7	0.003200918	0.022084186
1372475_at	PTEN induced putative kinase 1 (predicted)	Pink1_predicted	0.5	0.003206143	0.022107952
1382100_at	similar to RIKEN cDNA 2310047O13 (predicted)	RGD1309605_predicted	0.7	0.003207991	0.022107952
1383692_at	hypothetical protein LOC681037 /// hypothetical protein LOC683855	LOC681037 /// LOC683855	0.6	0.003208395	0.022107952
1376651_at	Transcribed locus	---	0.7	0.003222833	0.022179565
1368292_at	dynamin 1	Dnm1	0.5	0.003226416	0.022185643
1393230_s_at	similar to KIAA0564 protein (predicted)	RGD1308772_predicted	0.6	0.003227763	0.022185643
1398662_at	Transcribed locus	---	0.7	0.003237836	0.022245585
1388889_at	putative aminopeptidase Fxna	Fxna	0.7	0.003240805	0.022256683
1378309_at	ubiquitin specific protease 20 (predicted)	Usp20_predicted	0.7	0.003249865	0.022282817
1370672_a_at	dynamin 3	Dnm3	0.4	0.003251241	0.022282817
1389167_at	mitogen-activated protein kinase associated protein 1	Mapkap1	0.6	0.003251382	0.022282817
1388532_at	similar to hypothetical protein	RGD1310571	0.7	0.003252739	0.022282817
1383726_at	hypothetical LOC294715	RGD1309360	0.5	0.003254953	0.022288705

1377729_at	elongation of very long chain fatty acids (FEN1/Elo2, SUR4/Elo3, yeast)-like 4 (predicted)	Elovl4_predicted	0.7	0.003269031	0.022357179
1383701_at	---	---	0.5	0.00327655	0.022371396
1368123_at	insulin-like growth factor 1 receptor	Igf1r	0.5	0.003283141	0.022407096
1385170_at	ollistatin-like 5 (predicted)	Fstl5_predicted	0.7	0.003287451	0.022427207
1376310_at	---	---	0.5	0.003296746	0.022481293
1395474_at	Transcribed locus	---	0.5	0.003300597	0.02248255
1373794_at	EGL nine homolog 2 (C. elegans)	Egln2	0.5	0.003301031	0.02248255
1375127_at	---	---	0.5	0.003304279	0.022490272
1380128_at	Leucine zipper-EF-hand containing transmembrane protein 2	Letm2	0.7	0.003304899	0.022490272
1367900_at	glycogenin 1	Gyg1	0.7	0.00330743	0.022498186
1385460_at	WD repeat and SOCS box-containing 2	Wsb2	0.6	0.003321254	0.022564221
1377267_at	melanoma antigen, family E, 1 (predicted)	Magee1_predicted	0.6	0.003325615	0.022575204
1388705_at	similar to selenoprotein SelM (predicted)	RGD1565037_predicted	0.4	0.003328618	0.02257564
1398785_at	multiple endocrine neoplasia 1	Men1	0.6	0.003329797	0.02257564
1377112_at	cytidine deaminase (predicted)	Cda_predicted	0.7	0.003334367	0.022597308
1388831_at	Solute carrier family 9 (sodium/hydrogen exchanger), isoform 3 regulator 2	Slc9a3r2	0.6	0.003337534	0.02260825
1377408_at	phospholipase A2, group VI	Pla2g6	0.5	0.003349847	0.022655544
1378524_at	ring finger protein (C3HC4 type) 19 (predicted)	Rnf19_predicted	0.6	0.003353122	0.022668374
1392467_at	inositol (myo)-1(or 4)-monophosphatase 2	Impa2	0.5	0.003364992	0.02271539
1376215_at	similar to hypothetical protein 9630025C22 (predicted)	RGD1559945_predicted	0.5	0.003367486	0.02271539
1374582_at	potassium channel tetramerisation domain containing 9 (predicted)	Kctd9_predicted	0.5	0.003369152	0.02271539
1387407_at	nucleosome assembly protein 1-like 3	Nap1l3	0.6	0.00337911	0.022721768
1383642_at	---	---	0.5	0.003387881	0.022758525
1379354_at	exostoses (multiple)-like 2	Extl2	0.6	0.003388596	0.022758525
1373848_at	similar to RIKEN cDNA 5730449L18 (predicted)	RGD1308584_predicted	0.6	0.003391831	0.022761663
1369065_a_at	ATPase, Ca++ transporting, cardiac muscle, slow twitch 2	Atp2a2	0.5	0.00340647	0.02285058
1390707_at	regulator of G-protein signalling 10	Rgs10	0.5	0.003417826	0.022908475
1369674_at	purinergic receptor P2X, ligand-gated ion channel, 5	P2rx5	0.5	0.003435435	0.023016353
1394290_at	Transcribed locus	---	0.4	0.003477041	0.02321038
1394258_at	---	---	0.6	0.003484879	0.023253264
1376842_at	Transcribed locus	---	0.5	0.003498449	0.023305989
1389307_at	similar to Amyloid beta (A4) precursor-like protein 1 (predicted)	RGD1561211_predicted	0.5	0.003502317	0.023320401
1373170_at	G protein pathway suppressor 2 (predicted)	Gps2_predicted	0.8	0.003503448	0.023320401
1375028_at	Transcribed locus	---	0.6	0.00350517	0.023322423
1381974_at	BTB (POZ) domain containing 3 (predicted)	Btbd3_predicted	0.7	0.003512932	0.023364617
1381178_at	Transcribed locus	---	0.7	0.003522086	0.023416034
1371211_a_at	neuregulin 1	Nrg1	0.3	0.003538914	0.023489935
1371920_at	polymerase (DNA-directed), delta interacting protein 2 (predicted)	Poldip2_predicted	0.6	0.003544687	0.023518762
1382807_at	similar to hypothetical protein	RGD1310450	0.6	0.003555006	0.023577714
1384834_at	cordon-bleu (predicted)	Cobl_predicted	0.7	0.003563064	0.023586925
1397015_at	Transcribed locus	---	0.5	0.0035639	0.023586925
1398471_at	component of oligomeric golgi complex 6	Cog6	0.7	0.003566531	0.023587577
1382013_at	---	---	0.5	0.003573745	0.023620688
1378026_at	Transcribed locus	---	0.5	0.003575244	0.023620688
1383843_at	similar to homolog of Human holocarboxylase synthetase gene HLCS (predicted)	RGD1565360_predicted	0.4	0.003579766	0.023637087
1377601_at	similar to C21orf70 protein	RGD1311257	0.7	0.003591541	0.023705321
1395921_at	similar to Ubiquitin carboxyl-terminal hydrolase 35 (Ubiquitin thiolesterase 35) (predicted)	RGD1565984_predicted	0.7	0.003594524	0.023705986
1384745_at	piwi like homolog 2 (Drosophila) (predicted)	Piwil2_predicted	0.4	0.00360102	0.023729795
1383014_at	Transcribed locus	---	0.4	0.003613314	0.02377472
1386282_x_at	SNF related kinase /// hypothetical gene supported by NM_138833	LOC367880 /// Snrk	0.7	0.003631816	0.023865804
1396172_at	CDC16 cell division cycle 16 homolog (S. cerevisiae)	Cdc16	0.5	0.003634625	0.023874438

1382866_at	similar to Putative protein 15E1.2 (predicted)	RGD1309698_predicted	0.6	0.003648471	0.023920789
1372553_at	similar to RIKEN cDNA 2610301B20; EST AI428449	MGC94199	0.6	0.003648816	0.023920789
1392422_at	asparagine-linked glycosylation 9 homolog (yeast, alpha 1,2 mannosyltransferase) (predicted)	Alg9_predicted	0.7	0.003653009	0.023920789
1382386_at	similar to Lmbr1 protein (predicted)	RGD1563910_predicted	0.5	0.003653272	0.023920789
1382319_at	G protein-coupled receptor 68 (predicted)	Gpr68_predicted	0.7	0.003665214	0.02397989
1380082_at	Transcribed locus	---	0.7	0.003683854	0.024073115
1374663_at	Transcribed locus	---	0.7	0.003698856	0.024152348
1368528_at	MIC2 like 1	Mic2l1	0.7	0.003698916	0.024152348
1372548_at	Cryptochrome 2 (photolyase-like)	Cry2	0.4	0.003701105	0.024157052
1391802_at	Transcribed locus	---	0.7	0.00370343	0.024162638
1387898_at	heat shock protein, alpha-crystallin-related, B6	Hspb6	0.6	0.003706822	0.024175181
1387023_at	glutathione S-transferase, mu type 3	Gstm3	0.4	0.003708687	0.024177761
1370200_at	glutamate dehydrogenase 1	Glud1	0.7	0.003714383	0.024190595
1390899_at	similar to leucine-rich repeat transmembrane neuronal 1	LOC679668 /// LOC684054	0.6	0.003716539	0.024190595
1372532_at	phosphatidylinositol transfer protein, membrane-associated 2 (predicted)	Pitpnm2_predicted	0.6	0.00372766	0.024253384
1371935_at	transmembrane protein 9 (predicted)	Tmem9_predicted	0.5	0.003743521	0.024317716
1373971_at	1-acylglycerol-3-phosphate O-acyltransferase 7 (lysophosphatidic acid acyltransferase, eta) (predicted)	Agpat7_predicted	0.7	0.003765324	0.024411528
1390547_at	Similar to Transmembrane anchor protein 1	LOC690599	0.7	0.003775078	0.024465126
1388738_at	similar to mKIAA0534 protein (predicted)	RGD1563485_predicted	0.4	0.003802361	0.02462253
1381749_at	Transcribed locus	---	0.7	0.003805459	0.024632899
1368506_at	regulator of G-protein signaling 4	Rgs4	0.3	0.003808426	0.024642403
1386088_at	similar to RIKEN cDNA 1200007B05 gene	RGD1305072	0.4	0.003816514	0.024665628
1385007_at	zinc finger, CCHC domain containing 9	Zcchc9	0.6	0.003823152	0.024679436
1396408_at	similar to RAS-related C3 botulinum substrate 3	LOC688319	0.7	0.003831112	0.02472112
1398326_at	similar to Nur77 downstream protein 2	MGC105647	0.5	0.003836964	0.024749169
1377673_at	Transcribed locus	---	0.8	0.00384416	0.024773335
1388891_at	Transcribed locus	---	0.3	0.003852439	0.024788306
1383558_at	similar to MJ0495-like protein SelB (predicted)	RGD1560552_predicted	0.5	0.003854649	0.024788306
1372524_at	Transcribed locus	---	0.7	0.003856736	0.024788306
1375575_at	calcium/calmodulin-dependent protein kinase II, beta	Camk2b	0.7	0.003859056	0.024788306
1369309_a_at	tachykinin 1	Tac1	0.5	0.003864057	0.02479751
1394431_at	cytidine and dCMP deaminase domain containing 1	Cdadc1	0.7	0.003870589	0.024817968
1388760_at	solute carrier family 35, member B4 (predicted)	Slc35b4_predicted	0.6	0.003870684	0.024817968
1393623_at	Coilin	Coil	0.6	0.003873411	0.024817968
1384879_at	tRNA phosphotransferase 1 (predicted)	Trpt1_predicted	0.7	0.003873581	0.024817968
1380852_at	hypothetical LOC294883	RGD1305844	0.7	0.003875052	0.024817968
1377147_at	TBC1 domain family, member 7 (predicted)	Tbc1d7_predicted	0.7	0.003884778	0.024843244
1376176_at	similar to Microtubule-associated serine/threonine-protein kinase 3	LOC684053 /// LOC688540	0.7	0.003894793	0.024897611
1386955_at	septin 5	Sep-05	0.5	0.003903322	0.024925577
1396212_at	Transmembrane and tetratricopeptide repeat containing 2 (predicted)	Tmtc2_predicted	0.7	0.003913828	0.024970767
1382423_at	Transcribed locus	---	0.7	0.003928842	0.025037426
1390104_at	Transcribed locus	---	0.7	0.003935075	0.025067435
1368669_at	uncoupling protein 2 (mitochondrial, proton carrier)	Ucp2	0.4	0.00393798	0.025073969
1373248_at	Similar to hypothetical protein FLJ20674 (predicted)	RGD1565800_predicted	0.7	0.00394147	0.025074846
1370404_at	cystatin TE-1	LOC266776	0.3	0.003942337	0.025074846
1385950_at	hypothetical protein LOC500102	LOC500102	0.5	0.00395531	0.025128213
1396190_x_at	Transcribed locus	---	0.6	0.003971692	0.025206829

1370185_at	contactin associated protein 1	Cntnap1	0.6	0.003972282	0.025206829
1392893_a_at	Transcribed locus	---	0.7	0.003993979	0.025305455
1372594_at	SUMO/sentrin specific protease 5 (predicted) /// similar to SUMO/sentrin specific protease 5	LOC686286 /// Senp5_predicted	0.7	0.00399872	0.025322133
1383174_at	Transcribed locus	---	0.7	0.00399969	0.025322133
1387941_s_at	phospholipase A2, group VI	Pla2g6	0.6	0.004002051	0.025327331
1383722_at	proline synthetase co-transcribed (predicted)	Prosc_predicted	0.7	0.004030066	0.025426364
1387265_at	diacylglycerol kinase, gamma	Dgkg	0.7	0.004047743	0.025498764
1374845_at	Transcribed locus	---	0.5	0.004060422	0.025568844
1368286_at	solute carrier family 2, (facilitated glucose transporter) member 8	Slc2a8	0.6	0.004098344	0.025768183
1370544_at	echinoderm microtubule associated protein like 2	Eml2	0.5	0.004120552	0.025888019
1391598_at	Transcribed locus	---	0.4	0.00413292	0.025955812
1378854_at	5-methyltetrahydrofolate-homocysteine methyltransferase reductase	Mtrr	0.8	0.004135388	0.025960813
1374107_at	elaC homolog 2 (E. coli)	Elac2	0.6	0.004136873	0.025960813
1375173_at	RDCR-0918-3 protein	LOC246120	0.7	0.004146074	0.026008628
1390097_at	TSPY-like 4	Tspyl4	0.5	0.004165408	0.026100051
1372583_at	Transcribed locus	---	0.7	0.004167598	0.026103829
1377404_at	stanniocalcin 1	Stc1	0.6	0.004172435	0.026113384
1380585_at	Transcribed locus	---	0.5	0.004186412	0.026170965
1391153_at	Transcribed locus	---	0.6	0.004204091	0.026242464
1385046_at	hypothetical protein LOC681096 /// hypothetical protein LOC682630	LOC681096 /// LOC682630	0.6	0.004211897	0.026253253
1375940_a_at	---	---	0.4	0.004212204	0.026253253
1383799_at	---	---	0.6	0.004219777	0.026280537
1379536_at	pleckstrin homology domain containing, family A member 6 (predicted)	Plekha6_predicted	0.6	0.004229644	0.026332018
1398342_at	similar to Fus1 protein	LOC501052	0.4	0.004247307	0.026431972
1371855_at	---	---	0.5	0.004264124	0.026516565
1374243_at	Transcribed locus	---	0.7	0.004272266	0.026537094
1375972_at	similar to membrane protein expressed in epithelial-like lung adenocarcinoma (predicted)	RGD1307493_predicted	0.6	0.004274968	0.026543857
1388309_at	high mobility group AT-hook 1	Hmga1	0.4	0.004284167	0.026580908
1377857_at	Transcribed locus	---	0.6	0.004314211	0.026737054
1374553_at	Transcribed locus	---	0.6	0.004319527	0.026759915
1370535_at	myelin transcription factor 1-like	Myt1l	0.7	0.00432235	0.026767326
1371473_at	cleft lip and palate associated transmembrane protein 1 (predicted)	Clptm1_predicted	0.5	0.004328149	0.026793149
1376520_at	Transcribed locus	---	0.7	0.004331775	0.026805506
1389736_at	Transcribed locus	---	0.5	0.004341163	0.026846393
1372213_at	similar to hypothetical protein MGC6835	LOC500300	0.6	0.004348995	0.026861534
1399038_at	ubiquitin specific protease 19	Usp19	0.6	0.004386058	0.027019432
1376232_at	Transcribed locus	---	0.6	0.004393896	0.02704502
1371597_at	ring finger protein 187 (predicted)	Rnf187_predicted	0.6	0.004396891	0.02704502
1387435_at	ST8 alpha-N-acetyl-neuraminide alpha-2,8-sialyltransferase 3	St8sia3	0.7	0.004401469	0.027053576
1373965_at	similar to hypothetical protein BC013949 (predicted)	RGD1310931_predicted	0.5	0.004420765	0.027162023
1377865_at	Transcribed locus	---	0.7	0.004433136	0.027227863
1390540_at	syntaphilin (predicted)	Snph_predicted	0.7	0.004447572	0.027296144
1389063_at	exportin 6	Xpo6	0.7	0.004451269	0.027300819
1386338_at	Ubiquitin specific protease 22 (predicted)	Usp22_predicted	0.6	0.004466366	0.027350255
1372879_at	AKT1 substrate 1 (proline-rich) (predicted)	Akt1s1_predicted	0.3	0.004470775	0.027360564
1376919_at	Similar to expressed sequence AW212394 (predicted)	RGD1562317_predicted	0.7	0.004474938	0.027362
1383178_at	F-box protein 28 (predicted)	Fbxo28_predicted	0.6	0.004488375	0.027410884
1367604_at	cysteine-rich protein 2	Crip2	0.6	0.004489186	0.027410884
1387171_at	glutamate receptor, ionotropic, AMPA2	Gria2	0.5	0.004490392	0.027410884
1393636_at	Transcribed locus	---	0.6	0.004493667	0.027415368
1390336_at	similar to Protein C21orf63 homolog precursor (predicted)	RGD1307569_predicted	0.6	0.004506584	0.027461108
1390791_at	growth arrest-specific 2 like 1 (predicted)	Gas2l1_predicted	0.5	0.004512345	0.027478362
1375954_at	S100 calcium binding protein A13 (predicted)	S100a13_predicted	0.6	0.004539131	0.027590404
1372551_at	Fas-activated serine/threonine kinase	Fastk	0.5	0.004546417	0.02761428
1370374_at	STEAP family member 3	Steap3	0.6	0.004550392	0.02761779

1372276_at	Transcribed locus	---	0.7	0.004552032	0.02761779
1374999_at	Transcribed locus	---	0.8	0.004556506	0.02762455
1370933_at	myosin IE	Myo1e	0.5	0.004560884	0.027640908
1384460_at	Transcribed locus	---	0.5	0.004593374	0.027807075
1398606_at	golgi phosphoprotein 4	Golph4	0.5	0.004599545	0.027834184
1380675_s_at	carbonic anhydrase 11	Car11	0.6	0.004611914	0.027855173
1371068_at	protocadherin alpha 13	Pcdha13	0.7	0.004613174	0.027855173
1377424_at	Transcribed locus	---	0.6	0.00461791	0.027873532
1386927_at	carnitine palmitoyltransferase 2	Cpt2	0.6	0.00463072	0.027914124
1387505_at	guanine nucleotide binding protein, alpha inhibiting 1	Gnai1	0.4	0.004654532	0.027992425
1375272_at	Transcribed locus	---	0.8	0.004655635	0.027992425
1378805_at	Transcribed locus	---	0.4	0.004656328	0.027992425
1383280_at	Transcribed locus	---	0.5	0.004662418	0.028018796
1395541_at	Transcribed locus	---	0.7	0.004665032	0.028023421
1391146_at	cadherin 11	Cdh11	0.6	0.004668298	0.028023421
1383258_at	similar to hypothetical protein FLJ14146	RGD1310587	0.7	0.00468298	0.028085946
1384696_at	similar to RIKEN cDNA 1700001E04	MGC116197	0.3	0.004690343	0.028085946
1384936_at	---	---	0.6	0.004690667	0.028085946
1397764_at	potassium channel tetramerisation domain containing 5 (predicted)	Kctd5_predicted	0.6	0.004710506	0.028194467
1373069_at	mitochondrial ribosomal protein S30 (predicted)	Mrps30_predicted	0.5	0.00471746	0.028225819
1368117_at	gephyrin	Gphn	0.7	0.00472271	0.028231717
1373542_at	sphingosine kinase 2	Sphk2	0.7	0.004744374	0.028283969
1371721_at	hypothetical LOC500532	LOC500532	0.6	0.004754247	0.028322297
1368208_at	camello-like 1	Cml1	0.6	0.004758231	0.028335764
1368646_at	mitogen-activated protein kinase 9	Mapk9	0.4	0.004761997	0.028341259
1389252_at	Zinc finger protein 238	Zfp238	0.5	0.004766946	0.028346613
1387498_a_at	Fibroblast growth factor receptor 1	Fgfr1	0.4	0.004778596	0.028374852
1383664_a_at	similar to chromosome 11 open reading frame2 (predicted)	RGD1560544_predicted	0.5	0.004803288	0.028459827
1397442_at	---	---	0.7	0.004807817	0.028476401
1369024_at	rabaptin, RAB GTPase binding effector protein 2	Rabep2	0.7	0.004828013	0.028575441
1379736_at	solute carrier family 43, member 2 (predicted)	Slc43a2_predicted	0.7	0.004832082	0.028589239
1376038_at	testis expressed gene 2	Tex2	0.7	0.004847607	0.028656687
1390958_at	Transcribed locus	---	0.4	0.004848709	0.028656687
1369816_at	RAB3A, member RAS oncogene family	Rab3a	0.5	0.004850773	0.02865859
1396058_at	Transcribed locus	---	0.5	0.004853128	0.028662208
1382227_at	Transcribed locus	---	0.7	0.004858456	0.028683377
1383492_at	similar to Homeobox protein Hox-D9 (Hox-4.4) (Hox-5.2)	LOC682957	0.5	0.004873449	0.028751251
1395286_at	Transcribed locus	---	0.5	0.004878362	0.02876802
1372235_at	---	---	0.7	0.004886354	0.028775782
1375223_at	---	---	0.5	0.004907922	0.028865082
1389532_at	Nebulette (predicted)	Nebl_predicted	0.6	0.004908538	0.028865082
1387030_at	ATP-binding cassette, sub-family C (CFTR/MRP), member 5	Abcc5	0.6	0.00492099	0.028907302
1383483_at	Transcribed locus	---	0.6	0.004923408	0.02891118
1384608_at	Optic atrophy 1 homolog (human)	Opa1	0.4	0.00493267	0.02895523
1373861_at	Nedd4 family interacting protein 2 (predicted)	Ndfip2_predicted	0.7	0.004943261	0.028996702
1371985_a_at	HLA-B associated transcript 5	Bat5	0.8	0.004979511	0.029163972
1393309_at	zinc finger protein ZFP	LOC503192	0.7	0.004981983	0.029163972
1390455_at	abhydrolase domain containing 2 (predicted)	Abhd2_predicted	0.7	0.00498454	0.029166033
1389567_at	SREBP cleavage activating protein (predicted)	Scap_predicted	0.6	0.005020989	0.029309365
1377522_a_at	similar to KIAA1086 protein (predicted)	RGD1304737_predicted	0.5	0.00502151	0.029309365
1375851_at	kelch-like 8 (Drosophila) (predicted)	Klhl8_predicted	0.7	0.005031235	0.02935571
1394481_at	ubiquitin-conjugating enzyme E2Z (putative)	Ube2z	0.6	0.005039146	0.029391446
1385981_at	plasticity related gene 1	Prg1	0.7	0.005060436	0.029479248
1368964_at	leucine rich repeat protein 3, neuronal	Lrrn3	0.6	0.005072206	0.029496544
1391661_at	Transcribed locus	---	0.6	0.005073433	0.029496544
1384138_at	Transcribed locus	---	0.5	0.005077918	0.029496544
1384839_at	Transcribed locus	---	0.7	0.005100409	0.029591365
1389414_at	Transcribed locus, weakly similar to XP_001379341.1 similar to env polyprotein [Monodelphis domestica]	---	0.6	0.005103119	0.029596649

1388976_at	similar to BolA domain-containing protein like (11.4 kD) (1P25) (predicted)	RGD1305975_predicted	0.6	0.005128292	0.029721685
1398604_at	oxysterol binding protein 2 (predicted)	Osbp2_predicted	0.7	0.005165181	0.029880447
1383613_at	Transcribed locus	---	0.6	0.005168402	0.029880447
1380833_at	glycosylphosphatidylinositol specific phospholipase D1	Gpld1	0.7	0.005193272	0.030003141
1371723_at	Ras-related GTP binding C (predicted)	Rragc_predicted	0.5	0.005201801	0.030031315
1376490_at	Transcribed locus	---	0.7	0.005204114	0.030034126
1370978_at	secretory carrier membrane protein 1	Scamp1	0.7	0.005223625	0.030073361
1398272_at	beta-1,4-N-acetyl-galactosaminyl transferase 1	B4galnt1	0.6	0.005238298	0.030118075
1370639_at	PR domain containing 2, with ZNF domain	Prdm2	0.7	0.005239828	0.030118075
1378857_at	similar to transcription elongation factor A (SII)-like 5	LOC678833	0.5	0.005240633	0.030118075
1375081_at	5',3'-nucleotidase, mitochondrial (predicted)	Nt5m_predicted	0.5	0.00526839	0.030265409
1385972_at	kelch repeat and BTB (POZ) domain containing 3 (predicted)	Kbtbd3_predicted	0.7	0.005272654	0.030265409
1378105_at	Transcribed locus	---	0.4	0.005292591	0.030338045
1383560_at	Similar to RIKEN cDNA 2610528A15	RGD1305984	0.7	0.005295278	0.030338045
1382498_x_at	ganglioside-induced differentiation-associated protein 1-like 1 (predicted)	Gdap1l1_predicted	0.7	0.005303571	0.03036636
1378003_at	similar to T-cell activation leucine repeat-rich protein (predicted)	RGD1563429_predicted	0.7	0.005306586	0.03036636
1389195_at	DNA fragmentation factor, alpha subunit	Dffa	0.7	0.005309682	0.03036636
1373149_at	similar to yippee-like 3 (predicted)	RGD1564579_predicted	0.4	0.005313591	0.030373074
1387799_at	FXYD domain-containing ion transport regulator 2	Fxyd2	0.6	0.005316665	0.03037461
1385322_at	Transcribed locus	---	0.7	0.005320711	0.030387172
1389541_at	malignant fibrous histiocytoma amplified sequence 1 (predicted)	Mfhas1_predicted	0.8	0.005345589	0.030486908
1383032_at	Transcribed locus	---	0.7	0.005362438	0.030559333
1396215_at	Similar to RIKEN cDNA 2610022G08	LOC502782	0.6	0.005394161	0.030700055
1384194_at	Frizzled homolog 7 (Drosophila) (predicted)	Fzd7_predicted	0.7	0.005408418	0.03075991
1371600_at	protein kinase inhibitor, gamma	Pkig	0.7	0.005418139	0.030797084
1370472_a_at	potassium large conductance calcium-activated channel, subfamily M, alpha member 1	Kcnma1	0.7	0.005421351	0.030801515
1390763_at	ephrin A3	Efna3	0.7	0.005434882	0.03085707
1373613_at	similar to RIKEN cDNA 4930570C03	LOC300191	0.6	0.005442649	0.030869203
1378853_at	similar to FLJ22405 protein (predicted)	RGD1304842_predicted	0.7	0.005476103	0.031048238
1373162_at	similar to transmembrane protein 41a	LOC681708	0.5	0.005486763	0.031087246
1379757_at	Transcribed locus	---	0.6	0.005494118	0.031118198
1368822_at	follistatin-like 1	Fstl1	0.5	0.00550179	0.031149284
1386914_at	guanosine monophosphate reductase	Gmpr	0.5	0.005510174	0.03116621
1371606_at	WD and tetratricopeptide repeats 1 (predicted)	Wdtc1_predicted	0.5	0.005514979	0.031182665
1389549_at	proline synthetase co-transcribed (predicted)	Prosc_predicted	0.7	0.00551816	0.031185568
1391879_at	Transcribed locus	---	0.4	0.005519284	0.031185568
1372206_at	similar to chromosome 14 open reading frame 9	RGD1307475	0.5	0.005529412	0.031221342
1373582_at	similar to CLIP-170-related protein (predicted)	RGD1306245_predicted	0.5	0.005538086	0.031247203
1394815_at	---	---	0.6	0.005539691	0.031247203
1383482_at	Transcribed locus	---	0.4	0.005542903	0.031248323
1399166_a_at	similar to B-cell CLL/lymphoma 7B	LOC368001	0.7	0.005558116	0.031297467
1397756_at	similar to BC068281 protein (predicted)	RGD1563701_predicted	0.6	0.005561508	0.031301989
1389725_at	transmembrane 7 superfamily member 2	Tm7sf2	0.5	0.005562726	0.031301989
1385314_at	Transcribed locus	---	0.5	0.005580756	0.031338792
1375137_at	actin related protein 2/3 complex, subunit 2 (predicted)	Arpc2_predicted	0.3	0.005600905	0.031398667
1369922_at	RDCR-0918-3 protein	LOC246120	0.5	0.005607262	0.031409251
1368254_a_at	sphingosine kinase 1	Sphk1	0.6	0.005608522	0.031409251
1391274_at	Transcribed locus	---	0.6	0.005620783	0.031462445
1380401_at	Transcribed locus	---	0.4	0.005622242	0.031462445
1385512_at	similar to APG4-D protein	LOC686505	0.7	0.005626249	0.031465674
1374811_at	---	---	0.5	0.005646187	0.031519981
1371029_at	polycystic kidney disease 1 homolog	Pkd1	0.6	0.005646202	0.031519981

1370555_at	voltage gated channel like 1	Vgcnl1	0.7	0.005649406	0.031520159
1368600_at	solute carrier family 26 (sulfate transporter), member 1	Slc26a1	0.7	0.005657266	0.031542617
1388933_at	Transcribed locus	---	0.4	0.005669471	0.031584079
1372176_at	protein kinase C, alpha	Prkca	0.7	0.005670502	0.031584079
1398890_at	similar to 2310044H10Rik protein	MGC93975	0.5	0.005672384	0.031584079
1369446_at	cryptochrome 2 (photolyase-like)	Cry2	0.8	0.005676979	0.031595111
1381557_at	guanine nucleotide binding protein, alpha 14	Gna14	0.6	0.00568063	0.031597902
1367812_at	spectrin beta 3	Spnb3	0.7	0.00568475	0.031610133
1373459_at	serine/threonine kinase 11 interacting protein (predicted)	Stk11ip_predicted	0.6	0.005710426	0.031720729
1370132_at	FK506 binding protein 1b	Fkbp1b	0.4	0.005714634	0.031733387
1368313_a_at	transient receptor potential cation channel, subfamily V, member 1	Trpv1	0.3	0.005738194	0.031831979
1388490_at	Brca1 associated protein 1 (predicted)	Bap1_predicted	0.7	0.005753336	0.031884731
1379465_at	hypothetical LOC311134	LOC311134	0.6	0.005754579	0.031884731
1395547_at	Guanine nucleotide binding protein, alpha q polypeptide	Gnaq	0.5	0.005755458	0.031884731
1367549_a_at	adaptor-related protein complex 3, delta 1 subunit	Ap3d1	0.7	0.00577415	0.031955993
1373952_at	protein kinase, AMP-activated, gamma 2 non-catalytic subunit	Prkag2	0.6	0.005786439	0.032008559
1388030_a_at	gamma-aminobutyric acid (GABA) B receptor 1	Gabbr1	0.5	0.005787679	0.032008559
1378386_at	solute carrier family 2 (facilitated glucose transporter), member 6 (predicted)	Slc2a6_predicted	0.5	0.005811266	0.032107391
1371979_at	sterol regulatory element binding factor 2 (predicted)	Srebf2	0.5	0.005823342	0.032141723
1374480_at	dishevelled associated activator of morphogenesis 1 (predicted)	Daam1_predicted	0.7	0.005855058	0.0322951
1392941_at	Transcribed locus	---	0.6	0.005866987	0.032344792
1392607_at	Similar to erythrocyte membrane protein band 4.1 (elliptocytosis 1, RH-linked) isoform 1 (predicted)	RGD1564762_predicted	0.7	0.005868	0.032344792
1385620_at	heat shock 105kDa/110kDa protein 1	Hsph1	0.3	0.005883882	0.032421469
1388968_at	Transcribed locus	---	0.5	0.005898131	0.032472269
1383134_at	similar to hypothetical protein FLJ12118 (predicted)	RGD1311612_predicted	0.6	0.005911754	0.032498842
1369417_a_at	opioid binding protein/cell adhesion molecule-like	Opcml	0.4	0.005927406	0.032550322
1387147_at	RAB3C, member RAS oncogene family	Rab3c	0.7	0.005929035	0.032550322
1368386_at	growth factor receptor bound protein 2	Grb2	0.7	0.005947137	0.032638807
1369733_at	catenin (cadherin associated protein), beta 1	Ctnnb1	0.5	0.005968245	0.032743728
1382435_at	fizzy/cell division cycle 20 related 1 (Drosophila) (predicted)	Fzr1_predicted	0.6	0.005971405	0.032750137
1374781_at	Transcribed locus	---	0.7	0.005977572	0.032773034
1394286_at	ganglioside-induced differentiation-associated protein 1-like 1 (predicted)	Gdap1l1_predicted	0.6	0.005983675	0.032784637
1372617_at	similar to BTB (PO)Z domain containing 2 (predicted)	RGD1566094_predicted	0.6	0.005987046	0.032792186
1393890_at	similar to protein phosphatase 1, regulatory (inhibitor) subunit 1C (predicted)	RGD1562095_predicted	0.7	0.005990622	0.032800851
1376139_at	plexin A3	Plxna3	0.7	0.006006434	0.032832778
1374908_at	Similar to hypothetical protein B230399E16 (predicted)	RGD1559694_predicted	0.6	0.006018889	0.03288993
1377599_at	---	---	0.6	0.006041941	0.032961143
1376653_at	putative homeodomain transcription factor 1	Phtf1	0.7	0.006058213	0.033006125
1370857_at	smooth muscle alpha-actin	Acta2	0.7	0.006069791	0.033058251
1392632_at	similar to mKIAA0227 protein (predicted)	RGD1565985_predicted	0.4	0.006077512	0.033089348
1370060_at	solute carrier family 25 (mitochondrial carrier; oxoglutarate carrier), member 11	Slc25a11	0.6	0.006085883	0.033123959
1388406_at	similar to RIKEN cDNA 1200013P24	RGD1308915	0.6	0.006106313	0.033202194
1389041_at	Vac14 homolog (S. cerevisiae)	Vac14	0.6	0.006117578	0.033252451
1373494_at	breakpoint cluster region (predicted)	Bcr_predicted	0.7	0.006120426	0.033256943
1373070_at	Transcribed locus	---	0.6	0.006125583	0.033273974
1369924_at	synuclein, beta	Sncb	0.7	0.006147416	0.033360263
1376817_at	Transcribed locus	---	0.7	0.006165104	0.033411411
1386971_at	protein phosphatase 1, regulatory subunit 10	Ppp1r10	0.7	0.006261178	0.033820643

1389804_at	Transcribed locus	---	0.7	0.006272992	0.033861157
1382063_at	galactosidase, alpha	Gla	0.6	0.006274854	0.033861157
1377011_at	similar to hypothetical protein CG003 (predicted)	RGD1307034_predicted	0.7	0.006277398	0.033863775
1372358_at	similar to IQ motif and Sec7 domain 1	LOC686590	0.7	0.006283957	0.033888042
1380760_at	Transcribed locus	---	0.7	0.006309363	0.033980487
1388493_at	similar to Expressed sequence AW146242 (predicted)	RGD1306494_predicted	0.7	0.006325248	0.03405325
1398300_at	ATPase, Na+/K+ transporting, beta 3 polypeptide	Atp1b3	0.6	0.006331255	0.034063557
1393084_at	Transcribed locus	---	0.6	0.006339458	0.034065854
1392836_at	Upstream binding protein 1 (predicted)	Ubp1_predicted	0.7	0.006339712	0.034065854
1372998_at	---	---	0.7	0.006373666	0.034203606
1377181_at	Transcribed locus	---	0.8	0.006379192	0.034210938
1393115_at	similar to ubiquitin specific protease 46 (predicted)	RGD1564808_predicted	0.7	0.006386734	0.034240224
1390474_at	similar to RIKEN cDNA 9330161F08 (predicted)	RGD1564315_predicted	0.6	0.006423001	0.034401015
1386052_at	Transcribed locus	---	0.5	0.006430429	0.03442959
1376821_at	Transcribed locus	---	0.7	0.006436325	0.03444994
1372040_at	calmodulin binding transcription activator 2 (predicted)	Camta2_predicted	0.6	0.006439706	0.034456828
1395226_at	similar to RIKEN cDNA 9130023F12 gene (predicted)	RGD1311920_predicted	0.7	0.006452938	0.034493965
1396059_at	UBX domain containing 2	Ubxd2	0.5	0.006458306	0.034500231
1387809_at	mitogen-activated protein kinase kinase 6	Map2k6	0.6	0.006467242	0.0345089
1383831_at	glutaminyl-tRNA synthase (glutamine-hydrolyzing)-like 1	Qrsl1	0.7	0.006468321	0.0345089
1370058_at	neurofilament, light polypeptide	Nefl	0.6	0.006480315	0.034516914
1373483_at	ankyrin repeat domain 47 (predicted)	Ankrd47_predicted	0.7	0.006510593	0.034645428
1387652_at	insulin degrading enzyme	Ide	0.7	0.006510762	0.034645428
1383933_at	Similar to KIAA0564 protein (predicted)	RGD1308772_predicted	0.6	0.006527478	0.034691842
1385025_a_at	G protein-coupled receptor 137B (predicted)	Gpr137b_predicted	0.5	0.006549688	0.03472897
1389471_at	translocase of outer mitochondrial membrane 34 (predicted)	Tomm34_predicted	0.7	0.00657122	0.034792246
1392932_at	leukocyte receptor cluster (LRC) member 8	Leng8	0.5	0.006572198	0.034792246
1372074_at	nudix (nucleotide diphosphate linked moiety X)-type motif 3	Nudt3	0.5	0.006578208	0.034801664
1368391_at	solute carrier family 7 (cationic amino acid transporter, y+ system), member 1	Slc7a1	0.7	0.006593216	0.034858634
1382479_at	leucine rich repeat containing 4C (predicted)	Lrrc4c_predicted	0.7	0.006619708	0.034931332
1373118_at	similar to Protein KIAA1543	LOC682941 /// LOC689074	0.6	0.006639973	0.035027031
1380741_at	Transcribed locus	---	0.6	0.006642636	0.03502984
1389491_at	Transcribed locus	---	0.6	0.006657121	0.03508373
1382280_at	ubiquinol cytochrome c reductase core protein 2	Uqcrc2	0.7	0.006677168	0.035155588
1387367_at	golgi apparatus protein 1	Glg1	0.5	0.006714403	0.035307499
1386259_a_at	similar to RIKEN cDNA 1110008J03 (predicted)	RGD1306772_predicted	0.7	0.006714607	0.035307499
1367823_at	tissue inhibitor of metalloproteinase 2	Timp2	0.6	0.006725763	0.035354859
1393554_at	similar to hypothetical protein DKFZp761E198 (predicted)	RGD1562657_predicted	0.8	0.006743692	0.035426461
1398942_at	CDNA clone MGC:95041 IMAGE:7123253	---	0.6	0.006763619	0.035485809
1367648_at	insulin-like growth factor binding protein 2	Igfbp2	0.5	0.006784947	0.035559091
1371343_at	signal recognition particle receptor ('docking protein')	Srpr	0.8	0.006786235	0.035559091
1382537_at	Ras-related GTP binding C (predicted)	Rragc_predicted	0.3	0.006794807	0.035579749
1371254_at	ubiquinol-cytochrome c reductase, Rieske iron-sulfur polypeptide 1	Uqcrfs1	0.6	0.006796667	0.035579749
1369061_at	glutathione reductase	Gsr	0.5	0.006807253	0.035605435
1389388_at	Transcribed locus	---	0.6	0.00682997	0.035631654
1372966_at	hypothetical LOC298504 (predicted)	RGD1310174_predicted	0.4	0.006830822	0.035631654
1376626_at	similar to Mkrn1 protein	MGC94941	0.6	0.006846201	0.035670832
1392077_at	Transcribed locus	---	0.6	0.006846596	0.035670832

1390254_at	Transcribed locus	---	0.8	0.006885374	0.035837922
1395354_at	lanC (bacterial lantibiotic synthetase component C)-like 1	Lancl1	0.3	0.006887383	0.035837922
1377646_at	Transcribed locus	---	0.6	0.006903863	0.035912317
1378131_at	similar to solute carrier family 9 (sodium/hydrogen exchanger), isoform 9 (predicted)	RGD1560736_predicted	0.5	0.006915151	0.035927296
1390114_at	myelin protein zero-like 1	Mpzl1	0.6	0.006929001	0.035952115
1377791_at	Ras-related GTP binding B	RragB	0.7	0.00693335	0.035963336
1393096_at	---	---	0.8	0.006943829	0.035983648
1374176_at	similar to DNA segment, Chr 4, Brigham & Womens Genetics 0951 expressed	RGD1308059	0.5	0.006975039	0.036134
1389605_at	multiple coiled-coil GABABR1-binding protein	MGC125215	0.6	0.007003206	0.036230857
1379911_at	death associated protein kinase 1 (predicted)	Dapk1_predicted	0.7	0.007011354	0.036253614
1387003_at	leucine zipper protein 1	Luzp1	0.6	0.007035685	0.03634917
1376933_at	Transcribed locus	---	0.7	0.007036464	0.03634917
1374116_at	Similar to Metaxin 1, isoform 2	LOC295241	0.6	0.00704256	0.036369238
1388652_at	Transcribed locus	---	0.7	0.007056002	0.036394374
1376463_at	Transcribed locus	---	0.6	0.007060045	0.03639899
1384001_at	Transcribed locus	---	0.4	0.00707217	0.036430648
1377392_at	Transcribed locus	---	0.6	0.007118204	0.0366219
1379109_at	---	---	0.5	0.007144433	0.036709105
1377890_at	Transcribed locus	---	0.6	0.007146241	0.036709105
1369090_at	protein kinase, cGMP-dependent, type II	Prkg2	0.6	0.007146313	0.036709105
1384247_at	Transcribed locus	---	0.6	0.00715214	0.036716103
1374592_at	similar to BTB/POZ domain containing protein 6	LOC684566	0.6	0.007156375	0.03672408
1376885_at	Transcribed locus	---	0.7	0.007158159	0.03672408
1373605_at	Similar to 106 kDa O-GlcNAc transferase-interacting protein (predicted)	RGD1307844_predicted	0.6	0.007169849	0.036772582
1374741_at	---	---	0.7	0.0071918	0.03683618
1384538_at	misato homolog 1 (Drosophila) (predicted)	Msto1_predicted	0.6	0.007193565	0.03683618
1385663_at	ubiquitin specific protease 13 (isopeptidase T-3) (predicted)	Usp13_predicted	0.6	0.007195686	0.03683618
1391111_at	Transcribed locus	---	0.7	0.007264265	0.037117936
1370268_at	potassium voltage-gated channel, shaker-related subfamily, member 5	Kcna5	0.6	0.007282968	0.037178849
1377860_at	similar to hypothetical protein FLJ33868 (predicted)	RGD1305797_predicted	0.7	0.007297658	0.037242286
1372131_at	ubiquilin 2 (predicted)	Ubqln2_predicted	0.7	0.00730518	0.037269105
1393635_x_at	tubulin, beta 4	Tubb4	0.6	0.007330085	0.037361399
1385074_at	SWI/SNF related, matrix associated, actin dependent regulator of chromatin, subfamily a, member 2	Smarca2	0.7	0.007344884	0.037407054
1389677_at	synapse defective 1, Rho GTPase, homolog 1 (C. elegans) (predicted)	Syde1_predicted	0.7	0.007345865	0.037407054
1377770_at	Transcribed locus	---	0.6	0.007369992	0.0375183
1374026_at	similar to zinc finger protein 341	LOC296300	0.7	0.007409731	0.037673959
1387175_a_at	HLA-B-associated transcript 3	Bat3	0.6	0.007419834	0.037705144
1367789_at	solute carrier family 27 (fatty acid transporter), member 1	Slc27a1	0.7	0.007446975	0.03780489
1371424_at	similar to RIKEN cDNA 3110005O21	RGD1304823	0.7	0.007455468	0.037814869
1373478_at	Myosin binding protein H	Mybph	0.5	0.007455838	0.037814869
1375674_at	similar to chromosome 16 open reading frame 5	RGD1310686	0.7	0.007460265	0.037825661
1367999_at	aldehyde dehydrogenase 2	Aldh2	0.6	0.007466011	0.037831467
1375140_at	similar to muscleblind-like 1 isoform d	LOC686892	0.2	0.007483524	0.037896863
1383444_at	solute carrier family 24 (sodium/potassium/calcium exchanger), member 2	Slc24a2	0.7	0.007556001	0.038193331
1369756_a_at	solute carrier family 4, member 4	Slc4a4	0.7	0.007589133	0.03831371
1374142_at	similar to RIKEN cDNA E130201N16 (predicted)	RGD1311589_predicted	0.7	0.007603578	0.038374855
1388125_a_at	kinesin light chain 1	Klc1	0.3	0.007631286	0.038432148
1389561_at	similar to RIKEN cDNA 1810021J13	RGD1311364	0.6	0.007633628	0.038432177
1378629_at	special AT-rich sequence binding protein 1	Satb1	0.6	0.007655038	0.038501439
1376213_at	similar to Rap2-binding protein 9	LOC682723 /// MGC124740	0.7	0.007656749	0.038501439
1388078_a_at	amiloride-sensitive cation channel 2, neuronal	Accn2	0.4	0.007663043	0.038521309

1388193_at	huntingtin interacting protein 1	Hip1	0.7	0.007687163	0.038607162
1384069_at	low density lipoprotein receptor-related protein 11 (predicted)	Lrp11_predicted	0.7	0.007692534	0.038622342
1398424_at	Transcribed locus	---	0.6	0.007744976	0.038858566
1391213_at	protein phosphatase 3, regulatory subunit B, alpha isoform (calcineurin B, type I) /// similar to Calcineurin subunit B isoform 1 (Protein phosphatase 2B regulatory subunit 1) (Protein phosphatase 3 regulatory subunit B alpha isoform 1)	LOC300429 /// Ppp3r1	0.7	0.007746671	0.038858566
1388336_at	similar to hypothetical protein D2Ertd391e (predicted)	RGD1310685_predicted	0.6	0.007751661	0.038871745
1393825_at	guanine nucleotide binding protein, alpha 14	Gna14	0.4	0.007773402	0.038957012
1369847_at	potassium voltage-gated channel, shaker-related subfamily, beta member 1	Kcnab1	0.5	0.007779407	0.03897523
1388582_at	proteaseome (prosome, macropain) 28 subunit, 3	Psme3	0.5	0.007802019	0.039044564
1367756_at	G elongation factor	Gfm	0.7	0.007834314	0.039142381
1368065_at	GIPC PDZ domain containing family, member 1	Gipc1	0.5	0.007852492	0.039198114
1389335_at	WD repeat domain 22	Wdr22	0.6	0.007856688	0.039207163
1398624_x_at	tubulin, beta 4	Tubb4	0.5	0.007863364	0.039228574
1394395_at	Transcribed locus	---	0.6	0.007894088	0.039357977
1390552_at	membrane associated guanylate kinase 1 b NT-short isoform	LOC500261	0.6	0.007929024	0.039484285
1388366_at	mitochondrial ribosomal protein L4 (predicted)	Mrpl4_predicted	0.8	0.007951586	0.039572674
1383903_at	ST8 alpha-N-acetyl-neuraminide alpha-2,8-sialyltransferase 5	St8sia5	0.7	0.007958556	0.039594786
1382451_at	heme binding protein 2 (predicted)	Hebp2_predicted	0.6	0.007960844	0.039594786
1388588_at	mammary tumor virus receptor 2	Mtvr2	0.5	0.007972528	0.039640913
1394931_at	similar to muscleblind-like 1 isoform d	LOC686892	0.4	0.007977533	0.039653813
1370066_at	Kelch-like ECH-associated protein 1	Keap1	0.6	0.00798901	0.039684349
1386023_at	leucine-rich repeat LGI family, member 1	Lgi1	0.6	0.007990915	0.039684349
1379913_at	ubiquitin specific protease 13 (isopeptidase T-3) (predicted)	Usp13_predicted	0.6	0.00800546	0.039720606
1396512_at	Transcribed locus	---	0.5	0.008017582	0.039756765
1380072_at	similar to 2310044H10Rik protein	MGC93975	0.6	0.008057294	0.039881545
1368069_at	SHANK-associated RH domain interacting protein	Sharpin	0.6	0.008063156	0.039898552
1372261_at	Transcribed locus	---	0.7	0.008069587	0.039918366
1383229_at	ATP-binding cassette, sub-family A (ABC1), member 7	Abca7	0.5	0.008082444	0.039969948
1367889_at	calcium/calmodulin-dependent protein kinase I	Camk1	0.7	0.00809804	0.040010982
1376872_at	plexin C1 (predicted)	Plxnc1_predicted	0.7	0.008131971	0.040154505
1376907_at	Microtubule-associated protein 6	Mtap6	0.7	0.008136738	0.040165985
1390597_at	Transcribed locus	---	0.7	0.008144726	0.040193357
1373422_at	La ribonucleoprotein domain family, member 1 (predicted)	Larp1_predicted	0.5	0.008166969	0.040266877
1383302_at	DnaJ (Hsp40) homolog, subfamily B, member 1 (predicted)	Dnajb1_predicted	0.6	0.008180821	0.040311007
1394430_at	Transcribed locus	---	0.6	0.008201547	0.040377359
1369046_at	synaptotagmin VI	Syt6	0.4	0.008203363	0.040377359
1388584_at	Transcribed locus	---	0.7	0.008204106	0.040377359
1387057_at	solute carrier family 7 (cationic amino acid transporter, y+ system), member 8 /// synaptic Ras GTPase activating protein 1 homolog (rat)	Slc7a8 /// Syngap1	0.7	0.008224015	0.040451134
1378818_at	Transcribed locus	---	0.5	0.008227069	0.04045406
1368035_a_at	protein tyrosine phosphatase, receptor type, F	Ptprf	0.7	0.008231472	0.040463616
1380648_at	Mitogen activated protein kinase 10	Mapk10	0.7	0.008241912	0.040502827
1386988_at	deformed epidermal autoregulatory factor 1 (Drosophila)	Deaf1	0.5	0.008254908	0.040534945
1383765_at	similar to RIKEN cDNA 4921520P21	MGC114388	0.5	0.00825617	0.040534945
1373637_at	---	---	0.6	0.008257817	0.040534945
1388690_at	breast cancer metastasis-suppressor 1-like (predicted)	Brms1l_predicted	0.7	0.008260095	0.040534945
1393143_at	Transcribed locus	---	0.7	0.008278415	0.040609417
1387132_at	lipase, hormone sensitive	Lipe	0.7	0.008284301	0.040626175
1389420_at	signal-transducing adaptor protein-2	Stap2	0.7	0.00829298	0.040656613

1389128_at	WD repeat and FYVE domain containing 3 (predicted)	Wdfy3_predicted	0.6	0.008302503	0.040666938
1385836_at	---	---	0.7	0.008343559	0.040843706
1376637_at	Transcribed locus	---	0.7	0.008354379	0.040884507
1369666_at	Glycerol-3-phosphate dehydrate dehydrogenase (mtGPDH) mRNA, 3'UTR, mRNA sequence	---	0.7	0.008380523	0.04098806
1394957_at	nischarin	Nisch	0.4	0.008401102	0.041042234
1395817_at	Transcribed locus	---	0.5	0.008401581	0.041042234
1388593_at	mitogen-activated protein kinase 8 interacting protein 3	Mapk8ip3	0.7	0.008404438	0.041044001
1389185_at	Adenylate kinase 1	Ak1	0.5	0.008409589	0.041056967
1381158_at	speedy homolog 1 (Drosophila)	Spdy1	0.6	0.008420649	0.041086571
1392518_at	similar to Protein C22orf5	RGD1306591	0.7	0.008423921	0.041090347
1370158_at	myosin, heavy polypeptide 10, non-muscle	Myh10	0.8	0.008437521	0.041132289
1394590_at	NFKB inhibitor interacting Ras-like protein 1 (predicted)	Nkiras1_predicted	0.6	0.008458446	0.041209231
1394567_at	---	---	0.7	0.00846082	0.041209231
1392161_at	ATP synthase mitochondrial F1 complex assembly factor 1 (predicted)	Atpaf1_predicted	0.6	0.008464295	0.041213948
1390437_at	Transcribed locus	---	0.5	0.008503249	0.041330212
1388121_at	amyloid beta (A4) precursor-like protein 2	Aplp2	0.3	0.008578469	0.041609748
1379325_at	---	---	0.6	0.008589172	0.041637106
1374681_at	Transcribed locus	---	0.7	0.008594469	0.041643841
1367876_at	importin 13	Ipo13	0.5	0.008596885	0.041643841
1388888_at	---	---	0.8	0.008600689	0.041643841
1382555_at	Transcribed locus	---	0.7	0.008633207	0.041764409
1376654_at	similar to RIKEN cDNA B130016O10 gene (predicted)	RGD1308448_predicted	0.7	0.008660788	0.041860901
1382331_at	similar to RIKEN cDNA 0610038L10 gene (predicted) /// riboflavin kinase	Rfk /// RGD1560961_predicted	0.4	0.008678247	0.041920647
1390818_at	Transcribed locus	---	0.7	0.008685443	0.041930778
1370823_at	BMP and activin membrane-bound inhibitor, homolog (Xenopus laevis)	Bambi	0.5	0.008726366	0.042075245
1382265_at	similar to KIAA1128 protein (predicted)	RGD1304626_predicted	0.7	0.008737244	0.042104871
1370821_at	similar to Thiopurine S-methyltransferase (Thiopurine methyltransferase)	LOC681730	0.8	0.008739423	0.042104871
1376388_at	Peroxin 2	Pex2	0.7	0.008742675	0.042105866
AFFX_Rat_Hexo	hexokinase 1	Hk1	0.5	0.008744749	0.042105866
1377650_at	Transcribed locus	---	0.7	0.008770973	0.042170407
1385675_at	Transcribed locus	---	0.8	0.008782254	0.04220454
1397644_at	---	---	0.7	0.008783204	0.04220454
1388196_at	NCK-associated protein 1	Nckap1	0.7	0.008810618	0.042286853
1393063_at	Transcribed locus	---	0.7	0.008819308	0.042316213
1374187_at	---	---	0.7	0.008829429	0.042340072
1371526_at	ring finger protein 10	Rnf10	0.7	0.008836594	0.042362079
1379566_at	RNA binding motif protein 11 (predicted)	Rbm11_predicted	0.6	0.008868987	0.042468038
1390546_at	---	---	0.8	0.008876318	0.042472133
1367909_at	dicarbonyl L-xylulose reductase	Dcxr	0.5	0.008877626	0.042472133
1368156_at	calcium/calmodulin-dependent protein kinase kinase 1, alpha	Camkk1	0.4	0.008921765	0.042621316
1374832_at	Transcribed locus	---	0.6	0.008956135	0.042760672
1388811_at	Synapsin II	Syn2	0.7	0.008978349	0.042812374
1390954_at	protein phosphatase 1H (PP2C domain containing)	Ppm1h	0.7	0.008982582	0.042812374
1370616_at	neuregulin 1	Nrg1	0.7	0.008989051	0.042818392
1368086_a_at	lanosterol synthase	Lss	0.5	0.009025018	0.042968139
1371650_at	SUMO/sentrin specific peptidase 3	Senp3	0.8	0.009025713	0.042968139
1384128_at	glycine-, glutamate-, thienylcyclohexylpiperidine-binding protein	LOC246295	0.7	0.009046586	0.043030144
1378152_at	Transcribed locus	---	0.7	0.009059045	0.043076952
1373084_at	WD repeat domain 20	Wdr20	0.7	0.009067285	0.043097779
1376171_at	ubiquitin specific protease 11	Usp11	0.7	0.009068666	0.043097779
1393983_at	exportin, tRNA (nuclear export receptor for tRNAs) (predicted)	Xpot_predicted	0.7	0.009082373	0.043137994
1371023_at	EGF-like-domain, multiple 4	Egfl4	0.7	0.009088168	0.043153057
1372049_at	similar to RIKEN cDNA 5730411O18 gene	RGD1311827	0.6	0.009098693	0.043167739

1390951_at	Transcribed locus	---	0.7	0.009101367	0.043167739
1367493_at	similar to DNA segment, Chr 18, Wayne State University 98, expressed (predicted)	RGD1560212_predicted	0.6	0.009101758	0.043167739
1376202_at	UDP-GlcNAc:betaGal beta-1,3-N-acetylglucosaminyltransferase 6 (predicted)	B3gnt6_predicted	0.5	0.009113117	0.043208808
1371748_at	1-acylglycerol-3-phosphate O-acyltransferase 1	Agpat1	0.5	0.009124778	0.043227059
1389514_at	leucine rich repeat neuronal 6A	Lrrn6a	0.4	0.009149641	0.043319887
1390864_at	Transcribed locus	---	0.7	0.009167651	0.043373062
1384862_at	Transcribed locus	---	0.6	0.009168958	0.043373062
1382137_at	abhydrolase domain containing 3 (predicted)	Abhd3_predicted	0.7	0.009173031	0.043373062
1376402_a_at	RAS-like, family 10, member B (predicted)	Rasl10b_predicted	0.8	0.009184309	0.043394069
1392210_at	similar to mKIAA0319 protein (predicted)	RGD1307443_predicted	0.5	0.009186415	0.043394069
1387521_at	programmed cell death 4	Pdcd4	0.5	0.009198143	0.043424528
1376668_at	similar to RIKEN cDNA 4922503N01 (predicted)	RGD1311126_predicted	0.6	0.009248968	0.043618941
1374960_at	similar to 9130011E15Rik protein (predicted)	RGD1564887_predicted	0.7	0.009249931	0.043618941
1383593_at	Transcribed locus	---	0.7	0.009265955	0.043663038
1384190_at	mitogen-activated protein kinase 8 interacting protein 3	Mapk8ip3	0.4	0.009272556	0.043663038
1372381_at	Transcribed locus	---	0.7	0.009280902	0.043689833
1371602_at	tetraspanin 9 (predicted)	Tspan9_predicted	0.8	0.009300963	0.043771741
1381176_at	Transcribed locus	---	0.7	0.009315226	0.043813793
1370409_at	solute carrier family 38, member 1	Slc38a1	0.7	0.009330382	0.043869372
1385758_at	SAPS domain family, member 1 (predicted)	Saps1_predicted	0.7	0.009333399	0.043869372
1372580_at	MAP-kinase activating death domain	Madd	0.6	0.009368653	0.04399369
1373278_at	nuclear factor, erythroid derived 2,-like 1 (predicted)	Nfe2l1_predicted	0.7	0.00939518	0.044086734
1367762_at	somatostatin	Sst	0.4	0.009411286	0.044139389
1373503_at	leucine-rich repeats and calponin homology (CH) domain containing 4 (predicted)	Lrch4_predicted	0.7	0.009414993	0.044144184
1367467_at	similar to nitrogen fixation cluster-like (predicted)	RGD1309562_predicted	0.5	0.009421433	0.044161791
1389464_at	ligand of numb-protein X 1 (predicted)	Lnx1_predicted	0.7	0.009436336	0.044193866
1398297_at	mitogen-activated protein kinase 12	Mapk12	0.5	0.009440996	0.044203103
1372521_at	Rho family GTPase 2	Rnd2	0.7	0.009477515	0.044351429
1369428_a_at	5-hydroxytryptamine (serotonin) receptor 3a	Htr3a	0.3	0.009480106	0.044351429
1370556_at	vesicle-associated membrane protein 1	Vamp1	0.2	0.009480765	0.044351429
1376680_at	---	---	0.7	0.009489652	0.044380381
1381812_at	Transcribed locus	---	0.7	0.009504123	0.044422794
1389735_at	ribosomal protein S6 kinase polypeptide 6 (predicted)	Rps6ka6_predicted	0.7	0.009508505	0.04443065
1376233_at	Discs, large (Drosophila) homolog-associated protein 3	Dlgap3	0.6	0.009517942	0.044462117
1370335_at	disabled homolog 2 (Drosophila) interacting protein	Dab2ip	0.7	0.009522395	0.044470286
1367894_at	similar to Insulin-induced gene 1 protein (INSIG-1) (Insulin-induced growth response protein CL-6) (Immediate-early protein CL-6)	LOC683385 /// LOC688922	0.6	0.009542923	0.044528227
1369562_at	hippocalcin-like 1	Hpcal1	0.5	0.009560847	0.044599211
1386959_a_at	mitogen activated protein kinase kinase 5	Map2k5	0.7	0.009577879	0.044638549
1388037_at	ATPase, Ca++ transporting, plasma membrane 3	Atp2b3	0.5	0.009580083	0.044638549
1379545_at	Transcribed locus	---	0.7	0.009592291	0.044657231
1379091_at	similar to D8Ertd354e protein (predicted)	RGD1560755_predicted	0.6	0.009681328	0.044998439
1389171_at	transmembrane protein 38a (predicted)	Tmem38a_predicted	0.5	0.009686685	0.044998439
1383451_at	WD repeat and SOCS box-containing 2	Wsb2	0.6	0.009690204	0.044998439
1392974_at	WAS protein family, member 1	Wasf1	0.7	0.009704193	0.045044077
1383669_at	---	---	0.8	0.009705509	0.045044077
1377526_at	sorting nexin family member 27	Snx27	0.5	0.009715726	0.045066065
1384315_at	pyruvate dehydrogenase kinase, isoenzyme 3	Pdk3	0.5	0.009720056	0.045073438
1383224_at	---	---	0.8	0.009726747	0.045083819

1394264_at	similar to N-acetylglucosamine 6-O-sulfotransferase (predicted)	RGD1561144_predicted	0.6	0.009728068	0.045083819
1390854_at	transmembrane protein 24	Tmem24	0.7	0.009731635	0.045083819
1388341_at	RAN GTPase activating protein 1	Rangap1	0.5	0.00974435	0.045108804
1368476_at	nuclear receptor subfamily 3, group C, member 2	Nr3c2	0.7	0.009753677	0.045127563
1398343_at	similar to DnaJ (Hsp40) homolog, subfamily A, member 4	LOC498996	0.6	0.009787897	0.045260425
1388600_at	CDNA clone IMAGE:7320582	---	0.8	0.009820486	0.045372852
1382428_at	taspase, threonine aspartase 1 (predicted)	Tasp1_predicted	0.7	0.009829447	0.045388756
1391698_at	microtubule associated serine/threonine kinase 1	MAST1	0.5	0.009838104	0.045415978
1394097_at	Transcribed locus	---	0.7	0.009892818	0.045579012
1386941_at	plectin 1	Plec1	0.7	0.009918364	0.04568391
1388486_at	dipeptidylpeptidase 8 (predicted)	Dpp8_predicted	0.6	0.009924772	0.045700628
1368994_a_at	GTPase activating RANGAP domain-like 1	Garnl1	0.4	0.009931273	0.045717766
1381394_at	sorting nexin family member 27	Snx27	0.6	0.009952135	0.045786228
1376449_at	---	---	0.7	0.009954496	0.045786228
1374275_at	carbonic anhydrase 11	Car11	0.6	0.00996423	0.045818187
1371584_at	transient receptor potential cation channel, subfamily C, member 4 associated protein	Trpc4ap	0.7	0.009997956	0.045947578
1376878_at	similar to RIKEN cDNA 2310022B05 (predicted)	RGD1559896_predicted	0.7	0.010003542	0.045960409
1388334_at	similar to SEC14-like 1 (predicted)	RGD1563123_predicted	0.6	0.010007148	0.045964134
1368928_at	tripartite motif protein 3	Trim3	0.4	0.010069738	0.046174254
1372103_at	similar to DnaJ (Hsp40) homolog, subfamily A, member 4	LOC498996	0.6	0.010079882	0.046207886
1380013_at	Transcribed locus	---	0.7	0.010085923	0.046218199
1368309_at	thioredoxin reductase 2	Txnrd2	0.7	0.010100568	0.04623828
1390388_at	ferrochelatase (predicted)	Fech_predicted	0.7	0.010118373	0.0463069
1384469_at	ATP-binding cassette, sub-family A (ABC1), member 5	Abca5	0.5	0.010127523	0.046335881
1384656_at	---	---	0.7	0.010183946	0.046528792
1374120_at	similar to KIAA1126 protein (predicted)	RGD1309971_predicted	0.7	0.010192027	0.046540382
1387182_at	G protein-coupled receptor 37	Gpr37	0.6	0.010201562	0.046570993
1374483_at	Transcribed locus	---	0.8	0.010223406	0.046657713
1373752_at	DEP domain containing 5 (predicted)	Depdc5_predicted	0.6	0.01023046	0.04666406
1392495_at	similar to dJ202D23.2 (novel protein similar to C21ORF5 (KIAA0933)) (predicted)	RGD1305534_predicted	0.7	0.010294199	0.046876796
1370030_at	glutamate cysteine ligase, modifier subunit	Gclm	0.6	0.010297139	0.046877206
1372184_at	similar to hypothetical protein BC011833 (predicted)	RGD1309188_predicted	0.4	0.010314007	0.046941003
1393995_at	Glutamate receptor, ionotropic, AMPA2	Gria2	0.7	0.010383986	0.047181159
1388802_at	brain expressed X-linked 1	Bex1	0.6	0.010402591	0.047239595
1393133_at	Transcribed locus	---	0.6	0.010433509	0.047328547
1389426_at	glutamate-ammonia ligase (glutamine synthase)	Glul	0.7	0.010451809	0.047384605
1378587_at	similar to ankyrin repeat domain 40	LOC688144 /// LOC690586	0.7	0.010470408	0.047442775
1373500_at	leucine-rich PPR-motif containing	Lrpprc	0.7	0.010561875	0.047765113
1375995_at	similar to KIAA0528 protein (predicted)	RGD1304592_predicted	0.8	0.010582665	0.047835009
1387771_a_at	mitogen activated protein kinase 3	Mapk3	0.4	0.010587139	0.047835009
1370402_at	EGF-like domain 7	Egfl7	0.7	0.01059636	0.047835009
1372572_at	sialyltransferase 7F	Siat7F	0.6	0.010597688	0.047835009
1368034_at	chromogranin B	Chgb	0.6	0.01062332	0.0479244
1384106_at	transmembrane protein 23	Tmem23	0.7	0.010629476	0.047925883
1398660_at	Solute carrier family 35, member F2 (predicted)	Slc35f2_predicted	0.7	0.010659642	0.048035559
1387155_at	proprotein convertase subtilisin/kexin type 2	Pcsk2	0.5	0.01066753	0.048057939
1384903_at	glutamic pyruvate transaminase (alanine aminotransferase) 2 (predicted)	Gpt2_predicted	0.7	0.010706445	0.048159383
1397740_at	sideroflexin 1	Sfxn1	0.6	0.010707642	0.048159383

1394637_at	Similar to Leucine-rich repeats and calponin homology domain-containing protein 1 (Calponin homology domain-containing protein 1)	LOC502020	0.7	0.010721022	0.048193324
1374077_at	Phosphatidylserine synthase 1	Ptdss1	0.7	0.010732898	0.048220352
1377331_at	Transcribed locus	---	0.8	0.010818599	0.048486192
1387447_at	ADP-ribosylation factor 3	Arf3	0.6	0.010873619	0.04865916
1374459_at	asparagine-linked glycosylation 2 homolog (yeast, alpha-1,3-mannosyltransferase)	Alg2	0.7	0.01087863	0.04865916
1367815_at	solute carrier family 5 (sodium-dependent vitamin transporter), member 6	Slc5a6	0.6	0.01088086	0.04865916
1373019_at	Transcribed locus	---	0.7	0.010946593	0.048873387
1395467_at	similar to N-copine /// similar to Copine-6 (Copine VI) (Neuronal-copine) (N-copine)	LOC305894 /// LOC691478	0.7	0.010966536	0.048924116
1367842_at	amyloid beta (A4) precursor protein-binding, family B, member 1	Apbb1	0.5	0.010966878	0.048924116
1388898_at	heat shock 105kDa/110kDa protein 1	Hsph1	0.5	0.010989885	0.04897362
1378555_at	Transcribed locus	---	0.7	0.011004256	0.049000199
1380023_at	Transcribed locus	---	0.8	0.011030563	0.049101677
1368138_at	microtubule-associated protein tau	Mapt	0.7	0.011039745	0.049129256
1393072_at	ubiquitin-conjugating enzyme E2Q (putative) 2 (predicted)	Ube2q2_predicted	0.6	0.011043382	0.049132148
1368978_at	scrapie responsive gene 1	Scrg1	0.7	0.011086362	0.049283376
1382477_at	SUMO/sentrin specific protease 5 (predicted) /// similar to SUMO/sentrin specific protease 5 (predicted) /// similar to SUMO/sentrin specific protease 5	LOC686286 /// RGD1564247_predicted /// Senp5_predicted	0.7	0.011091977	0.049295012
1395311_at	isoprenylcysteine carboxyl methyltransferase	Icmt	0.6	0.011118133	0.049360034
1367649_at	paralemmin	Palm	0.7	0.011118612	0.049360034
1395726_at	Transcribed locus	---	0.4	0.011143323	0.049443048
1368807_at	reticulon 3	Rtn3	0.8	0.011152882	0.049445444
1368667_at	purinergic receptor P2X, ligand-gated ion channel, 3	P2rx3	0.6	0.011160518	0.049465967
1388396_at	serine/threonine kinase 25 (STE20 homolog, yeast)	Stk25	0.7	0.011251986	0.049790869
1379607_at	Transcribed locus	---	0.7	0.01126657	0.049839752
1397824_at	similar to WAC (predicted)	RGD1562407_predicted	0.7	0.011270627	0.049839752
1371819_at	histone deacetylase 5	Hdac5	0.6	0.011275072	0.049839752
1373602_at	leucyl-tRNA synthetase, mitochondrial (predicted)	Lars2_predicted	0.7	0.01128482	0.049864217
1389124_at	similar to ankyrin repeat domain 40	LOC688144 /// LOC690586	0.7	0.011310519	0.049934837
1388653_at	similar to Paired amphipathic helix protein Sin3b (Transcriptional corepressor Sin3b) (Histone deacetylase complex subunit Sin3b)	LOC683381	0.7	0.011312506	0.049934837
1391118_at	Transcribed locus	---	0.5	0.011320748	0.049934837
1385826_at	Transcribed locus	---	0.5	0.011331707	0.049942099
1371772_at	alkB, alkylation repair homolog 3 (E. coli)	Alkbh3	0.7	0.011350654	0.049998811
1373922_at	Transcribed locus	---	0.7	0.011367391	0.050059128
1373211_at	F-box and WD-40 domain protein 4 (predicted)	Fbxw4_predicted	0.7	0.011376393	0.050085364
1370690_at	heat shock 70kDa protein 9A (predicted)	Hspa9a_predicted	0.4	0.01138638	0.050115915
1381125_at	Tetraspanin 8	Tspan8	0.7	0.011449006	0.050364604
1392505_at	---	---	0.8	0.011552533	0.050792854
1370300_at	prolactin regulatory element binding	Preb	0.5	0.01156625	0.050836735
1374541_at	similar to glycogen synthase 1, muscle	LOC687978 /// LOC690987	0.3	0.011568695	0.050836735
1383771_at	similar to ubiquitin specific protease 46 (predicted)	RGD1564808_predicted	0.6	0.01158297	0.050872282
1368075_at	lysosomal acid lipase 1	Lip1	0.7	0.011591204	0.05087813
1384041_at	Transcribed locus	---	0.6	0.011596588	0.05087813
1388719_at	Ubiquilin 1	Ubqln1	0.7	0.011602276	0.05087813
1376806_at	Transcribed locus	---	0.7	0.011606359	0.050879902
1398112_at	similar to Putative protein C21orf56 homolog	LOC499418	0.7	0.011616772	0.05089842
1389635_at	dishevelled 3, dsh homolog (Drosophila) (predicted)	Dvl3_predicted	0.7	0.011664461	0.051080152
1385182_at	plakophilin 1 (predicted)	Pkp1_predicted	0.6	0.011676081	0.051117427
1377955_at	CDNA clone IMAGE:7383152	---	0.7	0.011682674	0.051121115

1392939_at	solute carrier family 41, member 3	Slc41a3	0.7	0.011683139	0.051121115
1374312_at	uridine-cytidine kinase 1 (predicted)	Uck1_predicted	0.6	0.011722881	0.051254107
1390785_at	Transcribed locus	---	0.7	0.011727746	0.051260464
1377795_at	Transcribed locus	---	0.8	0.011745369	0.051289708
1370700_at	phosphate cytidylyltransferase 1, choline, beta isoform	Pcyt1b	0.6	0.011749734	0.051289708
1398403_at	---	---	0.7	0.011753059	0.051290609
1371615_at	diacylglycerol O-acyltransferase homolog 2 (mouse)	Dgat2	0.7	0.011786782	0.051410497
1376494_at	polymerase (DNA directed), epsilon 3 (p17 subunit)	Pole3	0.6	0.011804872	0.051443188
1375934_at	similar to RIKEN cDNA D330045A20 (predicted)	RGD1566282_predicted	0.7	0.011825354	0.051496802
1371342_at	cytochrome c-1 (predicted)	Cyc1_predicted	0.7	0.011830431	0.051497253
1369106_at	transcription elongation factor A (SII), 2	Tcea2	0.5	0.011853809	0.051570777
1393584_at	tumor necrosis factor receptor superfamily, member 21 (predicted)	Tnfrsf21_predicted	0.4	0.011854883	0.051570777
1387602_a_at	5-hydroxytryptamine (serotonin) receptor 3b	Htr3b	0.2	0.011953451	0.05190894
1370545_at	potassium voltage-gated channel, shaker-related subfamily, member 1	Kcna1	0.7	0.011996501	0.052049181
1377141_at	similar to UPF0308 protein C9orf21	LOC498685	0.8	0.01205619	0.052253019
1386755_at	similar to RIKEN cDNA 1810060J02	LOC312863	0.4	0.012122224	0.052486741
1382344_at	Transcribed locus	---	0.7	0.01212288	0.052486741
1387485_a_at	protein phosphatase 2 (formerly 2A), regulatory subunit B (PR 52), alpha isoform	Ppp2r2a	0.6	0.01214662	0.052533607
1389129_at	similar to mKIAA0665 protein	RGD1308952	0.7	0.012147204	0.052533607
1372248_at	sestrin 1 (predicted)	Sesn1_predicted	0.7	0.012183247	0.052623436
1394047_at	---	---	0.7	0.012200908	0.052658235
1372465_at	nucleolar protein family 6 (RNA-associated) (predicted)	Nol6_predicted	0.8	0.012212279	0.052693484
1381883_at	CDNA clone IMAGE:7315883	---	0.7	0.012273543	0.052916184
1376771_at	Transcribed locus	---	0.7	0.012363567	0.053233959
1373269_at	Transcribed locus	---	0.7	0.012372756	0.053246231
1382752_at	---	---	0.8	0.012399424	0.053319146
1388819_at	secretory carrier membrane protein 1	Scamp1	0.5	0.012403938	0.053324612
1390294_at	Transcribed locus	---	0.5	0.012467934	0.053523705
1389509_at	Sin3A associated protein 130 (predicted)	Sap130_predicted	0.8	0.012504227	0.05364359
1372140_at	similar to chromosome 6 open reading frame 80; chemokine C-C motif receptor-like 1 adjacent	RGD1310326	0.6	0.012507832	0.053645064
1393181_at	Transcribed locus	---	0.4	0.012518435	0.053662553
1384157_at	similar to ADP-ribosylation factor-like 10C	LOC500282	0.4	0.012560809	0.053816147
1369651_at	thymus cell antigen 1, theta	Thy1	0.7	0.012564866	0.053819511
1388027_a_at	reticulon 4	Rtn4	0.4	0.012642275	0.054114399
1384398_at	WW domain containing E3 ubiquitin protein ligase 2 (predicted)	Wwp2_predicted	0.6	0.012655704	0.05412403
1386120_at	hypothetical protein LOC679389 /// hypothetical protein LOC689147	LOC679389 /// LOC689147	0.6	0.012675333	0.054189548
1390456_at	Transcribed locus	---	0.8	0.012677613	0.054189548
1383869_at	Transcribed locus	---	0.7	0.012703259	0.054242784
1372832_at	Transcribed locus	---	0.6	0.012720486	0.054302244
1379519_at	Transcribed locus	---	0.6	0.012763171	0.054417051
1380850_at	Transcribed locus	---	0.6	0.012770336	0.054417051
1388259_at	small nuclear ribonucleoprotein N	Snrpn	0.6	0.012779334	0.054417051
1372874_at	Serologically defined colon cancer antigen 8	Sdccag8	0.7	0.01278674	0.054417051
1371932_at	similar to hypothetical protein FLJ38482	RGD1309341	0.7	0.012790758	0.054417051
1393847_at	NFKB inhibitor interacting Ras-like protein 1 (predicted)	Nkiras1_predicted	0.6	0.012793698	0.054417051
1379073_at	similar to solute carrier family 25 (mitochondrial carrier; phosphate carrier), member 23	LOC301113	0.6	0.012803106	0.054442989
1392477_at	ets variant gene 1 (predicted)	Etv1_predicted	0.8	0.012809646	0.054456721
1387900_at	CDP-diacylglycerol--inositol 3-phosphatidyltransferase (phosphatidylinositol synthase)	Cdipt	0.6	0.012814798	0.054464549
1376738_at	tetratricopeptide repeat domain 23	Ttc23	0.7	0.012836792	0.054543932
1388475_at	similar to prion protein interacting protein 1	LOC313535	0.8	0.012857838	0.054618119
1368052_at	tetraspanin 8	Tspan8	0.6	0.012860893	0.054618119

1385622_at	---	---	0.6	0.012868618	0.054636816
1389759_at	cadherin EGF LAG seven-pass G-type receptor 1	Celsr1	0.8	0.012882819	0.054682994
1370318_at	phosphatidylinositol 4-kinase, catalytic, alpha polypeptide	Pik4ca	0.8	0.012903801	0.054743803
1389552_at	LIM and senescent cell antigen-like domains 1 (predicted)	Lims1_predicted	0.7	0.012957911	0.054930915
1384227_at	Protein tyrosine phosphatase, receptor type, K, extracellular region	Ptprk	0.6	0.012961424	0.05493159
1391305_at	---	---	0.5	0.013043269	0.055164743
1386154_at	similar to KIAA1411 protein (predicted)	RGD1304927_predicted	0.8	0.013069192	0.0552458
1372924_at	Transcribed locus, strongly similar to XP_001077303.1 similar to mediator of RNA polymerase II transcription, subunit 9 homolog [Rattus norvegicus]	---	0.6	0.013069215	0.0552458
1376787_at	similar to hypothetical protein FLJ31810 (predicted)	RGD1310773_predicted	0.7	0.013072511	0.0552458
1373300_at	---	---	0.4	0.013080906	0.055258826
1395384_at	ubiquitin specific protease 31 (predicted)	Usp31_predicted	0.7	0.013082312	0.055258826
1395540_at	similar to Solute carrier family 35, member E3 (predicted)	RGD1564876_predicted	0.6	0.013107171	0.055349617
1385254_at	similar to Serine/threonine-protein kinase ATR (Ataxia telangiectasia and Rad3-related protein)	LOC685055	0.7	0.013116895	0.055363937
1392994_at	similar to Nicotinamide riboside kinase 1	LOC499330	0.7	0.013119743	0.055363937
1389155_at	downstream of Stk11 (predicted)	Dos_predicted	0.6	0.013124027	0.055363937
1382259_a_at	---	---	0.6	0.013138205	0.055407342
1393952_at	similar to CTCL tumor antigen se57-1 (predicted)	RGD1561424_predicted	0.6	0.013141053	0.055407342
1398425_at	similar to hypothetical protein MGC39325-like protein	MGC109194	0.8	0.013175986	0.055497725
1382131_at	Transcribed locus	---	0.7	0.013204993	0.055578936
1373282_at	similar to mitochondrial carrier protein MGC4399	LOC686808	0.6	0.013205687	0.055578936
1373993_at	Similar to small nuclear RNA activating complex, polypeptide 5	LOC691501	0.3	0.013215726	0.055589883
1386685_at	sacsin (predicted)	Sacs_predicted	0.4	0.013218144	0.055589883
1367609_at	macrophage migration inhibitory factor	Mif	0.6	0.013241071	0.055643622
1397815_at	similar to ADP-ribosylation factor-like 10C	LOC500282	0.4	0.013267638	0.055712564
1371113_a_at	transferrin receptor	Tfrc	0.4	0.013279142	0.055742272
1373635_at	similar to Myb protein P42POP (predicted)	RGD1565160_predicted	0.6	0.013281491	0.055742272
1377456_at	---	---	0.8	0.013336468	0.055901688
1382858_at	intimal thickness-related receptor	MGC94555	0.7	0.013400962	0.056043485
1385285_at	Transcribed locus	---	0.6	0.013406471	0.05605227
1370527_a_at	casein kinase 1, delta	Csnk1d	0.4	0.013411301	0.056058215
1393707_at	Bcl2-like 2	Bcl2l2	0.6	0.013426364	0.056102551
1390253_at	ankyrin repeat domain 9	Ankrd9	0.6	0.01344794	0.05615428
1385124_at	similar to Tribbles homolog 2 (predicted)	RGD1564451_predicted	0.7	0.013461281	0.056181462
1368345_at	microtubule-associated protein 6	Mtap6	0.6	0.013468826	0.056198693
1395621_at	similar to Mitochondrial carrier triple repeat 1 (predicted)	RGD1565119_predicted	0.6	0.01350622	0.056311866
1371884_at	tetratricopeptide repeat domain 3 (predicted)	Ttc3_predicted	0.8	0.013545326	0.056446294
1371432_at	vesicle amine transport protein 1 homolog (T californica)	Vat1	0.4	0.013652749	0.05678595
1372332_at	Transcribed locus	---	0.8	0.013660788	0.05678595
1383801_at	Transcribed locus	---	0.5	0.013661357	0.05678595
1379443_at	Hermansky-Pudlak syndrome 3 homolog (human) (predicted)	Hps3_predicted	0.7	0.013668812	0.056802583
1376179_at	Inter-alpha trypsin inhibitor, heavy chain 3	Itih3	0.8	0.013677385	0.056823851
1367872_at	adaptor protein complex AP-1, beta 1 subunit	Ap1b1	0.7	0.013701026	0.056844472
1377662_at	pirin	Pir	0.8	0.013702615	0.056844472
1379324_at	Transcribed locus	---	0.6	0.013735378	0.056921012
1387916_at	cytochrome P450 4F6	Cyp4f6	0.7	0.013766502	0.056980884
1369390_a_at	dipeptidylpeptidase 6	Dpp6	0.3	0.013767413	0.056980884
1372731_at	Ring finger protein 135	Rnf135	0.7	0.013770477	0.056980884
1389845_at	Transcribed locus	---	0.7	0.013771008	0.056980884

1389120_at	potassium voltage gated channel, Shaw-related subfamily, member 3	Kcnc3	0.8	0.013829848	0.057154104
1377817_at	Bwk1 leukemia-related gene	Bwk1	0.7	0.013852446	0.057218045
1374783_at	Transcribed locus	---	0.5	0.013859342	0.057218045
1373839_at	Neighbor of Punc E11 (predicted)	Nope_predicted	0.7	0.013861851	0.057218045
1392357_at	Transcribed locus	---	0.7	0.013865841	0.057218045
1370325_at	golgi reassembly stacking protein 2	Gorasp2	0.6	0.01387398	0.057235824
1375897_at	Transcribed locus	---	0.6	0.013899701	0.057309671
1368595_at	matrix metallopeptidase 24	Mmp24	0.7	0.013909591	0.057321853
1389726_at	Transcribed locus	---	0.8	0.013912259	0.057321853
1373129_at	Transcribed locus	---	0.7	0.013925629	0.05734821
1370925_at	similar to Potential phospholipid-transporting ATPase IIB	LOC291411	0.4	0.013943603	0.057385309
1393405_at	Transcribed locus	---	0.7	0.013954946	0.057385309
1372434_at	CASK-interacting protein CIP98	Cip98	0.8	0.013955571	0.057385309
1372728_at	Sortilin 1	Sort1	0.8	0.013997694	0.057529752
1382088_at	Ryanodine receptor 2, cardiac	Ryr2	0.7	0.014005015	0.057545463
1379203_at	transcription factor Dp 2 (predicted)	Tcfdp2_predicted	0.7	0.014031273	0.057638955
1375213_at	phosphoenolpyruvate carboxykinase 2 (mitochondrial) (predicted)	Pck2_predicted	0.5	0.014037996	0.057652175
1372943_at	similar to Ubiquitin-associated protein 2-like	LOC497952	0.6	0.014070005	0.057769205
1378774_at	Procollagen, type XXIII, alpha 1	Col23a1	0.7	0.014101005	0.057882038
1388691_at	ubiquilin 4 (predicted)	Ubqln4_predicted	0.8	0.01410476	0.057883009
1397637_at	Transcribed locus	---	0.6	0.014127369	0.057946872
1370990_at	similar to cytokine receptor related protein 4	Cytor4	0.6	0.014145151	0.058001183
1389317_at	similar to hypothetical protein FLJ20507 (predicted)	RGD1309744_predicted	0.8	0.014196562	0.058143689
1390472_at	N-acylsphingosine amidohydrolase (acid ceramidase)-like (predicted)	Asahl_predicted	0.7	0.014238542	0.058301106
1373127_at	Transcribed locus	---	0.7	0.014242608	0.058303241
1372071_at	CD320 antigen	Cd320	0.7	0.014259146	0.058339791
1381084_at	Transcribed locus	---	0.8	0.014265683	0.058339791
1367955_at	RAB4B, member RAS oncogene family	Rab4b	0.6	0.014291103	0.058429049
1380024_at	Transcribed locus	---	0.5	0.014356443	0.058637888
1380308_at	---	---	0.7	0.014396501	0.058786906
1398415_at	Transcribed locus	---	0.4	0.014408105	0.058805088
1391639_at	CDNA clone IMAGE:7313169	---	0.7	0.014433646	0.058880113
1369696_at	Ras-related GTP binding B	RragB	0.6	0.014439087	0.058887703
1388428_at	histidyl tRNA synthetase 2 (predicted)	Hars2_predicted	0.7	0.014447428	0.058907115
1376678_at	similar to amyloid beta (A4) precursor protein-binding, family B, member 2 (predicted)	RGD1562438_predicted	0.7	0.014463066	0.05895626
1378400_at	similar to RIKEN cDNA 4632404H22 (predicted)	RGD1560129_predicted	0.6	0.01448576	0.059003614
1375855_at	ependymin related protein 2 (zebrafish)	Epdr2	0.6	0.014489383	0.059003614
1369371_a_at	gamma-aminobutyric acid (GABA) B receptor 1	Gabbr1	0.4	0.014495406	0.059003614
1374045_at	component of oligomeric golgi complex 8 (predicted)	Cog8_predicted	0.6	0.014538319	0.059116505
1383327_at	---	---	0.6	0.014558199	0.059182712
1392663_at	Transcribed locus, strongly similar to XP_001065986.1 hypothetical protein [Rattus norvegicus]	---	0.6	0.014599379	0.059320797
1373910_at	Transcribed locus	---	0.7	0.014641868	0.059464062
1381358_at	similar to AP2 associated kinase 1 (predicted)	RGD1563580_predicted	0.8	0.014677419	0.059579022
1370419_a_at	SH3-domain kinase binding protein 1	Sh3kbp1	0.8	0.014705546	0.059667889
1383182_at	serine/arginine-rich protein specific kinase 1	Srpk1	0.8	0.014713443	0.059667889
1384444_at	---	---	0.6	0.014713461	0.059667889
1372920_at	similar to Proline oxidase, mitochondrial precursor (Proline dehydrogenase)	LOC680409 /// LOC682565	0.7	0.014713823	0.059667889
1389712_at	early B-cell factor 3 (predicted)	Ebf3_predicted	0.6	0.014749737	0.059778095
1389139_at	similar to Ttc15 protein (predicted)	RGD1566054_predicted	0.7	0.014751902	0.059778095
1383864_at	Transcribed locus	---	0.6	0.014785347	0.059898864
1393564_at	similar to Ubiquitin ligase protein DZIP3 (DAZ-interacting protein 3 homolog) (predicted)	RGD1563278_predicted	0.7	0.014801196	0.059948306
1391928_at	golgi associated PDZ and coiled-coil motif containing (predicted)	Gopc_predicted	0.7	0.014816073	0.059979022
1376141_at	Transcribed locus	---	0.4	0.014820085	0.059980501

1383066_at	---	---	0.4	0.014842661	0.060057093
1389341_at	plexin D1 (predicted)	Plxnd1_predicted	0.8	0.014922795	0.060247945
1384410_at	Sorting nexin 12 (predicted)	Snx12_predicted	0.7	0.014935881	0.060279935
1393695_at	Tudor domain containing 3	Tdrd3	0.7	0.014997545	0.060490344
1369391_at	glutamate receptor, metabotropic 8	Grm8	0.5	0.01500323	0.060498437
1374090_at	similar to DKFZP547E1010 protein	LOC361990	0.4	0.015022089	0.060525868
1378315_at	Transcribed locus	---	0.5	0.015032112	0.060525868
1396202_at	Transcribed locus	---	0.7	0.015072697	0.060674431
1376753_at	Fucose-1-phosphate guanylyltransferase	Fpgt	0.6	0.015112384	0.060804426
1383262_at	---	---	0.7	0.015170734	0.06097953
1396952_at	Transcribed locus	---	0.6	0.015192862	0.061023737
1398371_at	similar to RIKEN cDNA 1700034M03 gene	RGD1304751	0.7	0.01520887	0.061058212
1371534_at	solute carrier family 25 (mitochondrial carrier, glutamate), member 22	Slc25a22	0.8	0.015258775	0.061228673
1389794_at	Transcribed locus	---	0.7	0.01530769	0.061394998
1373215_at	active BCR-related gene (predicted)	Abr_predicted	0.6	0.015334892	0.061474116
1370834_at	heparan sulfate (glucosamine) 3-O-sulfotransferase 1	Hs3st1	0.7	0.015342779	0.061490748
1396330_at	Transcribed locus	---	0.8	0.01536024	0.061541845
1390794_at	similar to peptide deformylase-like protein	LOC686957 /// LOC690214	0.7	0.015369729	0.061553761
1368619_at	DEAD (Asp-Glu-Ala-Asp) box polypeptide 25	Ddx25	0.6	0.015410343	0.061641742
1368539_at	sodium channel, voltage-gated, type IX, alpha	Scn9a	0.8	0.015444354	0.061762395
1379031_at	Transcribed locus	---	0.4	0.01546672	0.061836802
1376630_at	---	---	0.6	0.015517293	0.06202392
1371625_at	brain glycogen phosphorylase	Pygb	0.5	0.015568057	0.062181496
1392835_at	---	---	0.8	0.015583812	0.062229312
1381807_at	Transcribed locus	---	0.6	0.015632847	0.062374082
1387904_at	inositol hexaphosphate kinase 1	Ihpk1	0.6	0.015637721	0.062374082
1383124_at	---	---	0.7	0.015678943	0.06251812
1377751_at	Transcribed locus	---	0.8	0.015694004	0.062539676
1374577_at	transforming growth factor beta regulated gene 4	Tbrg4	0.7	0.015695755	0.062539676
1389452_at	zinc finger, DHHC domain containing 8	Zdhhc8	0.7	0.015714325	0.062598503
1377707_at	solute carrier family 35, member B4 (predicted)	Slc35b4_predicted	0.4	0.01573377	0.062620792
1395703_at	Transcribed locus	---	0.3	0.015744526	0.062627801
1390876_at	similar to KIAA0377-like protein (predicted)	RGD1311552_predicted	0.8	0.01577761	0.062744229
1389044_at	similar to golgi-specific brefeldin A-resistance guanine nucleotide exchange factor 1 (predicted)	RGD1307160_predicted	0.5	0.015931103	0.06323233
1372489_at	sarcolemma associated protein (predicted)	Slmap_predicted	0.8	0.015936328	0.063237809
1375448_at	Transcribed locus	---	0.7	0.015946404	0.063262529
1368902_at	p21 (CDKN1A)-activated kinase 3	Pak3	0.7	0.016024013	0.063460875
1370129_at	meningioma expressed antigen 5 (hyaluronidase)	Mgea5	0.7	0.016043455	0.063509686
1371886_at	carnitine acetyltransferase	Crat	0.8	0.016047986	0.063512337
1371563_at	regulator of chromosome condensation 2 (predicted)	Rcc2_predicted	0.7	0.016116897	0.063723731
1389591_at	similar to HS1 binding protein 3 (predicted)	RGD1311331_predicted	0.7	0.016124659	0.063723786
1390871_at	transcription elongation regulator 1 (CA150) (predicted)	Tcerg1_predicted	0.6	0.016160603	0.063789202
1377789_at	Transcribed locus	---	0.5	0.016175783	0.063833799
1372104_at	---	---	0.7	0.016184653	0.063853483
1386983_at	hydroxymethylbilane synthase	Hmbs	0.6	0.01619636	0.063854403
1397262_at	Transcribed locus	---	0.7	0.016244584	0.063997823
1370831_at	monoglyceride lipase	Mgll	0.7	0.016272471	0.064053944
1371593_at	Proliferating cell nuclear antigen	Pcna	0.4	0.016274406	0.064053944
1390179_at	ankyrin repeat domain 52 (predicted)	Ankrd52_predicted	0.7	0.016300388	0.064140858
1373976_at	similar to 8430411H09Rik protein (predicted)	RGD1563990_predicted	0.7	0.016334066	0.064242637
1392295_a_at	---	---	0.7	0.016341173	0.064255229
1388603_a_at	HESB like domain containing 2	Hbld2	0.5	0.016362276	0.064284549
1367866_at	fibulin 5	Fbln5	0.4	0.016374344	0.064284549
1388623_at	EH-domain containing 1	Ehd1	0.7	0.016378443	0.064284549
1395665_at	Transcribed locus	---	0.7	0.01638543	0.064284549

1382219_at	similar to RIKEN cDNA 1700108L22	RGD1307509	0.7	0.016393179	0.064284549
1381627_at	syntaphilin (predicted)	Snph_predicted	0.7	0.016402657	0.064284549
1388165_at	similar to Protein KIAA1688	LOC500901	0.7	0.016407962	0.064287319
1374217_at	similar to chromosome 16 open reading frame 5	RGD1310686	0.5	0.016417945	0.064311111
1368772_at	solute carrier family 4, member 3	Slc4a3	0.8	0.016485889	0.064546512
1371334_at	integral membrane protein 2C	Itm2c	0.7	0.016523953	0.064656133
1385819_at	misato homolog 1 (Drosophila) (predicted)	Msto1_predicted	0.6	0.016551305	0.064710212
1399146_at	transmembrane protein 15 (predicted)	Tmem15_predicted	0.7	0.016621022	0.064936477
1390912_at	hypothetical protein LOC678810 /// hypothetical protein LOC685448	LOC678810 /// LOC685448	0.5	0.016674272	0.065112926
1398658_at	Similar to RUN and FYVE domain-containing 2	LOC690777	0.8	0.016678062	0.065112926
1383694_at	similar to RIKEN cDNA 1110061N23 (predicted)	RGD1565957_predicted	0.6	0.016684886	0.065119714
1374814_at	---	---	0.5	0.016723274	0.065222424
1398368_at	Transcribed locus	---	0.6	0.016723965	0.065222424
1385577_at	---	---	0.7	0.016728998	0.065222424
1395265_at	---	---	0.5	0.016731512	0.065222424
1372118_at	HMP19 protein	MGC125201	0.5	0.016749529	0.065252546
1370029_at	C-terminal binding protein 1	Ctbp1	0.7	0.016772478	0.065287709
1387073_at	synaptosomal-associated protein 25	Snap25	0.7	0.016836395	0.065451434
1392711_at	Transcribed locus	---	0.7	0.016855151	0.065508869
1380712_at	Transcribed locus	---	0.7	0.016863931	0.06551203
1377812_a_at	transmembrane protein 24	Tmem24	0.7	0.016916902	0.065655781
1372537_at	---	---	0.7	0.016943685	0.065713213
1395808_at	---	---	0.7	0.016954099	0.065738101
1373198_at	similar to RIKEN cDNA 2810451A06	RGD1311098	0.6	0.016964843	0.065764258
1388536_at	HLA-B associated transcript 2	Bat2	0.6	0.016979845	0.065806902
1385340_at	Exostoses (multiple)-like 2	Extl2	0.7	0.01698447	0.065809319
1393429_at	Transcribed locus	---	0.8	0.017000022	0.065854065
1373541_at	Rho guanine nucleotide exchange factor (GEF) 17 (predicted)	Arhgef17_predicted	0.8	0.01702785	0.065946333
1377145_at	similar to monogenic, audiogenic seizure susceptibility 1	LOC362068	0.7	0.017040475	0.065964165
1370994_at	huntingtin interacting protein 1 related	Hip1r	0.8	0.017101394	0.066184407
1367746_a_at	flotillin 2	Flot2	0.4	0.017108292	0.066195528
1368417_at	synaptotagmin V	Syt5	0.7	0.017131526	0.066269833
1369627_at	synaptic vesicle glycoprotein 2b	Sv2b	0.6	0.017150161	0.066279578
1379333_at	armadillo repeat containing, X-linked 6	Armcx6	0.8	0.017150163	0.066279578
1375049_at	---	---	0.6	0.017177201	0.066352891
1367907_a_at	clathrin, light polypeptide (Lcb)	Cltb	0.4	0.017201525	0.066415653
1382174_at	Coxsackie virus and adenovirus receptor-like 1	Cxadrl1	0.7	0.017217368	0.066461221
1393149_at	protocadherin alpha 4 /// protocadherin alpha 13 /// protocadherin alpha 10 /// protocadherin alpha 12 /// protocadherin alpha 3 /// protocadherin alpha 8 /// protocadherin alpha 1 /// protocadherin alpha 2 /// protocadherin alpha 5 /// protocadherin alpha 6 /// protocadherin alpha 7 /// protocadherin alpha 9 /// protocadherin alpha subfamily C, 1 /// protocadherin alpha subfamily C, 2 /// protocadherin alpha 11	Pcdha1 /// Pcdha10 /// Pcdha11 /// Pcdha12 /// Pcdha13 /// Pcdha2 /// Pcdha3 /// Pcdha4 /// Pcdha5 /// Pcdha6 /// Pcdha7 /// Pcdha8 /// Pcdha9 /// Pcdhac1 /// Pcdhac2	0.8	0.017232573	0.066488708
1383972_at	---	---	0.7	0.01724749	0.066513152
1394450_at	Transcribed locus	---	0.8	0.01725104	0.066513152
1373603_at	similar to RIKEN cDNA 0610007P22 (predicted)	RGD1565744_predicted	0.5	0.017307434	0.066714947
1383646_at	Transcribed locus	---	0.8	0.017351936	0.066870816
1374602_at	testis-specific protein, Y-encoded-like	Tspyl	0.6	0.01741274	0.06704231
1389910_at	similar to chromosome 20 open reading frame 30; HSPC274 protein	RGD1307399	0.7	0.017488557	0.067271232
1383515_at	Transcribed locus	---	0.7	0.017601535	0.067610939
1384927_at	tubulin, beta 4	Tubb4	0.7	0.017617024	0.067654639

1389437_at	sal-like 2 (Drosophila) (predicted)	Sall2_predicted	0.6	0.017663881	0.067787102
1378046_at	fucokinase (predicted)	Fuk_predicted	0.8	0.017676929	0.067805532
1367519_at	oxysterol binding protein-like 2	Osbpl2	0.8	0.017705433	0.067883207
1375692_at	Transcribed locus	---	0.8	0.017731278	0.067966457
1379491_at	melanocortin 2 receptor accessory protein (predicted)	Mrap_predicted	0.6	0.017746116	0.068007481
1387910_at	erythrocyte protein band 4.1-like 1	Epb4.1l1	0.6	0.017841492	0.068341141
1369042_at	phosphatidylinositol glycan, class M	Pigm	0.5	0.017876108	0.068408718
1391144_at	Transcribed locus	---	0.7	0.017882313	0.068408718
1377917_at	Transcribed locus, weakly similar to XP_001074405.1 similar to zinc finger protein 709 [Rattus norvegicus]	---	0.6	0.017883359	0.068408718
1372712_at	tetratricopeptide repeat domain 1	Ttc1	0.8	0.017884089	0.068408718
1372292_at	similar to Serine/threonine protein kinase 24 (predicted)	RGD1561742_predicted	0.6	0.017901913	0.068420423
1395914_at	---	---	0.3	0.017907947	0.068420423
1377633_a_at	Transcribed locus	---	0.5	0.017922395	0.068443823
1390986_at	Transcribed locus	---	0.7	0.017930473	0.06845878
1384941_at	similar to Shb-like adapter protein, Shf - human (predicted)	RGD1562705_predicted	0.7	0.017941584	0.068485304
1387757_at	LMBR1 domain containing 1	Lmbrd1	0.5	0.017951701	0.068508022
1378021_at	Transcribed locus	---	0.8	0.017984739	0.068602268
1384471_at	AP1 gamma subunit binding protein 1	Ap1gbp1	0.8	0.018008775	0.068659229
1370819_at	casein kinase 1, delta	Csnk1d	0.7	0.018061128	0.068773766
1367611_at	transketolase	Tkt	0.6	0.018100641	0.068900564
1368858_at	UDP galactosyltransferase 8	Ugt8	0.4	0.018134315	0.068996811
1368322_at	superoxide dismutase 3, extracellular	Sod3	0.6	0.018169729	0.069106659
1391702_at	zinc finger protein 446 (predicted)	Zfp446_predicted	0.7	0.018171589	0.069106659
1388322_at	eukaryotic translation initiation factor 4 gamma, 1	Eif4g1	0.5	0.01824561	0.069356089
1376459_at	similar to AP2 associated kinase 1 (predicted)	RGD1563580_predicted	0.8	0.018272712	0.06942957
1388938_at	ubiquitin specific protease 5 (isopeptidase T) (predicted)	Usp5_predicted	0.7	0.018354523	0.069620156
1399103_at	hypothetical protein LOC681972 /// hypothetical protein LOC685634	LOC681972 /// LOC685634	0.7	0.018360652	0.069620156
1372426_at	ADAMTS-like 4	Adamtsl4	0.7	0.018400963	0.069733534
1384915_at	intraflagellar transport 88 homolog (Chlamydomonas) (predicted)	Ift88_predicted	0.7	0.01840426	0.069733534
1369176_at	solute carrier family 36 (proton/amino acid symporter), member 1	Slc36a1	0.7	0.018439326	0.069850309
1370906_at	branched chain keto acid dehydrogenase E1, beta polypeptide	Bckdhb	0.6	0.018468562	0.069914922
1368272_at	glutamate oxaloacetate transaminase 1	Got1	0.4	0.018469135	0.069914922
1384002_at	similar to Potential phospholipid-transporting ATPase IF (ATPase class I type 11B) (ATPase IR)	LOC361929	0.5	0.018514629	0.070033329
1389463_at	protein kinase, cAMP dependent regulatory, type I, beta	Prkar1b	0.7	0.018526268	0.070033329
1399136_at	LEM domain containing 2	Lemd2	0.7	0.018600397	0.070259926
1377852_at	Transcribed locus	---	0.5	0.01860299	0.070259926
1367517_at	Transcribed locus	---	0.6	0.018623128	0.070319838
1393152_at	similar to Mut protein (predicted) /// similar to Methylmalonyl-CoA mutase, mitochondrial precursor (MCM) (Methylmalonyl-CoA isomerase)	LOC688517 /// RGD1564912_predicted	0.6	0.018628243	0.070323006
1388039_a_at	gamma-aminobutyric acid (GABA) B receptor 1	Gabbr1	0.4	0.018634141	0.07032913
1368225_at	exocyst complex component 2	Exoc2	0.8	0.01864101	0.070338917
1372540_at	ubiquitin-like domain containing CTD phosphatase 1	Ublcp1	0.8	0.018649	0.070352923
1380086_at	Transcribed locus	---	0.7	0.018719243	0.070507459
1383162_at	---	---	0.8	0.01871997	0.070507459
1388815_at	SAPS domain family, member 1 (predicted)	Saps1_predicted	0.7	0.018787484	0.070745543
1369163_at	glutamate receptor, ionotropic, kainate 4	Grik4	0.6	0.018808191	0.070789459
1395167_at	Transcribed locus	---	0.7	0.018824773	0.070821105
1371840_at	endothelial differentiation sphingolipid G-protein-coupled receptor 1	Edg1	0.7	0.018851585	0.070857147
1373469_at	Transcribed locus	---	0.7	0.0188839	0.070925044

1371008_at	peptidase (mitochondrial processing) alpha	Pmpca	0.6	0.018900684	0.070925044
1383025_at	---	---	0.7	0.018911032	0.070925044
1387020_at	cytochrome P450, subfamily 51	Cyp51	0.7	0.018912677	0.070925044
1387599_a_at	NAD(P)H dehydrogenase, quinone 1	Nqo1	0.6	0.01891277	0.070925044
1393407_at	F-box and WD-40 domain protein 2 (predicted)	Fbxw2_predicted	0.5	0.019002248	0.071195666
1386012_at	Transcribed locus	---	0.3	0.019006649	0.071195936
1387836_at	prenylated SNARE protein	Ykt6	0.6	0.019013089	0.071203846
1391269_at	---	---	0.6	0.019025501	0.071218949
1381792_at	mitochondrial ribosomal protein S15	Mrps15	0.8	0.019029549	0.071218949
1383353_at	Transcribed locus	---	0.7	0.019066675	0.071323327
1397190_at	Transcribed locus	---	0.8	0.019072378	0.071328437
1390977_at	suppression of tumorigenicity 7-like	St7l	0.6	0.019120222	0.071458627
1382150_at	similar to RIKEN cDNA 1200007B05 gene	RGD1305072	0.7	0.019129822	0.071478261
1389154_at	lipoic acid synthetase	Lias	0.8	0.019186644	0.071674295
1373903_at	RCSD domain containing 1 (predicted)	Rcsd1_predicted	0.7	0.019195899	0.071692586
1377195_at	ATPase, Na+/K+ transporting, beta 3 polypeptide	Atp1b3	0.7	0.019239857	0.0718139
1372089_at	Transcribed locus, strongly similar to XP_001061027.1 similar to Heat shock factor protein 1 (HSF 1) (Heat shock transcription factor 1) (HSTF 1) [Rattus norvegicus]	---	0.7	0.019296594	0.071970606
1378976_x_at	Exosome component 4 (predicted)	Exosc4_predicted	0.8	0.019309769	0.07198342
1374475_at	abhydrolase domain containing 1	Abhd1	0.6	0.01935308	0.072099538
1374022_at	Transcribed locus	---	0.6	0.019374692	0.072163707
1372804_at	MMR_HSR1 domain containing protein RGD1359460	RGD1359460	0.6	0.0193915	0.072193421
1389493_at	ankyrin repeat and BTB (POZ) domain containing 1	Abtb1	0.6	0.01942641	0.07227726
1381019_x_at	development and differentiation enhancing (predicted)	Ddef1_predicted	0.8	0.019445379	0.072284537
1398950_at	---	---	0.7	0.019446685	0.072284537
1372487_at	hypothetical protein LOC619573	LOC619573	0.7	0.019469625	0.072320773
1370278_at	ATP synthase, H+ transporting, mitochondrial F1 complex, delta subunit	Atp5d	0.5	0.019483117	0.072338219
1375533_at	vestigial like 4 (Drosophila)	Vgll4	0.8	0.019507629	0.072380212
1391684_at	transmembrane protein 14A (predicted)	Tmem14a_predicted	0.7	0.019516622	0.072380924
1391425_at	NCK interacting protein with SH3 domain (predicted)	Nckipsd_predicted	0.7	0.019535399	0.07243423
1373618_at	cAMP responsive element binding protein-like 2	Crebl2	0.5	0.01953992	0.072434663
1370691_a_at	thyroid hormone receptor alpha	Thra	0.7	0.01956873	0.072501148
1383622_at	O-acyltransferase (membrane bound) domain containing 5	Oact5	0.8	0.019572587	0.072501148
1392984_at	copine III (predicted)	Cpne3_predicted	0.8	0.019579727	0.072501148
1367548_at	similar to FLJ00052 protein (predicted)	RGD1305986_predicted	0.7	0.019579894	0.072501148
1384491_at	similar to aldehyde oxidase structural homolog 2	RGD1311975	0.7	0.019605275	0.072578788
1383700_at	similar to D15Wsu75e protein	RGD1305776	0.8	0.019631728	0.072644017
1378292_at	Transcribed locus	---	0.8	0.019654593	0.072679573
1388750_at	transferrin receptor	Tfrc	0.6	0.019699717	0.072764642
1380170_at	GABA(A) receptor-associated protein like 2	Gabarapl2	0.6	0.019719178	0.072803649
1373018_at	similar to hypothetical protein FLJ14800 (predicted)	RGD1561500_predicted	0.5	0.019719404	0.072803649
1373525_at	Transcribed locus	---	0.5	0.019727983	0.072803649
1384413_at	similar to solute carrier family 35, member A5 (predicted)	RGD1564361_predicted	0.7	0.019733661	0.072808267
1381063_at	adenylate cyclase 9 (predicted)	Adcy9_predicted	0.8	0.019746505	0.072818822
1374736_at	Transcribed locus	---	0.7	0.019747348	0.072818822
1377431_at	similar to CG13901-PA	RGD1307648	0.6	0.019777905	0.072899074
1385049_at	Transcribed locus	---	0.6	0.019781287	0.072899074
1388487_at	adducin 1 (alpha)	Add1	0.8	0.019806134	0.072961095
1380120_at	Microtubule-associated protein 7 (predicted)	Mtap7_predicted	0.7	0.019824187	0.072994902
1393719_at	Transcribed locus	---	0.5	0.019841301	0.073041566
1389297_at	ERO1-like (S. cerevisiae)	Ero1l	0.7	0.019864061	0.073108991
1384284_at	Transcribed locus	---	0.8	0.019885628	0.073155626
1371728_at	similar to ubiquitin protein ligase E3B	LOC687633	0.6	0.019921971	0.073256557

1370085_at	RAS p21 protein activator 1	Rasa1	0.8	0.019959944	0.07329856
1391308_at	Similar to vacuolar protein sorting 13C protein (predicted)	RGD1560364_predicted	0.8	0.019960132	0.07329856
1372982_at	protein phosphatase 3, regulatory subunit B, alpha isoform (calcineurin B, type I)	Ppp3r1	0.6	0.02002205	0.073493122
1386551_at	Wolf-Hirschhorn syndrome candidate 1 (predicted)	Whsc1_predicted	0.6	0.020039071	0.073518654
1389656_at	similar to Tetraspanin-15 (Tspan-15) (Transmembrane 4 superfamily member 15) (Tetraspan NET-7)	LOC679462	0.4	0.020050379	0.073523058
1387046_at	selective LIM binding factor, rat homolog	Slb	0.5	0.020058023	0.073523058
1370181_at	RAB4A, member RAS oncogene family	Rab4a	0.5	0.020063876	0.073523058
1384379_at	Syntaxin 12	Stx12	0.7	0.020070436	0.073523058
1395081_at	similar to nuclear receptor coactivator 7 (predicted)	RGD1566426_predicted	0.6	0.020147607	0.073730203
1391158_a_at	ataxin 2 (predicted)	Atxn2_predicted	0.8	0.020147908	0.073730203
1381220_at	activin receptor IIA	Acvr2a	0.8	0.020149396	0.073730203
1374678_at	sema domain, immunoglobulin domain (Ig), transmembrane domain (TM) and short cytoplasmic domain, (semaphorin) 4B	Sema4b	0.8	0.020167641	0.073780551
1392525_at	Prostaglandin F receptor	Ptgfr	0.6	0.0201794	0.073797833
1378245_at	similar to 6430514L14Rik protein (predicted)	RGD1311958_predicted	0.6	0.020181338	0.073797833
1395102_at	potassium channel, subfamily T, member 1	Kcnt1	0.6	0.020238892	0.073958963
1393054_at	dedicator of cyto-kinesis 3 (predicted)	Dock3_predicted	0.7	0.020272046	0.074047216
1390036_at	solute carrier family 16 (monocarboxylic acid transporters), member 6	Slc16a6	0.6	0.020282403	0.074068599
1369041_at	neuroligin 1	Nlgn1	0.7	0.02029002	0.074073055
1369066_at	MAP-kinase activating death domain	Madd	0.5	0.020314867	0.074121324
1376378_at	Putative GTP-binding protein	LOC293589	0.7	0.020363373	0.074248895
1391306_at	Exosome component 4 (predicted)	Exosc4_predicted	0.8	0.020372968	0.074267415
1390309_a_at	Transcribed locus	---	0.8	0.020399116	0.074329789
1372127_at	ubiquitin-associated protein 2 (predicted)	Ubap2_predicted	0.6	0.020427751	0.07441764
1371666_at	Transcribed locus	---	0.8	0.020441486	0.074451186
1376921_at	FAD-dependent oxidoreductase domain containing 1 (predicted)	Foxred1_predicted	0.7	0.020456279	0.074457056
1379817_at	purine-rich element binding protein G (predicted)	Purg_predicted	0.7	0.020462314	0.074457056
1395821_at	core binding factor beta	Cbfb	0.3	0.020464252	0.074457056
1395151_at	Transcribed locus	---	0.7	0.020467746	0.074457056
1392405_at	transcription factor Dp 2 (predicted)	Tcfdp2_predicted	0.8	0.020519	0.074601351
1389256_at	Transcribed locus	---	0.7	0.020556992	0.074673454
1385543_at	Transcribed locus	---	0.7	0.02058853	0.074725507
1391005_at	F-box and leucine-rich repeat protein 4 (predicted)	Fbxl4_predicted	0.8	0.020606096	0.074752771
1371711_at	Transcribed locus	---	0.7	0.020633312	0.074834997
1376715_at	calcium binding atopy-related autoantigen 1	Cbara1	0.6	0.020752609	0.075234497
1398299_at	Rho guanine nucleotide exchange factor (GEF) 11	Arhgef11	0.8	0.020761181	0.075248987
1389309_at	sno, strawberry notch homolog 1 (Drosophila)	Sbno1	0.7	0.020824999	0.075430429
1385167_at	exportin 5 (predicted)	Xpo5_predicted	0.8	0.020852727	0.075514235
1384270_at	Transcribed locus	---	0.6	0.020891643	0.075638509
1367845_at	neurofilament 3, medium	Nef3	0.7	0.020903277	0.075663975
1375339_at	enthoprotin	Enth	0.7	0.020911786	0.075678119
1394604_at	Neural precursor cell expressed, developmentally down-regulated 4-like	Nedd4l	0.8	0.02091916	0.075688152
1374327_at	Transcribed locus	---	0.7	0.020936669	0.075734841
1367959_a_at	sodium channel, voltage-gated, type I, beta	Scn1b	0.5	0.020952995	0.075777236
1391246_at	dystrobrevin alpha (predicted)	Dtna_predicted	0.7	0.021036487	0.076029033
1383575_at	catenin (cadherin-associated protein), delta 2	Ctnnd2	0.7	0.021043853	0.07603895
1390020_at	---	---	0.6	0.02107498	0.076134695
1368387_at	3-hydroxybutyrate dehydrogenase, type 1	Bdh1	0.7	0.021147518	0.076310277
1372498_at	cytokine induced apoptosis inhibitor 1	Ciapin1	0.7	0.021156059	0.076310277
1376718_at	similar to CG9117-PA	RGD1306932	0.6	0.021189665	0.076399972
1398721_at	similar to fibronectin type 3 and SPRY domain-containing protein	LOC301506	0.6	0.02121654	0.07646136
1374608_at	similar to TatD DNase domain containing 2	LOC500295	0.7	0.02123688	0.076501139
1373188_at	sodium channel, voltage-gated, type IV, beta	Scn4b	0.8	0.02132054	0.076763701

1379660_at	Transcribed locus	---	0.7	0.021332946	0.076763701
1374287_at	paladin	Pald	0.8	0.021335096	0.076763701
1382388_at	similar to hypothetical protein MGC27699 (predicted)	RGD1305013_predicted	0.8	0.021356696	0.076814986
1390486_at	ubiquitin specific protease 32 (predicted)	Usp32_predicted	0.6	0.021368182	0.076839496
1371402_at	ATPase, H transporting, lysosomal V1 subunit B2	Atp6v1b2	0.6	0.021398767	0.076899044
1369135_at	synaptotagmin XI	Syt11	0.7	0.021410542	0.076909474
1369453_at	Epsin 1	Epn1	0.7	0.021411021	0.076909474
1386828_at	RUN domain containing 1 (predicted)	Rundc1_predicted	0.7	0.021486221	0.077095411
1398458_at	similar to protein kinase, lysine deficient 1; kinase deficient protein (predicted)	RGD1307284_predicted	0.8	0.021495369	0.077111413
1371103_at	RAB6A, member RAS oncogene family	Rab6a	0.7	0.021566269	0.077281481
1383270_at	similar to hypothetical protein	RGD1310571	0.8	0.021645166	0.077462943
1388570_at	similar to RIKEN cDNA 2310005O14	LOC498909	0.8	0.021651368	0.077467036
1368515_at	erythrocyte protein band 4.1-like 3	Epb4.1l3	0.7	0.021655729	0.077467036
1393290_at	similar to myocyte enhancer factor 2C	LOC309957	0.7	0.021664732	0.077482391
1384869_at	abhydrolase domain containing 7 (predicted)	Abhd7_predicted	0.6	0.021701743	0.077597885
1392507_at	Transcribed locus	---	0.7	0.021764848	0.077755913
1382995_at	Neuropilin 2	Nrp2	0.7	0.021812456	0.077866906
1398376_at	---	---	0.7	0.021854519	0.07789178
1375580_at	similar to Dipeptidyl peptidase 9 (Dipeptidyl peptidase IX) (DP9) (Dipeptidyl peptidase-like protein 9) (DPLP9)	LOC301130	0.7	0.021854971	0.07789178
1391559_at	TLC domain containing 1	Tlcd1	0.6	0.021865806	0.077901662
1398910_at	STIP1 homology and U-Box containing protein 1	Stub1	0.6	0.021897144	0.077974502
1379480_at	diacylglycerol kinase, iota /// similar to diacylglycerol kinase iota-1 /// similar to Diacylglycerol kinase iota (Diglyceride kinase iota) (DGK-iota) (DAG kinase iota)	Dgki /// LOC500082 /// LOC688705	0.8	0.021933486	0.078070109
1382787_at	kinesin family member 5A	Kif5a	0.5	0.021938629	0.078071522
1376894_at	pallidin	Pldn	0.5	0.021955534	0.078106477
1386963_at	thyroid hormone receptor interactor 10	Trip10	0.8	0.021962698	0.078106477
1388924_at	angiopoietin-like 4	Angptl4	0.7	0.021992214	0.078179523
1393441_at	similar to cyclin-dependent kinase inhibitor 2D	Cdkn2d	0.5	0.022043963	0.078297781
1377786_at	Transcribed locus	---	0.7	0.022052417	0.078297781
1369571_at	golgi phosphoprotein 3	Golph3	0.6	0.02207099	0.078322252
1388384_at	Dynein light chain LC8-type 1	Dynll1	0.8	0.022084701	0.078337105
1389386_at	---	---	0.8	0.022095763	0.078359445
1378965_at	Transcribed locus	---	0.7	0.022212807	0.078704529
1397669_at	similar to Fgfr1 oncogene partner	LOC683722	0.6	0.022230306	0.078717776
1375443_at	similar to gene trap ROSA b-geo 22	LOC687166 /// LOC691093	0.6	0.02233677	0.079060725
1385075_at	coiled-coil domain containing 32	Ccdc32	0.6	0.022343907	0.079068971
1372746_at	similar to RIKEN cDNA 2810428I15 (predicted)	RGD1566239_predicted	0.4	0.022403235	0.079210749
1395820_at	similar to RIKEN cDNA A630007N06 gene (predicted)	RGD1304572_predicted	0.7	0.022427068	0.079277975
1373954_at	similar to FLJ00052 protein (predicted)	RGD1305986_predicted	0.7	0.022447071	0.079331632
1389382_at	makorin, ring finger protein, 2	Mkrn2	0.7	0.022474925	0.079398371
1388642_at	etoposide induced 2.4 mRNA	Ei24	0.6	0.022481912	0.079403583
1371663_at	similar to expressed sequence AW556797 (predicted)	RGD1305138_predicted	0.6	0.022519468	0.079514959
1399019_at	abhydrolase domain containing 4 (predicted)	Abhd4_predicted	0.7	0.022559418	0.079591872
1372719_at	---	---	0.7	0.022596613	0.079634717
1391045_at	diacylglycerol O-acyltransferase homolog 2 (mouse)	Dgat2	0.8	0.022600612	0.079634717
1391658_at	archain 1	Arcn1	0.6	0.02261935	0.079683672
1385173_at	early B-cell factor 3 (predicted)	Ebf3_predicted	0.7	0.022635025	0.079721818
1380960_at	coronin, actin binding protein 2A	Coro2a	0.7	0.02264595	0.07973193
1374431_at	similar to ATPase, H+ transporting, V1 subunit A, isoform 1	LOC685232	0.8	0.022656427	0.07973193
1381686_at	similar to CG12753-PA (predicted)	RGD1305773_predicted	0.7	0.022657286	0.07973193
1397561_at	Transcribed locus	---	0.7	0.022710134	0.079900808
1372826_at	---	---	0.7	0.022756811	0.080013686

1383086_at	Transcribed locus	---	0.7	0.022904981	0.080380016
1396092_at	NFKB inhibitor interacting Ras-like protein 1 (predicted)	Nkiras1_predicted	0.4	0.022914887	0.080381691
1384408_at	Transcribed locus	---	0.6	0.022915233	0.080381691
1389381_at	sequestosome 1	Sqstm1	0.5	0.02299317	0.080552003
1393274_at	similar to PRO1853 homolog	RGD1311578	0.7	0.023012403	0.080602214
1368109_at	ST3 beta-galactoside alpha-2,3-sialyltransferase 5	St3gal5	0.6	0.023017546	0.080603064
1368058_at	scaffold attachment factor B	Safb	0.7	0.023050287	0.080700538
1391805_at	similar to KIAA1900 protein (predicted)	RGD1310364_predicted	0.8	0.023081807	0.080793692
1387437_at	F-box only protein 2	Fbxo2	0.4	0.023095667	0.080825006
1375672_at	---	---	0.7	0.023112678	0.080867333
1393541_at	Transcribed locus	---	0.7	0.023198353	0.081107783
1392829_at	similar to cleavage stimulation factor, 3 pre-RNA subunit 2	LOC683927	0.8	0.023210988	0.081107783
1369689_at	N-ethylmaleimide sensitive fusion protein	Nsf	0.3	0.023217332	0.081112717
1377091_at	NG5 protein	Ng5	0.7	0.023232069	0.081146967
1390440_at	solute carrier family 9 (sodium/hydrogen exchanger), member 6 (predicted)	Slc9a6_predicted	0.7	0.023263979	0.081206692
1372447_at	---	---	0.6	0.023302426	0.081238142
1376727_at	Yip1 domain family, member 4	Yipf4	0.5	0.023304004	0.081238142
1388437_at	Transcribed locus	---	0.6	0.02333307	0.081292593
1388551_at	protocadherin gamma subfamily C, 3 /// protocadherin gamma subfamily A, 9 /// protocadherin gamma subfamily A, 11 /// protocadherin gamma subfamily A, 7 /// protocadherin gamma subfamily A, 5 /// protocadherin gamma subfamily A, 8 /// protocadherin gamma b7 /// protocadherin gamma subfamily B, 5 /// protocadherin gamma a2 /// protocadherin gamma subfamily A, 3 /// protocadherin gamma subfamily A, 10 /// protocadherin gamma subfamily A, 12 /// protocadherin gamma a1	Pcdhga1 /// Pcdhga10 /// Pcdhga11 /// Pcdhga12 /// Pcdhga2 /// Pcdhga3 /// Pcdhga5 /// Pcdhga7 /// Pcdhga8 /// Pcdhga9 /// Pcdhgb5 /// Pcdhgb7 /// Pcdhgc3	0.7	0.023358851	0.081357234
1387376_at	aldehyde oxidase 1	Aox1	0.5	0.023367043	0.081359257
1368473_at	gap junction membrane channel protein alpha 5	Gja5	0.7	0.023403438	0.081468731
1379784_at	peroxisome biogenesis factor 7	Pex7	0.6	0.02342287	0.081519123
1374777_at	hypothetical protein LOC502894	LOC502894	0.7	0.023435912	0.081547255
1370606_at	purinergic receptor P2Y, G-protein coupled 1	P2ry1	0.7	0.023459672	0.081612667
1389906_at	farnesyl diphosphate farnesyl transferase 1	Fdft1	0.6	0.023478586	0.081643928
1374512_at	similar to CG9886-like	LOC684314	0.8	0.023509405	0.081733819
1373974_at	oxysterol binding protein (predicted)	Osbp_predicted	0.7	0.023545368	0.081789697
1371881_at	zinc finger, HIT domain containing 2 (predicted)	Znhit2_predicted	0.6	0.023611466	0.081984672
1394738_at	Transcribed locus	---	0.6	0.02365211	0.082091137
1393765_at	Transcribed locus	---	0.8	0.023704036	0.082197308
1398251_a_at	calcium/calmodulin-dependent protein kinase II, beta	Camk2b	0.7	0.023707687	0.082197308
1374215_at	pleckstrin homology domain containing, family J member 1	Plekhj1	0.7	0.023727708	0.082214732
1387341_a_at	myelin basic protein	Mbp	0.6	0.02376368	0.082304699
1397555_at	---	---	0.7	0.02380929	0.082427954
1395736_at	receptor interacting protein kinase 5	Ripk5	0.5	0.023847931	0.082509627
1371674_at	kelch domain containing 3	Klhdc3	0.7	0.023878791	0.082591817
1393510_at	similar to RIKEN cDNA 5730410E15 gene (predicted)	RGD1564335_predicted	0.8	0.023881729	0.082591817
1370519_at	syntaxin binding protein 1	Stxbp1	0.8	0.023961798	0.082851304
1371980_at	ATPase family, AAA domain containing 3A	Atad3a	0.7	0.024016019	0.082969013
1374637_at	similar to hypothetical protein PRO1580 (predicted)	RGD1309263_predicted	0.7	0.024048293	0.08303879
1392773_at	proprotein convertase subtilisin/kexin type 5	Pcsk5	0.8	0.02408554	0.083121897
1367887_at	lecithin cholesterol acyltransferase	Lcat	0.6	0.024100156	0.083131787
1375574_at	golgi associated, gamma adaptin ear containing, ARF binding protein 3 (predicted)	Gga3_predicted	0.6	0.024136745	0.083211315
1393620_at	sestrin 3 (predicted)	Sesn3_predicted	0.7	0.024157699	0.083248656
1368868_at	A kinase (PRKA) anchor protein (gravin) 12	Akap12	0.6	0.024207939	0.083350794

1391725_at	Transcribed locus	---	0.8	0.024251386	0.083449122
1372923_at	peroxisomal biogenesis factor 11b	Pex11b	0.8	0.024294699	0.083554007
1392999_at	neuropilin (NRP) and tolloid (TLL)-like 2 (predicted)	Neto2_predicted	0.7	0.024297107	0.083554007
1382527_at	Transcribed locus	---	0.4	0.024308782	0.083576683
1372158_at	LRP16 protein	Lrp16	0.6	0.02435259	0.083674378
1386943_at	plasma membrane proteolipid	Pllp	0.6	0.024413493	0.083849058
1387356_at	Wolfram syndrome 1 homolog (human)	Wfs1	0.6	0.024430449	0.083872266
1368061_at	potassium voltage-gated channel, subfamily H (eag-related), member 1	Kcnh1	0.8	0.024451226	0.083918635
1369637_at	kinesin family member 3C	Kif3c	0.6	0.02445416	0.083918635
1370052_at	3-phosphoinositide dependent protein kinase-1	Pdpk1	0.7	0.024504914	0.084022687
1395412_at	GPI-anchored membrane protein 1	Gpiap1	0.5	0.024529121	0.084078886
1387966_at	asparaginase like 1	Asrgl1	0.7	0.024539712	0.084089413
1370833_at	peroxin 2	Pex2	0.6	0.024552119	0.084114403
1384437_at	SWI/SNF related, matrix associated, actin dependent regulator of chromatin, subfamily a, member 1 (predicted)	Smarca1_predicted	0.7	0.024584223	0.084206852
1387670_at	glycerol-3-phosphate dehydrogenase 2, mitochondrial	Gpd2	0.5	0.024604167	0.084257617
1387299_at	Msx-interacting-zinc finger	Miz1	0.7	0.024652076	0.084386546
1380335_at	Transcribed locus	---	0.8	0.024755449	0.084687402
1376679_at	Transcribed locus	---	0.5	0.024834284	0.084904242
1389969_at	translocase of outer mitochondrial membrane 40 homolog (yeast)	Tomm40	0.6	0.024882287	0.085033005
1373045_at	potassium channel tetramerisation domain containing 13	Kctd13	0.6	0.024944115	0.08522659
1373551_at	similar to KIAA0614 protein (predicted)	RGD1309762_predicted	0.8	0.025029281	0.08544005
1379637_at	similar to c-myc promoter binding protein (predicted)	RGD1562639_predicted	0.7	0.025083347	0.085575671
1383259_at	Transcribed locus	---	0.8	0.025163626	0.085760187
1392463_at	similar to hypothetical protein FLJ14503 (predicted)	RGD1564852_predicted	0.7	0.025172552	0.085760187
1368074_at	galactose-4-epimerase, UDP	Gale	0.6	0.025173284	0.085760187
1375289_at	---	---	0.8	0.025180734	0.085767801
1373874_at	sphingosine-1-phosphate phosphatase 1	Sgpp1	0.7	0.025259076	0.085963436
1391853_at	Transcribed locus	---	0.6	0.025295644	0.086070078
1371966_at	protein-L-isoaspartate (D-aspartate) O-methyltransferase 1	Pcmt1	0.7	0.025317386	0.08612624
1371828_at	similar to RIKEN cDNA 1500011H22	RGD1310861	0.4	0.025338742	0.086176293
1381976_at	kinesin family member 21A (predicted)	Kif21a_predicted	0.7	0.025342578	0.086176293
1383289_at	Ngg1 interacting factor 3-like 1 (S. pombe)	Nif3l1	0.7	0.025362265	0.08619851
1371726_at	Transcribed locus	---	0.8	0.025364834	0.08619851
1382028_at	similar to Peroxisomal biogenesis factor 19 (Peroxin-19) (Peroxisomal farnesylated protein) /// peroxisome biogenesis factor 19	LOC289233 /// Pex19	0.7	0.025387125	0.086238626
1380348_at	wingless related MMTV integration site 9A (predicted)	Wnt9a_predicted	0.7	0.025409787	0.086297787
1385560_at	---	---	0.7	0.025444601	0.086344709
1389448_at	septin 4	Sep-04	0.5	0.025510146	0.086531425
1390255_at	similar to mKIAA0843 protein (predicted)	RGD1565118_predicted	0.8	0.025544547	0.086630249
1379684_at	Hypothetical protein LOC681219	LOC681219	0.7	0.025611515	0.086803669
1388940_at	similar to Rho-GTPase-activating protein 25 (predicted)	RGD1562105_predicted	0.8	0.025693647	0.086992404
1383913_at	---	---	0.6	0.025707611	0.086998315
1383159_at	Transcribed locus	---	0.6	0.025716549	0.086998315
1398375_at	metastasis associated 3 (predicted)	Mta3_predicted	0.6	0.025738648	0.087037269
1370893_at	acetyl-coenzyme A carboxylase alpha	Acaca	0.8	0.025782918	0.087169053
1375182_at	Solute carrier family 3, member 1	Slc3a1	0.4	0.025799163	0.087200254
1378606_at	---	---	0.7	0.025856101	0.087344659
1372921_at	Transcribed locus	---	0.8	0.025897369	0.087412256
1379943_at	Transcribed locus	---	0.7	0.025914048	0.087450606
1371709_at	mitochondrial ribosomal protein L3 (predicted)	Mrpl3_predicted	0.7	0.025929212	0.087465891
1387199_a_at	Cdc42 guanine nucleotide exchange factor (GEF) 9	Arhgef9	0.6	0.02594944	0.087516176
1371922_at	Transcribed locus	---	0.7	0.02597347	0.087573024
1392558_at	Transcribed locus	---	0.8	0.026041294	0.087736009

1383547_at	similar to neuropathy target esterase homolog (predicted)	RGD1564611_predicted	0.8	0.026061049	0.087776875
1390311_at	Tubulin tyrosine ligase	Ttl	0.7	0.026084663	0.087810181
1377933_at	solute carrier family 39 (zinc transporter), member 3	Slc39a3	0.6	0.026102847	0.087846617
1372831_at	Transcribed locus	---	0.7	0.026106169	0.087846617
1369249_at	progressive ankylosis homolog (mouse)	Ank	0.7	0.026121557	0.087862717
1373158_at	Transcribed locus	---	0.6	0.02614335	0.087911873
1367892_at	pyruvate dehydrogenase kinase, isoenzyme 2	Pdk2	0.8	0.026202625	0.088045102
1387091_at	peptidyl arginine deiminase, type II	Padi2	0.7	0.02626387	0.088196843
1384525_at	dedicator of cytokinesis 11	Dock11	0.8	0.026361439	0.088506419
1380465_at	---	---	0.7	0.026394567	0.088545346
1379013_at	similar to mKIAA1402 protein (predicted)	RGD1306404_predicted	0.8	0.026401188	0.088549498
1368991_at	sphingomyelin phosphodiesterase 3, neutral	Smpd3	0.8	0.026431432	0.088614797
1377839_at	---	---	0.6	0.026437366	0.08861663
1388243_at	G protein-coupled receptor 176	Gpr176	0.7	0.026452354	0.088648801
1372527_at	reticulon 2 (Z-band associated protein)	Rtn2	0.6	0.026461226	0.088660469
1374807_at	similar to RIKEN cDNA 1810063B07 gene (predicted)	RGD1310877_predicted	0.8	0.026511779	0.088793677
1376240_at	similar to MYST histone acetyltransferase monocytic leukemia 4 (predicted)	RGD1566399_predicted	0.7	0.026547732	0.088895989
1398616_at	adaptor-related protein complex 1, sigma 2 subunit (predicted)	Ap1s2_predicted	0.8	0.026689443	0.08922597
1395530_at	Transcribed locus	---	0.8	0.026689675	0.08922597
1398489_at	---	---	0.6	0.026883262	0.089800142
1372287_at	myotubularin related protein 6 (predicted)	Mtmr6_predicted	0.8	0.026891475	0.089809335
1384038_at	Transcribed locus	---	0.6	0.026934528	0.08989836
1394393_at	ubiquitin specific protease 7 (herpes virus-associated)	Usp7	0.6	0.027014456	0.090092005
1372191_at	Transcribed locus	---	0.7	0.027049209	0.09017134
1387782_at	dynein light chain LC8-type 2	Dynll2	0.5	0.027075638	0.090241155
1391944_at	---	---	0.7	0.027090258	0.090271589
1394793_at	---	---	0.8	0.027111426	0.090315756
1388525_at	similar to HGFL protein	RGD1311203	0.5	0.027141714	0.090388119
1382320_at	LOC362793	RGD1307315	0.8	0.027153071	0.090407632
1377941_at	Ubiquitin specific protease 20 (predicted)	Usp20_predicted	0.7	0.027163131	0.090416693
1398351_at	Ubiquitin specific protease 7 (herpes virus-associated)	Usp7	0.8	0.027208552	0.090463047
1373844_at	protein phosphatase methylesterase 1	Ppme1	0.7	0.027208708	0.090463047
1384953_at	protein tyrosine phosphatase, receptor type, M	Ptprm	0.8	0.027213713	0.090463047
1377493_at	mitochondrial translation optimization 1 homolog (S. cerevisiae) (predicted)	Mto1_predicted	0.7	0.027248337	0.090517115
1372895_at	similar to RIKEN cDNA 5730469M10	RGD1309676	0.7	0.027303103	0.090629923
1374596_at	similar to RIKEN cDNA 1810043G02; DNA segment, Chr 10, Johns Hopkins University 13, expressed	RGD1309594	0.8	0.02730484	0.090629923
1382261_at	similar to CG8841-PA (predicted)	RGD1311422_predicted	0.7	0.027310457	0.090629923
1369027_at	alpha 1,4-galactosyltransferase	A4galt	0.5	0.027313505	0.090629923
1377149_at	---	---	0.7	0.027325643	0.09065191
1385713_at	A kinase (PRKA) anchor protein 8-like	Akap8l	0.5	0.027424159	0.090901272
1385681_at	similar to RIKEN cDNA 5430437P03	MGC94542	0.7	0.027428442	0.090901272
1376619_at	similar to protein tyrosine phosphatase, receptor type, D (predicted)	RGD1561090_predicted	0.8	0.027448409	0.090949119
1370607_a_at	neuregulin 1	Nrg1	0.7	0.02748348	0.091002001
1386960_at	solute carrier family 37 (glycerol-6-phosphate transporter), member 4	Slc37a4	0.6	0.027486499	0.091002001
1383328_x_at	---	---	0.7	0.027535062	0.091052814
1372707_at	RAB6A, member RAS oncogene family	Rab6a	0.7	0.027551842	0.091054542
1398247_at	protease, serine, 15	Prss15	0.5	0.027552192	0.091054542
1373915_at	dystrophia myotonica-protein kinase (predicted)	Dmpk_predicted	0.6	0.027558457	0.09105695
1377669_at	RAB27A, member RAS oncogene family	Rab27a	0.6	0.027585028	0.09112644
1391007_s_at	potassium inwardly rectifying channel, subfamily J, member 11	Kcnj11	0.7	0.027610938	0.091193718
1392982_at	Transcribed locus	---	0.8	0.027660344	0.091301895
1370809_at	tubulin, gamma 1	Tubg1	0.6	0.027682301	0.09133771

1389334_at	NADH dehydrogenase (ubiquinone) 1 alpha subcomplex 10 /// NADH dehydrogenase 1 alpha subcomplex 10-like protein /// hypothetical protein LOC678759	LOC316632 /// LOC678759 /// Ndufa10	0.7	0.027697999	0.091371178
1397575_at	similar to RIKEN cDNA D930015E06 /// similar to KIAA0922 protein	LOC679811 /// LOC683642	0.8	0.027796798	0.091592188
1376564_at	spectrin beta 4	Spnb4	0.7	0.027796969	0.091592188
1387940_at	eukaryotic translation initiation factor 2B, subunit 5 epsilon	Eif2b5	0.5	0.027799921	0.091592188
1388948_at	START domain containing 10	Stard10	0.6	0.027863691	0.091733785
1367704_at	adaptor-related protein complex 2, beta 1 subunit	Ap2b1	0.7	0.027925288	0.091881404
1368541_at	embigin	Emb	0.8	0.027937466	0.091903088
1383895_at	dynein light chain roadblock-type 2 (predicted)	Dynlrb2_predicted	0.7	0.027946199	0.091913434
1376946_at	Transcribed locus	---	0.8	0.027963126	0.091950719
1372925_at	sirtuin 3 (silent mating type information regulation 2, homolog) 3 (S. cerevisiae) (predicted)	Sirt3_predicted	0.7	0.027974801	0.091958242
1379932_at	putative chloride channel 4-2	Clcn4-2	0.5	0.027976596	0.091958242
1373120_at	Transcribed locus, strongly similar to NP_446127.3 associated 2 [Rattus norvegicus]	---	0.7	0.028046733	0.092119875
1391855_at	Similar to B-cell CLL/lymphoma 7A	LOC690085	0.7	0.02806972	0.092135453
1398740_at	Transcribed locus	---	0.7	0.028076315	0.092138712
1382367_at	NADH dehydrogenase (ubiquinone) 1 beta subcomplex, 7 (predicted)	Ndufb7_predicted	0.8	0.028092997	0.092175066
1374447_at	ubiquitin specific peptidase 9, X chromosome (predicted)	Usp9x_predicted	0.7	0.02811762	0.092237458
1388598_at	hypothetical protein LOC691995	LOC691995	0.6	0.028186513	0.092352942
1388795_at	Transcribed locus	---	0.8	0.028237475	0.092483071
1372616_at	tripartite motif protein 36 (predicted)	Trim36_predicted	0.6	0.028249136	0.09248982
1373512_at	ilvB (bacterial acetolactate synthase)-like (predicted)	Ilvbl_predicted	0.6	0.028329141	0.092654147
1373208_at	Transcribed locus	---	0.6	0.028351919	0.092692973
1385503_at	similar to O-acetyltransferase	LOC678772	0.8	0.028415276	0.092806684
1381986_at	neuron navigator 1 (predicted) /// similar to neuron navigator 1	LOC685707 /// Nav1_predicted	0.6	0.028423107	0.092813831
1371115_at	protein tyrosine phosphatase, receptor type, E	Ptpre	0.8	0.028443917	0.092852485
1371877_at	similar to MYLE protein (Dexamethasone-induced protein) (predicted)	RGD1564938_predicted	0.6	0.028464792	0.092852485
1372899_at	Solute carrier family 9 (sodium/hydrogen exchanger), member 8	Slc9a8	0.8	0.028465007	0.092852485
1397596_at	tripartite motif protein 2	Trim2	0.4	0.028468532	0.092852485
1377638_at	Bwk1 leukemia-related gene	Bwk1	0.8	0.028468816	0.092852485
1385191_at	Transcribed locus	---	0.7	0.028488863	0.092899448
1369886_a_at	calcium binding protein 1	Cabp1	0.5	0.028511827	0.092917696
1371609_at	homolog of zebrafish ES1	RGD1303003	0.7	0.028532981	0.092932765
1377028_at	Transcribed locus	---	0.8	0.028564681	0.093017595
1367521_at	---	---	0.8	0.028760117	0.09343329
1385852_at	CREB binding protein	Crebbp	0.6	0.028786622	0.093499677
1382118_at	Transcribed locus, strongly similar to NP_001029332.1 protein LOC619476 [Rattus norvegicus]	---	0.8	0.028795485	0.093509996
1372751_at	Transcribed locus, strongly similar to XP_580137.1 hypothetical protein XP_580137 [Rattus norvegicus]	---	0.7	0.028861389	0.093650055
1390144_at	similar to RIKEN cDNA 3110043O21	RGD1359108	0.7	0.028889057	0.093691704
1372724_at	glutamate receptor, ionotropic, N-methyl D-asparate-associated protein 1 (glutamate binding)	Grina	0.4	0.028957578	0.093888084
1389116_at	myotubularin related protein 9	Mtmr9	0.6	0.028969585	0.093893609
1387427_at	RAD50 homolog (S. cerevisiae)	Rad50	0.7	0.028977185	0.093896128
1398417_at	feminization 1 homolog a (C. elegans)	Fem1a	0.7	0.029019318	0.093995618
1368114_at	fibroblast growth factor 13	Fgf13	0.7	0.02902609	0.093999042
1373780_at	tetraspanin 1	Tspan1	0.6	0.029036252	0.09401344
1369405_a_at	cholinergic receptor, nicotinic, beta polypeptide 4	Chrnb4	0.7	0.029066193	0.094073345

1372458_at	similar to SET domain-containing protein	LOC498178 /// LOC687538 /// LOC689820	0.7	0.029072557	0.094075432
1378343_at	tuftelin interacting protein 11	Tfip11	0.8	0.029113548	0.094189542
1389476_at	similar to RIKEN cDNA D630029K19	RGD1307100	0.7	0.02918655	0.094388591
1371876_at	tumor necrosis factor superfamily, member 5-induced protein 1 (predicted)	Tnfsf5ip1_predicted	0.8	0.029224925	0.094469205
1382655_at	Transcribed locus	---	0.7	0.029235637	0.094469205
1382104_at	---	---	0.7	0.029259509	0.094510419
1383471_at	similar to RIKEN cDNA 1200006F02	RGD1307594	0.6	0.02927899	0.094538836
1388595_at	DEAH (Asp-Glu-Ala-His) box polypeptide 30	Dhx30	0.6	0.029338488	0.094675193
1394433_at	katanin p60 subunit A-like 1	Katnal1	0.6	0.029382875	0.094777665
1372636_at	Rab40c, member RAS oncogene family	Rab40c	0.8	0.02938753	0.094777665
1397414_at	---	---	0.7	0.029406209	0.094819316
1367462_at	calpain, small subunit 1	Capns1	0.3	0.029429857	0.094858317
1391071_at	Transcribed locus, weakly similar to XP_001166661.1 synaptic vesicle glycoprotein 2 isoform 1 [Pan troglodytes]	---	0.7	0.029431974	0.094858317
1374844_at	RNA binding motif protein 14	Rbm14	0.6	0.029450722	0.094888439
1393909_at	similar to KIAA1841 protein (predicted)	RGD1305110_predicted	0.7	0.029456692	0.094889084
1386973_a_at	mitogen activated protein kinase 8 interacting protein	Mapk8ip	0.7	0.029462809	0.094890205
1394117_at	Transcribed locus, moderately similar to XP_001167874.1 damage-specific DNA binding protein 2 (48kD) isoform 5 [Pan troglodytes]	---	0.8	0.029510194	0.0950054
1367520_at	apolipoprotein A-I binding protein (predicted)	Apoa1bp_predicted	0.6	0.029510623	0.0950054
1371388_at	pyruvate dehydrogenase (lipoamide) beta	Pdhb	0.7	0.029515904	0.0950054
1374161_at	similar to importin 11	LOC314407 /// LOC683400 /// LOC683676	0.6	0.029528034	0.095025846
1383704_at	similar to actin-related protein 3-beta (predicted)	RGD1565759_predicted	0.7	0.029548639	0.095073555
1387388_at	calcium binding protein p22 /// similar to calcium binding protein P22 (predicted)	Chp /// RGD1564956_predicted /// RGD1565588_predicted	0.6	0.029646964	0.095282895
1379900_at	Transcribed locus	---	0.8	0.029649291	0.095282895
1379654_at	GIY-YIG domain containing 2	Giyd2	0.6	0.029653227	0.095282895
1377047_at	Transcribed locus, strongly similar to XP_219005.3 similar to tRNA nucleotidyl transferase, CCA-adding, 1 [Rattus norvegicus]	---	0.7	0.029678631	0.095323984
1399049_at	similar to RIKEN cDNA 1200003C05 (predicted)	RGD1308917_predicted	0.8	0.029751902	0.095468091
1389221_at	monocyte to macrophage differentiation-associated 2 (predicted)	Mmd2_predicted	0.7	0.029752519	0.095468091
1394700_at	Interleukin 12 receptor, beta 2	Il12rb2	0.7	0.029784356	0.095532974
1387962_at	zinc finger protein 180	Zfp180	0.6	0.029804461	0.095576323
1371374_at	MAF1 homolog (S. cerevisiae)	Maf1	0.5	0.029862283	0.095708268
1390330_at	Transmembrane protein 60 (predicted)	Tmem60_predicted	0.8	0.029905745	0.095810226
1372557_at	ADP-ribosylation factor-like 6 (predicted)	Arl6_predicted	0.8	0.029932028	0.095819958
1389788_at	similar to hypothetical protein	RGD1307336	0.6	0.029932085	0.095819958
1389251_at	nudix (nucleoside diphosphate linked moiety X)-type motif 7 (predicted)	Nudt7_predicted	0.7	0.029979897	0.095898357

Supplementary table 5: Comparison of microarray and known regulation data for genes whose expression pattern within the DRG following nerve injury has been studied previously

Gene Title	Gene Symbol	SNT	gp120+ ddC	Valder et al, 2003	Wang et al, 2002	Probe Set ID
**Cell-cell communication and signalling related genes**						
adenosine kinase	Adk			↓ (2-3)		1369629_at
ATPase, Na+/K+ transporting, beta 3 polypeptide/Na K-ATPase beta-3 subunit	Atp1b3	↓ (1.6)		↓ (2-3)		1398300_at
Ca<2+-dependent activator protein for secretion	Cadps	↓ (1.6)		↓ (2-3)		1368523_at
calcium/calmodulin-dependent protein kinase II gamma	Camk2g	↓ (2.1)		↓ (2-3)		1369993_at
creatine kinase, mitochondrial 1, ubiquitous	Ckmt1	↓ (3.1)		↓ (2-4)	↓ (2.0)	1390566_a_at
diacylglycerol kinase zeta	Dgkz	↓ (2.8)		↓ (3-5)		1367745_at
mitogen activated protein kinase kinase kinase 12	Map3k12 /MUK	↓ (1.5)		↓ (2-5)		1368886_at
ATPase, Na+/K+ transporting, alpha 1 polypeptide truncated isoform	Nkaa1b	↓ (4.6)		↓ (3-34)		1371108_a_at
phosphofructokinase, platelet	Pfkp			↓ (2-4)		1372182_at
protein phosphatase 1, regulatory (inhibitor) subunit 1A	Ppp1r1a			↓ (3-5)		1386968_at
protein phosphatase 1, regulatory (inhibitor) subunit 2	Ppp1r2	↓ (2.6)		↓ (2-15)		1383180_at
protein phosphatase 2 (formerly 2A), regulatory subunit A (PR 65), alpha isoform	Ppp2r1a			↓ (2-3)		1375245_at
protein kinase C, epsilon	Prkce	↓ (1.85)	↑ (1.2)	↓ (2-11)		1374593_at
putative protein kinase C regulatory protein	Ywhag			↓ (2-4)		1386866_at
**Cell cycle / cell death related**						
cyclin D1	Ccnd1	↑ (1.9)			↑ (2.0-2.1)	1371150_at
cyclin D2	Ccnd2	↑ (2.7)			↑ (2.0)	1370810_at
cyclin D3	Ccnd3				↑ (2.0-2.2)	1369935_at
chemokine (C-X-C motif) ligand 14/NADH dehydrogenase	Cxcl14	↑ (5.1)			↑ (7.8-37.5)	1388485_at
DNA synthesis-dihydrofolate reductase	Dhfr	↑ (3.9)			↑ (3.3)	1383536_at
growth arrest and DNA-damage-inducible 45 alpha	Gadd45a	↑ (2.8)		↑ (3-12)	↑ (3.5-3.8)	1368947_at
DNA replication protein	Mcmd6				↑ (2.0)	1371074_a_at
**immediate early genes and transcription factors**						
activating transcription factor 3	Atf3	↑ (8.4)		↑ (7-15)	↑ (8.3)	1369268_at
paired related homeobox protein-like 1	DRG11/ Prrxl1	↓ (3.5)		↓ (2-8)		1388012_at
early growth response 1	Egr1/ NGFI-A	↑ (2.4)			↑ (2.1-2.4)	1368321_at
POU domain, class 3, transcription factor	Pou3f1	↑ (4.0)			↑ (2.8)	1370432_at

1/transcriptional repressor of myelin-specific genes						
RAB3A, member RAS oncogene family	Rab3a	↓ (2.2)		↓ (3-4)	↓ (2.8)	1369816_at
stress-induced-phosphoprotein 1/heat shock protein 70/90 organising protein	Stip1			↓ (3-5)		1398877_at
activity and neurotransmitter-induced early gene protein 4 (ania-4)	Ania4	↑ (2.0)		↑ (3-4)		1387276_at
**ion channel / ion pump**						
ATPase, Na+/K+ transporting, alpha 1 polypeptide/Na, K-ATPase alpha-1 subunit	Atp1a1	↓ (3.4)		↓ (4-11)	↓ (2.2)	1367585_a_at
ATPase, Ca++ transporting, plasma membrane 3	Atp2b3	↓ (1.9)			↓ (2.1)	1388037_at
calcium channel, voltage-dependent, alpha2/delta subunit 1	Cacna2d 1	↑ (2.7)		↑ (3-6)	↑ (2.9)	1369649_at
cholinergic receptor, nicotinic, alpha polypeptide 3	Chrna3	↓ (4.6)		↓ (4-32)	↓ (2.2)	1369001_at
glutamate receptor, ionotropic, kainate 1	Grik1	↓ (3.7)			↓ (2.3)	1387309_a_at
5-hydroxytryptamine (serotonin) receptor 3a	Htr3a	↓ (3.55)		↓ (10-79)	↓ (2.5-4.4)	1369428_a_at
potassium voltage-gated channel, shaker-related subfamily, member 4	Kcna4/ Kv1.4	↓ (3.0)			↓ (2.3)	1369043_at
potassium voltage gated channel, Shal-related family, member 3	Kcnd3/ Kv4.3	↓ (5.1)			↓ (3.5)	1369144_a_at
potassium voltage-gated channel, delayed-rectifier, subfamily S, member 3	Kcns3/ Kv9.3	↓ (3.2)		↓ (3-48)	↓ (2.4)	1368751_at
sodium channel, voltage-gated, type 10, alpha polypeptide	Scn10a/ Nav1.8	↓ (4.1)		↓ (3-5)	↓ (2.8)	1368351_at
sodium channel, voltage-gated, type11, alpha polypeptide	Scn11a/ Nav1.9	↓ (4.35)		↓ (4-60)	↓ (3.6)	1368768_at
sodium channel, voltage-gated, type 1, alpha polypeptide	Scn1a/ Nav1.1	↓ (2.3)		↓ (23-26)	↓ (2.2)	1369210_at
solute carrier family 24 (sodium/potassium/calcium exchanger), member 2	Slc24a2	↓ (3.1)			↓ (2.2)	1388000_at
**neuroinflammation and immune activation**						
alpha-2-macroglobulin	A2m	↑ (2.6)			↑ (2.3)	1367794_at
a disintegrin and metalloproteinase domain 17/TNF converting	Adam17	↑ (1.8)			↑ (2.2)	1367922_at
allograft inflammatory factor 1	Aif1	↑ (8.9)		↑ (3-6)	↑ (3.1)	1368558_s_at
complement component 1, q subcomponent, beta polypeptide	C1qb			↑ (8-69)	↑ (6.5-9.0)	1370215_at
complement component 1, r subcomponent (predicted)	C1r_ predicted	↑ (3.5)			↑ (2.9)	1383241_at
complement component 3	C3	↑ (6.4)			↑ (2.3-2.7)	1368000_at
complement component 4a /// complement	C4a /// C4-2	↑ (4.25)		↑ (7-26)	↑ (3.1)	1370892_at

component 4, gene 2						
CD24 antigen	Cd24			↓ (3-4)		1369953_a_at
CD74 antigen /MHCII(IA)-associated invariant chain	Cd74	↑ (6.0)		↑ (9-21)	↑ (5.5-7.4)	1367679_at
Fc receptor, IgG, low affinity III /// Fc gamma receptor II beta	Fcgr3	↑ (2.9)			↑ (3.2-4.0)	1398246_s_at
guanylate nucleotide binding protein 2	Gbp2	↑ (3.5)			↑ (3.6)	1368332_at
interferon induced transmembrane protein 3	Ifitm3	↑ (2.4)		↑ (2-4)	↑ (2.1)	1387995_a_at
interleukin 18	Il18				↑ (2.6)	1369665_a_at
lectin, galactose binding, soluble 3/IgE binding protein	Lgals3	↑ (1.7)		↑ (2-3)		1386879_at
Fc gamma receptor II beta	LOC498 276	↑ (5.8)			↑ (3.2-4.0)	1367850_at
major histocompatibility complex, class II, DM beta	MHCII M-beta	↑ (3.2)			↑ (4.3)	1370882_at
pancreatitis-associated protein	Pap	↑ (22.8)	↑ (1.2)	↑ (2-17)	↑ (4.6)	1368238_at
RT1 class II, locus Ba/MHC RT1-B region class II (Ia antigen) A-alpha glycoprotein	RT1-Ba	↑ (2.85)		↑ (5-7)	↑ (5.7)	1370822_at
RT1 class II, locus Bb/MHC class II antigen RT1.B-1 beta-chain	RT1-Bb	↑ (6.9)		↑ (3-6)	↑ (5.2-6.2)	1371033_at
RT1 class II, locus Bb/MHC class II antigen RT1.B beta-chain	RT1-Bb			↑ (14-97)	↑ (5.2-6.2)	1371065_at
RT1 class II, locus Da/MHC class II RT1.u-D-alpha chain	RT1-Da	↑ (4.8)		↑ (6-21)	↑ (5.4-7.0)	1370883_at
RT1 class II, locus Db1/RT1.D beta chain	RT1-Db1	↑ (3.55)		↑ (3-79)	↑ (5.7-6.5)	1370382_at
serine (or cysteine) proteinase inhibitor, clade G (C1 inhibitor), member 1, (angioedema, hereditary)	Serping1	↑ (2.9)			↑ (2.3)	1372254_at
**neuropeptides/neurotransmission**						
adenylate cyclase activating polypeptide 1	Adcyap1/ PACAP	↑ (3.9)			↑ (2.3)	1368892_at
Adenylate cyclase activating polypeptide 1	Adcyap1/ PACAP	↑ (5.8)			↑ (2.3)	1394908_at
calcitonin/calcitonin-related polypeptide, alpha	Calca/ CGRP	↓ (2.2)		↓ (4-15)		1370775_a_at
calcitonin-related polypeptide, beta	Calcb	↓ (2.3)		↓ (3-18)		1370562_at
galanin	Gal	↑ (6.8)		↑ (16-42)	↑ (18.5)	1387088_at
islet amyloid polypeptide	Iapp				↓ (4.4)	1387660_at
neuropeptide Y	Npy	↑ (8.6)	↑ (1.9)	↑ (16-181)	↑ (45.6)	1387154_at
secretory granule neuroendocrine protein 1	Sgne1			↓ (2-3)		1367992_at
somatostatin	Sst	↓ (2.8)		↓ (9-42)	↓ (3.3-4.4)	1367762_at
tachykinin 1/substance P	Tac1	↓ (2.2)		↓ (3-10)	↓ (2.1)	1369309_a_at
vasoactive intestinal polypeptide	Vip	↑ (19.3)		↑ (10-64)	↑ (14.5)	1377146_at
**other**						
amyloid beta (A4) precursor protein-binding, family B, member 1	Apbb1/F E65	↓ (2.0)		↓ (2-3)		1367842_at
apolipoprotein B editing complex 1	Apobec1				↑ (2.7)	1368270_at

cytochrome c oxidase, subunit Va	Cox5a			↓ (2-4)		1370888_at
SH3 domain binding protein CR16	Cr16	↓ (2.2)	↑ (1.3)	↓ (3-12)		1370648_a_at
decay accelarating factor 1	Daf1	↓ (3.8)			↓ (2.3)	1370406_a_at
dynein, cytoplasmic, intermediate chain 1	Dncic1			↓ (2-4)		1387025_at
dynamin 1-like	Dnm1l			↓ (2-23)		1369220_at
dipeptidylpeptidase 6	Dpp6	↓ (3.9)		↓ (3-15)		1369390_a_at
Fatty acid binding protein 7, brain / Brain lipid-binding protein	Fabp7		↓ (1.3)		↓ (2.0)	1370024_at
glutathione peroxidase 1	Gpx1	↑ (1.4)		↑ (2-3)		1367576_at
glycogenin 1	Gyg1	↓ (1.5)			↓ (2.0)	1367900_at
Hexokinase 2	Hk2	↑ (1.9)			↑ (2.4)	1383519_at
high mobility group box 2	Hmgb2/ HMG2	↑ (3.6)		↑ (3-16)		1367676_at
leukocyte cell derived chemotaxin 1	Lect1/ Chm-1	↓ (1.8)		↓ (2-5)		1387164_at
lin-7 homolog b (C. elegans)	Lin7b	↓ (3.2)			↓ (2.3)	1370078_at
lumican	Lum	↑ (2.7)		↑ (2-6)	↑ (2.2)	1367749_at
lysozyme	Lysz	↑ (5.5)			↑ (3.0)	1370154_at
malate dehydrogenase 1, NAD (soluble)	Mdh1			↓ (2-3)		1367653_a_at
matrix metallopeptidase 2/gelatinase	Mmp2	↑ (2.3)			↑ (2.1)	1370301_at
myocilin	Myoc	↑ (2.5)		↑ (2-3)		1387313_at
NCK-associated protein 1	Nckap1/ Nap1	↓ (1.5)		↓ (2-8)		1388196_at
proteosome (prosome, macropain) subunit, beta type 9	Psmb9	↑ (3.4)			↑ (2.2)	1370186_at
parvalbumin	Pvalb	↓ (3.1)			↓ (2.1)	1370214_at
poliovirus receptor/Tumor-associated glycoprotein E4	PVR	↑ (5.4)			↑ (2.0)	1370177_at
cysteine-rich protein 2	RGD:130 2959	↓ (1.8)		↓ (2-3)		1367604_at
LPS-induced TNF-alpha factor/Estrogen responsive uterine RNA	RGD:692 94	↑ (2.2)			↑ (2.2)	1370928_at
major histocompatibility complex, class II, DM alpha	RT1.Ma	↑ (2.7)		↑ (2-13)		1370904_at
secretogranin 2	Scg2	↓ (2.9)		↓ (4-14)		1368044_at
steroid 5-alpha-reductase 2 / Xenosine dehydrogenase/oxidase	Srd5a2				↑ (2.8)	1387580_at
transglutaminase 1	Tgm1	↑ (3.0)			↑ (3.8)	1370051_at
transmembrane 4 superfamily member 3	Tm4sf3/ D6.1 A	↓ (1.6)		↓ (3-4)	↓ (2.2)	1368052_at
xanthine dehydrogenase / Xenosine dehydrogenase/oxidase	Xdh	↑ (2.4)			↑ (2.8)	1369973_at
ATPase, H+ transporting, V1 subunit B, isoform 2	Atp6v1b2	↓ (3.9)		↓ (2-10)		1387664_at
benzodiazepin receptor	Bzrp	↑ (5.3)		↑ (3-7)	↑ (3.2)	1370249_at
calcium-sensing receptor/Kidney extracellular calcium-sensing receptor	Casr	↓ (3.9)	↑ (1.4)	↓ (7-20)		1369158_at
ceroid-lipofuscinosis, neuronal 6 (predicted) / calmodulin homolog	Cln6_pre dicted	↓ (3.6)			↓ (3.1)	1375026_at

chemokine (C-X-C motif) ligand 10/ras activating protein	Cxcl10	↑ (7.5)			↑ (2.7)	1387969_at
dual specificity phosphatase 6	Dusp6	↑ (2.4)			↑ (2.0)	1377064_at
GTP cyclohydrolase 1	Gch	↑ (5.4)			↑ (2.8)	1387221_at
glial cell line derived neurotrophic factor family receptor alpha 1	Gfra1/ GDNFR1	↑ (2.5)			↑ (2.2)	1367954_at
guanine nucleotide binding protein, alpha o/G-alpha-0	Gnao	↓ (2.6)		↓ (2-3)	↓ (2.1)	1368879_a_at
similar to Tescalcin / Calcium binding protein	LOC288 689	↓ (2.2)			↓ (2.3)	1384202_at
NEL-like 1 (chicken)/protein kinace C-binding protein Nel-homolog protein	Nell1	↓ (2.0)		↓ (4-5)	↓ (2.4)	1368120_at
neurotrophic tyrosine kinase, receptor, type 1	Ntrk1	↓ (3.6)		↓ (3)		1369000_at
purinergic receptor P2X, ligand-gated ion channel, 3	P2rx3	↓ (1.7)		↓ (2-3)		1368667_at
phospholipase C, delta 4	Plcd4	↓ (4.3)		↓ (2-24)	↓ (2.8)	1387065_at
protein tyrosine phosphatase, non-receptor type 5/striatum-enriched phosphatase	Ptpn5	↑ (4.6)			↑ (3.3)	1368421_at
protein tyrosine phosphatase, receptor type, O/Tyr phosphatase D30	Ptpro	↑ (2.5)			↑ (2.0)	1368412_a_at
regulator of G-protein signaling 4	Rgs4	↓ (2.75)		↓ (10-15)	↓ (2.7)	1368505_at
S100 calcium-binding protein A4/protein p9Ka homologous to calcium binding protein	S100a4	↑ (2.2)		↑ (3-5)	↑ (2.0)	1367846_at
Transferrin receptor	Tfrc	↓ (1.55)			↓ (2.1)	1388750_at
visinin-like 1	Vsnl1/ NVP	↓ (3.6)		↓ (4-37)	↓ (2.1)	1368853_at
**related to growth and synaptogenesis**						
acyl-CoA synthetase long-chain family member 4	Acsl4	↑ (2.1)		↑ (3-7)		1387101_at
amyloid beta (A4) precursor-like protein 2	Aplp2/Y WK-II	↓ (3.2)		↓ (2-3)		1388121_at
apolipoprotein D	ApoD	↑ (3.2)		↑ (3-8)	↑ (2.4)	1398258_at
arginase 1	Arg1	↑ (18.0)		↑ (11-17)	↑ (4.2)	1368266_at
actin related protein 2/3 complex, subunit 1B	Arpc1b/ p41-Arc			↑ (2-3)	↑ (2.4-2.6)	1386925_at
asialoglycoprotein receptor 2/hepatic lectin	Asgr2				↑ (2.1)	1368362_a_at
biglycan	Bgn			↑ (2-3)		1367594_at
B-cell translocation gene 1, anti-proliferative	Btg1	↑ (1.8)		↑ (2-3)	↑ (2.2)	1367657_at
complement component 1, s subcomponent/ serine protease	C1s	↑ (3.8)		↑ (3-12)	↑ (3.7)	1387893_at
carbonic anhydrase 2	Ca2	↓ (1.9)			↓ (2.0)	1367733_at
collagen, type 1, alpha 1	Col1a1			↑ (2-3)		1370864_at
collagen, type III, alpha 1	Col3a1	↑ (1.8)		↑ (3-6)		1370959_at
Collagen, type V, alpha 2	Col5a2	↑ (2.8)			↑ (2.4)	1373463_at
cellular retinoic acid binding protein 2/Retinoid acid-binding protein	Crabp2	↑ (4.4)		↑ (4-5)	↑ (3.5)	1370391_at
cysteine-rich secretory	Crisp1	↑ (24.0)			↑ (6.0)	1368633_at

protein 1/epididymal glycoprotein						
cold shock domain protein A	Csda/ RYB-a			↑ (2-4)		1370376_a_at
cysteine and glycine-rich protein 2	Csrp2/ SmLIM	↑ (3.0)		↑ (2-4)		1370282_at
cysteine-rich protein/muscle LIM protein	Csrp3	↑ (16.7)		↑ (3-13)	↑ (4.2-5.0)	1398243_at
cathepsin S	Ctss	↑ (6.9)		↑ (3-9)	↑ (4.0)	1387005_at
decorin	Dcn	↑ (2.2)		↑ (3-5)		1370956_at
dihydropyrimidinase-like 4 / Collapsin response mediating protein 3	Dpysl4/ CRMP-3	↓ (2.3)		↓ (4-42)	↓ (3.0)	1371049_at
fatty acid binding protein 3/low molecular weight fatty acid binding protein	Fabp3	↓ (2.2)		↓ (2-11)	↓ (2.4)	1367660_at
Fas apoptotic inhibitory molecule 2/neural membrane protein 35	Faim2	↓ (5.05)		↓ (3-4)		1370625_at
fasciculation and elongation protein zeta 1 (zygin I)	Fez1	↓ (2.0)		↓ (3-5)		1367918_at
fibroblast growth factor 9	Fgf9	↓ (2.8)		↓ (2-3)		1370747_at
fibronectin 1	Fn1	↑ (3.4)		↑ (3-9)	↑ (2.3-2.4)	1370234_at
growth associated protein 43	Gap43	↑ (1.9)		↑ (2-5)	↑ (2.2)	1367930_at
growth arrest specific 7	Gas7	↑ (2.1)		↑ (2-3)	↑ (2.3)	1370963_at
glial fibrillary acidic protein	Gfap	↑ (2.6)		↑ (3-7)	↑ (6.1)	1368353_at
glypican 3/Developmentally regulated protein	Gpc3/ OCI-5			↑ (3-13)	↑ (2.2)	1368395_at
H19 fetal liver mRNA	H19/ ASM15	↑ (3.4)		↑ (9-18)		1371298_at
insulin-like growth factor binding protein 3	Igfbp3	↑ (3.0)			↑ (2.0)	1386881_at
insulin-like growth factor binding protein 6	Igfbp6	↑ (1.69)		↑ (2-4)	↑ (2.0)	1372168_s_at
insulin induced gene 1	Insig1	↓ (1.7)		↓ (2-3)		1367894_at
insulin related protein 2/islet-2	Isl2	↓ (3.3)		↓ (5-10)		1369530_at
lactate dehydrogenase B	Ldhb			↓ (2-3)		1370218_at
lipase A, lysosomal acid	Lipa	↓ (1.48)		↓ (2-3)		1368075_at
lysyl oxidase	Lox	↑ (3.1)		↑ (3-21)		1368172_a_at
microtubule-associated protein tau	Mapt		↑ (1.4)	↓ (2)		1368137_at
macrophage galactose N-acetyl-galactosamine specific lectin 1	Mgl1	↑ (4.00)			↑ (2.3)	1368464_at
matrix metalloproteinase 16/MT3-MMP-del	Mmp16	↑ (4.7)			↑ (2.2)	1368590_at
Matrix metalloproteinase 23	Mmp23/ MMP-11				↑ (2.1)	1368961_at
myosin light chain, regulatory B	Mrlcb	↑ (2.2)		↑ (2-3)		1388114_at
N-myc downstream regulated 4/development-related protein	Ndr4			↓ (2-3)		1370229_at
neurofilament 3, medium	Nef3	↓ (1.4)		↓ (5-6)		1367845_at
neuronal pentraxin 1	Nptx1	↓ (4.05)			↓ (2.0-2.5)	1370517_at
neuronal pentraxin II	Nptx2/ Narp	↓ (3.7)		↓ (2-3)		1373559_at
neuritin	Nrn1	↓ (2.9)		↓ (5-9)	↓ (2.7)	1386969_at
nuclear protein 1	Nupr1/p8	↑ (4.6)		↑ (3-8)		1367847_at
parathyroid hormone-like peptide	Pthlh/ PTHrP	↑ (1.6)			↑ (10.1)	1368681_at

retinol binding protein 1, cellular/Retinoid acid-binding protein CRBP	Rbp1	↑ (4.7)		↑ (2-6)	↑ (3.1)	1367939_at
neurofilament, light polypeptide	RGD:621458	↓ (1.7)		↓ (4-7)		1370058_at
syndecan 1 / heparan sulpfate proteoglycan core protein	Sdc1	↑ (4.7)			↑ (2.4)	1367849_at
serine (or cysteine) proteinase inhibitor, clade H, member 1	Serpinh1/gp46			↑ (2-16)		1369009_at
SH3 domain protein 2A	Sh3gl2/SH3p4			↓ (5-11)		1396464_at
synaptosomal-associated protein 25	SNAP-25B	↓ (1.5)			↓ (2.2)	1387073_at
similar to Cornifin alpha (Small proline-rich protein 1)	Sprr1a	↑ (19.5)			↑ (3.3-7.0)	1371248_at
stathmin-like 4	Stmn4	↑ (4.0)		↑ (2-3)	↑ (3.2)	1370315_a_at
synaptic vesicle glycoprotein 2b	Sv2b	↓ (1.6)	↓ (2.3)	↓ (11-26)	↓ (2.0)	1369627_at
synapsin II	Syn2			↓ (3-5)		1370431_at
synaptophysin/major synaptic vesicle protein p38	Syp	↓ (2.25)		↓ (2-3)		1368276_at
thrombospondin 4	Thbs4	↑ (3.8)		↑ (3-20)	↑ (2.9)	1388138_at
tissue inhibitor of metalloproteinase 1	Timp1	↑ (3.2)			↑ (2.3)	1367712_at
urocortin / corticotensin	Ucn				↑ (2.3)	1387645_at
UDP glycoronosyltransferase	Ugt1	↑ (2.6)		↑ (2-30)	↑ (2.5-3.1)	1370613_s_at
vesicle-associated membrane protein 1	Vamp1	↓ (5.2)		↓ (2-23)	↓ (2.0-2.5)	1370556_at
VGF nerve growth factor inducible	Vgf	↑ (3.45)	↑ (1.3)	↑ (2-3)	↑ (2.6)	1368359_a_at
vimentin	Vim			↑ (2-4)		1367574_at

Supplementary Table 6: Functional categories of genes highlighted by MAPPFinder for the SNT model

GO Name	GO Type	% genes up-regulated compared to genes present in array	% genes measured in the array compared to GP genes at this term	Z Score	Permute P
		**Up-regulated**			
structural constituent of ribosome	F	58.4	39.2	10.98	0
immune response	P	45.8	42.9	7.66	0
antigen processing and presentation	P	66.7	43.5	6.43	0
immunoglobulin mediated immune response	P	70.0	44.4	5.92	0
antigen processing and presentation of peptide antigen	P	72.2	75.0	5.86	0
defense response	P	39.4	39.6	5.65	0
antigen processing and presentation of exogenous antigen	P	76.9	81.3	5.41	0
antigen processing and presentation of exogenous peptide antigen via MHC class II	P	81.8	84.6	5.39	0
antigen processing and presentation of peptide or polysaccharide antigen via MHC class II	P	81.8	73.3	5.39	0

cytokine activity	F	44.0	34.2	4.64	0
regulation of immune response	P	44.4	46.4	4.47	0
acute inflammatory response	P	48.5	47.8	4.43	0
response to wounding	P	33.6	50.2	4.36	0
positive regulation of immune response	P	45.0	49.4	4.31	0
innate immune response	P	66.7	34.3	4.28	0
rRNA binding	F	66.7	80.0	4.28	0.001
inflammatory response	P	37.2	45.6	4.25	0
complement activation\, classical pathway	P	83.3	35.3	4.08	0
cytokine production	P	46.7	42.3	3.96	0
double-stranded DNA binding	F	56.3	80.0	3.88	0
phosphate transport	P	45.2	52.5	3.81	0
T cell differentiation	P	52.9	37.8	3.65	0.001
response to biotic stimulus	P	34.7	54.7	3.60	0
peptide antigen transport	P	71.4	70.0	3.60	0.002
acute-phase response	P	71.4	35.0	3.60	0.001
peptide antigen-transporting ATPase activity	F	71.4	70.0	3.60	0.002
neutrophil chemotaxis	P	80.0	38.5	3.53	0.006
response to virus	P	53.3	68.2	3.46	0.003
hemopoiesis	P	34.2	48.0	3.46	0.001
defense response to bacterium	P	60.0	26.3	3.37	0.006
extracellular matrix organization and biogenesis	P	45.5	57.9	3.24	0.001
caspase activity	F	62.5	61.5	3.19	0.009
cell activation	P	33.3	39.9	3.17	0.001

cysteine-type endopeptidase activity	F	42.3	48.1	3.12	0.006
carbohydrate binding	F	32.1	34.2	3.07	0.003
T cell selection	P	66.7	42.9	3.03	0.009
response to ionizing radiation	P	66.7	54.5	3.03	0.009
cytokine binding	F	50.0	26.4	3.02	0.007
myeloid cell differentiation	P	37.8	49.3	3.02	0.001
cell proliferation	P	26.6	52.0	2.96	0.007
embryonic development	P	28.3	51.1	2.95	0.004
T cell receptor signaling pathway	P	55.6	56.3	2.85	0.006
urogenital system development	P	44.4	40.9	2.82	0.007
leukocyte migration	P	50.0	48.0	2.80	0.008
transcription factor activity	F	25.8	40.1	2.63	0.003
negative regulation of cell proliferation	P	31.3	52.9	2.62	0.009
	**Down-regulated**				
cation channel activity	F	47.1	45.3	7.80	0
ion channel activity	F	41.3	45.8	7.63	0
potassium channel activity	F	55.3	48.5	7.24	0
potassium ion transport	P	52.9	45.9	7.08	0
potassium ion binding	F	58.3	47.4	6.82	0
voltage-gated ion channel activity	F	46.2	48.5	6.52	0
voltage-gated potassium channel activity	F	54.3	46.7	6.08	0
exocytosis	P	47.1	69.9	5.94	0
metal ion transport	P	34.8	45.4	5.91	0
cation transporter activity	F	31.2	49.9	5.72	0
cation transport	P	30.7	47.7	5.20	0

neurological process	P	29.6	45.0	5.08	0
axonogenesis	P	39.7	56.3	5.02	0
cell-cell signaling	P	30.5	60.1	5.01	0
neurotransmitter secretion	P	48.5	78.6	5.00	0
cellular morphogenesis during differentiation	P	37.2	58.2	4.99	0
neuron differentiation	P	31.3	58.0	4.66	0
G-protein coupled receptor protein signaling pathway	P	30.3	9.0	4.60	0
transmission of nerve impulse	P	30.5	66.5	4.58	0
neurite development	P	34.1	59.9	4.52	0
regulation of axonogenesis	P	55.6	66.7	4.50	0
synaptic transmission	P	30.8	67.2	4.25	0
homophilic cell adhesion	P	42.9	39.3	4.24	0
extracellular ligand-gated ion channel activity	F	46.2	44.1	4.11	0
generation of neurons	P	28.4	58.5	4.09	0
synaptic vesicle transport	P	50.0	87.0	4.07	0.001
MAPKKK cascade	P	36.4	51.9	4.02	0
diacylglycerol kinase activity	F	71.4	58.3	3.94	0.004
synaptogenesis	P	58.3	92.3	3.93	0.001
regulation of exocytosis	P	53.3	88.2	3.87	0
glutamate signaling pathway	P	80.0	55.6	3.85	0.002
voltage-gated sodium channel activity	F	80.0	50.0	3.85	0.001
regulation of axon extension	P	60.0	66.7	3.73	0.002
negative regulation of neurogenesis	P	60.0	66.7	3.73	0.001

vesicle-mediated transport	P	26.3	72.4	3.73	0
secretion	P	27.5	65.9	3.72	0
cAMP-mediated signaling	P	45.5	44.9	3.69	0.004
cell-cell adhesion	P	32.0	47.2	3.68	0
sodium ion binding	F	41.4	42.6	3.65	0.001
vesicle targeting	P	62.5	100.0	3.53	0.006
embryonic limb morphogenesis	P	45.0	44.4	3.46	0.002
synaptic vesicle exocytosis	P	54.5	91.7	3.42	0.005
G-protein signaling\, adenylate cyclase activating pathway	P	66.7	25.0	3.33	0.009
negative regulation of axonogenesis	P	66.7	66.7	3.33	0.009
phosphotyrosine binding	F	66.7	66.7	3.33	0.008
embryonic morphogenesis	P	32.7	44.7	3.29	0.001
cell maturation	P	37.5	52.5	3.24	0.003
JNK cascade	P	44.4	56.3	3.22	0.005
cytokinesis	P	55.6	90.0	3.18	0.008
phosphoprotein binding	F	55.6	60.0	3.18	0.008
regulation of heart contraction	P	46.7	55.6	3.17	0.007
synapse organization and biogenesis	P	40.9	81.5	3.11	0.003
GTP binding	F	24.2	75.4	2.96	0.003
calcium ion-dependent exocytosis	P	46.2	76.5	2.90	0.006
sodium ion transport	P	34.3	39.8	2.87	0.007
small GTPase mediated signal transduction	P	23.9	71.2	2.84	0.007
skeletal development	P	28.4	47.1	2.81	0.008
endocytosis	P	29.5	64.9	2.78	0.009

carboxylic ester hydrolase activity	F	31.0	46.2	2.56	0.006

Supplementary table 7: Gene sets associated with gene up-regulation in SNT using Gene Set Enrichment Analysis

Gene set name	Source	Size	NOM p-val	FDR q-val
RIBOSOMAL_PROTEINS	GenMAPP	86	0	0
D4GDIPATHWAY	BioCarta	22	0	0
CYTOKINE_SIGNALING	NetAffx	213	0	0
MACROPHAGE_GENES	NetAffx	138	0	4.54E-04
CHEMOKINE_RECEPTORS	NetAffx	116	0	6.81E-04
COMPLEMENT_PATHWAY	BioCarta	16	0	0.001086
JECHLINGER_EMT_UP	Broad Institute	56	0	0.001926
HYPERTROPHY_MODEL	GenMAPP	32	0	0.012161
CASPASEPATHWAY	BioCarta	32	0	0.012678
DORSAM_HOXA9_DN	Broad Institute	33	0	0.015717
IL1RPATHWAY	BioCarta	35	0	0.016604
CELL_PROLIFERATION	GO	181	0	0.022541
NTHIPATHWAY	BioCarta	29	0	0.022855
BRENTANI_TRANSCRIPTION_FACTORS	Broad Institute	54	0	0.032373
PHENYLALANINE_METABOLISM	GenMAPP	24	0	0.039248
BRENTANI_DEATH	Broad Institute	73	0	0.057608
HINATA_NFKB_UP	Broad Institute	119	0	0.061267
PROLIFERATION_GENES	Broad Institute	385	0	0.06437
TGF_BETA_SIGNALING_PATHWAY	Broad Institute	76	0	0.064403
APOPTOSIS	GenMAPP	72	0.00159	0.059885
PPARAPATHWAY	BioCarta	81	0.001661	0.062156
APOPTOSIS_GENMAPP	GenMAPP	56	0.001733	0.06894
CELL_GROWTH_AND_OR_MAINTENANCE	GO	55	0.001736	0.016469
INFLAMMATORY_RESPONSE_PATHWAY	Broad Institute	29	0.001751	0.011817
IL12PATHWAY	BioCarta	20	0.001805	0.01518
LAIRPATHWAY	BioCarta	16	0.001815	0.02375
CELL_ADHESION	GO	178	0.00292	0.098306
BREAST_CANCER_ESTROGEN_SIGNALING	Gearray	118	0.00318	0.08811
BRENTANI_CELL_ADHESION	Broad Institute	123	0.00319	0.085128
DNAFRAGMENTPATHWAY	BioCarta	16	0.003597	0.021337
MRNA_PROCESSING	Broad Institute	45	0.005236	0.063732
MATRIX_METALLOPROTEINASES	Broad Institute	30	0.005338	0.058942
PORPHYRIN_AND_CHLOROPHYLL_METABOLISM	GenMAPP	18	0.005618	0.053267
BRENTANI_IMMUNE_FUNCTION	Broad Institute	34	0.00678	0.058494
CORDERO_KRAS_KD_VS_CONTROL_UP	Broad Institute	91	0.007899	0.08787
RACCYCDPATHWAY	BioCarta	37	0.008576	0.070361
G1_TO_S_CELL_CYCLE_REACTOME	GenMAPP	69	0.008606	0.103301
SA_MMP_CYTOKINE_CONNECTION	SigmaAldrich	23	0.008881	0.062922
SMITH_HTERT_UP	Broad Institute	98	0.009539	0.089285

Supplementary table 8: Gene sets associated with gene down-regulation in SNT using Gene Set Enrichment Analysis

Gene set name	Source	Size	NOM p-val	FDR q-val
GPCRS_CLASS_C_METABOTROPIC_GLUTAMATE_PHEROMONE	GO	22	0	0
CANNABINOID_RELATED	NetAffx	52	0	0
OPIOID_RECEPTOR_RELATED	NetAffx	108	0	0
GPCRDB_CLASS_C_METABOTROPIC_GLUTAMATE_PHEROMONE	GenMAPP	23	0	4.25E-04
RABPATHWAY	BioCarta	20	0	0.004319
GLYCOLYSIS_AND_GLUCONEOGENESIS	GenMAPP	53	0	0.004534
CHOLESTEROL_BIOSYNTHESIS	GenMAPP	21	0	0.005172
PAIN_RELATED	NetAffx	257	0	0.007622
GLUCONEOGENESIS	GenMAPP	62	0	0.076639
CALCIUM_REGULATION_IN_CARDIAC_CELLS	GenMAPP	243	0	0.176252
BIOSYNTHESIS_OF_STEROIDS	GenMAPP	19	0.002128	0.021544
BRENTANI_TRANSPORT_OF_VESICLES	Broad Institute	31	0.002278	0.050005
GLYCEROPHOSPHOLIPID_METABOLISM	KEGG	51	0.002299	0.036047
GPCRDB_CLASS_A_RHODOPSIN_LIKE	GenMAPP	180	0.002825	0.241339
PENTOSE_PHOSPHATE_PATHWAY	GenMAPP	18	0.004505	0.052498
GLYCOLYSIS	GenMAPP	62	0.004785	0.075994
S1P_SIGNALING	Broad Institute	34	0.007212	0.046803
GLYCEROLIPID_METABOLISM	KEGG	43	0.007229	0.080335
CARBON_FIXATION	KEGG	22	0.009132	0.068452
FATTY_ACID_SYNTHESIS	Broad Institute	26	0.009195	0.081248
